# Supplementary material for: Resolving the full spectrum of human genome variation using Linked-Reads
Source: Genome Res. 2019 Apr;29(4):635–45. doi: 10.1101/gr.234443.118 (PMC6442396; doi:10.1101/gr.234443.118)
Supplement: Supplemental Material [file supp_gr.234443.118_Supplemental_longranger-2.2.zip › longranger-2.2.tar.bz2!/lr2.2-public/lib/stan/cmdstan/stan/doc/stan-reference-2.5.0.pdf]

Stan Modeling Language

User's Guide and Reference Manual

Stan Development Team

Stan Version 2.5.0

Monday 20th October, 2014

http://mc-stan.org/

Stan Development Team. 2014. *Stan Modeling Language: User's Guide and Reference Manual.* Version 2.5.0

Copyright © 2011–2014, Stan Development Team.

This document is distributed under the Creative Commons Attribute 4.0 Unported License (CC BY 4.0). For full details, see

https://creativecommons.org/licenses/by/4.0/legalcode

Contents

Preface vi

Acknowledgements xi

I Introduction 13

1. Overview 14

II Programming Techniques 19

2. Model Building as Software Development 20
3. Data Types 26
4. Containers: Arrays, Vectors, and Matrices 29
5. Regression Models 33
6. Time-Series Models 59
7. Missing Data & Partially Known Parameters 77
8. Truncated or Censored Data 81
9. Finite Mixtures 86
10. Measurement Error and Meta-Analysis 91
11. Latent Discrete Parameters 96
12. Clustering Models 115
13. Gaussian Processes 128
14. Reparameterization & Change of Variables 141
15. Custom Probability Functions 147
16. User-Defined Functions 149
17. Solving Differential Equations 160
18. Problematic Posteriors 166
19. Optimizing Stan Code 180
20. Reproducibility 200

III Modeling Language Reference **201**
21. Execution of a Stan Program 202
22. Data Types and Variable Declarations 208
23. Expressions 225
24. Statements 241
25. User-Defined Functions 258
26. Program Blocks 266
27. Modeling Language Syntax 277

IV Built-In Functions **281**
28. Vectorization 282
29. Void Functions 284
30. Integer-Valued Basic Functions 286
31. Real-Valued Basic Functions 289
32. Array Operations 311
33. Matrix Operations 317
34. Mixed Operations 336

V Discrete Distributions **338**
35. Binary Distributions 339
36. Bounded Discrete Distributions 341
37. Unbounded Discrete Distributions 347
38. Multivariate Discrete Distributions 351

VI Continuous Distributions **352**
39. Unbounded Continuous Distributions 353
40. Positive Continuous Distributions 361
41. Non-negative Continuous Distributions 369
42. Positive Lower-Bounded Probabilities 370
43. Continuous Distributions on [0, 1] 372

44. Circular Distributions 374
45. Bounded Continuous Probabilities 376
46. Distributions over Unbounded Vectors 377
47. Simplex Distributions 384
48. Correlation Matrix Distributions 385
49. Covariance Matrix Distributions 388

VII Additional Topics 390
50. Point Estimation 391
51. Bayesian Data Analysis 401
52. Markov Chain Monte Carlo Sampling 405
53. Transformations of Constrained Variables 413

VIII Algorithms & Implementations 430
54. Hamiltonian Monte Carlo Sampling 431
55. Optimization Algorithms 441
56. Diagnostic Mode 444

IX Contributed Modules 446
57. Contributed Modules 447

Appendices 449
A. Licensing 449
B. Stan for Users of BUGS 451
C. Stan Program Style Guide 460
D. Warning and Error Messages 468
E. Mathematical Functions 470
Bibliography 471
Index 477

Preface

Why Stan?

We[1] did not set out to build Stan as it currently exists. We set out to apply full Bayesian inference to the sort of multilevel generalized linear models discussed in Part II of (Gelman and Hill, 2007). These models are structured with grouped and interacted predictors at multiple levels, hierarchical covariance priors, nonconjugate coefficient priors, latent effects as in item-response models, and varying output link functions and distributions.

The models we wanted to fit turned out to be a challenge for current general-purpose software to fit. A direct encoding in BUGS or JAGS can grind these tools to a halt. Matt Schofield found his multilevel time-series regression of climate on tree-ring measurements wasn't converging after hundreds of thousands of iterations.

Initially, Aleks Jakulin spent some time working on extending the Gibbs sampler in the Hierarchical Bayesian Compiler (Daumé, 2007), which as its name suggests, is compiled rather than interpreted. But even an efficient and scalable implementation does not solve the underlying problem that Gibbs sampling does not fare well with highly correlated posteriors. We finally realized we needed a better sampler, not a more efficient implementation.

We briefly considered trying to tune proposals for a random-walk Metropolis-Hastings sampler, but that seemed too problem specific and not even necessarily possible without some kind of adaptation rather than tuning of the proposals.

The Path to Stan

We were at the same time starting to hear more and more about Hamiltonian Monte Carlo (HMC) and its ability to overcome some of the the problems inherent in Gibbs sampling. Matt Schofield managed to fit the tree-ring data using a hand-coded implementation of HMC, finding it converged in a few hundred iterations.

[1]In Fall 2010, the "we" consisted of Andrew Gelman and his crew of Ph.D. students (Wei Wang and Vince Dorie), postdocs (Ben Goodrich, Matt Hoffman and Michael Malecki), and research staff (Bob Carpenter and Daniel Lee). Previous postdocs whose work directly influenced Stan included Matt Schofield, Kenny Shirley, and Aleks Jakulin. Jiqiang Guo joined as a postdoc in Fall 2011. Marcus Brubaker, a computer science postdoc at Toyota Technical Institute at Chicago, joined the development team in early 2012. Michael Betancourt, a physics Ph.D. about to start a postdoc at University College London, joined the development team in late 2012 after months of providing useful feedback on geometry and debugging samplers at our meetings. Yuanjun Gao, a statistics graduate student at Columbia, and Peter Li, an undergraduate student at Columbia, joined the development team in the Fall semester of 2012. Allen Riddell joined the development team in Fall of 2013 and is currently maintaining PyStan. In the summer of 2014, Marco Inacio (University of São Paulo), Mitzi Morris (independent contractor), and Jeffrey Arnold (University of Washington) joined the development team.

HMC appeared promising but was also problematic in that the Hamiltonian dynamics simulation requires the gradient of the log posterior. Although it's possible to do this by hand, it is very tedious and error prone. That's when we discovered reverse-mode algorithmic differentiation, which lets you write down a templated C++ function for the log posterior and automatically compute a proper analytic gradient up to machine precision accuracy in only a few multiples of the cost to evaluate the log probability function itself. We explored existing algorithmic differentiation packages with open licenses such as RAD (Gay, 2005) and its repackaging in the Sacado module of the Trilinos toolkit and the CppAD package in the COIN-OR toolkit. But neither package supported very many special functions (e.g., probability functions, log gamma, inverse logit) or linear algebra operations (e.g., Cholesky decomposition) and were not easily and modularly extensible.

So we built our own reverse-mode algorithmic differentiation package. But once we'd built our own reverse-mode algorithmic differentiation package, the problem was that we could not just plug in the probability functions from a package like Boost because they weren't templated on all the arguments. We only needed algorithmic differentiation variables for parameters, not data or transformed data, and promotion is very inefficient in both time and memory. So we wrote our own fully templated probability functions.

Next, we integrated the Eigen C++ package for matrix operations and linear algebra functions. Eigen makes extensive use of expression templates for lazy evaluation and the curiously recurring template pattern to implement concepts without virtual function calls. But we ran into the same problem with Eigen as with the existing probability libraries — it doesn't support mixed operations of algorithmic differentiation variables and primitives like `double`. This is a problem we have yet to optimize away as of Stan version 1.3, but we have plans to extend Eigen itself to support heterogeneous matrix operator types.

At this point (Spring 2011), we were happily fitting models coded directly in C++ on top of the pre-release versions of the Stan API. Seeing how well this all worked, we set our sights on the generality and ease of use of BUGS. So we designed a modeling language in which statisticians could write their models in familiar notation that could be transformed to efficient C++ code and then compiled into an efficient executable program.

The next problem we ran into as we started implementing richer models is variables with constrained support (e.g., simplexes and covariance matrices). Although it is possible to implement HMC with bouncing for simple boundary constraints (e.g., positive scale or precision parameters), it's not so easy with more complex multivariate constraints. To get around this problem, we introduced typed variables and automatically transformed them to unconstrained support with suitable adjustments to the log probability from the log absolute Jacobian determinant of the inverse trans-

forms.

Even with the prototype compiler generating models, we still faced a major hurdle to ease of use. HMC requires two tuning parameters (step size and number of steps) and is very sensitive to how they are set. The step size parameter could be tuned during warmup based on Metropolis rejection rates, but the number of steps was not so easy to tune while maintaining detailed balance in the sampler. This led to the development of the No-U-Turn sampler (NUTS) (Hoffman and Gelman, 2011, 2013), which takes an ever increasing number of steps until the direction of the simulation turns around, then uses slice sampling to select a point on the simulated trajectory.

We thought we were home free at this point. But when we measured the speed of some BUGS examples versus Stan, we were very disappointed. The very first example model, Rats, ran more than an order of magnitude faster in JAGS than in Stan. Rats is a tough test case because the conjugate priors and lack of posterior correlations make it an ideal candidate for efficient Gibbs sampling. But we thought the efficiency of compilation might compensate for the lack of ideal fit to the problem.

We realized we were doing redundant calculations, so we wrote a vectorized form of the normal distribution for multiple variates with the same mean and scale, which sped things up a bit. At the same time, we introduced some simple template metaprograms to remove the calculation of constant terms in the log probability. These both improved speed, but not enough. Finally, we figured out how to both vectorize and partially evaluate the gradients of the densities using a combination of expression templates and metaprogramming. At this point, we are within a factor of two or so of a hand-coded gradient function.

Later, when we were trying to fit a time-series model, we found that normalizing the data to unit sample mean and variance sped up the fits by an order of magnitude. Although HMC and NUTS are rotation invariant (explaining why they can sample effectively from multivariate densities with high correlations), they are not scale invariant. Gibbs sampling, on the other hand, is scale invariant, but not rotation invariant.

We were still using a unit mass matrix in the simulated Hamiltonian dynamics. The last tweak to Stan before version 1.0 was to estimate a diagonal mass matrix during warmup; this has since been upgraded to a full mass matrix in version 1.2. Both these extensions go a bit beyond the NUTS paper on *arXiv*. Using a mass matrix sped up the unscaled data models by an order of magnitude, though it breaks the nice theoretical property of rotation invariance. The full mass matrix estimation has rotational invariance as well, but scales less well because of the need to invert the mass matrix once and then do matrix multiplications every leapfrog step.

Stan 2

It's been over a year since the initial release of Stan, and we have been overjoyed by the quantity and quality of models people are building with Stan. We've also been a bit overwhelmed by the volume of traffic on our user's list and issue tracker.

We've been particularly happy about all the feedback we've gotten about installation issues as well as bugs in the code and documentation. We've been pleasantly surprised at the number of such requests which have come with solutions in the form of a GitHub pull request. That certainly makes our life easy.

As the code base grew and as we became more familiar with it, we came to realize that it required a major refactoring (see, for example, (Fowler et al., 1999) for a nice discussion of refactoring). So while the outside hasn't changed dramatically in Stan 2, the inside is almost totally different in terms of how the HMC samplers are organized, how the output is analyzed, how the mathematics library is organized, etc.

We've also improved our optimization algorithm (BFGS) and its parameterization. We've added more compile-time and run-time error checking for models. We've added many new functions, including new matrix functions and new distributions. We've added some new parameterizations and managed to vectorize all the univariate distributions. We've increased compatibility with a range of C++ compilers.

We've also tried to fill out the manual to clarify things like array and vector indexing, programming style, and the I/O and command-line formats. Most of these changes are direct results of user-reported confusions. So please let us know where we can be clearer or more fully explain something.

Finally, we've fixed all the bugs which we know about. It was keeping up with the latter that really set the development time back, including bugs that resulted in our having to add more error checking.

Stan's Future

We're not done. There's still an enormous amount of work to do to improve Stan. Some older, higher-level goals are in a standalone to-do list:

`https://github.com/stan-dev/stan/wiki/To-Do-List`

We are gradually weaning ourselves off of the to-do list in favor of the GitHub issue tracker (see the next section for a link).

Some major features are on our short-term horizon: Riemannian manifold Hamiltonian Monte Carlo (RHMC), transformed Laplace approximations with uncertainty quantification for maximum likelihood estimation, marginal maximum likelihood estimation, data-parallel expectation propagation, and streaming (stochastic) variational inference.

We will also continue to work on improving numerical stability and efficiency throughout. In addition, we plan to revise the interfaces to make them easier to understand and more flexible to use (a difficult pair of goals to balance).

You Can Help

Please let us know if you have comments about this manual or suggestions for Stan. We're especially interested in hearing about models you've fit or had problems fitting with Stan. The best way to communicate with the Stan team about user issues is through the following user's group.

`http://groups.google.com/group/stan-users`

For reporting bugs or requesting features, Stan's issue tracker is at the following location.

`https://github.com/stan-dev/stan/issues`

One of the main reasons Stan is freedom-respecting, open-source software[2] is that we love to collaborate. We're interested in hearing from you if you'd like to volunteer to get involved on the development side. We have all kinds of projects big and small that we haven't had time to code ourselves. For developer's issues, we have a separate group.

`http://groups.google.com/group/stan-dev`

To contact the project developers off the mailing lists, send email to

`stan@mc-stan.org`

The Stan Development Team
Monday 20th October, 2014

[2]See Appendix A for more information on Stan's licenses and the licenses of the software on which it depends.

Acknowledgements

Institutions

We thank Columbia University along with the Departments of Statistics and Political Science, the Applied Statistics Center, the Institute for Social and Economic Research and Policy (ISERP), and the Core Research Computing Facility.

Grants

Stan was supported in part by the U. S. Department of Energy (DE-SC0002099), the U. S. National Science Foundation ATM-0934516 “Reconstructing Climate from Tree Ring Data.” and the U. S. Department of Education Institute of Education Sciences (ED-GRANTS-032309-005: “Practical Tools for Multilevel Hierarchical Modeling in Education Research” and R305D090006-09A: “Practical solutions for missing data”). The high-performance computing facility on which we ran evaluations was made possible through a grant from the U. S. National Institutes of Health (1G20RR030893-01: “Research Facility Improvement Grant”).

Stan is currently supported in part by a grant from the National Science Foundation (CNS-1205516)

Individuals

We thank John Salvatier for pointing us to automatic differentiation and HMC in the first place. And a special thanks to Kristen van Leuven (formerly of Columbia’s ISERP) for help preparing our initial grant proposals.

Code and Doc Patches

Thanks for bug reports, code patches, pull requests, and diagnostics to: Ethan Adams, Avraham Adler, Jeffrey Arnold, Jarret Barber, David R. Blair, Ross Boylan, Eric N. Brown, Devin Caughey, Ctross (GitHub ID), Jan Gläscher, Robert J. Goedman, Marco Inacio, B. Harris, Kevin Van Horn, Andrew Hunter, Filip Krynicki Dan Lakeland, Devin Leopold, Nathanael I. Lichti, Titus van der Malsburg, P. D. Metcalfe, Linas Mockus, Jeffrey Oldham, Patrick Snape, Matthew Spencer, Alexey Stukalov, Fernando H. Toledo, Zhenming Su, and Matius Simkovic.

Thanks for documentation bug reports and patches to: Jeffrey Arnold, Asim, Jarret Barber, Guido Biele, Luca Billi, Arthur Breitman, Eric C. Brown, David Chudzicki, Andria Dawson, Gökçen Eraslan, Rick Farouni, Avi Feller, Seth Flaxman, Wayne Folta, Kyle Foreman, jonathan-g (GitHub handle), Mauricio Garnier-Villarreal, Herra Huu,

Marco Inacio, Fränzi Korner-Nievergelt, Louis Luangkesorn, Mitzi Morris, Tamas Papp, Tomi Peltola, Sergio Polini, Sean O'Riordain, Cody Ross, Mike Ross, Nathan Sanders, Terrance Savitsky, Dan Schrage, Janne Sinkkonen, Dan Stowell, Alexey Stukalov, Dougal Sutherland, Andrew J. Tanentzap, Shravan Vashisth, Matt Wand, Sebastian Weber, Sam Weiss, and Howard Zail.

Thanks to Kevin van Horn for install instructions for Cygwin and to Kyle Foreman for instructions on using the MKL compiler.

Bug Reports

We're really thankful to everyone who's had the patience to try to get Stan working and reported bugs. All the gory details are available from Stan's issue tracker at the following URL.

`https://github.com/stan-dev/stan/issues`

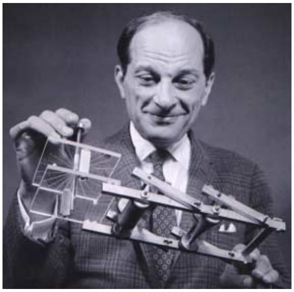

Stanislaw Ulam, namesake of Stan and co-inventor of Monte Carlo methods (Metropolis and Ulam, 1949), shown here holding the Fermiac, Enrico Fermi's physical Monte Carlo simulator for neutron diffusion.

Image from (Giesler, 2000).

Part I

Introduction

1. Overview

This document is both a user's guide and a reference manual for Stan's probabilistic modeling language. This introductory chapter provides a high-level overview of Stan. The remaining parts of this document include a practically-oriented user's guide for programming models and a detailed reference manual for Stan's modeling language and associated programs and data formats.

1.1. Stan Home Page

For links to up-to-date code, examples, manuals, bug reports, feature requests, and everything else Stan related, see the Stan home page:

    http://mc-stan.org/

1.2. Stan Interfaces

There are three interfaces for Stan that are supported as part of the Stan project. Models and their use are the same across the three interfaces, and this manual is the modeling language manual for all three interfaces. All of the interfaces share initialization, sampling and tuning controls, and roughly share posterior analysis functionality.

CmdStan

CmdStan allows Stan to be run from the command line. In some sense, CmdStan is the reference implementation of Stan. The CmdStan documentation used to be part of this document, but is now its own standalone document. The CmdStan home page, with links to download and installation instructions and the manual is code and manual is

    http://mc-stan.org/cmdstan.html

RStan

RStan is the R interface to Stan. RStan interfaces to Stan through R's memory rather than just calling Stan from the outside, as in the R2WinBUGS and R2jags interfaces on which it was modeled. The RStan home page, with links to download and installation instructions and the manual is

    http://mc-stan.org/rstan.html

PyStan

PyStan is the Python interface to Stan. Like RStan, it interfaces at the Python memory level rather than calling Stan from the outside. The PyStan home page, with links to download and installation instructions and the manual is

`http://mc-stan.org/pystan.html`

Future Interfaces

Work is underway to develop interfaces for Stan in:

- MATLAB
- Julia
- Stata

For more information, or to get involved in the design or coding, see the Stan message groups at

`http://mc-stan.org/groups.html`

1.3. Stan Programs

A Stan program defines a statistical model through a conditional probability function $p(\theta|y, x)$, where θ is a sequence of modeled unknown values (e.g., model parameters, latent variables, missing data, future predictions), y is a sequence of modeled known values, and x is a sequence of unmodeled predictors and constants (e.g., sizes, hyperparameters).

Stan programs consist of variable type declarations and statements. Variable types include constrained and unconstrained integer, scalar, vector, and matrix types, as well as (multidimensional) arrays of other types. Variables are declared in blocks corresponding to the variable's use: data, transformed data, parameter, transformed parameter, or generated quantity. Unconstrained local variables may be declared within statement blocks.

Statements in Stan are interpreted imperatively, so their order matters. Atomic statements involve the assignment of a value to a variable. Sequences of statements (and optionally local variable declarations) may be organized into a block. Stan also provides bounded for-each loops of the sort used in R and BUGS.

The transformed data, transformed parameter, and generated quantities blocks contain statements defining the variables declared in their blocks. A special model block consists of statements defining the log probability for the model.

Within the model block, BUGS-style sampling notation may be used as shorthand for incrementing an underlying log probability variable, the value of which defines the log probability function. The log probability variable may also be accessed directly, allowing user-defined probability functions and Jacobians of transforms.

1.4. Compiling and Running Stan Programs

A Stan program is first compiled to a C++ program by the Stan compiler `stanc`, then the C++ program compiled to a self-contained platform-specific executable. Stan can generate executables for various flavors of Windows, Mac OS X, and Linux.[1] Running the Stan executable for a model first reads in and validates the known values y and x, then generates a sequence of (non-independent) identically distributed samples $\theta^{(1)}, \theta^{(2)}, \ldots$, each of which has the marginal distribution $p(\theta|y, x)$.

1.5. Sampling

For continuous parameters, Stan uses Hamiltonian Monte Carlo (HMC) sampling (Duane et al., 1987; Neal, 1994, 2011), a form of Markov chain Monte Carlo (MCMC) sampling (Metropolis et al., 1953). Stan 1.0 does not do discrete sampling. Chapter 9 and Chapter 11 discuss how finite discrete parameters can be summed out of models.

HMC accelerates both convergence to the stationary distribution and subsequent parameter exploration by using the gradient of the log probability function. The unknown quantity vector θ is interpreted as the position of a fictional particle. Each iteration generates a random momentum and simulates the path of the particle with potential energy determined the (negative) log probability function. Hamilton's decomposition shows that the gradient of this potential determines change in momentum and the momentum determines the change in position. These continuous changes over time are approximated using the leapfrog algorithm, which breaks the time into discrete steps which are easily simulated. A Metropolis reject step is then applied to correct for any simulation error and ensure detailed balance of the resulting Markov chain transitions (Metropolis et al., 1953; Hastings, 1970).

Standard HMC involves three "tuning" parameters to which its behavior is quite sensitive. Stan's samplers allow these parameters to be set by hand or set automatically without user intervention.

The first two tuning parameters set the temporal step size of the discretization of the Hamiltonian and the total number of steps taken per iteration (with their product determining total simulation time). Stan can be configured with a user-specified

[1] A Stan program may also be compiled to a dynamically linkable object file for use in a higher-level scripting language such as R or Python.

step size or it can estimate an optimal step size during warmup using dual averaging (Nesterov, 2009; Hoffman and Gelman, 2011, 2013). In either case, additional randomization may be applied to draw the step size from an interval of possible step sizes (Neal, 2011).

Stan can be set to use a specified number of steps, or it can automatically adapt the number of steps during sampling using the No-U-Turn (NUTS) sampler (Hoffman and Gelman, 2011, 2013).

The third tuning parameter is a mass matrix for the fictional particle. Stan can be configured to estimate a diagonal mass matrix or a full mass matrix during warmup; Stan will support user-specified mass matrices in the future. Estimating a diagonal mass matrix normalizes the scale of each element θ_k of the unknown variable sequence θ, whereas estimating a full mass matrix accounts for both scaling and rotation,[2] but is more memory and computation intensive per leapfrog step due to the underlying matrix operations.

Convergence Monitoring and Effective Sample Size

Samples in a Markov chain are only drawn with the marginal distribution $p(\theta|y,x)$ after the chain has converged to its equilibrium distribution. There are several methods to test whether an MCMC method has failed to converge; unfortunately, passing the tests does not guarantee convergence. The recommended method for Stan is to run multiple Markov chains each with different diffuse initial parameter values, discard the warmup/adaptation samples, then split the remainder of each chain in half and compute the potential scale reduction statistic, $\hat{R}$ (Gelman and Rubin, 1992).

When estimating a mean based on M independent samples, the estimation error is proportional to $1/\sqrt{M}$. If the samples are positively correlated, as they typically are when drawn using MCMC methods, the error is proportional to $1/\sqrt{\textsc{ess}}$, where ESS is the effective sample size. Thus it is standard practice to also monitor (an estimate of) the effective sample size of parameters of interest in order to estimate the additional estimation error due to correlated samples.

Bayesian Inference and Monte Carlo Methods

Stan was developed to support full Bayesian inference. Bayesian inference is based in part on Bayes's rule,

$$p(\theta|y,x) \propto p(y|\theta,x)\, p(\theta,x),$$

[2] These estimated mass matrices are global, meaning they are applied to every point in the parameter space being sampled. Riemann-manifold HMC generalizes this to allow the curvature implied by the mass matrix to vary by position.

which, in this unnormalized form, states that the posterior probability $p(\theta|y, x)$ of parameters θ given data y (and constants x) is proportional (for fixed y and x) to the product of the likelihood function $p(y|\theta, x)$ and prior $p(\theta, x)$.

For Stan, Bayesian modeling involves coding the posterior probability function up to a proportion, which Bayes's rule shows is equivalent to modeling the product of the likelihood function and prior up to a proportion.

Full Bayesian inference involves propagating the uncertainty in the value of parameters θ modeled by the posterior $p(\theta|y, x)$. This can be accomplished by basing inference on a sequence of samples from the posterior using plug-in estimates for quantities of interest such as posterior means, posterior intervals, predictions based on the posterior such as event outcomes or the values of as yet unobserved data.

1.6. Optimization

Stan also supports optimization-based inference for models. Given a posterior $p(\theta|y)$, Stan can find the posterior mode θ^*, which is defined by

$$\theta^* = \text{argmax}_\theta \; p(\theta|y).$$

Here the notation $\text{argmax}_u \; f(v)$ is used to pick out the value of v at which $f(v)$ is maximized.

If the prior is uniform, the posterior mode corresponds to the maximum likelihood estimate (MLE) of the parameters. If the prior is not uniform, the posterior mode is sometimes called the maximum a posterior (MAP) estimate. If parameters (typically hierarchical) have been marginalized out, it's sometimes called a maximum marginal likelihood (MML) estimate.

For optimization, the Jacobian of any transforms induced by constraints on variables are ignored. It is more efficient in many optimization problems to remove lower and upper bound constraints in variable the declarations and instead rely on rejection in the model block to disallow out-of-support solutions.

Inference with Point Estimates

The estimate θ^* is a so-called "point estimate," meaning that it summarizes the posterior distribution by a single point, rather than with a distribution. Of course, a point estimate does not, in and of itself, take into account estimation variance. Posterior predictive inferences $p(\tilde{y}|y)$ can be made using the posterior mode given data y as $p(\tilde{y}|\theta^*)$, but they are not Bayesian inferences, even if the model involves a prior, because they do not take posterior uncertainty into account. If the posterior variance is low and the posterior mean is near the posterior mode, inference with point estimates can be very similar to full Bayesian inference.

Part II

Programming Techniques

2. Model Building as Software Development

Developing a Stan model is a software development process. Developing software is hard. Very hard. So many things can go wrong because there are so many moving parts and combinations of parts.

Software development practices are designed to mitigate the problems caused by the inherent complexity of software development. Unfortunately, many methodologies veer off into dogma, bean counting, or both. A couple we can recommend that provide solid, practical advice for developers are (Hunt and Thomas, 1999) and (McConnell, 2004). This section tries to summarize some of their advice.

2.1. Use Version Control

Version control software, such as Subversion or Git, should be in place before starting to code.[1] It may seem like a big investment to learn version control, but it's well worth it to be able to type a single command to revert to a previously working version or to get the difference between the current version and an old version. It's even better when you need to share work with others, even on a paper.

2.2. Make it Reproducible

Rather than entering commands on the command-line when running models (or entering commands directly into an interactive programming language like R or Python), try writing scripts to run the data through the models and produce whatever posterior analysis you need. Scripts can be written for the shell, R, or Python. Whatever language a script is in, it should be self contained and not depend on global variables having been set, other data being read in, etc.

See Chapter 20 for complete information on reproducibility in Stan and its interfaces.

Scripts are Good Documentation

It may seem like overkill if running the project is only a single line of code, but the script provides not only a way to run the code, but also a form of concrete documentation for what is run.

[1] Stan started using Subversion (SVN), then switched to the much more feature-rich Git package. Git does everything SVN does and a whole lot more. The price is a steeper learning curve. For individual or very-small-team development, SVN is just fine.

Randomization and Saving Seeds

Randomness defeats reproducibility. MCMC methods are conceptually randomized. Stan's samplers involve random initializations as well as randomization during each iteration (e.g., Hamiltonian Monte Carlo generates a random momentum in each iteration).

Computers are deterministic. There is no real randomness, just pseudo-random number generators. These operate by generating a sequence of random numbers based on a "seed." Stan (and other languages like R) can use time-based methods to generate a seed based on the time and date, or seeds can be provided to Stan (or R) in the form of long integers. Stan writes out the seed used to generate the data as well as the version number of the Stan software so that results can be reproduced at a later date.[2]

2.3. Make it Readable

Treating programs and scripts like other forms of writing for an audience provides an important perspective on how the code will be used. Not only might others want to read a program or model, the developer will want to read it later. One of the motivations of Stan's design was to make models self-documenting in terms of variable usage (e.g., data versus parameter), types (e.g., covariance matrix vs. unconstrained matrix) and sizes.

A large part of readability is consistency. Particularly in naming and layout. Not only of programs themselves, but the directories and files in which they're stored.

Readability of code is not just about comments (see Section Section 2.8 for commenting recommendations and syntax in Stan).

It is surprising how often the solution to a debugging or design problem occurs when trying to explain enough about the problem to someone else to get help. This can be on a mailing list, but it works best person-to-person. Finding the solution to your own problem when explaining it to someone else happens so frequently in software development that the listener is called a "rubber ducky," because they only have to nod along.[3]

[2]This also requires fixing compilers and hardware, because floating-point arithmetic does not have an absolutely fixed behavior across platforms or compilers, just operating parameters.

[3]Research has shown an actual rubber ducky won't work. For some reason, the rubber ducky must actually be capable of understanding the explanation.

2.4. Explore the Data

Although this should go without saying, don't just fit data blindly. Look at the data you actually have to understand its properties. If you're doing a logistic regression, is it separable? If you're building a multilevel model, do the basic outcomes vary by level? If you're fitting a linear regression, see whether such a model makes sense by scatterplotting x vs. y.

2.5. Design Top-Down, Code Bottom-Up

Software projects are almost always designed top-down from one or more intended use cases. Good software coding, on the other hand, is typically done bottom-up.

The motivation for top-down design is obvious. The motivation for bottom-up development is that it is much easier to develop software using components that have been thoroughly tested. Although Stan has no built-in support for either modularity or testing, many of the same principles apply.

The way the developers of Stan themselves build models is to start as simply as possibly, then build up. This is true even if we have a complicated model in mind as the end goal, and even if we have a very good idea of the model we eventually want to fit. Rather than building a hierarchical model with multiple interactions, covariance priors, or other complicated structure, start simple. Build just a simple regression with fixed (and fairly tight) priors. Then add interactions or additional levels. One at a time. Make sure that these do the right thing. Then expand.

2.6. Fit Simulated Data

One of the best ways to make sure your model is doing the right thing computationally is to generate simulated (i.e., "fake") data with known parameter values, then see if the model can recover these parameters from the data. If not, there is very little hope that it will do the right thing with data from the wild.

There are fancier ways to do this, where you can do things like run χ^2 tests on marginal statistics or follow the paradigm introduced in (Cook et al., 2006), which involves interval tests.

2.7. Debug by Print

Although Stan does not have a stepwise debugger or any unit testing framework in place, it does support the time-honored tradition of debug-by-printf. [4]

Stan supports print statements with one or more string or expression arguments. Because Stan is an imperative language, variables can have different values at different points in the execution of a program. Print statements can be invaluable for debugging, especially for a language like Stan with no stepwise debugger.

For instance, to print the value of variables `y` and `z`, use the following statement.

```
print("y=", y, " z=", z);
```

This print statement prints the string "y=" followed by the value of `y`, followed by the string " z=" (with the leading space), followed by the value of the variable `z`.

Each print statement is followed by a new line. The specific ASCII character(s) generated to create a new line are platform specific.

Arbitrary expressions can be used. For example, the statement

```
print("1+1=", 1+1);
```

will print "1 + 1 = 2" followed by a new line.

Print statements may be used anywhere other statements may be used, but their behavior in terms of frequency depends on how often the block they are in is evaluated. See Section 24.8 for more information on the syntax and evaluation of print statements.

2.8. Comments

Code Never Lies

The machine does what the code says, not what the documentation says. Documentation, on the other hand, might not match the code. Code documentation easily rots as the code evolves if the documentation is not well maintained.

Thus it is always preferable to write readable code as opposed to documenting unreadable code. Every time you write a piece of documentation, ask yourself if there's a way to write the code in such a way as to make the documentation unnecessary.

[4] The "f" is not a typo — it's a historical artifact of the name of the `printf` function used for formatted printing in C.

Comment Styles in Stan

Stan supports C++-style comments; see Section 26.1 for full details. The recommended style is to use line-based comments for short comments on the code or to comment out one or more lines of code. Bracketed comments are then reserved for long documentation comments. The reason for this convention is that bracketed comments cannot be wrapped inside of bracketed comments.

What Not to Comment

When commenting code, it is usually safe to assume that you are writing the comments for other programmers who understand the basics of the programming language in use. In other words, don't comment the obvious. For instance, there is no need to have comments such as the following, which add nothing to the code.

```
y ~ normal(0,1);  // y has a unit normal distribution
```

A Jacobian adjustment for a hand-coded transform might be worth commenting, as in the following example.

```
exp(y) ~ normal(0,1);
// adjust for change of vars: y = log | d/dy exp(y) |
increment_log_prob(y);
```

It's an art form to empathize with a future code reader and decide what they will or won't know (or remember) about statistics and Stan.

What to Comment

It can help to document variable declarations if variables are given generic names like N, mu, and sigma. For example, some data variable declarations in an item-response model might be usefully commented as follows.

```
int<lower=1> N;   // number of observations
int<lower=1> I;   // number of students
int<lower=1> J;   // number of test questions
```

The alternative is to use longer names that do not require comments.

```
int<lower=1> n_obs;
int<lower=1> n_students;
int<lower=1> n_questions;
```

Both styles are reasonable and which one to adopt is mostly a matter of taste (mostly because sometimes models come with their own naming conventions which should be followed so as not to confuse readers of the code familiar with the statistical conventions).

Some code authors like big blocks of comments at the top explaining the purpose of the model, who wrote it, copyright and licensing information, and so on. The following bracketed comment is an example of a conventional style for large comment blocks.

```
/*
 * Item-Response Theory PL3 Model
 * -----------------------------------------------------
 * Copyright: Joe Schmoe  <joe@schmoe.com>
 * Date:  19 September 2012
 * License: GPLv3
 */

data {
  ...
```

The use of leading asterisks helps readers understand the scope of the comment. The problem with including dates or other volatile information in comments is that they can easily get out of synch with the reality of the code. A misleading comment or one that is wrong is worse than no comment at all!

3. Data Types

This chapter discusses the data types available for variable declarations and expression values in Stan. Variable types are important for declaring parameters, checking data consistency, calling functions, and assigning values to variables.

In Stan, every expression and variable declaration has an associated type that is determined statically (i.e., when the program is compiled). Sizes of vectors, matrices, and arrays, on the other hand, are determined dynamically (i.e., when the program is run). This is very different than a language like R, which lets you assign a string to a variable and then later assign a matrix to it.

Expressions may be primitive, such as variables or constants, or they may be composed of other components, such as a function or operator applied to arguments.

This chapter concentrates on the basic data types and how they are declared, assigned, and used. The following chapter provides a detailed comparison of the differences among the container types: arrays, vectors, and matrices.

3.1. Basic Data Types

Arguments for built-in and user-defined functions and local variables are required to be basic data types, meaning an unconstrained primitive, vector, or matrix type or an array of such.

Primitive Types

Stan provides two primitive data types, `real` for continuous values and `int` for integer values.

Vector and Matrix Types

Stan provides three matrix-based data types, `vector` for column vectors, `row_vector` for row vectors, and `matrix` for matrices.

Array Types

Any type (including the constrained types discussed in the next section) can be made into an array type by declaring array arguments. For example,

```
real x[10];
matrix[3,3] m[6,7];
```

declares `x` to be a one-dimensional array of size 10 containing real values, and declares `m` to be a two-dimensional array of size 6×7 containing values that are 3×3 matrices.

3.2. Constrained Data Types

Declarations of variables other than local variables may be provided with constraints. Each constrained data type corresponds to a basic data type with constraints.

Constraints provide error checking for variables defined in the `data`, `transformed data`, `transformed parameters`, and `generated quantities` blocks.

Constraints are critical for variables declared in the `parameters` block, where they determine the transformation from constrained variables (those satisfying the declared constraint) to unconstrained variables (those ranging over all of $\mathbb{R}^n$).

It is worth calling out the most important aspect of constrained data types:

> *The model must have support (non-zero density) at every value of the parameters that meets their declared constraints.*

If the declared parameter constraints are less strict than the support, the samplers and optimizers may be have any of a number of pathologies including just getting stuck, failure to initialize, excessive Metropolis rejection, or biased samples due to inability to explore the tails of the distribution.

Upper and Lower Bounds

Variables may be declared with constraints All of the basic data types may be given lower and upper bounds using syntax such as

```
int<lower=1> N;
real<upper=0> log_p;
vector<lower=-1,upper=1>[3,3] corr;
```

Structured Vectors

There are also special data types for structured vectors. These are `ordered` for a vector of values ordered in increasing order, and `positive_ordered` for a vector of positive values ordered in increasing order.

There is also a type `simplex` for vectors of non-negative values that sum to one, and `unit_vector` for vectors of values whose squares sum to one.

Structured Matrices

Symmetric, positive-definite matrices have the type cov_matrix. Correlation matrices, which are symmetric positive-definite matrices with unit diagonals, have the type corr_matrix.

There is also a pair of Cholesky factor types. The first, cholesky_factor_cov, is for Cholesky factors of symmetric, positive definite matrices, which amounts to being a lower-triangular matrix with positive diagonal elements. The second, and cholesky_factor_corr, is for Cholesky factors of correlation matrices, which amounts to being a lower-triangular matrix with positive diagonal elements, where additionally the length of each row is 1. Using Cholesky factor types can be much more efficient than the full correlation or covariance matrices because they are easier to factor and scale.

3.3. Assignment and Argument Passing

Assignment

Constrained data values may be assigned to unconstrained variables of matching basic type and vice-versa. Matching is interpreted strictly as having the same basic type and number of array dimensions. Constraints are not considered, but basic data types are.

Arrays cannot be assigned to vectors and vice-versa. Similarly, vectors cannot be assigned to matrices and vice-versa, even if their dimensions conform. Chapter 4 provides more information on the distinctions between vectors and arrays and when each is appropriate.

Function Invocation

Passing arguments to functions in Stan works just like assignment to basic types. Stan functions are only specified for the basic data types of their arguments, including array dimensionality, but not for sizes or constraints. Of course, functions often check constraints as part of their behavior.

4. Containers: Arrays, Vectors, and Matrices

Stan provides three types of container objects: arrays, vectors, and matrices. The three types are not interchangeable. Vectors, matrices, and arrays are not assignable to one another, even if their dimensions are identical. A 3×4 matrix is a different kind of object in Stan than a 3×4 array.

4.1. Vectors and Matrices

Vectors and matrices are more limited kinds of data structures than arrays. Vectors are intrinsically one-dimensional collections of reals, whereas matrices are intrinsically two dimensional.

The intention of using matrix types is to call out their usage in the code. There are three situations in Stan where *only* vectors and matrices may be used,

- matrix arithmetic operations (e.g., matrix multiplication)
- linear algebra functions (e.g., eigenvalues and determinants), and
- multivariate function parameters and outcomes (e.g., multivariate normal distribution arguments).

Vectors and matrices cannot be typed to return integer values. They are restricted to `real` values.[1]

4.2. Arrays

Arrays, on the other hand, are intrinsically one-dimensional collections of other kinds of objects. The values in an array can be any type, so that arrays may contain values that are simple reals or integers, vectors, matrices, or other arrays. Arrays are the only way to store sequences of integers, and some functions in Stan, such as discrete distributions, require integer arguments.

A two-dimensional array is just an array of arrays, both conceptually and in terms of current implementation. When an index is supplied to an array, it returns the value at that index. When more than one index is supplied, this idexing operation is chained. For example, if `a` is a two-dimensional array, then `a[m,n]` is just a convenient shorthand for `a[m][n]`.

[1]This may change if Stan is called upon to do complicated integer matrix operations or boolean matrix operations. Integers are not appropriate inputs for linear algebra functions.

4.3. Efficiency Considerations

One of the motivations for Stan's underlying design is efficiency.

The underlying matrix and linear algebra operations are implemented in terms of data types from the Eigen C++ library. By having vectors and matrices as basic types, no conversion is necessary when invoking matrix operations or calling linear algebra functions.

Arrays, on the other hand, are implemented as instances of the C++ `std::vector` class (not to be confused with Eigen's `Eigen::Vector` class or Stan vectors). By implementing arrays this way, indexing is very efficient because values can be returned by reference rather than copied by value.

Matrices vs. Two-Dimensional Arrays

In Stan models, there are a few minor efficiency considerations in deciding between a two-dimensional array and a matrix, which may seem interchangeable at first glance.

First, matrices use a bit less memory than two-dimensional arrays. This is because they don't store a sequence of arrays, but just the data and the two dimensions.

Second, matrices store their data in column-major order. Furthermore, all of the data in a matrix is guaranteed to be contiguous in memory. This is an important consideration for optimized code because bringing in data from memory to cache is much more expensive than performing arithmetic operations with contemporary CPUs. Arrays, on the other hand, only guarantee that the values of primitive types are contiguous in memory; otherwise, they hold copies of their values (which are returned by reference wherever possible).

Third, both data structures are best traversed in the order in which they are stored. This also helps with memory locality. This is column-major for matrices, so the following order is appropriate.

```
matrix[M,N] a;
...
for (n in 1:N)
  for (m in 1:M)
    ... do something with a[m,n] ...
```

Arrays, on the other hand, should be traversed in row-major (or first-index fastest) order.

```
real a[M,N];
...
for (m in 1:M)
```

```
    for (n in 1:N)
      ... do something with a[m,n] ...
```

The first use of a[m,n] should bring a[m] into memory. Overall, traversing matrices is more efficient than traversing arrays.

This is true even for arrays of matrices. For example, the ideal order in which to traverse a two-dimensional array of matrices is

```
matrix[M,N] b[I,J];
...
for (i in 1:I)
  for (j in 1:J)
    for (n in 1:N)
      for (m in 1:M)
        ... do something with b[i,j,m,n] ...
```

If a is a matrix, the notation a[m] picks out row m of that matrix. This is a rather inefficient operation for matrices. If indexing of vectors is needed, it is much better to declare an array of vectors. That is, this

```
row_vector[N] b[M];
...
for (m in 1:M)
   ... do something with row vector b[m] ...
```

is much more efficient than the pure matrix version

```
matrix b[M,N];
...
for (m in 1:M)
   ... do something with row vector b[m] ...
```

Similarly, indexing an array of column vectors is more efficient than using the col function to pick out a column of a matrix.

In contrast, whatever can be done as pure matrix algebra will be the fastest. So if I want to create a row of predictor-coefficient dot-products, it's more efficient to do this

```
matrix[N,K] x;    // predictors (aka covariates)
...
vector[K] beta;   // coeffs
...
vector[N] y_hat;  // linear prediction
```

```
...
y_hat <- x * beta;
```

than it is to do this

```
row_vector[K] x[N];    // predictors (aka covariates)
...
vector[K] beta;   // coeffs
...
vector[N] y_hat;  // linear prediction
...
for (n in 1:N)
  y_hat[n] <- x[n] * beta;
```

(Row) Vectors vs. One-Dimensional Arrays

For use purely as a container, there is really nothing to decide among vectors, row vectors and one-dimensional arrays. The `Eigen::Vector` template specialization and the `std::vector` template class are implemented very similarly as containers of `double` values (the type `real` in Stan). Only arrays in Stan are allowed to store integer values.

5. Regression Models

Stan supports regression models from simple linear regressions to multilevel generalized linear models.

5.1. Linear Regression

The simplest linear regression model is the following, with a single predictor and a slope and intercept coefficient, and normally distributed noise. This model can be written using standard regression notation as

$$y_n = \alpha + \beta x_n + \epsilon_n \quad \text{where} \quad \epsilon_n \sim \mathsf{Normal}(0, \sigma).$$

This is equivalent to the following sampling involving the residual,

$$y_n - (\alpha + \beta X_n) \sim \mathsf{Normal}(0, \sigma),$$

and reducing still further, to

$$y_n \sim \mathsf{Normal}(\alpha + \beta X_n,\, \sigma).$$

This latter form of the model is coded in Stan as follows.

```
data {
  int<lower=0> N;
  vector[N] x;
  vector[N] y;
}
parameters {
  real alpha;
  real beta;
  real<lower=0> sigma;
}
model {
  for (n in 1:N)
    y[n] ~ normal(alpha + beta * x[n], sigma);
}
```

There are `N` observations, each with predictor `x[n]` and outcome `y[n]`. The intercept and slope parameters are `alpha` and `beta`. The model assumes a normally distributed noise term with scale `sigma`. This model has improper priors for the two regression coefficients.

Matrix Notation and Vectorization

The sampling statement in the previous model can be vectorized and written equivalently as follows:

```
model {
  y ~ normal(alpha + beta * x, sigma);
}
```

The main difference is that the vectorized form is much faster.[1]

In general, Stan allows the arguments to distributions such as `normal` to be vectors. If any of the other arguments are vectors or arrays, they have to be the same size. If any of the other arguments is a scalar, it is reused for each vector entry. See Chapter 28 for more information on vectorization.

The other reason this works is that Stan's arithmetic operators are overloaded to perform matrix arithmetic on matrices. In this case, because `x` is of type `vector` and `beta` of type `real`, the expression `beta * x` is of type `vector`. Because Stan supports vectorization, a regression model with more than one predictor can be written directly using matrix notation.

```
data {
  int<lower=0> N;   // number of data items
  int<lower=0> K;   // number of predictors
  matrix[N,K] x;    // predictor matrix
  vector[N] y;      // outcome vector
}
parameters {
  real alpha;           // intercept
  vector[K] beta;       // coefficients for predictors
  real<lower=0> sigma;  // error scale
}
model {
  y ~ normal(x * beta, sigma);  // likelihood
}
```

The constraint `lower=0` in the declaration of `sigma` constrains the value to be greater than or equal to 0. With no prior in the model block, the effect is an improper prior

[1]Unlike in Python and R, which are interpreted, Stan is translated to C++ and compiled, so loops and assignment statements are fast. Vectorized code is faster in Stan because (a) the expression tree used to compute derivatives can be simplified, leading to fewer virtual function calls, and (b) computations that would be repeated in the looping version, such as `log(sigma)` in the above model, will be computed once and reused.

on non-negative real numbers. Althogh a more informative prior may be added, improper priors are acceptable as long as they lead to proper posteriors.

In the model above, `x` is an $N \times K$ matrix of predictors and `beta` a K-vector of coefficients, so `x * beta` is an N-vector of predictions, one for each of the N data items. These predictions line up with the outcomes in the N-vector `y`, so the entire model may be written using matrix arithmetic as shown.

The sampling statement in the model above is just a more efficient, vector-based approach to coding the model with a loop, as in the following statistically equivalent model.

```
model {
  for (n in 1:N)
    y[n] ~ normal(x[n] * beta, sigma);
}
```

With Stan's matrix indexing scheme, `x[n]` picks out row `n` of the matrix `x`; because `beta` is a column vector, the product `x[n] * beta` is a scalar of type `real`.

Intercepts as Inputs

In the model formulation

```
y ~ normal(x * beta, sigma);
```

there is no longer an intercept coefficient `alpha`. Instead, we have assumed that the first column of the input matrix `x` is a column of 1 values. This way, `beta[1]` plays the role of the intercept. If the intercept gets a different prior than the the slope terms, then it would be clearer to break it out. It is also slightly more efficient in its explicit form with the intercept variable singled out because there's one fewer multiplications; it should not make that much of a difference to speed, though, so the choice should be based on clarity.

5.2. Priors for Coefficients and Scales

This section describes the choices available for modeling priors for regression coefficients and scales. Priors for univariate parameters in hierarchical models are discussed in Section 5.8 and multivariate parameters in Section 5.11. There is also a discussion of priors used to identify models in Section 5.10.

Background Reading

See (Gelman, 2006) for more an overview of choices for priors for scale parameters, (Chung et al., 2013) for an overview of choices for scale priors in penalized maximum likelihood estimates, and Gelman et al. (2008) for a discussion of prior choice for regression coefficients.

Improper Uniform Priors

The default in Stan is to provide uniform (or "flat") priors on parameters over their legal values as determined by their declared constraints. A parameter declared without constraints is thus given a uniform prior on $(-\infty, \infty)$ by default, whereas a scale parameter declared with a lower bound of zero gets an improper uniform prior on $(0, \infty)$. Both of these priors are improper in the sense that there is no way formulate a density function for them that integrates to 1 over its support.

Stan allows models to be formulated with improper priors, but in order for sampling or optimization to work, the data provided must ensure a proper posterior. This usually requires a minimum quantity of data, but can be useful as a starting point for inference and as a baseline for sensitivity analysis (i.e., considering the effect the prior has on the posterior).

Uniform Priors and Transforms

Uniform priors are specific to the scale on which they are formulated. For instance, we could give a scale parameter $\sigma > 0$ a uniform prior on $(0, \infty)$, $q(\sigma) = c$ (we use q because the "density" is not only unnormalized, but unnormalizable), or we could work on the log scale and provide $\log \sigma$ a uniform prior on $(-\infty, \infty)$, $q(\log \sigma) = c$. These work out to be different priors on σ due to the Jacobian adjustment necessary for the log transform; see Section 53.1 for more information on changes of variables and their requisite Jacobian adjustments.

Stan automatically applies the necessary Jacobian adjustment for variables declared with constraints to ensure a uniform density on the legal constrained values. This Jacobian adjustment is turned off when optimization is being applied in order to produce appropriate maximum likelihood estimates.

"Uninformative" Proper Priors

It is not uncommon to see models with priors on regression coefficients such as $\mathsf{Normal}(0, 1000)$.[2] If the prior scale, such as 1000, is several orders of magnitude

[2] The practice was common in BUGS and can be seen in most of their examples Lunn et al. (2012).

larger than the estimated coefficients, then such a prior is effectively providing no effect whatsoever.

We actively discourage users from using the default scale priors suggested through the BUGS examples (Lunn et al., 2012), such as

$$\sigma^2 \sim \text{InvGamma}(0.001, 0.001).$$

Such priors concentrate too much probability mass outside of reasonable posterior values, and unlike the symmetric wide normal priors, can have the profound effect of skewing posteriors; see (Gelman, 2006) for examples and discussion.

Truncated Priors

If a variable is declared with a lower bound of zero, then assigning it a normal prior in a Stan model produces the same effect as providing a properly truncated half-normal prior. The truncation at zero need not be specified as Stan only requires the density up to a proportion. So a variable declared with

```
real<lower=0> sigma;
```

and given a prior

```
sigma ~ normal(0,1000);
```

gives `sigma` a half-normal prior, technically

$$p(\sigma) = \frac{\text{Normal}(\sigma|0, 1000)}{1 - \text{NormalCDF}(0|0, 1000)} \propto \text{Normal}(\sigma|0, 1000),$$

but Stan is able to avoid the calculation of the normal cumulative distribution (CDF) function required to normalize the half-normal density. If either the prior location or scale is a parameter or if the truncation point is a parameter, the truncation cannot be dropped, because the normal CDF term will not be a constant.

Weakly Informative Priors

Typically a researcher will have some knowledge of the scale of the variables being estimated. For instance, if we're estimating an intercept-only model for the mean population height for adult women, then we know the answer is going to be somewhere in the one to three meter range. That gives us information around which to form a weakly informative prior.

Similarly, logistic regression with predictors on the standard scale (roughly zero mean, unit variance), then it is unlikely to have a coefficient that's larger than five in

absolute value. In these cases, it makes sense to provide a weakly informative prior such as $\mathsf{Normal}(0, 5)$ for such a coefficient.

Weakly informative priors help control inference computationally and statistically. Computationally, a prior increases the curvature around the volume where the solution is expected to lie, which in turn guides both gradient-based like L-BFGS and Hamiltonian Monte Carlo sampling by not allowing them to stray too far from the location of a surface. Statistically, a weakly informative prior is more sensible for a problem like women's mean height, because a very diffuse prior like $\mathsf{Normal}(0, 1000)$ will ensure that the vast majority of the prior probability mass is outside the range of the expected answer, which can overwhelm the inferences available from a small data set.

Bounded Priors

Consider the women's height example again. One way to formulate a proper prior is to impose a uniform prior on a bounded scale. For example, we could declare the parameter for mean women's height to have a lower bound of one meter and an upper bound of three meters. Surely the answer has to lie in that range.

Similarly, it is not uncommon to see priors for scale parameters that impose lower bounds of zero and upper bounds of very large numbers, such as 10,000.[3] This provides roughly the same problem for estimation as a very diffuse inverse gamma prior on variance. We prefer to leave parameters which are not absolutely physically constrained to float and provide them informative priors. In the case of women's height, such a prior might be $\mathsf{Normal}(2, 0.5)$ on the scale of meters; it concentrates 95% of its mass in the interval $(1, 3)$, but still allows values outside of that region.

In cases where bounded priors are used, the posterior fits should be checked to make sure the parameter is not estimated at or very close to a boundary. This will not only cause computational problems, it indicates a problem with the way the model is formulated. In such cases, the interval should be widened to see where the parameter fits without such constraints, or boundary-avoid priors should be used (see Section 5.8.)

Fat-Tailed Priors and "Default" Priors

A reasonable alternative if we want to accomodate outliers is to use a prior that concentrates most of mass around the area where values are expected to be, but still leaves a lot of mass in its tails. The usual choice in such a situation is to use a Cauchy

[3]This was also a popular strategy in the BUGS example models (Lunn et al., 2012), which often went one step further and set the lower bounds to a small number like 0.001 to discourage numerical underflow to zero.

distribution for a prior, which can concentrate its mass around its median, but has tails that are so fat that the variance is infinite.

Without specific information, the Cauchy prior is a very good default parameter choice for regression coefficients (Gelman et al., 2008) and the half-Cauchy (coded implicitly in Stan) a good default choice for scale parameters (Gelman, 2006).

Informative Priors

Ideally, there will be substantive information about a problem that can be included in an even tighter prior than a weakly informative prior. This may come from actual prior experiments and thus be the posterior of other data, it may come from meta-analysis, or it may come simply by soliciting it from domain experts. All the goodness of weakly informative priors applies, only with more strength.

Conjugacy

Unlike in Gibbs sampling, there is no computational advantage to providing conjugate priors (i.e., priors that produce posteriors in the same family) in a Stan program.[4] Neither the Hamiltonian Monte Carlo samplers or the optimizers make use of conjugacy, working only on the log density and its derivatives.

5.3. Robust Noise Models

The standard approach to linear regression is to model the noise term ϵ as having a normal distribution. From Stan's perspective, there is nothing special about normally distributed noise. For instance, robust regression can be accommodated by giving the noise term a Student-t distribution. To code this in Stan, the sampling distribution is changed to the following.

```
data {
  ...
  real<lower=0> nu;
}
...
model {
  for (n in 1:N)
    y[n] ~ student_t(nu, alpha + beta * x[n], sigma);
}
```

[4] BUGS and JAGS both support conjugate sampling through Gibbs sampling. JAGS extended the range of conjugacy that could be exploited with its GLM module. Unlike Stan, both BUGS and JAGS are restricted to conjugate priors for constrained multivariate quantities such as covariance matrices or simplexes.

The degrees of freedom constant nu is specified as data.

5.4. Logistic and Probit Regression

For binary outcomes, either of the closely related logistic or probit regression models may be used. These generalized linear models vary only in the link function they use to map linear predictions in $(-\infty, \infty)$ to probability values in $(0, 1)$. Their respective link functions, the logistic function and the unit normal cumulative distribution function, are both sigmoid functions (i.e., they are both *S*-shaped).

A logistic regression model with one predictor and an intercept is coded as follows.

```
data {
  int<lower=0> N;
  vector[N] x;
  int<lower=0,upper=1> y[N];
}
parameters {
  real alpha;
  real beta;
}
model {
  y ~ bernoulli_logit(alpha + beta * x);
}
```

The noise parameter is built into the Bernoulli formulation here rather than specified directly.

Logistic regression is a kind of generalized linear model with binary outcomes and the log odds (logit) link function, defined by

$$\text{logit}(v) = \log\left(\frac{v}{1-v}\right).$$

The inverse of the link function appears in the model.

$$\text{logit}^{-1}(u) = \frac{1}{1+\exp(-u)}.$$

The model formulation above uses the logit-parameterized version of the Bernoulli distribution, which is defined by

$$\mathsf{BernoulliLogit}(y|\alpha) = \mathsf{Bernoulli}(y|\text{logit}^{-1}(\alpha)).$$

The formulation is also vectorized in the sense that alpha and beta are scalars and x is a vector, so that alpha + beta * x is a vector. The vectorized formulation is equivalent to the less efficient version

```
for (n in 1:N)
  y[n] ~ bernoulli_logit(alpha + beta * x[n]);
```

Expanding out the Bernoulli logit, the model is equivalent to the more explicit, but less efficient and less arithmetically stable

```
for (n in 1:N)
  y[n] ~ bernoulli(inv_logit(alpha + beta * x[n]));
```

Other link functions may be used in the same way. For example, probit regression uses the cumulative normal distribution function, which is typically written as

$$\Phi(x) = \int_{-\infty}^{x} \mathsf{Normal}(y|0,1)\,dy.$$

The cumulative unit normal distribution function Φ is implemented in Stan as the function `Phi`. The probit regression model may be coded in Stan by replacing the logistic model's sampling statement with the following.

```
y[n] ~ bernoulli(Phi(alpha + beta * x[n]));
```

A fast approximation to the cumulative unit normal distribution function Φ is implemented in Stan as the function `Phi_approx`. The approximate probit regression model may be coded with the following.

```
y[n] ~ bernoulli(Phi_approx(alpha + beta * x[n]));
```

5.5. Multi-Logit Regression

Multiple outcome forms of logistic regression can be coded directly in Stan. For instance, suppose there are K possible outcomes for each output variable y_n. Also suppose that there is a D-dimensional vector x_n of predictors for y_n. The multi-logit model with $\mathsf{Normal}(0,5)$ priors on the coefficients is coded as follows.

```
data {
  int K;
  int N;
  int D;
  int y[N];
  vector[D] x[N];
}
parameters {
  matrix[K,D] beta;
}
```

```
model {
  for (k in 1:K)
    for (d in 1:D)
      beta[k,d] ~ normal(0,5);
  for (n in 1:N)
    y[n] ~ categorical(softmax(beta * x[n]));
}
```

The softmax function is defined for a K-vector $y \in \mathbb{R}^K$ by

$$\text{softmax}(y) = \left(\frac{\exp(y_1)}{\sum_{k=1}^{K} \exp(y_k)}, \ldots, \frac{\exp(y_K)}{\sum_{k=1}^{K} \exp(y_k)} \right).$$

The result is in the unit K-simplex and thus appropriate to use as the parameter for a categorical distribution.

Constraints on Data Declarations

The data block in the above model is defined without constraints on sizes `K`, `N`, and `D` or on the outcome array `y`. Constraints on data declarations provide error checking at the point data is read (or transformed data is defined), which is before sampling begins. Constraints on data declarations also make the model author's intentions more explicit, which can help with readability. The above model's declarations could be tightened to

```
int<lower=2> K;
int<lower=0> N;
int<lower=1> D;
int<lower=1,upper=K> y[N];
```

These constraints arise because the number of categories, `K`, must be at least two in order for a categorical model to be useful. The number of data items, `N`, can be zero, but not negative; unlike R, Stan's for-loops always move forward, so that a loop extent of `1:N` when `N` is equal to zero ensures the loop's body will not be executed. The number of predictors, `D`, must be at least one in order for `beta * x[n]` to produce an appropriate argument for `softmax()`. The categorical outcomes `y[n]` must be between `1` and `K` in order for the discrete sampling to be well defined.

Constraints on data declarations are optional. Constraints on parameters declared in the `parameters` block, on the other hand, are *not* optional—they are required to ensure support for all parameter values satisfying their constraints. Constraints on transformed data, transformed parameters, and generated quantities are also optional.

Identifiability

Because softmax is invariant under adding a constant to each component of its input, the model is typically only identified if there is a suitable prior on the coefficients.

An alternative is to use $K - 1$ vectors by fixing one of them to be zero. See Section 7.2 for an example of how to mix known quantities and unknown quantities in a vector.

5.6. Ordered Logistic and Probit Regression

Ordered regression for an outcome $y_n \in \{1, \ldots, K\}$ with predictors $x_n \in \mathbb{R}^D$ is determined by a single coefficient vector $\beta \in \mathbb{R}^D$ along with a sequence of cutpoints $c \in \mathbb{R}^{D-1}$ sorted so that $c_d < c_{d+1}$. The discrete output is k if the linear predictor $x_n\beta$ falls between c_{k-1} and c_k, assuming $c_0 = -\infty$ and $c_K = \infty$. The noise term is fixed by the form of regression, with examples for ordered logistic and ordered probit models.

Ordered Logistic Regression

The ordered logistic model can be coded in Stan using the `ordered` data type for the cutpoints and the built-in `ordered_logistic` distribution.

```
data {
  int<lower=2> K;
  int<lower=0> N;
  int<lower=1> D;
  int<lower=1,upper=K> y[N];
  row_vector[D] x[N];
}
parameters {
  vector[D] beta;
  ordered[K-1] c;
}
model {
  for (n in 1:N)
    y[n] ~ ordered_logistic(x[n] * beta, c);
}
```

The vector of cutpoints `c` is declared as `ordered[K-1]`, which guarantees that `c[k]` is less than `c[k+1]`.

If the cutpoints were assigned independent priors, the constraint effectively truncates the joint prior to support over points that satisfy the ordering constraint. Luckily, Stan does not need to compute the effect of the constraint on the normalizing term because the probability is needed only up to a proportion.

Ordered Probit

An ordered probit model could be coded in exactly the same way by swapping the cumultive logistic (`inv_logit`) for the cumulative normal (`Phi`).

```
data {
  int<lower=2> K;
  int<lower=0> N;
  int<lower=1> D;
  int<lower=1,upper=K> y[N];
  row_vector[D] x[N];
}
parameters {
  vector[D] beta;
  ordered[K-1] c;
}
model {
  vector[K] theta;
  for (n in 1:N) {
    real eta;
    eta <- x[n] * beta;
    theta[1] <- 1 - Phi(eta - c[1]);
    for (k in 2:(K-1))
      theta[k] <- Phi(eta - c[k-1]) - Phi(eta - c[k]);
    theta[K] <- Phi(eta - c[K-1]);
    y[n] ~ categorical(theta);
  }
}
```

The logistic model could also be coded this way by replacing `Phi` with `inv_logit`, though the built-in encoding based on the softmax transform is more efficient and more numerically stable. A small efficiency gain could be achieved by computing the values `Phi(eta - c[k])` once and storing them for re-use.

5.7. Hierarchical Logistic Regression

The simplest multilevel model is a hierarchical model in which the data is grouped into L distinct categories (or levels). An extreme approach would be to completely pool all the data and estimate a common vector of regression coefficients β. At the other extreme, an approach would no pooling assigns each level l its own coefficient vector β_l that is estimated separately from the other levels. A hierarchical model is an intermediate solution where the degree of pooling is determined by the data and a prior on the amount of pooling.

Suppose each binary outcome $y_n \in \{0, 1\}$ has an associated level, $ll_n \in \{1, \ldots, L\}$. Each outcome will also have an associated predictor vector $x_n \in \mathbb{R}^D$. Each level l gets its own coefficient vector $\beta_l \in \mathbb{R}^D$. The hierarchical structure involves drawing the coefficients $\beta_{l,d} \in \mathbb{R}$ from a prior that is also estimated with the data. This hierarchically estimated prior determines the amount of pooling. If the data in each level are very similar, strong pooling will be reflected in low hierarchical variance. If the data in the levels are dissimilar, weaker pooling will be reflected in higher hierarchical variance.

The following model encodes a hierarchical logistic regression model with a hierarchical prior on the regression coefficients.

```
data {
  int<lower=1> D;
  int<lower=0> N;
  int<lower=1> L;
  int<lower=0,upper=1> y[N];
  int<lower=1,upper=L> ll[N];
  row_vector[D] x[N];
}
parameters {
  real mu[D];
  real<lower=0,upper=1000> sigma[D];
  vector[D] beta[L];
}
model {
  for (d in 1:D) {
    mu[d] ~ normal(0,100);
    for (l in 1:L)
      beta[l,d] ~ normal(mu[d],sigma[d]);
  }
  for (n in 1:N)
    y[n] ~ bernoulli(inv_logit(x[n] * beta[ll[n]]));
}
```

Optimizing the Model

Where possible, vectorizing sampling statements leads to faster log probability and derivative evaluations. The speed boost is not because loops are eliminated, but because vectorization allows sharing subcomputations in the log probability and gradient calculations and because it reduces the size of the expression tree required for gradient calculations.

The first optimization vectorizes the for-loop over `D` as

```
mu ~ normal(0,100);
```

```
for (l in 1:L)
  beta[l] ~ normal(mu,sigma);
```

The declaration of beta as an array of vectors means that the expression beta[l] denotes a vector. Although beta could have been declared as a matrix, an array of vectors (or a two-dimensional array) is more efficient for accessing rows; see Chapter 4 for more information on the efficiency tradeoffs among arrays, vectors, and matrices.

This model can be further sped up and at the same time made more arithmetically stable by replacing the application of inverse-logit inside the Bernoulli distribution with the logit-parameterized Bernoulli,

```
for (n in 1:N)
  y[n] ~ bernoulli_logit(x[n] * beta[ll[n]]);
```

See Section 35.2 for a definition of bernoulli_logit.

Unlike in R or BUGS, loops, array access and assignments are fast in Stan because they are translated directly to C++. In most cases, the cost of allocating and assigning to a container is more than made up for by the increased efficiency due to vectorizing the log probability and gradient calculations. Thus the following version is faster than the original formulation as a loop over a sampling statement.

```
{
  vector[N] x_beta_ll;
  for (n in 1:N)
    x_beta_ll[n] <- x[n] * beta[ll[n]];
  y ~ bernoulli_logit(x_beta_ll);
}
```

The brackets introduce a new scope for the local variable x_beta_ll; alternatively, the variable may be declared at the top of the model block.

In some cases, such as the above, the local variable assignment leads to models that are less readable. The recommended practice in such cases is to first develop and debug the more transparent version of the model and only work on optimizations when the simpler formulation has been debugged.

5.8. Hierarchical Priors

Priors on priors, also known as "hyperpriors," should be treated the same way as priors on lower-level parameters in that as much prior information as is available should be brought to bear. Because hyperpriors often apply to only a handful of lower-level parameters, care must be taken to ensure the posterior is both proper and not overly sensitive either statistically or computationally to wide tails in the priors.

Boundary-Avoiding Priors for MLE in Hierarchical Models

The fundamental problem with maximum likelihood estimation (MLE) in the hierarchical model setting is that as the hierarchical variance drops and the values cluster around the hierarchical mean, the overall density grows without bound. As an illustration, consider a simple hierarchical linear regression (with fixed prior mean) of $y_n \in \mathbb{R}$ on $x_n \in \mathbb{R}^K$, formulated as

$$\begin{aligned} y_n &\sim \text{Normal}(x_n\beta, \sigma) \\ \beta_k &\sim \text{Normal}(0, \tau) \\ \tau &\sim \text{Cauchy}(0, 2.5) \end{aligned}$$

In this case, as $\tau \to 0$ and $\beta_k \to 0$, the posterior density

$$p(\beta, \tau, \sigma | y, x) \propto p(y|x, \beta, \tau, \sigma)$$

grows without bound. There is a plot of a Neal's funnel density in Figure 19.1, which has similar behavior.

There is obviously no MLE estimate for β, τ, σ in such a case, and therefore the model must be modified if posterior modes are to be used for inference. The approach recommended by Chung et al. (2013) is to use a gamma distribution as a prior, such as

$$\sigma \sim \text{Gamma}(2, 1/A),$$

for a reasonably large value of A, such as $A = 10$.

5.9. Item-Response Theory Models

Item-response theory (IRT) models the situation in which a number of students each answer one or more of a group of test questions. The model is based on parameters for the ability of the students, the difficulty of the questions, and in more articulated models, the discriminativeness of the questions and the probability of guessing correctly; see (Gelman and Hill, 2007, pps. 314–320) for a textbook introduction to hierarchical IRT models and (Curtis, 2010) for encodings of a range of IRT models in BUGS.

Data Declaration with Missingness

The data provided for an IRT model may be declared as follows to account for the fact that not every student is required to answer every question.

```
data {
  int<lower=1> J;              // number of students
  int<lower=1> K;              // number of questions
  int<lower=1> N;              // number of observations
  int<lower=1,upper=J> jj[N];  // student for observation n
  int<lower=1,upper=K> kk[N];  // question for observation n
  int<lower=0,upper=1> y[N];   // correctness for observation n
}
```

This declares a total of `N` student-question pairs in the data set, where each `n` in `1:N` indexes a binary observation `y[n]` of the correctness of the answer of student `jj[n]` on question `kk[n]`.

The prior hyperparameters will be hard coded in the rest of this section for simplicity, though they could be coded as data in Stan for more flexibility.

1PL (Rasch) Model

The 1PL item-response model, also known as the Rasch model, has one parameter (1P) for questions and uses the logistic link function (L). This model is distributed with Stan in the file `example-models/misc/irt/irt.stan`.

The model parameters are declared as follows.

```
parameters {
  real delta;         // mean student ability
  real alpha[J];      // ability of student j - mean ability
  real beta[K];       // difficulty of question k
}
```

The parameter `alpha[j]` is the ability coefficient for student `j` and `beta[k]` is the difficulty coefficient for question `k`. The non-standard parameterization used here also includes an intercept term `delta`, which represents the average student's response to the average question.[5] The model itself is as follows.

```
model {
  alpha ~ normal(0,1);         // informative true prior
  beta ~ normal(0,1);          // informative true prior
  delta ~ normal(.75,1);       // informative true prior
  for (n in 1:N)
    y[n] ~ bernoulli_logit(alpha[jj[n]] - beta[kk[n]] + delta);
}
```

[5](Gelman and Hill, 2007) treat the δ term equivalently as the location parameter in the distribution of student abilities.

This model uses the logit-parameterized Bernoulli distribution, where

$$\texttt{bernoulli\_logit}(y|\alpha) = \texttt{bernoulli}(y|\text{logit}^{-1}(\alpha)).$$

The key to understanding it is the term inside the `bernoulli_logit` distribution, from which it follows that

$$\Pr[y_n = 1] = \text{logit}^{-1}(\alpha_{jj[n]} - \beta_{kk[n]} + \delta).$$

The model suffers from additive identifiability issues without the priors. For example, adding a term ξ to each α_j and β_k results in the same predictions. The use of priors for α and β located at 0 identifies the parameters; see (Gelman and Hill, 2007) for a discussion of identifiability issues and alternative approaches to identification.

For testing purposes, the IRT 1PL model distributed with Stan uses informative priors that match the actual data generation process used to simulate the data in R (the simulation code is supplied in the same directory as the models). This is unrealistic for most practical applications, but allows Stan's inferences to be validated. A simple sensitivity analysis with fatter priors shows that the posterior is fairly sensitive to the prior even with 400 students and 100 questions and only 25% missingness at random. For real applications, the priors should be fit hierarchically along with the other parameters, as described in the next section.

Multilevel 2PL Model

The simple 1PL model described in the previous section is generalized in this section with the addition of a discrimination parameter to model how noisy a question is and by adding multilevel priors for the student and question parameters.

The model parameters are declared as follows.

```
parameters {
  real delta;                  // mean student ability
  real alpha[J];               // ability for j - mean
  real beta[K];                // difficulty for k
  real log_gamma[K];           // discrimination of k
  real<lower=0> sigma_alpha;   // scale of abilities
  real<lower=0> sigma_beta;    // scale of difficulties
  real<lower=0> sigma_gamma;   // scale of log discrimination
}
```

The parameters should be clearer after the model definition.

```
model {
  alpha ~ normal(0,sigma_alpha);
  beta ~ normal(0,sigma_beta);
```

```
    log_gamma ~ normal(0,sigma_gamma);
    delta ~ cauchy(0,5);
    sigma_alpha ~ cauchy(0,5);
    sigma_beta ~ cauchy(0,5);
    sigma_gamma ~ cauchy(0,5);
    for (n in 1:N)
      y[n] ~ bernoulli_logit(
                exp(log_gamma[kk[n]])
                * (alpha[jj[n]] - beta[kk[n]] + delta) );
  }
```

First, the predictor inside the `bernoulli_logit` term is equivalent to the predictor of the 1PL model multiplied by the discriminativeness for the question, `exp(log_gamma[kk[n]])`. The parameter `log_gamma[k]` represents how discriminative a question is, with log discriminations above 0 being less (because their exponentiation drives the predictor away from zero, which drives the prediction away from 0.5) and discriminations below 0 being more noisy (driving the predictor toward zero and hence the prediction toward 0.5).

An alternative to explicitly exponentiating the unconstrained discrimination parameter `log_gamma` would be to declare a discrimination parameter `gamma` with a constraint `<lower=0>` and provide a lognormal or other positive-constrained prior. Either way, the positive-constrained discrimination term identifies the signs in the model, while at the same time eliminating the unlikely possibility that there is a question that is easier for less able students to answer correctly.

The intercept term `delta` can't be modeled hierarchically, so it is given a weakly informative $\mathsf{Cauchy}(0,5)$ prior. Similarly, the scale terms, `sigma_alpha`, `sigma_beta`, and `sigma_gamma`, are given half-Cauchy priors. The truncation in the half-Cauchy prior is implicit; explicit truncation is not necessary because the log probability need only be calculated up to a proportion and the scale variables are constrained to $(0,\infty)$ by their declarations.

5.10. Priors for Identifiabilty

Location and Scale Invariance

One application of (hierarchical) priors is to identify the scale and/or location of a group of parameters. For example, in the IRT models discussed in the previous section, there is both a location and scale non-identifiability. With uniform priors, the posteriors will float in terms of both scale and location. See Section 18.1 for a simple example of the problems this poses for estimation.

The non-identifiability is resolved by providing a unit normal (i.e., $\mathsf{Normal}(0,1)$)

prior on one group of coefficients, such as the student abilities. With a unit normal prior on the student abilities, the IRT model is identified in that the posterior will produce a group of estimates for student ability parameters that have a sample mean of close to zero and a sample variance of close to one. The difficulty and discrimination parameters for the questions should then be given a diffuse, or ideally a hierarchical prior, which will identify these parameters by scaling and locating relative to the student ability parameters.

Collinearity

Another case in which priors can help provide identifiability is in the case of collinearity in a linear regression. In linear regression, if two predictors are collinear (i.e, one is a linear function of the other), then their coefficients will have a correlation of 1 (or -1) in the posterior. This leads to non-identifiability. By placing normal priors on the coefficients, the maximum likelihood solution of two duplicated preditors (trivially collinear) will be half the value than would be obtained by only including one.

Separability

In a logistic regression, if a predictor is positive in cases of 1 outcomes and negative in cases of 0 outcomes, then the maximum likelihood estimate for the coefficient for that predictor diverges to infinity. This divergence can be controlled by providing a prior for the coefficient, which will "shrink" the estimate back toward zero and thus identify the model in the posterior.

Similar problems arise for sampling with improper flat priors. The sampler will try to draw very large values. By providing a prior, the posterior will be concentrated around finite values, leading to well-behaved sampling.

5.11. Multivariate Priors for Hierarchical Models

In hierarchical regression models (and other situations), several individual-level variables may be assigned hierarchical priors. For example, a model with multiple varying intercepts and slopes within might assign them a multivariate prior.

As an example, the individuals might people and the outcome income, with predictors such as income and age, and the groups might be states or other geographic divisions. The effect of education level and age as well as an intercept might be allowed to vary by state. Furthermore, there might be state-level predictors, such as average state income and unemployment level.

Multivariate Regression Example

(Gelman and Hill, 2007, Chapter 13, Chapter 17) discuss a hierarchical model with N individuals organized into J groups. Each individual has a predictor row vector x_n of size K; to unify the notation, they assume that $x_{n,1} = 1$ is a fixed "intercept" predictor. To encode group membership, they assume individual n belongs to group $jj[n] \in 1{:}J$. Each individual n also has an observed outcome y_n taking on real values.

Likelihood

The model is a linear regression with slope and intercept coefficients varying by group, so that β_j is the coefficient K-vector for group j. The likelihood function for individual n is then just

$$y_n \sim \mathsf{Normal}(x_n \, \beta_{jj[n]}, \sigma) \text{ for } n \in 1{:}N.$$

Coefficient Prior

Gelman and Hill model the coefficient vectors β_j as being drawn from a multivariate distribution with mean vector μ and covariance matrix Σ,

$$\beta_j \sim \mathsf{MultiNormal}(\mu, \Sigma) \text{ for } j \in 1{:}J.$$

Below, we discuss the full model of Gelman and Hill, which uses group-level predictors to model μ; for now, we assume μ is a simple vector parameter.

Hyperpriors

For hierarchical modeling, the group-level mean vector μ and covariance matrix Σ must themselves be given priors. The group-level mean vector can be given a reasonable weakly-informative prior for independent coefficients, such as

$$\mu_j \sim \mathsf{Normal}(0, 5).$$

Of course, if more is known about the expected coefficient values $\beta_{j,k}$, this information can be incorporated into the prior for μ_k.

For the prior on the covariance matrix, Gelman and Hill suggest using a scaled inverse Wishart. That choice was motivated primarily by convenience as it is conjugate to the multivariate likelihood function and thus simplifies Gibbs sampling.

In Stan, there is no restriction to conjugacy for multivariate priors, and we in fact recommend a slightly different approach. Like Gelman and Hill, we decompose our

prior into a scale and a matrix, but are able to do so in a more natural way based on the actual variable scales and a correlation matrix. Specifically, we define

$$\Sigma = \text{diag\_matrix}(\tau)\,\Omega\,\text{diag\_matrix}(\tau),$$

where Ω is a correlation matrix and τ is the vector of coefficient scales.

The components of the scale vector τ can be given any reasonable prior for scales, but we recommend something weakly informative like a half-Cauchy distribution with a small scale, such as

$$\tau_k \sim \mathsf{Cauchy}(0, 2.5) \text{ for } k \in 1{:}J \text{ and } \tau_k > 0.$$

As for the prior means, if there is information about the scale of variation of coefficients across groups, it should be incorporated into the prior for τ. For large numbers of exchangeable coefficients, the components of τ itself (perhaps excluding the intercept) may themselves be given a hierarchical prior.

Our final recommendation is to give the correlation matrix Ω an LKJ prior with shape $\nu \geq 1$,

$$\Omega \sim \mathsf{LKJcorr}(\nu).$$

The LKJ correlation distribution is defined in Section 48.1, but the basic idea for modeling is that as ν increases, the prior increasingly concentrates around the unit correlation matrix (i.e., favors less correlation among the components of β_j). At $\nu = 1$, the LKJ correlation distribution reduces to the identity distribution over correlation matrices. The LKJ prior may thus be used to control the expected amount of correlation among the parameters β_j.

Group-Level Predictors for Prior Mean

To complete Gelman and Hill's model, suppose each group $j \in 1{:}J$ is supplied with an L-dimensional row-vector of group-level predictors u_j. The prior mean for the β_j can then itself be modeled as a regression, using an L-dimensional coefficient vector γ. The prior for the group-level coefficients then becomes

$$\beta_j \sim \mathsf{MultiNormal}(u_j\,\gamma, \Sigma)$$

The group-level coefficients γ may themselves be given independent weakly informative priors, such as

$$\gamma_l \sim \mathsf{Normal}(0, 5).$$

As usual, information about the group-level means should be incorporated into this prior.

Coding the Model in Stan

The Stan code for the full hierarchical model with multivariate priors on the group-level coefficients and group-level prior means follows its definition.

```
data {
  int<lower=0> N;              // num individuals
  int<lower=1> K;              // num ind predictors
  int<lower=1> J;              // num groups
  int<lower=1> L;              // num group predictors
  int<lower=1,upper=J> jj[N];  // group for individual
  matrix[N,K] x;               // individual predictors
  matrix[J,L] u;               // group predictors
  vector[N] y;                 // outcomes
}
parameters {
  corr_matrix[K] Omega;        // prior correlation
  vector<lower=0>[K] tau;      // prior scale
  matrix[L,K] gamma;           // group coeffs
  vector[K] beta[J];           // indiv coeffs by group
  real<lower=0> sigma;         // prediction error scale
}
model {
  matrix[K,K] Sigma_beta;
  Sigma_beta <- quad_form_diag(Omega,tau);

  tau ~ cauchy(0,2.5);
  Omega ~ lkj_corr(2);
  to_vector(gamma) ~ normal(0,5);

  for (j in 1:J)
    beta[j] ~ multi_normal((u[j] * gamma)', Sigma_beta);

  for (n in 1:N)
    y[n] ~ normal(x[n] * beta[jj[n]], sigma);
}
```

The hyperprior covariance matrix is defined as a local variable in the model because the correlation matrix `Omega` and scale vector `tau` are more natural to inspect in the output; to output `Sigma`, define it as a transformed parameter. The function `quad_form_diag` is defined so that `quad_form_diag(Sigma,tau)` is equivalent to `diag_matrix(tau) * Sigma * diag_matrix(tau)`, where `diag_matrix(tau)` returns the matrix with `tau` on the diagonal and zeroes off diagonal; the version using `quad_form_diag` should be faster. See Section 33.2 for more information on special-

ized matrix operations.

This is not the most optimal way to define this model; it can be sped up through vectorization and through reparameterization in terms of Cholesky factors.

Optimization through Vectorization

Another optimization would be to vectorize the likelihood sampling statement by generating a temporary vector of the linear predictor.

```
{
  vector[N] x_beta_jj;
  for (n in 1:N)
    x_beta_jj[n] <- x[n] * beta[jj[n]];
  y ~ normal(x_beta_jj, sigma);
}
```

The outer brackets provide a local scope in which to define `x_beta_jj`.

The multi-normal prior on `beta` can also be optimized through vectorization with a bit more work.

```
{
   vector[K] u_gamma_t[J];
   for (j in 1:J)
     u_gamma_t[j] <- (u[j] * gamma)';
   beta ~ multi_normal(u_gamma_t, Sigma_beta);
}
```

Optimization through Cholesky Factorization

The multivariate normal density and LKJ prior on correlation matrices both require their matrix parameters to be factored. Vectorizing, as in the previous section, ensures this is only done once for each density. An even better solution, both in terms of efficiency and numerical stability, is to parameterize the model directly in terms of Cholesky factors of correlation matrices using the multivariate version of the non-centered parameterization. For the model in the previous section, the program fragment to replace the full matrix prior with an equivalent Cholesky factorized prior is as follows.

```
parameters {
  matrix[K,J] z;
  cholesky_factor_corr[K] L_Omega;
  ...
```

```
transformed parameters {
  matrix[J,K] beta;
  beta <- u * gamma + (diag_pre_multiply(tau,L_Omega) * z)';
}
model {
  to_vector(z) ~ normal(0,1);
  L_Omega ~ lkj_corr_cholesky(2);
  ...
```

This new parameter L_Omega is the Cholesky factor of the original correlation matrix Omega, so that

```
Omega = L_Omega * L_Omega'
```

The prior scale vector tau is unchanged, and furthermore, Pre-multiplying the Cholesky factor by the scale produces the Cholesky factor of the final covariance matrix,

```
Sigma_beta
= quad_form_diag(Omega,tau)
= diag_pre_multiply(tau,L_Omega) * diag_pre_multiply(tau,L_Omega)'
```

where the diagonal pre-multiply compound operation is defined by

```
diag_pre_multiply(a,b) = diag_matrix(a) * b
```

The new variable z is declared as a matrix, the entries of which are given independent unit normal priors; the to_vector operation turns the matrix into a vector so that it can be used as a vectorized argument to the univariate normal density. Multiplying the Cholesky factor of the covariance matrix by z and adding the mean (u * gamma)' produces a beta distributed as in the original model.

Omitting the data declarations, which are the same as before, the optimized model is as follows.

```
parameters {
  matrix[K,J] z;
  cholesky_factor_corr[K] L_Omega;
  vector<lower=0>[K] tau;      // prior scale
  matrix[L,K] gamma;           // group coeffs
  real<lower=0> sigma;         // prediction error scale
}
transformed parameters {
  matrix[J,K] beta;
  beta <- u * gamma + (diag_pre_multiply(tau,L_Omega) * z)';
```

```
}
model {
  vector[N] x_beta_jj;
  for (n in 1:N)
    x_beta_jj[n] <- x[n] * beta[jj[n]]';
  y ~ normal(x_beta_jj, sigma);

  tau ~ cauchy(0,2.5);
  to_vector(z) ~ normal(0,1);
  L_Omega ~ lkj_corr_cholesky(2);
  to_vector(gamma) ~ normal(0,5);
}
```

5.12. Prediction, Forecasting, and Backcasting

Stan models can be used for "predicting" the values of arbitrary model unknowns. When predictions are about the future, they're called "forecasts;" when they are predictions about the past, as in climate reconstruction or cosmology, they are sometimes called "backcasts" (or "aftcasts" or "hindcasts" or "antecasts," depending on the author's feelings about the opposite of "fore").

Programming Predictions

As a simple example, the following linear regression provides the same setup for estimating the coefficients beta as in our very first example above, using y for the N observations and x for the N predictor vectors. The model parameters and model for observations are exactly the same as before.

To make predictions, we need to be given the number of predictions, N_new, and their predictor matrix, x_new. The predictions themselves are modeled as a parameter y_new. The model statement for the predictions is exactly the same as for the observations, with the new outcome vector y_new and prediction matrix x_new.

```
data {
  int<lower=1> K;
  int<lower=0> N;
  matrix[N,K] x;
  vector[N] y;

  int<lower=0> N_new;
  matrix[N_new, K] x_new;
}
parameters {
```

```
  vector[K] beta;
  real<lower=0> sigma;

  vector[N_new] y_new;                  // predictions
}
model {
  y ~ normal(x * beta, sigma);          // observed model

  y_new ~ normal(x_new * beta, sigma);  // prediction model
}
```

Predictions as Generated Quantities

Where possible, the most efficient way to generate predictions is to use the generated quantities block. This provides proper Monte Carlo (not Markov chain Monte Carlo) inference, which can have a much higher effective sample size per iteration.

```
...data as above...

parameters {
  vector[K] beta;
  real<lower=0> sigma;
}
model {
  y ~ normal(x * beta, sigma);
}
generated quantities {
  vector[N_new] y_new;
  for (n in 1:N_new)
    y_new[n] <- normal_rng(x_new[n] * beta, sigma);
}
```

Now the data is just as before, but the parameter `y_new` is now declared as a generated quantity, and the prediction model is removed from the model and replaced by a pseudo-random draw from a normal distribution.

6. Time-Series Models

Times series data come arranged in temporal order. This chapter presents two kinds of time series models, regression-like models such as autogression and moving average models, and hidden Markov models.

In later chapters, we discuss two alternative models which may be applied to time-series data,

- Gaussian processes (GP) in Chapter 13 and
- hidden Markov models (HMM) in Section 6.6.

6.1. Autoregressive Models

A first-order autoregressive model (AR(1)) with normal noise takes each point y_n in a sequence y to be generated according to

$$y_n \sim \mathsf{Normal}(\alpha + \beta y_{n-1}, \sigma).$$

That is, the expected value of y_n is $\alpha + \beta y_{n-1}$, with noise scaled as σ.

AR(1) Models

With improper flat priors on the regression coefficients for slope (β), intercept (α), and noise scale (σ), the Stan program for the AR(1) model is as follows.

```
data {
  int<lower=0> N;
  vector[N] y;
}
parameters {
  real alpha;
  real beta;
  real sigma;
}
model {
  for (n in 2:N)
    y[n] ~ normal(alpha + beta * y[n-1], sigma);
}
```

The first observed data point, `y[1]`, is not modeled here because there is nothing to condition on; instead, it acts to condition `y[2]`. This model also uses an improper prior for `sigma`, but there is no obstacle to adding an informative prior if information is available on the scale of the changes in `y` over time, or a weakly informative prior to help guide inference if rough knowledge of the scale of `y` is available.

Slicing for Efficiency

Although perhaps a bit more difficult to read, a much more efficient way to write the above model is by slicing the vectors, with the model above being replaced with the one-liner

```
model {
  tail(y, N - 1) ~ normal(alpha + beta * head(y, N - 1), sigma);
}
```

The tail operation pulls out the last `N - 1` elements of `y` and the head operation pulls out the first `N - 1`; vector arithmetic is used to multiply the head elements by `beta`.

Extensions to the AR(1) Model

Proper priors of a range of different families may be added for the regression coefficients and noise scale. The normal noise model can be changed to a Student-t distribution or any other distribution with unbounded support. The model could also be made hierarchical if multiple series of observations are available.

To enforce the estimation of a stationary AR(1) process, the slope coefficient `beta` may be constrained with bounds as follows.

```
real<lower=-1,upper=1> beta;
```

In practice, such a constraint is not recommended. If the data is not stationary, it is best to discover this while fitting the model. Stationary parameter estimates can be encouraged with a prior favoring values of `beta` near zero.

AR(2) Models

Extending the order of the model is also straightforward. For example, an AR(2) model could be coded with the second-order coefficient `gamma` and the following model statement.

```
for (n in 3:N)
  y[n] ~ normal(alpha + beta*y[n-1] + gamma*y[n-2], sigma);
```

AR(K) Models

A general model where the order is itself given as data can be coded by putting the coefficients in an array and computing the linear predictor in a loop.

```
data {
  int<lower=0> K;
  int<lower=0> N;
  real y[N];
}
parameters {
  real alpha;
  real beta[K];
  real sigma;
}
model {
  for (n in (K+1):N) {
    real mu;
    mu <- alpha;
    for (k in 1:K)
      mu <- mu + beta[k] * y[n-k];
    y[n] ~ normal(mu, sigma);
  }
}
```

ARCH(1) Models

Econometric and financial time-series models usually assume heteroscedasticity (i.e., they allow the scale of the noise terms defining the series to vary over time). The simplest such model is the autoregressive conditional heteroscedasticity (ARCH) model (Engle, 1982). Unlike the autoregressive model AR(1), which modeled the mean of the series as varying over time but left the noise term fixed, the ARCH(1) model takes the scale of the noise terms to vary over time but leaves the mean term fixed. Of course, models could be defined where both the mean and scale vary over time; the econometrics literature presents a wide range of time-series modeling choices.

The ARCH(1) model is typically presented as the following sequence of equations, where r_t is the observed return at time point t and μ, α_0, and α_1 are unknown regression coefficient parameters.

$$
\begin{aligned}
r_t &= \mu + a_t \\
a_t &= \sigma_t \epsilon_t \\
\epsilon_t &\sim \mathsf{Normal}(0, 1) \\
\sigma_t^2 &= \alpha_0 + \alpha_1 a_{t-1}^2
\end{aligned}
$$

In order to ensure the noise terms σ_t^2 are positive, the scale coefficients are constrained to be positive, $\alpha_0, \alpha_1 > 0$. To ensure stationarity of the time series, the slope

is constrained to to be less than one, $\alpha_1 < 1$.[1] The ARCH(1) model may be coded directly in Stan as follows.

```
data {
  int<lower=0> T;   // number of time points
  real r[T];        // return at time t
}
parameters {
  real mu;                          // average return
  real<lower=0> alpha0;             // noise intercept
  real<lower=0,upper=1> alpha1;     // noise slope
}
model {
  for (t in 2:T)
    r[t] ~ normal(mu, sqrt(alpha0 + alpha1 * pow(r[t-1] - mu,2)));
}
```

The loop in the model is defined so that the return at time $t = 1$ is not modeled; the model in the next section shows how to model the return at $t = 1$. The model can be vectorized to be more efficient; the model in the next section provides an example.

6.2. Modeling Temporal Heteroscedasticity

A set of variables is homoscedastic if their variances are all the same; the variables are heteroscedastic if they do not all have the same variance. Heteroscedastic time-series models allow the noise term to vary over time.

GARCH(1,1) Models

The basic generalized autoregressive conditional heteroscedasticity (GARCH) model, GARCH(1,1), extends the ARCH(1) model by including the squared previous difference in return from the mean at time $t - 1$ as a predictor of volatility at time t, defining

$$\sigma_t^2 = \alpha_0 + \alpha_1 a_{t-1}^2 + \beta_1 \sigma_{t-1}^2.$$

To ensure the scale term is positive and the resulting time series stationary, the coefficients must all satisfy $\alpha_0, \alpha_1, \beta_1 > 0$ and the slopes $\alpha_1 + \beta_1 < 1$.

```
data {
  int<lower=0> T;
```

[1] In practice, it can be useful to remove the constraint to test whether a non-stationary set of coefficients provides a better fit to the data. It can also be useful to add a trend term to the model, because an unfitted trend will manifest as non-stationarity.

```
  real r[T];
  real<lower=0> sigma1;
}
parameters {
  real mu;
  real<lower=0> alpha0;
  real<lower=0,upper=1> alpha1;
  real<lower=0,upper=(1-alpha1)> beta1;
}
transformed parameters {
  real<lower=0> sigma[T];
  sigma[1] <- sigma1;
  for (t in 2:T)
    sigma[t] <- sqrt(alpha0
                     + alpha1 * pow(r[t-1] - mu, 2)
                     + beta1 * pow(sigma[t-1], 2));
}
model {
  r ~ normal(mu,sigma);
}
```

To get the recursive definition of the volatility regression off the ground, the data declaration includes a non-negative value `sigma1` for the scale of the noise at $t = 1$.

The constraints are coded directly on the parameter declarations. This declaration is order-specific in that the constraint on `beta1` depends on the value of `alpha1`.

A transformed parameter array of non-negative values `sigma` is used to store the scale values at each time point. The definition of these values in the transformed parameters block is where the regression is now defined. There is an intercept `alpha0`, a slope `alpha1` for the squared difference in return from the mean at the previous time, and a slope `beta1` for the previous noise scale squared. Finally, the whole regression is inside the `sqrt` function because Stan requires scale (deviation) parameters (not variance parameters) for the normal distribution.

With the regression in the transformed parameters block, the model reduces a single vectorized sampling statement. Because `r` and `sigma` are of length `T`, all of the data is modeled directly.

6.3. Moving Average Models

A moving average model uses previous errors as predictors for future outcomes. For a moving average model of order Q, $\mathrm{MA}(Q)$, there is an overall mean parameter μ and regression coefficients θ_q for previous error terms. With ϵ_t being the noise at time t,

the model for outcome y_t is defined by

$$y_t = \mu + \theta_1 \epsilon_{t-1} + \cdots + \theta_Q \epsilon_{t-Q} + \epsilon_t,$$

with the noise term ϵ_t for outcome y_t modeled as normal,

$$\epsilon_t \sim \mathsf{Normal}(0, \sigma).$$

In a proper Bayesian model, the parameters μ, θ, and σ must all be given priors.

MA(2) Example

An MA(2) model can be coded in Stan as follows.

```
data {
  int<lower=3> T;  // number of observations
  vector[T] y;     // observation at time T
}
parameters {
  real mu;              // mean
  real<lower=0> sigma;  // error scale
  vector[2] theta;      // lag coefficients
}
transformed parameters {
  vector[T] epsilon;    // error terms
  epsilon[1] <- y[1] - mu;
  epsilon[2] <- y[2] - mu - theta[1] * epsilon[1];
  for (t in 3:T)
    epsilon[t] <- ( y[t] - mu
                    - theta[1] * epsilon[t - 1]
                    - theta[2] * epsilon[t - 2] );
}
model {
  mu ~ cauchy(0,2.5);
  theta ~ cauchy(0,2.5);
  sigma ~ cauchy(0,2.5);
  for (t in 3:T)
    y[t] ~ normal(mu
                  + theta[1] * epsilon[t - 1]
                  + theta[2] * epsilon[t - 2],
                  sigma);
}
```

The error terms ϵ_t are defined as transformed parameters in terms of the observations and parameters. The definition of the sampling statement (defining the likelihood) follows the definition, which can only be applied to y_n for $n > Q$. In this

example, the parameters are all given Cauchy (half-Cauchy for σ) priors, although other priors can be used just as easily.

This model could be improved in terms of speed by vectorizing the sampling statement in the model block. Vectorizing the calculation of the ϵ_t could also be sped up by using a dot product instead of a loop.

Vectorized MA(Q) Model

A general MA(Q) model with a vectorized sampling probability may be defined as follows.

```
data {
  int<lower=0> Q;  // num previous noise terms
  int<lower=3> T;  // num observations
  vector[T] y;     // observation at time t
}
parameters {
  real mu;              // mean
  real<lower=0> sigma;  // error scale
  vector[Q] theta;      // error coeff, lag -t
}
transformed parameters {
  vector[T] epsilon;    // error term at time t
  for (t in 1:T) {
    epsilon[t] <- y[t] - mu;
    for (q in 1:min(t-1,Q))
      epsilon[t] <- epsilon[t] - theta[q] * epsilon[t - q];
  }
}
model {
  vector[T] eta;
  mu ~ cauchy(0,2.5);
  theta ~ cauchy(0,2.5);
  sigma ~ cauchy(0,2.5);
  for (t in 1:T) {
    eta[t] <- mu;
    for (q in 1:min(t-1,Q))
      eta[t] <- eta[t] + theta[q] * epsilon[t - q];
  }
  y ~ normal(eta,sigma);
}
```

Here all of the data is modeled, with missing terms just dropped from the regressions as in the calculation of the error terms. Both models converge very quickly and

mix very well at convergence, with the vectorized model being quite a bit faster (per iteration, not to converge — they compute the same model).

6.4. Autoregressive Moving Average Models

Autoregressive moving-average models (ARMA), combine the predictors of the autoregressive model and the oving average model. An ARMA(1,1) model, with a single state of history, can be encoded in Stan as follows.

```
data {
  int<lower=1> T;             // num observations
  real y[T];                  // observed outputs
}
parameters {
  real mu;                    // mean coeff
  real phi;                   // autoregression coeff
  real theta;                 // moving avg coeff
  real<lower=0> sigma;        // noise scale
}
model {
  vector[T] nu;               // prediction for time t
  vector[T] err;              // error for time t
  nu[1] <- mu + phi * mu;     // assume err[0] == 0
  err[1] <- y[1] - nu[1];
  for (t in 2:T) {
    nu[t] <- mu + phi * y[t-1] + theta * err[t-1];
    err[t] <- y[t] - nu[t];
  }
  mu ~ normal(0,10);          // priors
  phi ~ normal(0,2);
  theta ~ normal(0,2);
  sigma ~ cauchy(0,5);
  err ~ normal(0,sigma);     // likelihood
}
```

The data is declared in the same way as the other time-series regressions. Here the are parameters for the mean output `mu` and error scale `sigma`, as well as regression coefficients `phi` for the autoregression and `theta` for the moving average component of the model.

In the model block, the local vector `nu` stores the predictions and `err` the errors. These are computed similarly to the errors in the moving average models described in the previous section.

The priors are weakly informative for stationary processes. The likelihood only involves the error term, which is efficiently vectorized here.

Often in models such as these, it is desirable to inspect the calculated error terms. This could easily be accomplished in Stan by declaring `err` as a transformed parameter, then defining it the same way as in the model above. The vector `nu` could still be a local variable, only now it will be in the transformed parameter block.

Wayne Folta suggested encoding the model without local vector variables as follows.

```
model {
  real err;
  mu ~ normal(0,10);
  phi ~ normal(0,2);
  theta ~ normal(0,2);
  sigma ~ cauchy(0,5);
  err <- y[1] - mu + phi * mu;
  err ~ normal(0,sigma);
  for (t in 2:T) {
    err <- y[t] - (mu + phi * y[t-1] + theta * err);
    err ~ normal(0,sigma);
  }
}
```

This approach to ARMA models provides a nice example of how local variables, such as `err` in this case, can be reused in Stan. Folta's approach could be extended to higher order moving-average models by storing more than one error term as a local variable and reassigning them in the loop.

Both encodings are very fast. The original encoding has the advantage of vectorizing the normal distribution, but it uses a bit more memory. A halfway point would be to vectorize just `err`.

6.5. Stochastic Volatility Models

Stochastic volatility models treat the volatility (i.e., variance) of a return on an asset, such as an option to buy a security, as following a latent stochastic process in discrete time (Kim et al., 1998). The data consist of mean corrected (i.e., centered) returns y_t on an underlying asset at T equally spaced time points. Kim et al. formulate a typical stochastic volatility model using the following regression-like equations, with a latent parameter h_t for the log volatility, along with parameters μ for the mean log volatility, and ϕ for the persistence of the volatility term. The variable ϵ_t represents the white-noise shock (i.e., multiplicative error) on the asset return at time t, whereas

δ_t represents the shock on volatility at time t.

$$y_t = \epsilon_t \exp(h_t/2),$$

$$h_{t+1} = \mu + \phi(h_t - \mu) + \delta_t \sigma$$

$$h_1 \sim \text{Normal}\left(\mu, \frac{\sigma}{\sqrt{1-\phi^2}}\right)$$

$$\epsilon_t \sim \text{Normal}(0,1); \quad \delta_t \sim \text{Normal}(0,1)$$

Rearranging the first line, $\epsilon_t = y_t \exp(-h_t/2)$, allowing the sampling distribution for y_t to be written as

$$y_t \sim \text{Normal}(0, \exp(h_t/2)).$$

The recurrence equation for h_{t+1} may be combined with the scaling and sampling of δ_t to yield the sampling distribution

$$h_t \sim \text{Normal}(\mu + \phi(h_t - \mu), \sigma).$$

This formulation can be directly encoded, as shown in the following Stan model.

```
data {
  int<lower=0> T;   // # time points (equally spaced)
  vector[T] y;      // mean corrected return at time t
}
parameters {
  real mu;                     // mean log volatility
  real<lower=-1,upper=1> phi;  // persistence of volatility
  real<lower=0> sigma;         // white noise shock scale
  vector[T] h;                 // log volatility at time t
}
model {
  phi ~ uniform(-1,1);
  sigma ~ cauchy(0,5);
  mu ~ cauchy(0,10);
  h[1] ~ normal(mu, sigma / sqrt(1 - phi * phi));
  for (t in 2:T)
    h[t] ~ normal(mu + phi * (h[t - 1] -  mu), sigma);
  for (t in 1:T)
    y[t] ~ normal(0, exp(h[t] / 2));
}
```

Compared to the Kim et al. formulation, the Stan model adds priors for the parameters ϕ, σ, and μ. Note that the shock terms ϵ_t and δ_t do not appear explicitly in the model, although they could be calculated efficiently in a generated quantities block.

The posterior of a stochastic volatility model such as this one typically has high posterior variance. For example, simulating 500 data points from the above model with $\mu = -1.02$, $\phi = 0.95$, and $\sigma = 0.25$ leads to 95% posterior intervals for μ of $(-1.23, -0.54)$, for ϕ of $(0.82, 0.98)$ and for σ of $(0.16, 0.38)$.

The samples using NUTS show a high degree of autocorrelation among the samples, both for this model and the stochastic volatility model evaluated in (Hoffman and Gelman, 2011, 2013). Using a non-diagonal mass matrix provides faster convergence and more effective samples than a diagonal mass matrix, but will not scale to large values of T.

It is relatively straightforward to speed up the effective samples per second generated by this model by one or more orders of magnitude. First, the sampling statements for return y is easily vectorized to

```
y ~ normal(0, exp(h / 2));
```

This speeds up the iterations, but does not change the effective sample size because the underlying parameterization and log probability function have not changed. Mixing is improved by by reparameterizing in terms of a standardized volatility, then rescaling. This requires a standardized parameter `h_std` to be declared instead of `h`.

```
parameters {
  ...
  vector[T] h_std;              // std log volatility time t
```

The original value of `h` is then defined in a transformed parameter block.

```
transformed parameters {
  vector[T] h;             // log volatility at time t
  h <- h_std * sigma;      // now h ~ normal(0,sigma)
  h[1] <- h[1] / sqrt(1 - phi * phi);  // rescale h[1]
  h <- h + mu;
  for (t in 2:T)
    h[t] <- h[t] + phi * (h[t-1] - mu);
}
```

The first assignment rescales `h_std` to have a $\text{Normal}(0, \sigma)$ distribution and temporarily assigns it to `h`. The second assignment rescales `h[1]` so that its prior differs from that of `h[2]` through `h[T]`. The next assignment supplies a `mu` offset, so that `h[2]` throgh `h[T]` are now distributed $\text{Normal}(\mu, \sigma)$; note that this shift must be done after the rescaling of `h[1]`. The final loop adds in the moving average so that `h[2]` through `h[T]` are appropriately modeled relative to `phi` and `mu`.

As a final improvement, the sampling statement for `h[1]` and loop for sampling `h[2]` to `h[T]` are replaced with a single vectorized unit normal sampling statement.

```
model {
  ...
  h_std ~ normal(0,1);
```

Although the original model can take hundreds and sometimes thousands of iterations to converge, the reparameterized model reliably converges in tens of iterations. Mixing is also dramatically improved, which results in higher effective sample sizes per iteration. Finally, each iteration runs in roughly a quarter of the time of the original iterations.

6.6. Hidden Markov Models

A hidden Markov model (HMM) generates a sequence of T output variables y_t conditioned on a parallel sequence of latent categorical state variables $z_t \in \{1, \ldots, K\}$. These "hidden" state variables are assumed to form a Markov chain so that z_t is conditionally independent of other variables given z_{t-1}. This Markov chain is parameterized by a transition matrix θ where θ_k is a K-simplex for $k \in \{1, \ldots, K\}$. The probability of transitioning to state z_t from state z_{t-1} is

$$z_t \sim \mathsf{Categorical}(\theta_{z[t-1]}).$$

The output y_t at time t is generated conditionally independently based on the latent state z_t.

This section describes HMMs with a simple categorical model for outputs $y_t \in \{1, \ldots, V\}$. The categorical distribution for latent state k is parameterized by a V-simplex ϕ_k. The observed output y_t at time t is generated based on the hidden state indicator z_t at time t,

$$y_t \sim \mathsf{Categorical}(\phi_{z[t]}).$$

In short, HMMs form a discrete mixture model where the mixture component indicators form a latent Markov chain.

Supervised Parameter Estimation

In the situation where the hidden states are known, the following naive model can be used to fit the parameters θ and ϕ. (This model is distributed with Stan on the path `<stan>/example-models/misc/hmm/hmm.stan`.)

```
data {
  int<lower=1> K;  // num categories
  int<lower=1> V;  // num words
  int<lower=0> T;  // num instances
  int<lower=1,upper=V> w[T]; // words
```

```
  int<lower=1,upper=K> z[T]; // categories
  vector<lower=0>[K] alpha;  // transit prior
  vector<lower=0>[V] beta;   // emit prior
}
parameters {
  simplex[K] theta[K];  // transit probs
  simplex[V] phi[K];    // emit probs
}
model {
  for (k in 1:K)
    theta[k] ~ dirichlet(alpha);
  for (k in 1:K)
    phi[k] ~ dirichlet(beta);
  for (t in 1:T)
    w[t] ~ categorical(phi[z[t]]);
  for (t in 2:T)
    z[t] ~ categorical(theta[z[t - 1]]);
}
```

Explicit Dirichlet priors have been provided for θ_k and ϕ_k; dropping these two statements would implicitly take the prior to be uniform over all valid simplexes.

Start-State and End-State Probabilities

Although workable, the above description of HMMs is incomplete because the start state z_1 is not modeled (the index runs from 2 to T). If the data are conceived as a subsequence of a long-running process, the probability of z_1 should be set to the stationary state probabilities in the Markov chain. In this case, there is no distinct end to the data, so there is no need to model the probability that the sequence ends at z_T.

An alternative conception of HMMs is as models of finite-length sequences. For example, human language sentences have distinct starting distributions (usually a capital letter) and ending distributions (usually some kind of punctuation). The simplest way to model the sequence boundaries is to add a new latent state $K+1$, generate the first state from a categorical distribution with parameter vector θ_{K+1}, and restrict the transitions so that a transition to state $K + 1$ is forced to occur at the end of the sentence and is prohibited elsewhere.

Calculating Sufficient Statistics

The naive HMM estimation model presented above can be sped up dramatically by replacing the loops over categorical distributions with a single multinomial distribution. A complete implementation is available in the Stan source distribution at path

<stan>/example-models/misc/hmm/hmm-sufficient.stan. The data is declared as before, but now a transformed data blocks computes the sufficient statistics for estimating the transition and emission matrices.

```
transformed data {
  int<lower=0> trans[K,K];
  int<lower=0> emit[K,V];
  for (k1 in 1:K)
    for (k2 in 1:K)
      trans[k1,k2] <- 0;
  for (t in 2:T)
    trans[z[t - 1], z[t]] <- 1 + trans[z[t - 1], z[t]];
  for (k in 1:K)
    for (v in 1:V)
      emit[k,v] <- 0;
  for (t in 1:T)
    emit[z[t], w[t]] <- 1 + emit[z[t], w[t]];
}
```

The likelihood component of the model based on looping over the input is replaced with multinomials as follows.

```
model {
  ...
  for (k in 1:K)
    trans[k] ~ multinomial(theta[k]);
  for (k in 1:K)
    emit[k] ~ multinomial(phi[k]);
}
```

In a continuous HMM with normal emission probabilities could be sped up in the same way by computing sufficient statistics.

Analytic Posterior

With the Dirichlet-multinomial HMM, the posterior can be computed analytically because the Dirichlet is the conjugate prior to the multinomial. The following example, available in <stan>/example-models/hmm/hmm-analytic.stan, illustrates how a Stan model can define the posterior analytically. This is possible in the Stan language because the model only needs to define the conditional probability of the parameters given the data up to a proportion, which can be done by defining the (unnormalized) joint probability or the (unnormalized) conditional posterior, or anything in between.

The model has the same data and parameters as the previous models, but now computes the posterior Dirichlet parameters in the transformed data block.

```
transformed data {
  vector<lower=0>[K] alpha_post[K];
  vector<lower=0>[V] beta_post[K];
  for (k in 1:K)
    alpha_post[k] <- alpha;
  for (t in 2:T)
    alpha_post[z[t-1],z[t]] <- alpha_post[z[t-1],z[t]] + 1;
  for (k in 1:K)
    beta_post[k] <- beta;
  for (t in 1:T)
    beta_post[z[t],w[t]] <- beta_post[z[t],w[t]] + 1;
}
```

The posterior can now be written analytically as follows.

```
model {
  for (k in 1:K)
    theta[k] ~ dirichlet(alpha_post[k]);
  for (k in 1:K)
    phi[k] ~ dirichlet(beta_post[k]);
}
```

Semisupervised Estimation

HMMs can be estimated in a fully unsupervised fashion without any data for which latent states are known. The resulting posteriors are typically extremely multimodal. An intermediate solution is to use semisupervised estimation, which is based on a combination of supervised and unsupervised data. Implementing this estimation strategy in Stan requires calculating the probability of an output sequence with an unknown state sequence. This is a marginalization problem, and for HMMs, it is computed with the so-called forward algorithm.

In Stan, the forward algorithm is coded as follows (the full model is in `<stan>/example-models/misc/hmm/hmm-semisup.stan`). First, two additional data variable are declared for the unsupervised data.

```
data {
  ...
  int<lower=1> T_unsup;  // num unsupervised items
  int<lower=1,upper=V> u[T_unsup]; // unsup words
  ...
```

The model for the supervised data does not change; the unsupervised data is handled with the following Stan implementation of the forward algorithm.

```
model {
 ...
  {
    real acc[K];
    real gamma[T_unsup,K];
    for (k in 1:K)
      gamma[1,k] <- log(phi[k,u[1]]);
    for (t in 2:T_unsup) {
      for (k in 1:K) {
        for (j in 1:K)
          acc[j] <- gamma[t-1,j] + log(theta[j,k]) + log(phi[k,u[t]]);
        gamma[t,k] <- log_sum_exp(acc);
      }
    }
    increment_log_prob(log_sum_exp(gamma[T_unsup]));
  }
```

The forward values gamma[t,k] are defined to be the log marginal probability of the inputs u[1],...,u[t] up to time t and the latent state being equal to k at time t; the previous latent states are marginalized out. The first row of gamma is initialized by setting gamma[1,k] equal to the log probability of latent state k generating the first output u[1]; as before, the probability of the first latent state is not itself modeled. For each subsequent time t and output j, the value acc[j] is set to the probability of the latent state at time t-1 being j, plus the log transition probability from state j at time t-1 to state k at time t, plus the log probability of the output u[t] being generated by state k. The log_sum_exp operation just multiplies the probabilities for each prior state j on the log scale in an arithmetically stable way.

The brackets provide the scope for the local variables acc and gamma; these could have been declared earlier, but it is clearer to keep their declaration near their use.

Predictive Inference

Given the transition and emission parameters, $\theta_{k,k'}$ and $\phi_{k,v}$ and an observation sequence $u_1, \ldots, u_T \in \{1, \ldots, V\}$, the Viterbi (dynamic programming) algorithm computes the state sequence which is most likely to have generated the observed output u.

The Viterbi algorithm can be coded in Stan in the generated quantities block as follows. The predictions here is the most likely state sequence y_star[1], ..., y_star[T_unsup] underlying the array of observations u[1], ..., u[T_unsup]. Because this sequence is determined from the transition probabilities theta and emission probabilities phi, it may be different from sample to sample in the posterior.

```
generated quantities {
  int<lower=1,upper=K> y_star[T_unsup];
  real log_p_y_star;
  {
    int back_ptr[T_unsup,K];
    real best_logp[T_unsup,K];
    real best_total_logp;
    for (k in 1:K)
      best_logp[1,K] <- log(phi[k,u[1]]);
    for (t in 2:T_unsup) {
      for (k in 1:K) {
        best_logp[t,k] <- negative_infinity();
        for (j in 1:K) {
          real logp;
          logp <- best_logp[t-1,j]
                  + log(theta[j,k]) + log(phi[k,u[t]]);
          if (logp > best_logp[t,k]) {
            back_ptr[t,k] <- j;
            best_logp[t,k] <- logp;
          }
        }
      }
    }
    log_p_y_star <- max(best_logp[T_unsup]);
    for (k in 1:K)
      if (best_logp[T_unsup,k] == log_p_y_star)
        y_star[T_unsup] <- k;
    for (t in 1:(T_unsup - 1))
      y_star[T_unsup - t] <- back_ptr[T_unsup - t + 1,
                                      y_star[T_unsup - t + 1]];
  }
}
```

The bracketed block is used to make the three variables back_ptr, best_logp, and best_total_logp local so they will not be output. The variable y_star will hold the label sequence with the highest probability given the input sequence u. Unlike the forward algorithm, where the intermediate quantities were total probability, here they consist of the maximum probability best_logp[t,k] for the sequence up to time t with final output category k for time t, along with a backpointer to the source of the link. Following the backpointers from the best final log probability for the final time t yields the optimal state sequence.

This inference can be run for the same unsupervised outputs u as are used to fit the semisupervised model. The above code can be found in the same model file

as the unsupervised fit. This is the Bayesian approach to inference, where the data being reasoned about is used in a semisupervised way to train the model. It is not "cheating" because the underlying states for u are never observed — they are just estimated along with all of the other parameters.

If the outputs u are not used for semisupervised estimation but simply as the basis for prediction, the result is equivalent to what is represented in the BUGS modeling language via the cut operation. That is, the model is fit independently of u, then those parameters used to find the most likely state to have generated u.

7. Missing Data & Partially Known Parameters

Bayesian inference supports a very general approach to missing data in which any missing data item is represented as a parameter that is estimated in the posterior (Gelman et al., 2013). If the missing data is not explicitly modeled, as in the predictors for most regression models, then the result is an improper prior on the parameter representing the missing predictor.

Mixing arrays of observed and missing data can be difficult to include in Stan, partly because it can be tricky to model discrete unknowns in Stan and partly because unlike some other statistical languages (for example, R and Bugs), Stan requires observed and unknown quantities to be defined in separate places in the model. Thus it can be necessary to include code in a Stan program to splice together observed and missing parts of a data structure. Examples are provided later in the chapter.

7.1. Missing Data

Stan treats variables declared in the `data` and `transformed data` blocks as known and the variables in the `parameters` block as unknown.

An example involving missing normal observations[1] could be coded as follows.

```
data {
  int<lower=0> N_obs;
  int<lower=0> N_mis;
  real y_obs[N_obs];
}
parameters {
  real mu;
  real<lower=0> sigma;
  real y_mis[N_mis];
}
model {
  for (n in 1:N_obs)
    y_obs[n] ~ normal(mu,sigma);
  for (n in 1:N_mis)
    y_mis[n] ~ normal(mu,sigma);
}
```

The number of observed and missing data points are coded as data with non-negative integer variables `N_obs` and `N_mis`. The observed data is provided as an array data

[1] A more meaningful estimation example would involve a regression of the observed and missing observations using predictors that were known for each and specified in the `data` block.

variable y_obs. The missing data is coded as an array parameter, y_mis. The ordinary parameters being estimated, the location mu and scale sigma, are also coded as parameters. A better way to write the model would be to vectorize, so the body would be

```
y_obs ~ normal(mu,sigma);
y_mis ~ normal(mu,sigma);
```

The model contains one loop over the observed data and one over the missing data. This slight redundancy in specification leads to much more efficient sampling for missing data problems in Stan than the more general technique described in the next section.

7.2. Partially Known Parameters

In some situations, such as when a multivariate probability function has partially observed outcomes or parameters, it will be necessary to create a vector mixing known (data) and unknown (parameter) values. This can be done in Stan by creating a vector or array in the transformed parameters block and assigning to it.

The following example involves a bivariate covariance matrix in which the variances are known, but the covariance is not.

```
data {
  int<lower=0> N;
  vector[2] y[N];
  real<lower=0> var1;     real<lower=0> var2;
}
transformed data {
  real<upper=0> min_cov;
  real<lower=0> max_cov;
  max_cov <- sqrt(var1 * var2);
  min_cov <- -max_cov;
}
parameters {
  vector[2] mu;
  real<lower=min_cov,upper=max_cov> cov;
}
transformed parameters {
  matrix[2,2] sigma;
  sigma[1,1] <- var1;     sigma[1,2] <- cov;
  sigma[2,1] <- cov;      sigma[2,2] <- var2;
}
model {
```

```
  for (n in 1:N)
    y[n] ~ multi_normal(mu,sigma);
}
```

The variances are defined as data in variables var1 and var2, whereas the covariance is defined as a parameter in variable cov. The 2×2 covariance matrix sigma is defined as a transformed parameter, with the variances assigned to the two diagonal elements and the covariance to the two off-diagonal elements.

The constraint on the covariance declaration ensures that the resulting covariance matrix sigma is positive definite. The bound, plus or minus the square root of the product of the variances, is defined as transformed data so that it is only calculated once.

7.3. Efficiency Note

The missing-data example in the first section could be programmed with a mixed data and parameter array following the approach of the partially known parameter example in the second section. The behavior will be correct, but the computation is wasteful. Each parameter, be it declared in the parameters or transformed parameters block, uses an algorithmic differentiation variable which is more expensive in terms of memory and gradient-calculation time than a simple data variable. Furthermore, the copy takes up extra space and extra time.

7.4. Loading matrix for factor analysis

Rick Farouni, on the Stan users group, inquired as to how to build a Cholesky factor for a covariance matrix with a unit diagonal, as used in Bayesian factor analysis Aguilar and West (2000). This can be accomplished by declaring the below-diagonal elements as parameters, then filling the full matrix as a transformed parameter.

```
data {
  int<lower=2> K;
}
transformed data {
  int<lower=1> K_choose_2;
  K_choose_2 <- (K * (K - 1)) / 2;
}
parameters {
  vector[K_choose_2] L_lower;
}
transformed parameters {
```

```
    cholesky_factor_cov[K] L;
    for (k in 1:K)
      L[k,k] <- 1;
    {
      int i;
      for (m in 2:K) {
        for (n in 1:(m - 1)) {
          L[m,n] <- L_lower[i];
          L[n,m] <- 0;
          i <- i + 1;
        }
      }
    }
  }
```

It is most convenient to place a prior directly on L_lower. An alternative would be a prior for the full Cholesky factor L, because the transform from L_lower to L is just the identity and thus does not require a Jacobian adjustment (despite the warning from the parser, which is not smart enough to do the code analysis to infer that the transform is linear). It would not be at all convenient to place a prior on the full covariance matrix L * L', because that would require a Jacobian adjustment; the exact adjustment is provided in the subsection of Section 53.1 devoted to covariance matrices.

8. Truncated or Censored Data

Data in which measurements have been truncated or censored can be coded in Stan following their respective probability models.

8.1. Truncated Distributions

Truncation in Stan is restricted to univariate distributions for which the corresponding log cumulative distribution function (cdf) and log complementary cumulative distribution (ccdf) functions are available. See the subsection on truncated distributions in Section 24.3 for more information on truncated distributions, cdfs, and ccdfs.

8.2. Truncated Data

Truncated data is data for which measurements are only reported if they fall above a lower bound, below an upper bound, or between a lower and upper bound.

Truncated data may be modeled in Stan using truncated distributions. For example, suppose the truncated data is y_n with an upper truncation point of $U = 300$ so that $y_n < 300$. In Stan, this data can be modeled as following a truncated normal distribution for the observations as follows.

```
data {
  int<lower=0> N;
  real U;
  real<upper=U> y[N];
}
parameters {
  real mu;
  real<lower=0> sigma;
}
model {
  for (n in 1:N)
    y[n] ~ normal(mu,sigma) T[,U];
}
```

The model declares an upper bound `U` as data and constrains the data for `y` to respect the constraint; this will be checked when the data is loaded into the model before sampling begins.

This model implicitly uses an improper flat prior on the scale and location parameters; these could be given priors in the model using sampling statements.

Constraints and Out-of-Bounds Returns

If the sampled variate in a truncated distribution lies outside of the truncation range, the probability is zero, so the log probability will evaluate to $-\infty$. For instance, if variate y is sampled with the statement.

```
for (n in 1:N)
  y[n] ~ normal(mu,sigma) T[L,U];
```

then if the value of y[n] is less than the value of L or greater than the value of U, the sampling statement produces a zero-probability estimate.

To avoid variables straying outside of truncation bounds, appropriate constraints are required. For example, if y is a parameter in the above model, the declaration should constrain it to fall between the values of L and U.

```
parameters {
  real<lower=L,upper=U> y[N];
  ...
```

If in the above model, L or U is a parameter and y is data, then L and U must be appropriately constrained so that all data is in range and the value of L is less than that of U (if they are equal, the parameter range collapses to a single point and the Hamiltonian dynamics used by the sampler break down). The following declarations ensure the bounds are well behaved.

```
parameters {
  real<upper=min(y)> L; // L < y[n]
  real<lower=fmax(L,max(y))> U; // L < U; y[n] < U
```

Note that for pairs of real numbers, the function fmax is used rather than max.

Unknown Truncation Points

If the truncation points are unknown, they may be estimated as parameters. This can be done with a slight rearrangement of the variable declarations from the model in the previous section with known truncation points.

```
data {
  int<lower=1> N;
  real y[N];
}
parameters {
  real<upper = min(y)> L;
  real<lower = max(y)> U;
  real mu;
```

```
  real<lower=0> sigma;
}
model {
  L ~ ...;
  U ~ ...;
  for (n in 1:N)
    y[n] ~ normal(mu,sigma) T[L,U];
}
```

Here there is a lower truncation point `L` which is declared to be less than or equal to the minimum value of `y`. The upper truncation point `U` is declared to be larger than the maximum value of `y`. This declaration, although dependent on the data, only enforces the constraint that the data fall within the truncation bounds. With `N` declared as type `int<lower=1>`, there must be at least one data point. The constraint that `L` is less than `U` is enforced indirectly, based on the non-empty data.

The ellipses where the priors for the bounds `L` and `U` should go should be filled in with a an informative prior in order for this model to not concentrate `L` strongly around `min(y)` and `U` strongly around `max(y)`.

8.3. Censored Data

Censoring hides values from points that are too large, too small, or both. Unlike with truncated data, the number of data points that were censored is known. The textbook example is the household scale which does not report values above 300 pounds.

Estimating Censored Values

One way to model censored data is to treat the censored data as missing data that is constrained to fall in the censored range of values. Since Stan does not allow unknown values in its arrays or matrices, the censored values must be represented explicitly, as in the following right-censored case.

```
data {
  int<lower=0> N_obs;
  int<lower=0> N_cens;
  real y_obs[N_obs];
  real<lower=max(y_obs)> U;
}
parameters {
  real<lower=U> y_cens[N_cens];
  real mu;
  real<lower=0> sigma;
```

```
}
model {
  y_obs ~ normal(mu,sigma);
  y_cens ~ normal(mu,sigma);
}
```

Because the censored data array y_cens is declared to be a parameter, it will be sampled along with the location and scale parameters mu and sigma. Because the censored data array y_cens is declared to have values of type real<lower=U>, all imputed values for censored data will be greater than U. The imputed censored data affects the location and scale parameters through the last sampling statement in the model.

Integrating out Censored Values

Although it is wrong to ignore the censored values in estimating location and scale, it is not necessary to impute values. Instead, the values can be integrated out. Each censored data point has a probability of

$$\Pr[y > U] = \int_U^\infty \mathsf{Normal}(y|\mu,\sigma)\,dy = 1 - \Phi\left(\frac{y-\mu}{\sigma}\right),$$

where $\Phi()$ is the unit normal cumulative distribution function. With M censored observations, the total probability on the log scale is

$$\log \prod_{m=1}^{M} \Pr[y_m > U] = \log\left(1 - \Phi\left(\frac{y-\mu}{\sigma}\right)\right)^M = M\,\texttt{normal\_ccdf\_log}(y,\mu,\sigma),$$

where normal_ccdf_log is the log of complementary CDF (Stan provides <distr>_ccdf_log for each distribution implemented in Stan).

The following right-censored model assumes that the censoring point is known, so it is declared as data.

```
data {
  int<lower=0> N_obs;
  int<lower=0> N_cens;
  real y_obs[N_obs];
  real<lower=max(y_obs)> U;
}
parameters {
  real mu;
  real<lower=0> sigma;
}
model {
```

```
  y_obs ~ normal(mu,sigma);
  increment_log_prob(N_cens * normal_ccdf_log(U,mu,sigma));
}
```

For the observed values in y_obs, the normal sampling model is used without truncation. The log probability is directly incremented using the calculated log cumulative normal probability of the censored data items.

For the left-censored data the CDF (normal_cdf_log) has to be used instead of complementary CDF. If the censoring point variable (L) is unknown, its declaration should be moved from the data to the parameters block.

```
data {
  int<lower=0> N_obs;
  int<lower=0> N_cens;
  real y_obs[N_obs];
}
parameters {
  real<upper=min(y_obs)> L;
  real mu;
  real<lower=0> sigma;
}
model {
  L ~ normal(mu,sigma);
  y_obs ~ normal(mu,sigma);
  increment_log_prob(N_cens * normal_cdf_log(L,mu,sigma));
}
```

9. Finite Mixtures

Finite mixture models of an outcome assume that the outcome is drawn from one of several distributions, the identity of which is controlled by a categorical mixing distribution. Mixture models typically have multimodal densities with modes near the modes of the mixture components. Mixture models may be parameterized in several ways, as described in the following sections.

9.1. Latent Discrete Parameterization

One way to parameterize a mixture model is with a latent categorical variable indicating which mixture component was responsible for the outcome. For example, consider K normal distributions with locations $\mu_k \in \mathbb{R}$ and scales $\sigma_k \in (0, \infty)$. Now consider mixing them in proportion θ, where $\theta_k \geq 0$ and $\sum_{k=1}^{K} \theta_k = 1$ (i.e., θ lies in the unit K-simplex). For each outcome y_n there is a latent variable z_n in $\{1, \ldots, K\}$ with a categorical distribution parameterized by θ,

$$z_n \sim \mathsf{Categorical}(\theta).$$

The variable y_n is distributed according to the parameters of the mixture component z_n,

$$y_n \sim \mathsf{Normal}(\mu_{z[n]}, \sigma_{z[n]}).$$

This model is not directly supported by Stan because it involves discrete parameters z_n, but Stan can sample μ and σ by summing out the z parameter as described in the next section.

9.2. Summing out the Responsibility Parameter

To implement the normal mixture model outlined in the previous section in Stan, the discrete parameters can be summed out of the model. If Y is a mixture of K normal distributions with locations μ_k and scales σ_k with mixing proportions θ in the unit K-simplex, then

$$p_Y(y\theta, \mu, \sigma) = \sum_{k=1}^{K} \theta_k \, \mathsf{Normal}(\mu_k, \sigma_k).$$

9.3. Log Sum of Exponentials: Linear Sums on the Log Scale

The log sum of exponentials function is used to define mixtures on the log scale. It is defined for two inputs by

$$\text{log\_sum\_exp}(a, b) = \log(\exp(a) + \exp(b)).$$

If a and b are probabilities on the log scale, then $\exp(a) + \exp(b)$ is their sum on the linear scale, and the outer log converts the result back to the log scale; to summarize, log_sum_exp does linear addition on the log scale. The reason to use Stan's built-in log_sum_exp function is that it can prevent underflow and overflow in the exponentiation, by calculating the result as

$$\log(\exp(a) + \exp(b)) = \max(a, b) + \log(\exp(a - \max(a, b)), \exp(b - \max(a, b))).$$

In this evaluation, one of the terms $a - \max(a, b)$ or $b - \max(a, b)$ is zero and the other is negative, thus eliminating the possibility of overflow or underflow in the leading term and eking the most arithmetic precision possible out of the operation.

For example, the mixture of $\mathsf{Normal}(-1, 2)$ and $\mathsf{Normal}(3, 1)$ with mixing proportion $\theta = (0.3, 0.7)^\top$ can be implemented in Stan as follows.

```
parameters {
  real y;
}
model {
  increment_log_prob(log_sum_exp(log(0.3)
                                   + normal_log(y,-1,2),
                                 log(0.7)
                                   + normal_log(y,3,1));
}
```

The log probabilty term is derived by taking

$$\begin{aligned}
\log p_Y(y|\theta, \mu, \sigma) &= \log(0.3 \times \mathsf{Normal}(y| -1, 2) \ + \ 0.7 \times \mathsf{Normal}(y|3, 1)\,) \\
&= \log(\exp(\log(0.3 \times \mathsf{Normal}(y| -1, 2))) \\
&\qquad + \exp(\log(0.7 \times \mathsf{Normal}(y|3, 1)))\,) \\
&= \mathrm{log_sum_exp}(\log(0.3) + \log \mathsf{Normal}(y| -1, 2), \\
&\qquad\qquad \log(0.7) + \log \mathsf{Normal}(y|3, 1)\,).
\end{aligned}$$

Estimating Parameters of a Mixture

Given the scheme for representing mixtures, it may be moved to an estimation setting, where the locations, scales, and mixture components are unknown. Further generalizing to a number of mixture components specified as data yields the following model.

```
data {
  int<lower=1> K;          // number of mixture components
  int<lower=1> N;          // number of data points
  real y[N];               // observations
```

```
}
parameters {
  simplex[K] theta;          // mixing proportions
  real mu[K];                // locations of mixture components
  real<lower=0> sigma[K];    // scales of mixture components
}
model {
  real ps[K];                // temp for log component densities
  sigma ~ cauchy(0,2.5);
  mu ~ normal(0,10);
  for (n in 1:N) {
    for (k in 1:K) {
      ps[k] <- log(theta[k])
               + normal_log(y[n],mu[k],sigma[k]);
    }
    increment_log_prob(log_sum_exp(ps));
  }
}
```

The model involves K mixture components and N data points. The mixing proportion parameter theta is declared to be a unit K-simplex, whereas the component location parameter mu and scale parameter sigma are both defined to be arrays of size K. The values in the scale array sigma are constrained to be non-negative, and have a weakly informative prior given in the model. The model declares a local array variable ps to be size K and uses it to accumulate the contributions from the mixture components.

The locations and scales are drawn from simple priors for the sake of this example, but could be anything supported by Stan. The mixture components could even be modeled hierarchically.

The main action is in the loop over data points n. For each such point, the log of $\theta_k \times \mathsf{Normal}(y_n|\mu_k, \sigma_k)$ is calculated and added to the array ps. Then the log probability is incremented with the log sum of exponentials of those values.

9.4. Zero-Inflated and Hurdle Models

Zero-inflated and hurdle models both provide mixtures of a Poisson and Bernoulli probabilty mass function to allow more flexiblity in modeling the probability of a zero outcome. Zero-inflated models, as defined by Lambert (1992), add additional probability mass to the outcome of zero. Hurdle models, on the other hand, are formulated as pure mixtures of zero and non-zero outcomes.

Zero Inflation

Consider the following example for zero-inflated Poisson distributions. It uses a parameter theta here there is a probability θ of drawing a zero, and a probability $1-\theta$ of drawing from $\mathsf{Poisson}(\lambda)$. The probability function is thus

$$p(y_n|\theta,\lambda) = \begin{cases} \theta + (1-\theta) \times \mathsf{Poisson}(0|\lambda) & \text{if } y_n = 0, \text{ and} \\ (1-\theta) \times \mathsf{Poisson}(y_n|\lambda) & \text{if } y_n > 0. \end{cases}$$

The log probability function can be implemented directly in Stan as follows.

```
data {
  int<lower=0> N;
  int<lower=0> y[N];
}
parameters {
  real<lower=0,upper=1> theta;
  real lambda;
}
model {
  for (n in 1:N) {
    if (y[n] == 0)
      increment_log_prob(log_sum_exp(bernoulli_log(1,theta),
                                     bernoulli_log(0,theta)
                                     + poisson_log(y[n],lambda)));
    else
      increment_log_prob(bernoulli_log(0,theta)
                         + poisson_log(y[n],lambda));
  }
}
```

The `log_sum_exp(lp1,lp2)` function adds the log probabilities on the linear scale; it is defined to be equal to `log(exp(lp1) + exp(lp2))`, but is more arithmetically stable and faster.

Although it might be tempting to try to use the `if_else` syntax within the `increment_log_prob` function, it is not recommended because `if_else(c,e1,e2)` evaluates both `e1` and `e2` no matter what the value of `c` is.

Hurdle Models

The hurdle model is similar to the zero-inflated model, but more flexible in that the zero outcomes can be deflated as well as inflated. The probability mass function for

the hurdle model is defined by

$$p(y|\theta,\lambda) = \begin{cases} \theta & \text{if } y = 0, \text{ and} \\ (1-\theta)\,\dfrac{\mathsf{Poisson}(y|\lambda)}{1-\mathsf{PoissonCDF}(0|\lambda)} & \text{if } y > 0, \end{cases}$$

where PoissonCDF is the cumulative distribution function for the Poisson distribution. The hurdle model is even more straightforward to program in Stan, as it does not require an explicit mixture.

```
y[n] ~ bernoulli(theta);
if (y[n] > 0)
  y[n] ~ poisson(lambda) T[1,];
```

The `[1,]` after the Poisson indicates that it is truncated below at 1; see Section 37.3. Although the variable `y[n]` is being "sampled" twice, the effect on the log probability fucntion follows the definition (on the log scale).

10. Measurement Error and Meta-Analysis

Most quantities used in statistical models arise from measurements. Most of these measurements are taken with some error. When the measurement error is small relative to the quantity being measured, its effect on a model are usually small. When measurement error is large relative to the quantity being measured, or when very precise relations can be estimated being measured quantities, it is useful to introduce an explicit model of measurement error.

10.1. Bayesian Measurement Error Model

A Bayesian approach to measurement error can be formulated directly by treating the true quantities being measured as missing data (Clayton, 1992; Richardson and Gilks, 1993). This requires a model of how the measurements are derived from the true values.

Regression with Measurement Error

Before considering regression with measurement error, first consider a linear regression model where the observed data for N cases includes a predictor x_n and outcome y_n. In Stan, a linear regression for y based on x with a slope and intercept is modeled as follows.

```
data {
  int<lower=0> N;        // number of cases
  real x[N];             // predictor (covariate)
  real y[N];             // outcome (variate)
}
parameters {
  real alpha;           // intercept
  real beta;            // slope
  real<lower=0> sigma;  // outcome noise
}
model {
  y ~ normal(alpha + beta * x, sigma);
  alpha ~ normal(0,10);
  beta ~ normal(0,10);
  sigma ~ cauchy(0,5);
}
```

Now suppose that the true values of the predictors x_n are not known, but for each n, a measurement x_n^{meas} of x_n is available. If the error in measurement can be

modeled, the measured value x_n^{meas} can be modeled in terms of the true value x_n plus measurement noise. The true value x_n is treated as missing data and estimated along with other quantities in the model. A very simple approach is to assume the measurement error is normal with known deviation τ. This leads to the following regression model with constant measurement error.

```
data {
  ...
  real x_meas[N];     // measurement of x
  real<lower=0> tau;  // measurement noise
}
parameters {
  real x[N];          // unknown true value
  real mu_x;          // prior location
  real sigma_x        // prior scale
  ...
}
model {
  x ~ normal(mu_x, sigma_x);  // prior
  x_meas ~ normal(x, tau);    // measurement model
  y ~ normal(alpha + beta * x, sigma);
  ...
}
```

The regression coefficients `alpha` and `beta` and regression noise scale `sigma` are the same as before, but now `x` is declared as a parameter rather than as data. The data is now `x_meas`, which is a measurement of the true `x` value with noise scale `tau`. The model then specifies that the measurement error for `x_meas[n]` given true value `x[n]` is normal with deviation `tau`. Furthermore, the true values `x` are given a hierarchical prior; without

In cases where the measurement errors are not normal, richer measurement error models may be specified. The prior on the true values may also be enriched. For instance, (Clayton, 1992) introduces an exposure model for the unknown (but noisily measured) risk factors x in terms of known (without measurement error) risk factors c. A simple model would regress x_n on the covariates c_n with noise term υ,

$$x_n \sim \mathsf{Normal}(\gamma^\top c, \upsilon).$$

This can be coded in Stan just like any other regression. And, of course, other exposure models can be provided.

10.2. Meta-Analysis

Meta-analysis aims to pool the data from several studies, such as the application of a tutoring program in several schools or treatment using a drug in several clinical trials.

The Bayesian framework is particularly convenient for meta-analysis, because each previous study can be treated as providing a noisy measurement of some underlying quantity of interest. The model then follows directly from two components, a prior on the underlying quantities of interest and a measurement-error style model for each of the studies being analyzed.

Treatment Effects in Controlled Studies

Suppose the data in question arise from a total of M studies providing paired binomial data for a treatment and control group. For instance, the data might be post-surgical pain reduction under a treatment of ibuprofen (Warn et al., 2002) or mortality after myocardial infarction under a treatment of beta blockers (Gelman et al., 2013, Section 5.6).

Data

The clinical data consists of J trials, each with n^t treatment cases, n^c control cases, r^t successful outcomes among those treated and r^c successful outcomes among those in the control group. This data can be declared in Stan as follows.[1]

```
data {
  int<lower=0> J;
  int<lower=0> n_t[J];  // num cases, treatment
  int<lower=0> r_t[J];  // num successes, treatment
  int<lower=0> n_c[J];  // num cases, control
  int<lower=0> r_c[J];  // num successes, control
}
```

Converting to Log Odds and Standard Error

Although the clinical trial data is binomial in its raw format, it may be transformed to an unbounded scale by considering the log odds ratio

$$y_j = \log\left(\frac{r_j^t/(n_j^t - r_j^t)}{r_j^c/(n_j^c - r_j^c)}\right) \quad = \quad \log\left(\frac{r_j^t}{n_j^t - r_j^t}\right) - \log\left(\frac{r_j^c}{n_j^c - r_j^c}\right)$$

[1]Stan's integer constraints are not powerful enough to express the constraint that `r_t[j]` ≤ `n_t[j]`, but this constraint could be checked in the transformed data block.

and corresponding standard errors

$$\sigma_j = \sqrt{\frac{1}{r_i^T} + \frac{1}{n_i^T - r_i^T} + \frac{1}{r_i^C} + \frac{1}{n_i^C - r_i^C}}.$$

The log odds and standard errors can be defined in a transformed parameter block, though care must be taken not to use integer division (see Section 30.1).

```
transformed data {
  real y[J];
  real<lower=0> sigma[J];
  for (j in 1:J)
    y[j] <- log(r_t[j]) - log(n_t[j] - r_t[j])
            - (log(r_c[j]) - log(n_c[j] - r_c[j]);
  for (j in 1:J)
    sigma[j] <- sqrt(1.0/r_t[i] + 1.0/(n_t[i] - r_t[i])
                     + 1.0/r_c[i] + 1.0/(n_c[i] - r_c[i]));
}
```

This definition will be problematic if any of the success counts is zero or equal to the number of trials. If that arises, a direct binomial model will be required or other transforms must be used than the unregularized sample log odds.

Non-Hierarchical Model

With the transformed data in hand, two standard forms of meta-analysis can be applied. The first is a so-called "fixed effects" model, which assumes a single parameter for the global odds ratio. This model is coded in Stan as follows.

```
parameters {
  real theta;  // global treatment effect, log odds
}
model {
  y ~ normal(theta,sigma);
}
```

The sampling statement for y is vectorized; it has the same effect as the following.

```
  for (j in 1:J)
    y[j] ~ normal(theta,sigma[j]);
```

It is common to include a prior for theta in this model, but it is not strictly necessary for the model to be proper because y is fixed and $\mathsf{Normal}(y|\mu,\sigma) = \mathsf{Normal}(\mu|y,\sigma)$.

Hierarchical Model

To model so-called "random effects," where the treatment effect may vary by clinical trial, a hierarchical model can be used. The parameters include per-trial treatment effects and the hierarchical prior parameters, which will be estimated along with other unknown quantities.

```
parameters {
  real theta[J];      // per-trial treatment effect
  real mu;            // mean treatment effect
  real<lower=0> tau;  // deviation of treatment effects
}
model {
  y ~ normal(theta,sigma);
  theta ~ normal(mu,tau);
  mu ~ normal(0,10);
  tau ~ cauchy(0,5);
}
```

Although the vectorized sampling statement for `y` appears unchanged, the parameter `theta` is now a vector. The sampling statement for `theta` is also vectorized, with the hyperparameters `mu` and `tau` themselves being given wide priors compared to the scale of the data.

Rubin (1981) provided a hierarchical Bayesian meta-analysis of the treatment effect of Scholastic Aptitude Test (SAT) coaching in eight schools based on the sample treatment effect and standard error in each school. The model provided for this data in (Gelman et al., 2013, Section 5.5) is included with the data in the Stan distribution in directory `example-models/misc/eight-schools/`.

Extensions and Alternatives

Smith et al. (1995) and Gelman et al. (2013, Section 19.4) provide meta-analyses based directly on binomial data. Warn et al. (2002) consider the modeling implications of using alternatives to the log-odds ratio in transforming the binomial data.

If trial-specific predictors are available, these can be included directly in a regression model for the per-trial treatment effects θ_j.

11. Latent Discrete Parameters

Stan does not support sampling discrete parameters. So it is not possible to directly translate BUGS or JAGS models with discrete parameters (i.e., discrete stochastic nodes). Nevertheless, it is possible to code many models that involve bounded discrete parameters by marginalizing out the discrete parameters.[1] This chapter shows how to code several widely-used models involving latent discrete parameters. The next chapter, Chapter 12, on clustering models, considers further models involving latent discrete parameters.

11.1. The Benefits of Marginalization

Although it requires some algebra on the joint probability function, a pleasant byproduct of the required calculations is the posterior expectation of the marginalized variable, which is often the quantity of interest for a model. This allows far greater exploration of the tails of the distribution as well as more efficient sampling on an iteration-by-iteration basis because the expectation at all possible values is being used rather than itself being estimated through sampling a discrete parameter.

Standard optimization algorithms, including expectation maximization (EM), are often provided in applied statistics papers to describe maximum likelihood estimation algorithms. Such derivations provide exactly the marginalization needed for coding the model in Stan.

11.2. Change Point Models

The first example is a model of coal mining disasters in the the U.K. for the years 1851–1962.[2]

Model with Latent Discrete Parameter

(Fonnesbeck et al., 2013, Section 3.1) provide a Poisson model of disaster rate D_t in year t with two rate parameters, an early rate (e) and late rate (l), that change at a

[1] The computations are similar to those involved in expectation maximization (EM) algorithms (Dempster et al., 1977).

[2] The original source of the data is (Jarrett, 1979), which itself is a note correcting an earlier data collection.

given point in time s. The full model expressed using a latent discrete parameter s is

$$\begin{array}{rcl} e & \sim & \mathsf{Exponential}(r_e) \\ l & \sim & \mathsf{Exponential}(r_l) \\ s & \sim & \mathsf{Uniform}(1, T) \\ D_t & \sim & \mathsf{Poisson}(t < s \; ? \; e \; : \; l) \end{array}$$

The last line uses the conditional operator (also known as the ternary operator), which is borrowed from C and related languages. The conditional operator has the same behavior as the `ifelse` function in R, but uses a more compact notation involving separating its three arguments by a question mark (?) and colon (:). The conditional operator is defined by

$$c \; ? \; x_1 \; : \; x_2 = \begin{cases} x_1 & \text{if } c \text{ is true (i.e., non-zero), and} \\ x_2 & \text{if } c \text{ is false (i.e., zero).} \end{cases}$$

Although Stan itself does not currently support the conditional operator, it will in the future.

Marginalizing out the Discrete Parameter

To code this model in Stan, the discrete parameter s must be marginalized out to produce a model defining the log of the probability function $p(e, l, D_t)$. The full joint probability factors as

$$\begin{array}{rcl} p(e, l, s, D) & = & p(e)\, p(l)\, p(s)\, p(D|s, e, l) \\ & = & \mathsf{Exponential}(e|r_e)\; \mathsf{Exponential}(l|r_l)\; \mathsf{Uniform}(s|1, T) \\ & & \prod_{t=1}^{T} \mathsf{Poisson}(D_t|t < s \; ? \; e \; : \; l), \end{array}$$

To marginalize, an alternative factorization into prior and likelihood is used,

$$p(e, l, D) \quad = \quad p(e, l)\, p(D|e, l),$$

where the likelihood is defined by marginalizing s as

$$\begin{array}{rcl} p(D|e, l) & = & \sum_{s=1}^{T} p(s, D|e, l) \\ & = & \sum_{s=1}^{T} p(s) p(D|s, e, l) \\ & = & \sum_{s=1}^{T} \mathsf{Uniform}(s|1, T) \prod_{t=1}^{T} \mathsf{Poisson}(D_t|t < s \; ? \; e \; : \; l) \end{array}$$

Stan operates on the log scale and thus requires the log likelihood,

$$\begin{array}{rcl}\log p(D|e,l) & = & \text{log\_sum\_exp}_{s=1}^{T} \log \mathsf{Uniform}(s|1,T) \\ & & + \sum_{t=1}^{T} \log \mathsf{Poisson}(D_t|t < s\ ?\ e\ :\ l),\end{array}$$

where the log sum of exponents function is defined by

$$\text{log\_sum\_exp}_{n=1}^{N} \alpha_n = \log \sum_{n=1}^{N} \exp(\alpha_n).$$

The log sum of exponents function allows the model to be coded directly in Stan using the built-in function `log_sum_exp`, which provides both arithmetic stability and efficiency for mixture model calculations.

Coding the Model in Stan

The Stan program for the change point model is shown in Figure 11.1. The transformed parameter `lp[s]` stores the quantity $\log p(s, D|e, l)$.

Fitting the Model with MCMC

This model is easy to fit using MCMC with NUTS in its default configuration. Convergence is very fast and sampling produces roughly one effective sample every two iterations. Because it is a relatively small model (the inner double loop over time is roughly 20,000 steps), it is very fast.

The value of `lp` for each iteration for each change point is available because it is declared as a transformed parameter. If the value of `lp` were not of interest, it could be coded as a local variable in the model block and thus avoid the I/O overhead of saving values every iteration.

Posterior Distribution of the Discrete Change Point

The value of `lp[s]` in a given iteration is given by $\log p(s, D|e, l)$ for the values of the early and late rates, e and l, in the iteration. In each iteration after convergence, the early and late disaster rates, e and l, are drawn from the posterior $p(e, l|D)$ by MCMC sampling and the associated `lp` calculated. The value of `lp` may be normalized to calculate $p(s|e, l, D)$ in each iteration, based on on the current values of e and l. Averaging over iterations provides an unnormalized probability estimate of the change point being s,

$$\begin{array}{rcl}p(s|D) & \propto & q(s|D) \\ & = & \dfrac{1}{M} \displaystyle\sum_{m=1}^{M} \exp(\mathtt{lp}[m, s]).\end{array}$$

```
data {
  real<lower=0> r_e;
  real<lower=0> r_l;

  int<lower=1> T;
  int<lower=0> D[T];
}
transformed data {
  real log_unif;
  log_unif <- -log(T);
}
parameters {
  real<lower=0> e;
  real<lower=0> l;
}
transformed parameters {
  vector[T] lp;
  lp <- rep_vector(log_unif, T);
  for (s in 1:T)
    for (t in 1:T)
      lp[s] <- lp[s] + poisson_log(D[t], if_else(t < s, e, l));
}
model {
  e ~ exponential(r_e);
  l ~ exponential(r_l);
  increment_log_prob(log_sum_exp(lp));
}
```

Figure 11.1: *A change point model in which disaster rates* `D[t]` *have one rate,* `e`*, before the change point and a different rate,* `l`*, after the change point. The change point itself,* `s`*, is marginalized out as described in the text.*

where `lp`$[m, s]$ represents the value of `lp` in posterior draw m for change point s. By averaging over draws, e and l are themselves marginalized out, and the result has no dependence on a given iteration's value for e and l. A final normalization then produces the quantity of interest, the posterior probability of the change point being s conditioned on the data D,

$$p(s|D) = \frac{q(s|D)}{\sum_{s'=1}^{T} q(s'|D)}.$$

A plot of the values of $\log p(s|D)$ computed using Stan 2.4's default MCMC implementation is shown in Figure 11.2.

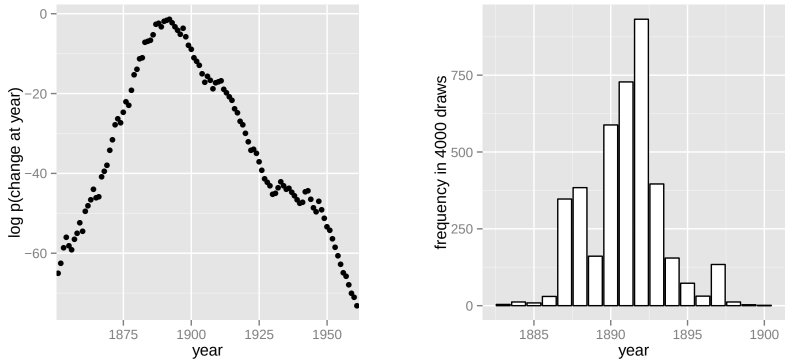

Figure 11.2: *The posterior estimates for the change point.* Left) *log probability of change point being in year, calculated analytically using* `lp`*;* Right) *frequency of change point draws in the posterior generated using* `lp`*. The plot on the left is on the log scale and the plot on the right on the linear scale; note the narrower range of years in the right-hand plot resulting from sampling. The posterior mean of* s *is roughly 1891.*

Discrete Sampling

Although not computationally advantageous compared to working in expectation using `lp`, it is possible to use Stan to sample the value of `s` at every iteration.

```
generated quantities {
  int<lower=1,upper=T> s;
  s <- categorical_rng(softmax(lp));
}
```

A posterior histogram of draws for s is shown on the right side of Figure 11.2.

Posterior Covariance

The discrete sample generated for s can be used to calculate covariance with other parameters. Although the sampling approach is straightforward, it is more statistically efficient (in the sense of requiring far fewer iterations for the same degree of accuracy) to calculate these covariances in expectation using `lp`.

11.3. Mark-Recapture Models

A widely applied field method in ecology is to capture (or sight) animals, mark them (e.g., by tagging), then release them. This process is then repeated one or more times,

and is often done for populations on an ongoing basis. The resulting data may be used to estimate population size.

The first subsection describes a very simple mark-recapture model that does not involve any latent discrete parameters. The following subsections describes the Cormack-Jolly-Seber model, which involves latent discrete parameters for animal death.

Simple Mark-Recapture Model

In the simplest case, a one-stage mark-recapture study produces the following data

- M : number of animals marked in first capture,
- C : number animals in second capture, and
- R : number of marked animals in second capture.

The estimand of interest is

- N : number of animals in the population.

Despite the notation, the model will take N to be a continuous parameter; just because the population must be finite doesn't mean the parameter representing it must be. The parameter will be used to produce a real-valued estimate of the population size.

The Lincoln-Petersen (Lincoln, 1930; Petersen, 1896) method for estimating population size is

$$\hat{N} = \frac{MC}{R}.$$

This population estimate would arise from a probabilistic model in which the number of recaptured animals is distributed binomially,

$$R \sim \mathsf{Binomial}(C, M/N)$$

given the total number of animals captured in the second round (C) with a recapture probability of M/N, the fraction of the total population N marked in the first round.

The probabilistic variant of the Lincoln-Petersen estimator can be directly coded in Stan as shown in Figure 11.3. The Lincoln-Petersen estimate is the maximum likelihood estimate (MLE) for this model.

To enusre the MLE is the Lincoln-Petersen estimate, an improper uniform prior for N is used; this could (and should) be replaced with a more informative prior if possible based on knowledge of the population under study.

The one tricky part of the model is the lower bound $C - R + M$ placed on the population size N. Values below this bound are impossible because it is otherwise not possible to draw R samples out of the C animals recaptured. Implementing this lower

```
data {
  int<lower=0> M;
  int<lower=0> C;
  int<lower=0,upper=min(M,C)> R;
}
parameters {
  real<lower=(C - R + M)> N;
}
model {
  R ~ binomial(C, M / N);
}
```

Figure 11.3: *A probabilistic formulation of the Lincoln-Petersen population estimator for population size based on data from a one-step mark-recapture study. The lower bound on N is necessary to efficiently eliminate impossible values.*

bound is necessary to ensure sampling and optimization can be carried out in an unconstrained manner with unbounded support for parameters on the transformed (unconstrained) space. The lower bound in the declaration for C implies a variable transform $f : (C - R + M, \infty) \to (-\infty, +\infty)$ defined by $f(N) = \log(N - (C - R + M))$; see Section 53.2 for more information on the transform used for variables declared with a lower bound.

Cormack-Jolly-Seber with Discrete Parameter

The Cormack-Jolly-Seber (CJS) model (Cormack, 1964; Jolly, 1965; Seber, 1965) is an open-population model in which the population may change over time due to death; the presentation here draws heavily on (Schofield, 2007).

The basic data is

- I : number of individuals,
- T : number of capture periods, and
- $y_{i,t}$: boolean indicating if individual i was captured at time t.

Each individual is assumed to have been captured at least once because an individual only contributes information conditionally after they have been captured the first time.

There are two Bernoulli parameters in the model,

- ϕ_t : probability that animal alive at time t survives until $t + 1$ and
- p_t : probability that animal alive at time t is captured at time t.

These parameters will both be given uniform priors, but information should be used to tighten these priors in practice.

The CJS model also employs a latent discrete parameter $z_{i,t}$ indicating for each individual i whether it is alive at time t, distributed as

$$z_{i,t} \sim \mathsf{Bernoulli}(z_{i,t-1} \; ? \; 0 \; : \; \phi_{t-1}).$$

The conditional prevents the model positing zombies; once an animal is dead, it stays dead. The data distribution is then simple to express conditional on z as

$$y_{i,t} \sim \mathsf{Bernoulli}(z_{i,t} \; ? \; 0 \; : \; p_t)$$

The conditional enforces the constraint that dead animals cannot be captured.

Collective Cormack-Jolly-Seber Model

This subsection presents an implementation of the model in terms of counts for different history profiles for individuals over three capture times. It assumes exchangeability of the animals in that each is assigned the same capture and survival probabilities.

In order to ease the marginalization of the latent discrete parameter $z_{i,t}$, the Stan models rely on a derived quantity χ_t for the probability that an individual is never captured again if it is alive at time t (if it is dead, the recapture probability is zero). this quantity is defined recursively by

$$\chi_t = \begin{cases} 1 & \text{if } t = T \\ (1 - \phi_t) + \phi_t (1 - p_{t+1}) \chi_{t+1} & \text{if } t < T \end{cases}$$

The base case arises because if an animal was captured in the last time period, the probability it is never captured again is 1 because there are no more capture periods. The recursive case defining χ_t in terms of χ_{t+1} involves two possibiliites: (1) not surviving to the next time period, with probability $(1 - \phi_t)$, or (2) suriviving to the next time period with probability ϕ_t, not being captured in the next time period with probabilty $(1 - p_{t+1})$, and not being captured again after being alive in period $t + 1$ with probability χ_{t+1}.

With three capture times, there are three captured/not-captured profiles an individual may have. These may be naturally coded as binary numbers as follows.

profile	*captures* 1	2	3	*probability*
0	-	-	-	n/a
1	-	-	+	n/a
2	-	+	-	χ_2
3	-	+	+	$\phi_2 \, \phi_3$
4	+	-	-	χ_1
5	+	-	+	$\phi_1 \, (1 - p_2) \, \phi_2 \, p_3$
6	+	+	-	$\phi_1 \, p_2 \, \chi_2$
7	+	+	+	$\phi_1 \, p_2 \, \phi_2 \, p_3$

History 0, for animals that are never captured, is unobservable because only animals that are captured are observed. History 1, for animals that are only captured in the last round, provides no information for the CJS model, because capture/non-capture status is only informative when conditioned on earlier captures. For the remaining cases, the contribution to the likelihood is provided in the final column.

By defining these probabilities in terms of χ directly, there is no need for a latent binary parameter indicating whether an animal is alive at time t or not. The definition of χ is typically used to define the likelihood (i.e., marginalize out the latent discrete parameter) for the CJS model (Schofield, 2007, page 9).

The Stan model defines χ as a transformed parameter based on parameters ϕ and p. In the model block, the log probability is incremented for each history based on its count. This second step is similar to collecting Bernoulli observations into a binomial or categorical observations into a multinomial, only it is coded directly in the Stan program using `increment_log_prob` rather than being part of a built-in probability function.

Identifiability

The parameters ϕ_2 and p_3, the probability of death at time 2 and probabilty of capture at time 3 are not identifiable, because both may be used to account for lack of capture at time 3. Their product, $\beta_3 = \phi_2 \, p_3$, is identified. The Stan model defines `beta3` as a generated quantity. Unidentified parameters pose a problem for Stan's samplers' adaptation. Although the problem posed for adaptation is mild here because the parameters are bounded and thus have proper uniform priors, it would be better to formulate an identified parameterization. One way to do this would be to formulate a hierarchical model for the p and ϕ parameters.

Individual Cormack-Jolly-Seber Model

This section presents a version of the Cormack-Jolly-Seber (CJS) model cast at the individual level rather than collectively as in the previous subsection. It also extends

```
data {
  int<lower=0> history[7];
}
parameters {
  real<lower=0,upper=1> phi[2];
  real<lower=0,upper=1> p[3];
}
transformed parameters {
  real<lower=0,upper=1> chi[2];
  chi[2] <- (1 - phi[2]) + phi[2] * (1 - p[3]);
  chi[1] <- (1 - phi[1]) + phi[1] * (1 - p[2]) * chi[2];
}
model {
  increment_log_prob(history[2] * log(chi[2]));
  increment_log_prob(history[3] * (log(phi[2]) + log(p[3])));
  increment_log_prob(history[4] * (log(chi[1])));
  increment_log_prob(history[5]
                     * (log(phi[1]) + log1m(p[2])
                        + log(phi[2]) + log(p[3])));
  increment_log_prob(history[6]
                     * (log(phi[1]) + log(p[2])
                        + log(chi[2])));
  increment_log_prob(history[7]
                     * (log(phi[1]) + log(p[2])
                        + log(phi[2]) + log(p[3])));
}
generated quantities {
  real<lower=0,upper=1> beta3;
  beta3 <- phi[2] * p[3];
}
```

Figure 11.4: *A Stan program for the Cormack-Jolly-Seber mark-recapture model that considers counts of individuals with observation histories of being observed or not in three capture periods.*

the model to allow an arbitrary number of time periods. The data will consist of the number T of capture events, the number I of individuals, and a boolean flag $y_{i,t}$ indicating if individual i was observed at time t. In Stan,

```
data {
  int<lower=2> T;
  int<lower=0> I;
  int<lower=0,upper=1> y[I,T];
}
```

The advantages to the individual-level model is that it becomes possible to add individual "random effects" that affect survival or capture probability, as well as to avoid the combinatorics involved in unfolding 2^T observation histories for T capture times.

Utility Functions

The individual CJS model is written involves several function definitions. The first two are used in the transformed data block to compute the first and last time period in which an animal was captured.[3]

```
functions {
  int first_capture(int[] y_i) {
    for (k in 1:size(y_i))
      if (y_i[k])
        return k;
    return 0;
  }
  int last_capture(int[] y_i) {
    for (k_rev in 0:(size(y_i) - 1)) {
      int k;
      k <- size(y_i) - k_rev;
      if (y_i[k])
        return k;
    }
    return 0;
  }
  ...
}
```

These two functions are used to define the first and last capture time for each individual in the transformed data block.[4]

```
transformed data {
  int<lower=0,upper=T> first[I];
```

[3] An alternative would be to compute this on the outside and feed it into the Stan model as preprocessed data. Yet another alternative encoding would be a sparse one recording only the capture events along with their time and identifying the individual captured.

[4] Both functions return 0 if the individual represented by the input array was never captured. Individuals with no captures are not relevant for estimating the model because all probability statements are conditional on earlier captures. Typically they would be removed from the data, but the program allows them to be included even though they make not contribution to the log probability function.

```
  int<lower=0,upper=T> last[I];
  vector<lower=0,upper=I>[T] n_captured;
  for (i in 1:I)
    first[i] <- first_capture(y[i]);
  for (i in 1:I)
    last[i] <- last_capture(y[i]);
  n_captured <- rep_vector(0,T);
  for (t in 1:T)
    for (i in 1:I)
      if (y[i,t])
        n_captured[t] <- n_captured[t] + 1;
}
```

The transformed data block also defines `n_captured[t]`, which is the total number of captures at time `t`. The variable `n_captured` is defined as a vector instead of an integer array so that it can be used in an elementwise vector operation in the generated quantities block to model the population estimates at each time point.

The parameters and transformed parameters are as before, but now there is a function definition for computing the entire vector `chi`, the probability that if an individual is alive at `t` that it will never be captured again.

```
parameters {
  vector<lower=0,upper=1>[T-1] phi;
  vector<lower=0,upper=1>[T] p;
}
transformed parameters {
  vector<lower=0,upper=1>[T] chi;
  chi <- prob_uncaptured(T,p,phi);
}
```

The definition of `prob_uncaptured`, from the functions block, is

```
funtions {
  ...
  vector prob_uncaptured(int T, vector p, vector phi) {
    vector[T] chi;
    chi[T] <- 1.0;
    for (t in 1:(T - 1)) {
      int t_curr;
      int t_next;
      t_curr <- T - t;
      t_next <- t_curr + 1;
```

```
        chi[t_curr] <- (1 - phi[t_curr])
                       + phi[t_curr]
                         * (1 - p[t_next])
                         * chi[t_next];
      }
      return chi;
    }
  }
```

The function definition directly follows the mathematical definition of χ_t, unrolling the recursion into an iteration and defining the elements of chi from T down to 1.

The Model

Given the precomputed quantities, the model block directly encodes the CJS model's log likelihood function. All parameters are left with their default uniform priors and the model simply encodes the log probability of the observations q given the parameters p and phi as well as the transformed parameter chi defined in terms of p and phi.

```
  model {
    for (i in 1:I) {
      if (first[i] > 0) {
        for (t in (first[i]+1):last[i]) {
          1 ~ bernoulli(phi[t-1]);
          y[i,t] ~ bernoulli(p[t]);
        }
        1 ~ bernoulli(chi[last[i]]);
      }
    }
  }
```

The outer loop is over individuals, conditional skipping individuals i which are never captured. The never-captured check depends on the convention of the first-capture and last-capture functions returning 0 for first if an individual is never captured.

The inner loop for individual i first increments the log probability based on the survival of the individual with proability phi[t-1]. The outcome of 1 is fixed because the individual must survive between the first and last capture (i.e., no zombies). Note that the loop starts after the first capture, because all information in the CJS model is conditional on the first capture.

In the inner loop, the observed capture status y[i,t] for individual i at time t has a Bernoulli distribution based on the capture probabilty p[t] at time t.

After the inner loop, the probability of an animal never being seen again after being observed at time `last[i]` is included, because `last[i]` was defined to be the last time period in which animal `i` was observed.

Identified Parameters

As with the collective model described in the previous subsection, this model does not identify `phi[T-1]` and `p[T]`, but does identify their product, `beta`. Thus `beta` is defined as a generated quantity to monitor convergence and report.

```
generated quantities {
  real beta;
  ...

  beta <- phi[T-1] * p[T];
  ...
}
```

The parameter `p[1]` is also not modeled and will just be uniform between 0 and 1. A more finely articulated model might have a hierarchical or time-series component, in which case `p[1]` would be an unknown intial condition and both `phi[T-1]` and `p[T]` could be identified.

Population Size Estimates

The generated quantities also calculates an estimate of the population mean at each time `t` in the same way as in the simple mark-recapture model as the number of individuals captured at time `t` divided by the probability of capture at time `t`. This is done with the elementwise division operation for vectors (`./`) in the generated quantities block.

```
generated quantities {
  ...
  vector<lower=0>[T] pop;
  ...
  pop <- n_captured ./ p;
  pop[1] <- -1;
}
```

Generalizing to Individual Effects

All individuals are modeled as having the same capture probability, but this model could be easily generalized to use a logistic regression here based on individual-level

inputs to be used as predictors.

11.4. Data Coding and Diagnostic Accuracy Models

Although seemingly disparate tasks, the rating/coding/annotation of items with categories and diagnostic testing for disease or other conditions share several characteristics which allow their statistical properties to modeled similarly.

Diagnostic Accuracy

Suppose you have diagnostic tests for a condition of varying sensitivity and specificity. Sensitivity is the probability a test returns positive when the patient has the condition and specificity is the probability that a test returns negative when the patient does not have the condition. For example, mammograms and puncture biopsy tests both test for the presence of breast cancer. Mammograms have high sensitivity and low specificity, meaning lots of false positives, whereas puncture biopsies are the opposite, with low sensitivity and high specificity, meaning lots of false negatives.

There are several estimands of interest in such studies. An epidemiological study may be interested in the prevalence of a kind of infection, such as malaria, in a population. A test development study might be interested in the diagnostic accuracy of a new test. A health care worker performing tests might be interested in the disease status of a particular patient.

Data Coding

Humans are often given the task of coding (equivalently rating or annotating) data. For example, journal or grant reviewers rate submissions, a political study may code campaign commercials as to whether they are attack ads or not, a natural language processing study might annotate Tweets as to whehter they are positive or negative in overall sentiment, or a dentist looking at an X-ray classifies a patient as having a cavity or not. In all of these cases, the data coders play the role of the diagnostic tests and all of the same estimands are in play — data coder accuracy and bias, true categories of items being coded, or the prevalence of various categories of items in the data.

Noisy Categorical Measurement Model

In this section, only categorical ratings are considered, and the challenge in the modeling for Stan is to marginalize out the discrete parameters.

Dawid and Skene (1979) introduce a noisy-measurement model for data coding and apply in the epidemiological setting of coding what doctor notes say about patient histories; the same model can be used for diagnostic procedures.

Data

The data for the model consists of J raters (diagnostic tests), I items (patients), and K categories (condition statuses) to annotate, with $y_{i,j} \in 1{:}K$ being the rating provided by rater j for item i. In a diagnostic test setting for a particular condition, the raters are diagnostic procedures and often $K = 2$, with values signaling the presense or absence of the condition.[5]

It is relatively straightforward to extend Dawid and Skene's model to deal with the situation where not every rater rates each item exactly once.

Model Parameters

The model is based on three parameters, the first of which is discrete:

- z_i : a value in $1{:}K$ indicating the true category of item i,
- π : a K-simplex for the prevalence of the K categories in the population, and
- $\theta_{j,k}$: a K-simplex for the response of annotator j to an item of true category k.

Noisy Measurement Model

The true category of an item is assumed to be generated by a simple categorical distribution based on item prevalence,

$$z_i \sim \mathsf{Categorical}(\pi).$$

The rating $y_{i,j}$ provided for item i by rater j is modeled as a categorical response of rater i to an item of category z_i,[6]

$$y_{i,j} \sim \mathsf{Categorical}(\theta_{j,\pi_{z[i]}}).$$

[5]Diagnostic procedures are often ordinal, as in stages of cancer in oncological diagnosis or the severity of a cavity in dental diagnosis. Dawid and Skene's model may be used as is or naturally generalized for ordinal ratings using a latent continuous rating and cutpoints as in ordinal logistic regression.

[6]In the subscript, $z[i]$ is written as z_i to improve legibility.

Priors and Hierarchical Modeling

Dawid and Skene provided maximum likelihood estimates for θ and π, which allows them to generate probabilty estimates for each z_i.

To mimic Dawid and Skene's maximum likelihood model, the parameters $\theta_{j,k}$ and π can be given uniform priors over K-simplexes. It is straightforward to generalize to Dirichlet priors,

$$\pi \sim \mathsf{Dirichlet}(\alpha)$$

and

$$\theta_{j,k} \sim \mathsf{Dirichlet}(\beta_k)$$

with fixed hyperparameters α and β. The prior for $\theta_{j,k}$ must be allowed to vary in k, so that, for instance, $\beta_{k,k}$ is large enough to allow the prior to favor better-than-chance annotators over random or adversarial ones.

Because there are J coders, it would be natural to extend the model to include a hierarchical prior for β and to partially pool the estimates of coder accuracy and bias.

Marginalizing out the True Category

Because the true category parameter z is discrete, it must be marginalized out of the joint posterior in order to carry out sampling or maximum likelihood estimation in Stan. The joint posterior factors as

$$p(y, \theta, \pi) = p(y|\theta, \pi)\, p(\pi)\, p(\theta),$$

where $p(y|\theta, \pi)$ is derived by marginalizing z out of

$$p(z, y|\theta, \pi) \;=\; \prod_{i=1}^{I} \left(\mathsf{Categorical}(z_i|\pi) \prod_{j=1}^{J} \mathsf{Categorical}(y_{i,j}|\theta_{j,z[i]}) \right).$$

This can be done item by item, with

$$p(y|\theta, \pi) \;=\; \prod_{i=1}^{I} \sum_{k=1}^{K} \left(\mathsf{Categorical}(z_i|\pi) \prod_{j=1}^{J} \mathsf{Categorical}(y_{i,j}|\theta_{j,z[i]}) \right).$$

In the missing data model, only the observed labels would be used in the inner product.

Dawid and Skene (1979) derive exactly the same equation in their Equation (2.7), required for the E-step in their expectation maximization (EM) algorithm. Stan requires the marginalized probability function on the log scale,

$$\begin{aligned} &\log p(y|\theta, \pi) \\ &\quad = \; \textstyle\sum_{i=1}^{I} \log \left(\sum_{k=1}^{K} \exp \left(\log \mathsf{Categorical}(z_i|\pi) + \sum_{j=1}^{J} \log \mathsf{Categorical}(y_{i,j}|\theta_{j,z[i]}) \right) \right), \end{aligned}$$

which can be directly coded using Stan's built-in `log_sum_exp` function.

```
data {
  int<lower=2> K;
  int<lower=1> I;
  int<lower=1> J;

  int<lower=1,upper=K> y[I,J];

  vector<lower=0>[K] alpha;
  vector<lower=0>[K] beta[K];
}
parameters {
  simplex[K] pi;
  simplex[K] theta[J,K];
}
transformed parameters {
  vector[K] log_q_z[I];
  for (i in 1:I) {
    log_q_z[i] <- log(pi);
    for (j in 1:J)
      for (k in 1:K)
        log_q_z[i,k] <- log_q_z[i,k]
                        + log(theta[j,k,y[i,j]]);
  }
}
model {
  pi ~ dirichlet(alpha);
  for (j in 1:J)
    for (k in 1:K)
      theta[j,k] ~ dirichlet(beta[k]);

  for (i in 1:I)
    increment_log_prob(log_sum_exp(log_q_z[i]));
}
```

Figure 11.5: *Stan program for the rating (or diagnostic accuracy) model of Dawid and Skene (1979). The model marginalizes out the discrete parameter z, storing the unnormalized conditional probability* $\log q(z_i = k|\theta,\pi)$ *in* `log_q_z[i,k]`.

Stan Implementation

The Stan program for the Dawid and Skene model is provided in Figure 11.5. The Stan model converges quickly and mixes well using NUTS starting at diffuse initial points, unlike the equivalent model implemented with Gibbs sampling over the dis-

crete parameter. Reasonable weakly informative priors are $\alpha_k = 3$ and $\beta_{k,k} = 2.5K$ and $\beta_{k,k'} = 1$ if $k \neq k'$. Taking α and β_k to be unit vectors and applying optimization will produce the same answer as the expectation maximization (EM) algorithm of Dawid and Skene (1979).

Inference for the True Category

The quantity `log_q_z[i]` is defined as a transformed parameter. It encodes the (unnormalized) log of $p(z_i|\theta, \pi)$. Each iteration provides a value conditioned on that iteration's values for θ and π. Applying the softmax function to `log_q_z[i]` provides a simplex corresponding to the probability mass function of z_i in the posterior. These may be averaged across the iterations to provide the posterior probability distribution over each z_i.

12. Clustering Models

Unsupervised methods for organizing data into groups are collectively referred to as clustering. This chapter describes the implementation in Stan of two widely used statistical clustering models, soft K-means and latent Dirichlet allocation (LDA). In addition, this chapter includes naive Bayesian classification, which can be viewed as a form of clustering which may be supervised. These models are typically expressed using discrete parameters for cluster assignments. Nevertheless, they can be implemented in Stan like any other mixture model by marginalizing out the discrete parameters (see Chapter 9).

12.1. Soft K-Means

K-means clustering is a method of clustering data represented as D-dimensional vectors. Specifically, there will be N items to be clustered, each represented as a vector $y_n \in \mathbb{R}^D$. In the “soft” version of K-means, the assignments to clusters will be probabilistic.

Geometric Hard K-Means Clustering

K-means clustering is typically described geometrically in terms of the following algorithm, which assumes the number of clusters K and data vectors y as input.

1. For each n in $1:N$, randomly assign vector y_n to a cluster in $1{:}K$;
2. Repeat
   (a) For each cluster k in $1{:}K$, compute the cluster centroid μ_k by averaging the vectors assigned to that cluster;
   (b) For each n in $1:N$, reassign y_n to the cluster k to for which the (Euclidean) distance from y_n to μ_k is smallest;
   (c) If no vectors changed cluster, return the cluster assignments.

This algorithm is guaranteed to terminate.

Soft K-Means Clustering

Soft K-means clustering treats the cluster assignments as probability distributions over the clusters. Because of the connection between Euclidean distance and multivariate normal models with a fixed covariance, soft K-means can be expressed (and coded in Stan) as a multivariate normal mixture model.

In the full generative model, each data point n in $1{:}N$ is assigned a cluster $z_n \in 1{:}K$ with symmetric uniform probability,

$$z_n \sim \mathsf{Categorical}(\mathbf{1}/K),$$

where $\mathbf{1}$ is the unit vector of K dimensions, so that $\mathbf{1}/K$ is the symmetric K-simplex. Thus the model assumes that each data point is drawn from a hard decision about cluster membership. The softness arises only from the uncertainty about which cluster generated a data point.

The data points themselves are generated from a multivariate normal distribution whose parameters are determined by the cluster assignment z_n,

$$y_n \sim \mathsf{Normal}(\mu_{z[n]}, \Sigma_{z[n]})$$

The sample implementation in this section assumes a fixed unit covariance matrix shared by all clusters k,

$$\Sigma_k = \text{diag\_matrix}(\mathbf{1}),$$

so that the log multivariate normal can be implemented directly up to a proportion by

$$\text{Normal}\left(y_n|\mu_k, \text{diag\_matrix}(\mathbf{1})\right) \propto \exp\left(-\frac{1}{2}\sum_{d=1}^{D}\left(\mu_{k,d} - y_{n,d}\right)^2\right).$$

The spatial perspective on K-means arises by noting that the inner term is just half the negative Euclidean distance from the cluster mean μ_k to the data point y_n.

Stan Implementation of Soft K-Means

The following model is available in the Stan distribution (along with an R program to randomly generate data sets and a sample data set) in the directory `stan/example-models/misc/soft-k-means`.

```
data {
  int<lower=0> N;  // number of data points
  int<lower=1> D;  // number of dimensions
  int<lower=1> K;  // number of clusters
  vector[D] y[N];  // observations
}
transformed data {
  real<upper=0> neg_log_K;
  neg_log_K <- -log(K);
}
parameters {
  vector[D] mu[K]; // cluster means
```

```
}
transformed parameters {
  real<upper=0> soft_z[N,K]; // log unnormalized clusters
  for (n in 1:N)
    for (k in 1:K)
      soft_z[n,k] <- neg_log_K
                     - 0.5 * dot_self(mu[k] - y[n]);
}
model {
  // prior
  for (k in 1:K)
    mu[k] ~ normal(0,1);

  // likelihood
  for (n in 1:N)
    increment_log_prob(log_sum_exp(soft_z[n]));
}
```

There is an independent unit normal prior on the centroid parameters; this prior could be swapped with other priors, or even a hierarchical model to fit an overall problem scale and location.

The only parameter is `mu`, where `mu[k]` is the centroid for cluster k. The transformed parameters `soft_z[n]` contain the log of the unnormalized cluster assignment probabilities. The vector `soft_z[n]` can be converted back to a normalized simplex using the softmax function (see Section 33.6), either externally externally or within the model's generated quantities block.

Generalizing Soft K-Means

The multivariate normal distribution with unit covariance matrix produces a log probability density proportional to Euclidean distance (i.e., L_2 distance). Other distributions relate to other geometries. For instance, replacing the normal distribution with the double exponential (Laplace) distribution produces a clustering model based on L_1 distance (i.e., Manhattan or taxicab distance).

Within the multivariate normal version of K-means, replacing the unit covariance matrix with a shared covariance matrix amounts to working with distances defined in a space transformed by the inverse covariance matrix.

Although there is no global spatial analog, it is common to see soft K-means specified with a per-cluster covariance matrix. In this situation, a hierarchical prior may be used for the covariance matrices.

12.2. The Difficulty of Bayesian Inference for Clustering

Two problems make it pretty much impossible to perform full Bayesian inference for clustering models, the lack of parameter identifiability and the extreme multimodality of the posteriors. There is additional discussion related to the non-identifiability due to label swtiching in Section 18.2.

Non-Identifiability

Cluster assignments are not identified — permuting the cluster mean vectors `mu` leads to a model with identical likelihoods. For instance, permuting the first two indexes in `mu` and the first two indexes in each `soft_z[n]` leads to an identical likelihood (and prior).

The lack of identifiability means that the the cluster parameters cannot be compared across multiple Markov chains. In fact, the only parameter in soft K-means is not identified, leading to problems in monitoring convergence. Clusters can even fail to be identified within a single chain, with indices swapping if the chain is long enough or the data is not cleanly separated.

Multimodality

The other problem with clustering models is that their posteriors are highly multimodal. One form of multimodality is the non-identifiability leading to index swapping. But even without the index problems the posteriors are highly mulitmodal.

Bayesian inference fails in cases of high multimodality because there is no way to visit all of the modes in the posterior in appropriate proportions and thus no way to evaluate integrals involved in posterior predictive inference.

In light of these two problems, the advice often given in fitting clustering models is to try many different initializations and select the sample with the highest overall probability. It is also popular to use optimization-based point estimators such as expectation maximization or variational Bayes, which can be much more efficient than sampling-based approaches.

12.3. Naive Bayes Classification and Clustering

Naive Bayes is a kind of mixture model that can be used for classification or for clustering (or a mix of both), depending on which labels for items are observed.[1]

[1] For clustering, the non-identifiability problems for all mixture models present a problem, whereas there is no such problem for classification. Despite the difficulties with full Bayesian inference for clustering, researchers continue to use it, often in an explorata data analysis setting rather than for predictive modeling.

Multinomial mixture models are referred to as "naive Bayes" because they are often applied to classification problems where the multinomial independence assumptions are clearly false.

Naive Bayes classification and clustering can be applied to any data with multinomial structure. A typical example of this is natural language text classification and clustering, which is used an example in what follows.

The observed data consists of a sequence of M documents made up of bags of words drawn from a vocabulary of V distinct words. A document m has N_m words, which are indexed as $w_{m,1}, \ldots, w_{m,N[m]} \in 1{:}V$. Despite the ordered indexing of words in a document, this order is not part of the model, which is clearly defective for natural human language data. A number of topics (or categories) K is fixed.

The multinomial mixture model generates a single category $z_m \in 1{:}K$ for each document $m \in 1{:}M$ according to a categorical distribution,

$$z_m \sim \mathsf{Categorical}(\theta).$$

The K-simplex parameter θ represents the prevalence of each category in the data.

Next, the words in each document are generated conditionally independently of each other and the words in other documents based on the category of the document, with word n of document m being generated as

$$w_{m,n} \sim \mathsf{Categorical}(\phi_{z[m]}).$$

The parameter $\phi_{z[m]}$ is a V-simplex representing the probability of each word in the vocabulary in documents of category z_m.

The parameters θ and π are typically given symmetric Dirichlet priors. The prevalence θ is sometimes fixed to produce equal probabilities for each category $k \in 1 : K$.

Representing Ragged Arrays in Stan

The specification for naive Bayes in the previous sections have used a ragged array notation for the words w. Because Stan does not support ragged arrays, the models are coded using an alternative strategy that provides an index for each word in a global list of words. The data is organized as follows, with the word arrays layed out in a column and each assigned to its document in a second column.

n	w[n]	doc[n]
1	$w_{1,1}$	1
2	$w_{1,2}$	1
$\vdots$	$\vdots$	$\vdots$
N_1	$w_{1,N[1]}$	1
$N_1 + 1$	$w_{2,1}$	2
$N_1 + 2$	$w_{2,2}$	2
$\vdots$	$\vdots$	$\vdots$
$N_1 + N_2$	$w_{2,N[2]}$	2
$N_1 + N_2 + 1$	$w_{3,1}$	3
$\vdots$	$\vdots$	$\vdots$
$\mathrm{N} = \sum_{m=1}^{M} N_m$	$w_{M,N[M]}$	M

The relevant variables for the program are N, the total number of words in all the documents, the word array w, and the document identity array doc.

Estimation with Category-Labeled Training Data

The naive Bayes models along with R programs to simulate data for them and a sample data set are available in the distribution in the directory example-models/misc/clustering/naive-bayes.

A naive Bayes model for estimating the simplex parameters given training data with documents of known categories can be coded in Stan as follows

```
data {
  // training data
  int<lower=1> K;               // num topics
  int<lower=1> V;               // num words
  int<lower=0> M;               // num docs
  int<lower=0> N;               // total word instances
  int<lower=1,upper=K> z[M];    // topic for doc m
  int<lower=1,upper=V> w[N];    // word n
  int<lower=1,upper=M> doc[N];  // doc ID for word n
  // hyperparameters
  vector<lower=0>[K] alpha;     // topic prior
  vector<lower=0>[V] beta;      // word prior
}
parameters {
  simplex[K] theta;   // topic prevalence
  simplex[V] phi[K];  // word dist for topic k
}
model {
```

```
  theta ~ dirichlet(alpha);
  for (k in 1:K)
    phi[k] ~ dirichlet(beta);
  for (m in 1:M)
    z[m] ~ categorical(theta);
  for (n in 1:N)
    w[n] ~ categorical(phi[z[doc[n]]]);
}
```

Note that the topic identifiers z_m are declared as data and the latent category assignments are included as part of the likelihood function.

Estimation without Category-Labeled Training Data

Naive Bayes models can be used in an unsupervised fashion to cluster multinomial-structured data into a fixed number K of categories. The data declaration includes the same variables as the model in the previous section excluding the topic labels z. Because z is discrete, it needs to be summed out of the model calculation. This is done for naive Bayes as for other mixture models. The parameters are the same up to the priors, but the likelihood is now computed as the marginal document probability

$$\begin{aligned}
&\log p(w_{m,1}, \ldots, w_{m,N_m} | \theta, \phi) \\
&= \log \textstyle\sum_{k=1}^{K} \left(\mathsf{Categorical}(k|\theta) \times \prod_{n=1}^{N_m} \mathsf{Categorical}(w_{m,n}|\phi_k) \right) \\
&= \log \textstyle\sum_{k=1}^{K} \exp \left(\log \mathsf{Categorical}(k|\theta) + \sum_{n=1}^{N_m} \log \mathsf{Categorical}(w_{m,n}|\phi_k) \right).
\end{aligned}$$

The last step shows how the `log_sum_exp` function can be used to stabilize the numerical calculation and return a result on the log scale.

```
model {
  real gamma[M,K];
  theta ~ dirichlet(alpha);
  for (k in 1:K)
    phi[k] ~ dirichlet(beta);
  for (m in 1:M)
    for (k in 1:K)
      gamma[m,k] <- categorical_log(k,theta);
  for (n in 1:N)
    for (k in 1:K)
      gamma[doc[n],k] <- gamma[doc[n],k]
                         + categorical_log(w[n],phi[k]);
  for (m in 1:M)
    increment_log_prob(log_sum_exp(gamma[m]));
}
```

The local variable `gamma[m,k]` represents the value

$$\gamma_{m,k} = \log \mathsf{Categorical}(k|\theta) + \sum_{n=1}^{N_m} \log \mathsf{Categorical}(w_{m,n}|\phi_k).$$

Given γ, the posterior probability that document m is assigned category k is

$$\Pr[z_m = k|w, \alpha, \beta] = \exp\left(\gamma_{m,k} - \log \sum_{k=1}^{K} \exp\left(\gamma_{m,k}\right)\right).$$

If the variable `gamma` were declared and defined in the transformed parameter block, its sampled values would be saved by Stan. The normalized posterior probabilities could also be defined as generated quantities.

Full Bayesian Inference for Naive Bayes

Full Bayesian posterior predictive inference for the naive Bayes model can be implemented in Stan by combining the models for labeled and unlabeled data. The estimands include both the model parameters and the posterior distribution over categories for the unlabeled data. The model is essentially a missing data model assuming the unknown category labels are missing completely at random; see (Gelman et al., 2013; Gelman and Hill, 2007) for more information on missing data imputation. The model is also an instance of semisupervised learning because the unlabeled data contributes to the parameter estimations.

To specify a Stan model for performing full Bayesian inference, the model for labeled data is combined with the model for unlabeled data. A second document collection is declared as data, but without the category labels, leading to new variables `M2` `N2`, `w2`, `doc2`. The number of categories and number of words, as well as the hyperparameters are shared and only declared once. Similarly, there is only one set of parameters. Then the model contains a single set of statements for the prior, a set of statements for the labeled data, and a set of statements for the unlabeled data.

Prediction without Model Updates

An alternative to full Bayesian inference involves estimating a model using labeled data, then applying it to unlabeled data without updating the parameter estimates based on the unlabeled data. This behavior can be implemented by moving the definition of `gamma` for the unlabeled documents to the generated quantities block. Because the variables no longer contribute to the log probability, they no longer jointly contribute to the estimation of the model parameters.

12.4. Latent Dirichlet Allocation

Latent Dirichlet allocation (LDA) is a mixed-membership multinomial clustering model (Blei et al., 2003) that generalized naive Bayes. Using the topic and document terminology common in discussions of LDA, each document is modeled as having a mixture of topics, with each word drawn from a topic based on the mixing proportions.

The LDA Model

The basic model assumes each document is generated independently based on fixed hyperparameters. For document m, the first step is to draw a topic distribution simplex θ_m over the K topics,

$$\theta_m \sim \mathsf{Dirichlet}(\alpha).$$

The prior hyperparameter α is fixed to a K-vector of positive values. Each word in the document is generated independently conditional on the distribution θ_m. First, a topic $z_{m,n} \in 1{:}K$ is drawn for the word based on the document-specific topic-distribution,

$$z_{m,n} \sim \mathsf{Categorical}(\theta_m).$$

Finally, the word $w_{m,n}$ is drawn according to the word distribution for topic $z_{m,n}$,

$$w_{m,n} \sim \mathsf{Categorical}(\phi_{z[m,n]}).$$

The distributions ϕ_k over words for topic k are also given a Dirichlet prior,

$$\phi_k \sim \mathsf{Dirichlet}(\beta)$$

where β is a fixed V-vector of positive values.

Summing out the Discrete Parameters

Although Stan does not (yet) support discrete sampling, it is possible to calculate the marginal distribution over the continuous parameters by summing out the discrete parameters as in other mixture models. The marginal posterior of the topic and word variables is

$$\begin{aligned} p(\theta, \phi | w, \alpha, \beta) &\propto p(\theta|\alpha) \times p(\phi|\beta) \times p(w|\theta, \phi) \\ &= \prod_{m=1}^{M} p(\theta_m|\alpha) \times \prod_{k=1}^{K} p(\phi_k|\beta) \times \prod_{m=1}^{M} \prod_{n=1}^{M[n]} p(w_{m,n}|\theta_m, \phi). \end{aligned}$$

The inner word-probability term is defined by summing out the topic assignments,

$$
\begin{aligned}
p(w_{m,n}|\theta_m, \phi) &= \sum_{z=1}^{K} p(z, w_{m,n}|\theta_m, \phi). \\
&= \sum_{z=1}^{K} p(z|\theta_m) \times p(w_{m,n}|\phi_z).
\end{aligned}
$$

Plugging the distributions in and converting to the log scale provides a formula that can be implemented directly in Stan,

$$
\begin{aligned}
&\log p(\theta, \phi|w, \alpha, \beta) \\
&\quad = \textstyle\sum_{m=1}^{M} \log \mathsf{Dirichlet}(\theta_m|\alpha) \; + \; \sum_{k=1}^{K} \log \mathsf{Dirichlet}(\phi_k|\beta) \\
&\qquad + \textstyle\sum_{m=1}^{M} \sum_{n=1}^{N[m]} \log \left(\sum_{z=1}^{K} \mathsf{Categorical}(z|\theta_m) \times \mathsf{Categorical}(w_{m,n}|\phi_z) \right)
\end{aligned}
$$

Implementation of LDA

Applying the marginal derived in the last section to the data structure described in this section leads to the following Stan program for LDA.

```
data {
  int<lower=2> K;               // num topics
  int<lower=2> V;               // num words
  int<lower=1> M;               // num docs
  int<lower=1> N;               // total word instances
  int<lower=1,upper=V> w[N];    // word n
  int<lower=1,upper=M> doc[N];  // doc ID for word n
  vector<lower=0>[K] alpha;     // topic prior
  vector<lower=0>[V] beta;      // word prior
}
parameters {
  simplex[K] theta[M];   // topic dist for doc m
  simplex[V] phi[K];     // word dist for topic k
}
model {
  for (m in 1:M)
    theta[m] ~ dirichlet(alpha);  // prior
  for (k in 1:K)
    phi[k] ~ dirichlet(beta);     // prior
  for (n in 1:N) {
    real gamma[K];
    for (k in 1:K)
      gamma[k] <- log(theta[doc[n],k]) + log(phi[k,w[n]]);
```

```
    increment_log_prob(log_sum_exp(gamma));  // likelihood
  }
}
```

As in the other mixture models, the log-sum-of-exponents function is used to stabilize the numerical arithmetic.

Correlated Topic Model

To account for correlations in the distribution of topics for documents, (Blei and Lafferty, 2007) introduced a variant of LDA in which the Dirichlet prior on the per-document topic distribution is replaced with a multivariate logistic normal distribution.

The authors treat the prior as a fixed hyperparameter. They use an L_1-regularized estimate of covariance, which is equivalent to the maximum a posteriori estimate given a double-exponential prior. Stan does not (yet) support maximum a posteriori estimation, so the mean and covariance of the multivariate logistic normal must be specified as data.

Fixed Hyperparameter Correlated Topic Model

The Stan model in the previous section can be modified to implement the correlated topic model by replacing the Dirichlet topic prior `alpha` in the data declaration with the mean and covariance of the multivariate logistic normal prior.

```
data {
  ... data as before without alpha ...
  vector[K] mu;          // topic mean
  cov_matrix[K] Sigma;   // topic covariance
}
```

Rather than drawing the simplex parameter `theta` from a Dirichlet, a parameter `eta` is drawn from a multivariate normal distribution and then transformed using softmax into a simplex.

```
parameters {
  simplex[V] phi[K];  // word dist for topic k
  vector[K] eta[M];   // topic dist for doc m
}
transformed parameters {
  simplex[K] theta[M];
  for (m in 1:M)
    theta[m] <- softmax(eta[m]);
```

```
}
model {
  for (m in 1:M)
    eta[m] ~ multi_normal(mu,Sigma);
  ... model as before w/o prior for theta ...
}
```

Full Bayes Correlated Topic Model

By adding a prior for the mean and covariance, Stan supports full Bayesian inference for the correlated topic model. This requires moving the declarations of topic mean mu and covariance Sigma from the data block to the parameters block and providing them with priors in the model. A relatively efficient and interpretable prior for the covariance matrix Sigma may be encoded as follows.

```
... data block as before, but without alpha ...
parameters {
  vector[K] mu;              // topic mean
  corr_matrix[K] Omega;      // correlation matrix
  vector<lower=0>[K] sigma;  // scales
  vector[K] eta[M];          // logit topic dist for doc m
  simplex[V] phi[K];         // word dist for topic k
}
transformed parameters {
  ... eta as above ...
  cov_matrix[K] Sigma;       // covariance matrix
  for (m in 1:K)
    Sigma[m,m] <- sigma[m] * sigma[m] * Omega[m,m];
  for (m in 1:(K-1)) {
    for (n in (m+1):K) {
      Sigma[m,n] <- sigma[m] * sigma[n] * Omega[m,n];
      Sigma[n,m] <- Sigma[m,n];
    }
  }
}
model {
  mu ~ normal(0,5);       // vectorized, diffuse
  Omega ~ lkj_corr(2.0);  // regularize to unit correlation
  sigma ~ cauchy(0,5);    // half-Cauchy due to constraint
  ... words sampled as above ...
}
```

The LkjCorr distribution with shape $\alpha > 0$ has support on correlation matrices (i.e., symmetric positive definite with unit diagonal). Its density is defined by

$$\mathsf{LkjCorr}(\Omega|\alpha) \propto \det(\Omega)^{\alpha-1}$$

With a scale of $\alpha = 2$, the weakly informative prior favors a unit correlation matrix. Thus the compound effect of this prior on the covariance matrix Σ for the multivariate logistic normal is a slight concentration around diagonal covariance matrices with scales determined by the prior on `sigma`.

13. Gaussian Processes

Gaussian process are continuous stochastic processes and thus may be interpreted as providing a probability distribution over functions. A probability distribution over continuous functions may be viewed, roughly, as an uncountably infinite collection of random variables, one for each valid input. The generality of the supported functions makes Gaussian priors popular choices for priors in general multivariate (non-linear) regression problems.

The defining feature of a Gaussian process is that the distribution of the function's value at a finite number of input points is a multivariate normal distribution. This makes it tractable to both fit models from finite amounts of observed data and make predictions for finitely many new data points.

Unlike a simple multivariate normal distribution, which is parameterized by a mean vector and covariance matrix, a Gaussian process is parameterized by a mean function and covariance function. The mean and covariance functions apply to vectors of inputs and return a mean vector and covariance matrix which provide the mean and covariance of the outputs corresponding to those input points in the functions drawn from the process.

Gaussian processes can be encoded in Stan by implementing their mean and covariance functions and plugging the result into the Gaussian form of their sampling distribution. This form of model is easy to understand and may be used for simulation, model fitting, or posterior predictive inference. More efficient Stan implementation for the basic (non-logistic) regression applies a Cholesky-factor reparameterization of the Gaussian and computes the posterior predictive distribution analytically.

After defining Gaussian processes, this chapter covers the basic implementations for simulation, hyperparameter estimation, and posterior predictive inference for univariate regressions, multivariate regressions, and multivariate logistic regressions. Gaussian processes are very general, and by necessity this chapter only touches on some basic models. For more information, see (Rasmussen and Williams, 2006).

13.1. Gaussian Process Regression

The data for a multivariate Gaussian process regression consists of a series of N inputs $x_1, \ldots, x_N \in \mathbb{R}^D$ paired with outputs $y_1, \ldots, y_N \in \mathbb{R}$. The defining feature of Gaussian processes is that the probability of a finite number of outputs y conditioned on their inputs x is Gaussian,

$$y \sim \mathsf{Normal}(m(x), k(x)),$$

where $m(x)$ is an N-vector and $k(x)$ is an $N \times N$ covariance matrix. The mean function $m : \mathbb{R}^{N \times D} \to \mathbb{R}^N$ can be anything, but the covariance function $k : \mathbb{R}^{N \times D} \to \mathbb{R}^{N \times N}$ must

produce a positive-definite matrix for any input x.[1]

A popular covariance function, which will be used in the implementations later in this chapter, is a generalized, squared exponential function,

$$k(x)_{i,j} = \eta^2 \exp\left(-\rho^2 \sum_{d=1}^{D} (x_{i,d} - x_{j,d})^2\right) + \delta_{i,j}\sigma^2,$$

where η, ρ, and σ are hyperparameters defining the covariance function and where $\delta_{i,j}$ is the Kronecker delta function with value 1 if $i = j$ and value 0 otherwise; note that this test is between the indexes i and j, not between values x_i and x_j. The addition of σ^2 on the diagonal is important to ensure the positive definiteness of the resulting matrix in the case of two identical inputs $x_i = x_j$. In statistical terms, σ is the scale of the noise term in the regression.

The only term in the squared exponential covariance function involving the inputs x_i and x_j is their vector difference, $x_i - x_j$. This produces a process with stationary covariance in the sense that if an input vector x is translated by a vector ϵ to $x + \epsilon$, the covariance at any pair of outputs is unchanged, because $k(x) = k(x + \epsilon)$.

The summation involved is just the squared Euclidean distance between x_i and x_j (i.e., the L_2 norm of their difference, $x_i - x_j$). This results in support for smooth functions in the process. The amount of variation in the function is controlled by the free hyperparameters η, ρ, and σ.

Changing the notion of distance from Euclidean to taxicab distance (i.e., an L_1 norm) changes the support to functions which are continuous but not smooth.

13.2. Simulating from a Gaussian Process

It is simplest to start with a Stan model that does nothing more than simulate draws of functions f from a Gaussian process. In practical terms, the model will draw values $y_n = f(x_n)$ for finitely many input points x_n.

The Stan model defines the mean and covariance functions in a transformed data block and then samples outputs y in the model using a multivariate normal distribution. To make the model concrete, the squared exponential covariance function described in the previous section will be used with hyperparameters set to $\eta^2 = 1$, $\rho^2 = 1$, and $\sigma^2 = 0.1$, and the mean function m is defined to always return the zero vector, $m(x) = \mathbf{0}$. The following model is included in the Stan distribution in file `example-models/misc/gaussian-process/gp-sim.stan`.

[1]Gaussian processes can be extended to covariance functions producing positive semi-definite matrices, but Stan does not support inference in the resulting models because the resulting distribution does not have unconstrained support.

```
data {
  int<lower=1> N;
  real x[N];
}
transformed data {
  vector[N] mu;
  cov_matrix[N] Sigma;
  for (i in 1:N)
    mu[i] <- 0;
  for (i in 1:N)
    for (j in 1:N)
      Sigma[i,j] <- exp(-pow(x[i] - x[j],2))
                   + if_else(i==j, 0.1, 0.0);
}
parameters {
  vector[N] y;
}
model {
  y ~ multi_normal(mu,Sigma);
}
```

The input data is just the vector of inputs `x` and its size `N`. Such a model can be used with values of `x` evenly spaced over some interval in order to plot sample draws of functions from a Gaussian process. The covariance matrix `Sigma` is not being computed efficiently here; see Section Section 13.3 for a better approach.

Multivariate Inputs

Only the covariance function's distance computation needs to change in moving from a univariate model to a multivariate model. A multivariate sampling model is available in the source distribution at `example-models/misc/gaussian-process/gp-multi-sim.stan`. The only lines that change from the univariate model above are as follows.

```
data {
  int<lower=1> D;
  int<lower=1> N;
  vector[D] x[N];
}
transformed data {
...
      Sigma[i,j] <- exp(-dot_self(x[i] - x[j]))
                   + if_else(i==j, 0.1, 0.0);
...
```

The data is now declared as an array of vectors instead of an array of scalars; the dimensionality `D` is also declared. The squared Euclidean distance calculation is done using the `dot_self` function, which returns the dot product of its argument with itself, here `x[i] - x[j]`.

In the remainder of the chapter, univariate models will be used for simplicity, but any of them could be changed to multivariate in the same way as the simple sampling model. The only extra computational overhead from a multivariate model is in the distance calculation, which is only done once when the transformed data block is run after the data is read.

Cholesky Factored and Transformed Implementation

A much more efficient implementation of the simulation model can be coded in Stan by relocating, rescaling and rotating an isotropic unit normal variate. Suppose z is an an isotropic unit normal variate

$$z \sim \mathsf{Normal}(\mathbf{0}, \mathbf{1}),$$

where $\mathbf{0}$ is an N-vector of 0 values and $\mathbf{1}$ is the $N \times N$ unit matrix. Let L be the the Cholesky decomposition of $k(x)$, i.e., the lower-triangular matrix L such that $LL^\top = k(x)$. Then the transformed variable $\mu + Lz$ has the intended target distribution,

$$\mu + Lz \sim \mathsf{Normal}(\mu, k(x)).$$

This transform can be applied directly to Gaussian process simulation, as shown in the model `example-models/misc/gaussian-process/gp-sim-cholesky.stan` in the distribution. This model has the same data declarations for `N` and `x`, and the same transformed data definitions of `mu` and `Sigma` as the previous model, with the addition of a transformed data variable for the Cholesky decomposition. The parameters change to the raw parameters sampled from an isotropic unit normal, and the actual samples are defined as generated quantities.

```
...
transformed data {
  matrix[N,N] L;
...
  L <- cholesky_decompose(Sigma);
}
parameters {
  vector[N] z;
}
model {
  z ~ normal(0,1);
```

```
}
generated quantities {
  vector[N] y;
  y <- mu + L * z;
}
```

The Cholesky decomposition is only computed once, after the data is loaded and the covariance matrix `Sigma` computed. The isotropic normal distribution for `z` is specified as a vectorized univariate distribution for efficiency; this specifies that each `z[n]` has an independent unit normal distribution. The sampled vector `y` is then defined as a generated quantity using a direct encoding of the transform described above.

13.3. Fitting a Gaussian Process

The hyperparameters controlling the covariance function of a Gaussian process can be fit by assigning them priors, then computing the posterior distribution of the hyperparameters given observed data. Because the hyperparameters are required to be positive and expected to have reasonably small values, broad half-Cauchy distributions act as quite vague priors which could just as well be uniform over a constrained range of values. The priors on the parameters should be defined based on prior knowledge of the scale of the output values (η), the scale of the output noise (σ), and the scale at which distances are measured among inputs ($1/\rho$).

A Stan model to fit the hyperparameters of the general squared exponential covariance function is provided in the distribution in `example-models/misc/gaussian-process/gp-fit.stan`. The Stan code is very similar to the simulation models in terms of the computations, but the blocks in which variables are declared and statements are executed has changed to accommodate the hyperparameter estimation problem.

```
data {
  int<lower=1> N;
  vector[N] x;
  vector[N] y;
}
transformed data {
  vector[N] mu;
  for (i in 1:N) mu[i] <- 0;
}
parameters {
  real<lower=0> eta_sq;
  real<lower=0> rho_sq;
```

```
    real<lower=0> sigma_sq;
  }
  model {
    matrix[N,N] Sigma;
    // off-diagonal elements
    for (i in 1:(N-1)) {
      for (j in (i+1):N) {
        Sigma[i,j] <- eta_sq * exp(-rho_sq * pow(x[i] - x[j],2));
        Sigma[j,i] <- Sigma[i,j];
      }
    }
    // diagonal elements
    for (k in 1:N)
      Sigma[k,k] <- eta_sq + sigma_sq;  // + jitter

    eta_sq ~ cauchy(0,5);
    rho_sq ~ cauchy(0,5);
    sigma_sq ~ cauchy(0,5);

    y ~ multi_normal(mu,Sigma);
  }
```

The data block now declares a vector `y` of observed values `y[n]` for inputs `x[n]`. The transformed data block now only defines the mean vector to be zero. The three hyperparameters are defined as parameters constrained to be non-negative. The computation of the covariance matrix `Sigma` is now in the model block because it involves unknown parameters and thus can't simply be precomputed as transformed data. The rest of the model consists of the priors for the hyperparameters and the multivariate normal likelihood, only now the value `y` is known and the covariance matrix `Sigma` is an unknown dependent on the hyperparameters.

Hamiltonian Monte Carlo sampling is quite fast and effective for hyperparameter inference in this model (Neal, 1997), and the Stan implementation will fit hyperparameters in models with hundreds of data points in seconds.

Automatic Relevance Determination

"Automatic relevance determination" is the term used in machine learning corresponding to the term "hierarchical modeling" in statistics. In either case, the idea is that hyperparameters are estimated from data (in Bayesian inference, via the joint posteiror distribution) rather than being preset.

For multivariate inputs $x \in \mathbb{R}^D$, the squared exponential covariance function can

be further generalized by fitting a precision parameter ρ_d^2 for each dimension d,

$$k(x)_{i,j} = \eta^2 \exp\left(-\sum_{d=1}^{D} \rho_d^2 (x_{i,d} - x_{j,d})^2\right) + \delta_{i,j}\sigma^2.$$

The estimation of ρ was termed "automatic relevance determination" in (Neal, 1996a), because the larger ρ_d is, the more dimension d is weighted in the distance calculation.

The implementation of automatic relevance determination in Stan is straightforward. A model like the one to fit the basic hyperparameters can be generalized by declaring `rho` to be a vector of size `D` and defining the covariance function as in this subsection.

The collection of ρ_d parameters can also be modeled hierarchically.

13.4. Predictive Inference with a Gaussian Process

Suppose for a given sequence of inputs x that the corresponding outputs y are observed. Given a new sequence of inputs $\tilde{x}$, the posterior predictive distribution of their labels is computed by sampling outputs $\tilde{y}$ according to

$$p(\tilde{y}|\tilde{x}, x, y) = \frac{p(\tilde{y}, y|\tilde{x}, x)}{p(y|x)} \propto p(\tilde{y}, y|\tilde{x}, x).$$

A direct implementation in Stan defines a model in terms of the the joint distribution of the observed y and unobserved $\tilde{y}$. Although Stan does not support mixed vectors of parameters and data directly, such a vector may be synthesized as a local variable in the model block. The following model, which takes this approach, is available in the distribution as `example-models/misc/gaussian-process/gp-predict.stan`.

```
data {
  int<lower=1> N1;
  vector[N1] x1;
  vector[N1] y1;
  int<lower=1> N2;
  vector[N2] x2;
}
transformed data {
  int<lower=1> N;
  vector[N1+N2] x;
  vector[N1+N2] mu;
  cov_matrix[N1+N2] Sigma;
  N <- N1 + N2;
  for (n in 1:N1) x[n] <- x1[n];
```

```
  for (n in 1:N2) x[N1 + n] <- x2[n];
  for (i in 1:N) mu[i] <- 0;
  for (i in 1:N)
    for (j in 1:N)
      Sigma[i,j] <- exp(-pow(x[i] - x[j],2))
                    + if_else(i==j, 0.1, 0.0);
}
parameters {
  vector[N2] y2;
}
model {
  vector[N] y;
  for (n in 1:N1) y[n] <- y1[n];
  for (n in 1:N2) y[N1 + n] <- y2[n];

  y ~ multi_normal(mu,Sigma);
}
```

The input vectors x1 and x2 are declared as data, as is the observed output vector y1. The unknown output vector y2, which corresponds to input vector x2, is declared as a parameter and will be sampled when the model is executed.

A transformed data block is used to combine the input vectors x1 and x2 into a single vector x. The covariance function is then applied to this combined input vector to produce the covariance matrix Sigma. The mean vector mu is also declared and set to zero.

The model block declares and define a local variable for the combined output vector y, which consists of the concatenation of the known outputs y1 and unknown outputs y2. Thus the combined output vector y is aligned with the combined input vector x. All that is left is to define the multivariate normal sampling statement for y.

Cholesky Factorization Speedup

This model could be sped up fairly substantially by computing the Cholesky factor of Sigma in the transformed data block

```
transformed data {
  matrix[N1+N2,N1+N2] L;
...
  L <- cholesky_decompose(Sigma);
...
```

and then replacing multi_normal with the more efficient multi_normal_cholesky in the model block.

```
...
model {
...
  y ~ multi_normal_cholesky(mu,L);
}
```

At this point, Sigma could be declared as a local variable in the data block so that its memory may be recovered after the data is loaded.

Analytical Form of Joint Predictive Inference

Bayesian predictive inference for Gaussian processes can be sped up by deriving the posterior analytically, then directly sampling from it. This works for standard Gaussian processes, but not generalizations such as logistic Gaussian process regression.

Jumping straight to the result,

$$p(\tilde{y}|\tilde{x}, y, x) = \mathsf{Normal}(K^{\top}\Sigma^{-1}y,\ \Omega - K^{\top}\Sigma^{-1}K),$$

where $\Sigma = k(x)$ is the result of applying the covariance function to the inputs x with observed outputs y, $\Omega = k(\tilde{x})$ is the result of applying the covariance function to the inputs $\tilde{x}$ for which predictions are to be inferred, and K is the matrix of covariances between inputs x and $\tilde{x}$, which in the case of the generalized squared exponential covariance function would be

$$K_{i,j} = \eta^2 \exp(-\rho^2 \sum_{d=1}^{D} (x_{i,d} - \tilde{x}_{j,d})^2).$$

There is no noise term including σ^2 because the indexes of elements in x and $\tilde{x}$ are never the same.

Because a Stan model is only required to be proportional to the posterior, the posterior may be coded directly. An example that uses the analytic form of the posterior and provides sampling of the resulting multivariate normal through the Cholesky decomposition is provided in example-models/misc/gaussian-process/gp-predict-analytic.stan. The data declaration is the same as for the standard example. The calculation of the predictive mean mu and covariance Cholesky factor L is done in the transformed data block.

```
transformed data {
  vector[N2] mu;
  matrix[N2,N2] L;
  {
    matrix[N1,N1] Sigma;
    matrix[N2,N2] Omega;
```

```
    matrix[N1,N2] K;

    matrix[N2,N1] K_transpose_div_Sigma;
    matrix[N2,N2] Tau;

    for (i in 1:N1)
      for (j in 1:N1)
        Sigma[i,j] <- exp(-pow(x1[i] - x1[j],2))
          + if_else(i==j, 0.1, 0.0);
    for (i in 1:N2)
      for (j in 1:N2)
        Omega[i,j] <- exp(-pow(x2[i] - x2[j],2))
          + if_else(i==j, 0.1, 0.0);
    for (i in 1:N1)
      for (j in 1:N2)
        K[i,j] <- exp(-pow(x1[i] - x2[j],2));

    K_transpose_div_Sigma <- K' / Sigma;
<    mu <- K_transpose_div_Sigma * y1;
    Tau <- Omega - K_transpose_div_Sigma * K;
    for (i in 1:(N2-1))
      for (j in (i+1):N2)
        Tau[i,j] <- Tau[j,i];

    L <- cholesky_decompose(Tau);
  }
}
```

This block implements the definitions of Σ, Ω, and K directly. The posterior mean vector $K^\top \Sigma^{-1} y$ is computed as `mu`. The covariance has a Cholesky factor L such that $LL^\top = \Omega - K^\top \Sigma^{-1} K$. Given these two ingredients, sampling the predictive quantity $\tilde{y}$ is carried out by translating, scaling and rotating an isotropic normal sample using the posterior mean and the Cholesky factorization of the posterior covariance.

Joint Hyperparameter Fitting and Predictive Inference

Hyperparameter fitting may be carried out jointly with predictive inference in a single model. This allows full Bayesian inference to account for the affect of the uncertainty in the hyperparameter estimates on the predictive inferences.

To encode a joint hyperparameter fit and predictive inference model in Stan, declare the hyperparameters as additional parameters, give them a prior in the model, move the definition of `Sigma` to a local variable in the model defined using the hyperparameters.

13.5. Classification with Gaussian Processes

Gaussian processes can be generalized the same way as standard linear models by introducing a link function. This allows them to be used as discrete data models, and in particular to perform classification using posterior predictive inference. This section focuses on binary classification problems implemented with logistic Gaussian process regression.

Logistic Gaussian Process Regression

For binary classification problems, the observed outputs $z_n \in \{0, 1\}$ are binary. These outputs are modeled using a Gaussian process with (unobserved) outputs y_n through the logistic link,

$$z_n \sim \mathsf{Bernoulli}(\mathrm{logit}^{-1}(y_n)),$$

or in other words,

$$\Pr[z_n = 1] = \mathrm{logit}^{-1}(y_n).$$

Simulation

Simulation from a Gaussian process logistic regression is straightforward; just simulate from a Gaussian process and then simulate the z_n from the y_n using the sampling distribution above. Simulation of the z_n must be done in the generated quantities block, where it is possible to draw random variates from specified distributions.

Hyperparameter Estimation and Predictive Inference

For hyperparameter estimation and predictive inference applications, the y_n are typically latent parameters (i.e., not observed). Unfortunately, they cannot be easily marginalized out analytically, so they must be estimated from the data through the observed categorical outputs z_n. Predictive inference will proceed not by sampling z_n values, but directly through their probabilities, given by $\mathrm{logit}^{-1}(y_n)$.

Stan Implementations

Hyperparameter estimation and predictive inference are easily accomplished in Stan by declaring the vector y as a parameter, adding the sampling statements for observed z, and then proceeding as for the previous regression models.

The following full model for prediction using logistic Gaussian process regression is available in the distribution at `example-models/misc/gaussian-process/gp-logit-predict.stan`.

```
data {
  int<lower=1> N1;
  vector[N1] x1;
  int<lower=0,upper=1> z1[N1];
  int<lower=1> N2;
  vector[N2] x2;
}
transformed data {
 ... define mu as zero, compute Sigma from x1, x2 ...
}
parameters {
  vector[N1] y1;
  vector[N2] y2;
}
model {
  vector[N] y;
  for (n in 1:N1) y[n] <- y1[n];
  for (n in 1:N2) y[N1 + n] <- y2[n];

  y ~ multi_normal(mu,Sigma);
  for (n in 1:N1)
    z1[n] ~ bernoulli_logit(y1[n]);
}
```

The transformed data block in which `mu` and `Sigma` are defined is not shown because it is identical to the model for prediction in the previous section. Now the observed outcomes `z1`, declared as data, are binary. The variable `y1` is still drawn from the Gaussian process with values `y1[n]` being the values of the function for input `x1[n]`, only now `y1[n]` is interpreted as the logit-scaled probability that `z1[n]` is 1. The variable `y2` plays the same role for probabilistic predictions for inputs `x2` and is also declared as a parameter.

In the model, the full vector `y` is defined as before by concatenating `y1` and `y2`, only this time both `y1` and `y2` are parameters. The full vector `y` is defined as being multivariate normal as before. Additionally, the `z1[n]` variables are given a Bernoulli distribution with logit-scaled parameters. Only the `z1[n]` values are observed and hence only they are sampled. There is no `z2[n]` vector because Stan does not support discrete sampling; instead, the predictions are in the form of the logit-scaled probabilities `y2`.

Samples form this model do not mix as well as for the standard model. This is largely because the `z1` values are quantized forms of `y1`, and thus provide less precise data for estimation.

The model could be sped up by applying a Cholesky decomposition to the covariance matrix `Sigma` and then replacing the `multi_normal` distribution with

multi_normal_cholesky.

A pure logistic Gaussian process regression would not include a noise term in the definition of the covariance matrix. This can be implemented by simply removing the noise term(s) sigma_sq from the definition of Sigma. Probit regression can be coded by subsituting the probit link for the logit.[2]

This simple prediction model could be extended in the same way as previous models by declaring the hyperparameters as parameters and defining the covariance matrix in the model block as a local variable.

[2]Although it is possible to implement probit regression by including the noise term sigma_sq and then quantizing y1[n] to produce z1[n], this is not feasible in Stan because it requires a complex constraint on y to be enforced for multivariate normal distribution.

14. Reparameterization & Change of Variables

As with BUGS, Stan supports a direct encoding of reparameterizations. Stan also supports changes of variables by directly incrementing the log probability accumulator with the log Jacobian of the transform.

14.1. Reparameterizations

Reparameterizations may be implemented straightforwardly. For example, the Beta distribution is parameterized by two positive count parameters $\alpha, \beta > 0$. The following example illustrates a hierarchical Stan model with a vector of parameters `theta` are drawn i.i.d. for a Beta distribution whose parameters are themselves drawn from a hyperprior distribution.

```
parameters {
  real<lower = 0> alpha;
  real<lower = 0> beta;
  ...
model {
  alpha ~ ...
  beta ~ ...
  for (n in 1:N)
    theta[n] ~ beta(alpha,beta);
  ...
```

It is often more natural to specify hyperpriors in terms of transformed parameters. In the case of the Beta, the obvious choice for reparameterization is in terms of a mean parameter

$$\phi = \alpha/(\alpha + \beta)$$

and total count parameter

$$\lambda = \alpha + \beta.$$

Following (Gelman et al., 2013, Chapter 5), the mean gets a uniform prior and the count parameter a Pareto prior with $p(\lambda) \propto \lambda^{-2.5}$.

```
parameters {
  real<lower=0,upper=1> phi;
  real<lower=0.1> lambda;
  ...
transformed parameters {
  real<lower=0> alpha;
  real<lower=0> beta;
  ...
```

```
    alpha <- lambda * phi;
    beta <- lambda * (1 - phi);
    ...
  model {
    phi ~ beta(1,1); // uniform on phi, could drop
    lambda ~ pareto(0.1,1.5);
    for (n in 1:N)
      theta[n] ~ beta(alpha,beta);
    ...
```

The new parameters, phi and lambda, are declared in the parameters block and the parameters for the Beta distribution, alpha and beta, are declared and defined in the transformed parameters block. And If their values are not of interest, they could instead be defined as local variables in the model as follows.

```
model {
  real alpha;
  real beta;
  alpha <- lambda * phi;
  beta <- lambda * (1 - phi);
...
  for (n in 1:N)
    theta[n] ~ beta(alpha,beta);
...
}
```

With vectorization, this could be expressed more compactly and efficiently as follows.

```
model {
  theta ~ beta(lambda * phi, lambda * (1 - phi));
...
}
```

If the variables alpha and beta are of interest, they can be defined in the transformed parameter block and then used in the model.

Jacobians not Necessary

Because the transformed parameters are being used, rather than given a distribution, there is no need to apply a Jacobian adjustment for the transform. For example, in the beta distribution example, alpha and beta have the correct posterior distribution.

14.2. Changes of Variables

Changes of variables are applied when the transformation of a parameter is characterized by a distribution. The standard textbook example is the lognormal distribution, which is the distribution of a variable $y > 0$ whose logarithm $\log y$ has a normal distribution. Note that the distribution is being assigned to $\log y$.

The change of variables requires an adjustment to the probability to account for the distortion caused by the transform. For this to work, univariate changes of variables must be monotonic and differentiable everywhere in their support.

For univariate changes of variables, the resulting probability must be scaled by the absolute derivative of the transform (see Section 53.1 for more precise definitions of univariate changes of variables).

In the case of log normals, if y's logarithm is normal with mean μ and deviation σ, then the distribution of y is given by

$$p(y) = \mathsf{Normal}(\log y|\mu,\sigma) \left| \frac{d}{dy} \log y \right| = \mathsf{Normal}(\log y|\mu,\sigma) \frac{1}{y}.$$

Stan works on the log scale to prevent underflow, where

$$\log p(y) = \log \mathsf{Normal}(\log y|\mu,\sigma) - \log y.$$

In Stan, the change of variables can be applied in the sampling statement. To adjust for the curvature, the log probability accumulator is incremented with the log absolute derivative of the transform. The lognormal distribution can thus be implemented directly in Stan as follows.[1]

```
parameters {
  real<lower=0> y;
  ...
model {
  log(y) ~ normal(mu,sigma);
  increment_log_prob(-log(y));
  ...
```

It is important, as always, to declare appropriate constraints on parameters; here `y` is constrained to be positive.

It would be slightly more efficient to define a local variable for the logarithm, as follows.

[1]This example is for illustrative purposes only; the recommended way to implement the lognormal distribution in Stan is with the built-in `lognormal` probability function (see Section 40.1).

```
model {
  real log_y;
  log_y <- log(y);
  log_y ~ normal(mu,sigma);
  increment_log_prob(-log_y);
  ...
```

If y were declared as data instead of as a parameter, then the adjustment can be ignored because the data will be constant and Stan only requires the log probability up to a constant.

Change of Variables vs. Transformations

This section illustrates the difference between a change of variables and a simple variable transformation. A transformation samples a parameter, then transforms it, whereas a change of variables transforms a parameter, then samples it. Only the latter requires a Jacobian adjustment.

Note that it does not matter whether the probability function is expressed using a sampling statement, such as

```
log(y) ~ normal(mu,sigma);
```

or as an increment to the log probability function, as in

```
increment_log_prob(normal_log(log(y), mu, sigma));
```

Gamma and Inverse Gamma Distribution

Like the log normal, the inverse gamma distribution is a distribution of variables whose inverse has a gamma distribution. This section contrasts two approaches, first with a transform, then with a change of variables.

The transform based approach to sampling y_inv with an inverse gamma distribution can be coded as follows.

```
parameters {
  real<lower=0> y;
}
transformed parameters {
  real<lower=0> y_inv;
  y_inv <- 1 / y;
}
model {
  y ~ gamma(2,4);
}
```

The change-of-variables approach to sampling y_inv with an inverse gamma distribution can be coded as follows.

```
parameters {
  real<lower=0> y_inv;
}
transformed parameters {
  real<lower=0> y;
  y <- 1 / y_inv;                              // change
  increment_log_prob( -2 * log(y_inv) );  // adjustment
}
model {
  y ~ gamma(2,4);
}
```

The Jacobian adjustment is the log of the absolute derivative of the transform, which in this case is

$$\log \left| \frac{d}{du} \left(\frac{1}{u} \right) \right| \;=\; \log |-u^{-2}| \;=\; \log u^{-2} \;=\; -2 \log u.$$

Multivariate Changes of Variables

In the case of a multivariate transform, the log of the Jacobian of the transform must be added to the log probability accumulator (see the subsection of Section 53.1 on multivarate changes of variables for more precise definitions of multivariate transforms and Jacobians). In Stan, this can be coded as follows in the general case where the Jacobian is not a full matrix.

```
parameters {
  vector[K] u;      // multivariate parameter
   ...
transformed parameters {
  vector[K] v;     // transformed parameter
  matrix[K,K] J;   // Jacobian matrix of transform
  ... compute v as a function of u ...
  ... compute J[m,n] = d.v[m] / d.u[n] ...
  increment_log_prob(log(fabs(determinant(J))));
  ...
model {
  v ~ ...;
  ...
```

Of course, if the Jacobian is known analytically, it will be more efficient to apply it directly than to call the determinant function, which is neither efficient nor particularly stable numerically.

In many cases, the Jacobian matrix will be triangular, so that only the diagonal elements will be required for the determinant calculation. Triangular Jacobians arise when each element v[k] of the transformed parameter vector only depends on elements u[1], ..., u[k] of the parameter vector. For triangular matrices, the determinant is the product of the diagonal elements, so the transformed parameters block of the above model can be simplified and made more efficient by recoding as follows.

```
transformed parameters {
  ...
  vector[K] J_diag;  // diagonals of Jacobian matrix
  ...
  ... compute J[k,k] = d.v[k] / d.u[k] ...
  incement_log_prob(sum(log(J_diag)));
  ...
```

15. Custom Probability Functions

Custom distributions may also be implemented directly within Stan's programming language. The only thing that is needed is to increment the total log probability. The rest of the chapter provides two examples.

15.1. Examples

Triangle Distribution

A simple example is the triangle distribution, whose density is shaped like an isosceles triangle with corners at specified bounds and height determined by the constraint that a density integrate to 1. If $\alpha \in \mathbb{R}$ and $\beta \in \mathbb{R}$ are the bounds, with $\alpha < \beta$, then $y \in (\alpha, \beta)$ has a density defined as follows.

$$\mathsf{Triangle}(y|\alpha,\beta) = \frac{2}{\beta-\alpha}\left(1 - \left| y - \frac{\alpha+\beta}{\beta-\alpha} \right| \right)$$

If $\alpha = -1$, $\beta = 1$, and $y \in (-1, 1)$, this reduces to

$$\mathsf{Triangle}(y| -1, 1) = 1 - |y|.$$

The file `example-models/basic_distributions/triangle.stan` contains the following Stan implementation of a sampler from $\mathsf{Triangle}(-1, 1)$.

```
parameters {
  real<lower=-1,upper=1> y;
}
model {
  increment_log_prob(log1m(fabs(y)));
}
```

The single scalar parameter `y` is declared as lying in the interval `(-1,1)`. The total log probability is incremented with the joint log probability of all parameters, i.e., $\log \mathsf{Triangle}(y| -1, 1)$. This value is coded in Stan as `log1m(fabs(y))`. The function `log1m` is is defined so that `log1m(x)` has the same value as `log(1.0-x)`, but the computation is faster, more accurate, and more stable.

The constrained type `real<lower=-1,upper=1>` declared for `y` is critical for correct sampling behavior. If the constraint on `y` is removed from the program, say by declaring `y` as having the unconstrained scalar type `real`, the program would compile, but it would produce arithmetic exceptions at run time when the sampler explored values of `y` outside of $(-1, 1)$.

Now suppose the log probability function were extended to all of $\mathbb{R}$ as follows by defining the probability to be `log(0.0)`, i.e., $-\infty$, for values outside of $(-1, 1)$.

```
increment_log_prob(log(fmax(0.0,1 - fabs(y))));
```

With the constraint on y in place, this is just a less efficient, slower, and less arithmetically stable version of the original program. But if the constraint on y is removed, the model will compile and run without arithmetic errors, but will not sample properly.[1]

Exponential Distribution

If Stan didn't happen to include the exponential distribution, it could be coded directly using the following assignment statement, where lambda is the inverse scale and y the sampled variate.

```
increment_log_prob(log(lambda) - y * lambda);
```

This encoding will work for any lambda and y; they can be parameters, data, or one of each, or even local variables.

The assignment statement in the previous paragraph generates C++ code that is very similar to that generated by the following sampling statement.

```
y ~ exponential(lambda);
```

There are two notable differences. First, the sampling statement will check the inputs to make sure both lambda is positive and y is non-negative (which includes checking that neither is the special not-a-number value).

The second difference is that if lambda is not a parameter, transformed parameter, or local model variable, the sampling statement is clever enough to drop the log(lambda) term. This results in the same posterior because Stan only needs the log probability up to an additive constant. If lambda and y are both constants, the sampling statement will drop both terms (but still check for out-of-domain errors on the inputs).

[1]The problem is the (extremely!) light tails of the triangle distribution. The standard HMC and NUTS samplers can't get into the corners of the triangle properly. Because the Stan code declares y to be of type real<lower=-1,upper=1>, the inverse logit transform is applied to the unconstrained variable and its log absolute derivative added to the log probability. The resulting distribution on the logit-transformed y is well behaved. See Chapter 53 for more information on the transforms used by Stan.

16. User-Defined Functions

This chapter explains functions from a user perspective with examples; see Chapter 25 for the full specification. User-defined functions allow computations to be encapsulated into a single named unit and invoked elsewhere by name. Similarly, functions allow complex procedures to be broken down into more understandable components. Writing modular code using descriptively named functions is easier to understand than a monolithic program, even if the latter is heavily commented.[1]

16.1. Basic Functions

Here's an example of a skeletal Stan program with a user-defined relative difference function employed in the generated quantities block to compute a relative differences between two parameters.

```
functions {
  real relative_diff(real x, real y) {
    real abs_diff;
    real avg_scale;
    abs_diff <- fabs(x - y);
    avg_scale <- (fabs(x) + fabs(y)) / 2;
    return abs_diff / avg_scale;
  }
}
...
generated quantities {
  real rdiff;
  rdiff <- relative_diff(alpha,beta);
}
```

The function is named `relative_diff`, and is declared to have two real-valued arguments and return a real-valued result. It is used the same way a built-in function would be used in the generated quantities block.

User-Defined Functions Block

All functions are defined in their own block, which is labeled `functions` and must appear before all other program blocks. The user-defined functions block is optional.

[1]The main problem with comments is that they can be misleading, either due to misunderstandings on the programmer's part or because the program's behavior is modified after the comment is written. The program always behaves the way the code is written, which is why refactoring complex code into understandable units is preferable to simply adding comments.

Function Bodies

The body (the part between the curly braces) contains ordinary Stan code, including local variables. The new function is used in the generated quantities block just as any of Stan's built-in functions would be used.

Return Statements

Return statements, such as the one on the last line of the definition of `relative_diff` above, are only allowed in the bodies of function definitions. Return statements may appear anywhere in a function, but functions with non-void return types must end in a return statement; see Section 25.7 for details on how this is enforced.

Reject Statements

The Stan reject statement provides a mechanism to report errors or problematic values encountered during program execution. It accepts any number of quoted string literals or Stan expressions as arguments. This statement is typically embedded in a conditional statement in order to detect bad or illegal outcomes of some processing step.

To illustrate the use of this statement, we modify the user-defined relative difference function example from Section Section 16.1 so that rejection happens if the relative difference is less than some limit.

```
functions {
  real relative_diff(real x, real y, real min) {
    real abs_diff;
    real avg_scale;
    abs_diff <- fabs(x - y);
    avg_scale <- (fabs(x) + fabs(y)) / 2;
    if (abs_diff / avg_scale < min)
      reject("relative_diff below ",min);
    return abs_diff / avg_scale;
  }
}
```

The effect of rejection depends on the program block in which the function was invoked. See Section Section 24.9 for details.

Type Declarations for Functions

Function argument and return types are not declared with their sizes. They also may not contain any constraints; see Figure 16.1 for a list.

Functions: Undimensioned	*Locals Variables: Unconstrained*	*Nonlocal Variables: Constrained*
int	int	int<lower=L><br>int<upper=U><br>int<lower=L,upper=U>
real	real	real<lower=L><br>real<upper=U><br>real<lower=L,upper=U>
vector	vector[N]	vector<lower=L>[N]<br>vector<upper=U>[N]<br>vector<lower=L,upper=U>[N]<br><br>simplex[N]<br>ordered[N]<br>positive_ordered[N]<br>unit_vector[N]
row_vector	row_vector[M]	row_vector<lower=L>[M]<br>row_vector<upper=U>[M]<br>row_vector<lower=L,upper=U>[M]
matrix	matrix[M,N]	matrix<lower=L>[M,N]<br>matrix<upper=U>[M,N]<br>matrix<lower=L,upper=U>[M,N]<br><br>cov_matrix[K]<br>corr_matrix[K]<br>cholesky_factor_cov[K]<br>cholesky_factor_corr[K]

Figure 16.1: *The leftmost column is a list of the unconstrained and undimensioned basic types; these are used as function return types and argument types. The middle column is of unconstrained types with dimensions; these are used as local variable types. The rightmost column lists the corresponding constrained types. An expression of any righthand column type may be assigned to its corresponding lefthand column basic type. At runtime, dimensions are checked for consistency for all variables; containers of any sizes may be assigned to function arguments. The constrained matrix types* cov_matrix[K], corr_matrix[K], cholesky_factor_cov[K], *and* cholesky_factor_corr[K] *are only assignable to matrices of dimensions* matrix[K,K] *types. Stan also allows arrays of any of these types, with slightly different declarations for function arguments and return types and variables.*

Unlike type declarations for variables, function type declarations for matrix and vector types are not declared with their sizes. Like local variable declarations, function argument type declarations may not be declared with constraints (either lower or upper bounds or structured constraints like forming a simplex or correlation matrix).

For example, here's a function to compute the entropy of a categorical distribution with simplex parameter `theta`.

```
real entropy(vector theta) {
  return sum(theta .* log(theta));
}
```

Although `theta` must be a simplex, only the type `vector` is used.[2] Upper or lower bounds on values or constrained types are not allowed as return types or argument types in function declarations.

Array Types for Function Declarations

Array arguments have their own syntax, which follows that used in this manual for function signatures. For example, a function that operates on a two-dimensional array to produce a one-dimensional array might be declared as follows.

```
real[] baz(real[,] x);
```

The notation `[]` is used for one-dimensional arrays (as in the return above), `[,]` for two-dimensional arrays, `[,,]` for three-dimensional arrays, and so on.

Functions support arrays of any type, including matrix and vector types. As with other types, no constraints are allowed.

16.2. Functions as Statements

In some cases, it makes sense to have functions that do not return a value. For example, a routine to print the lower-triangular portion of a matrix can be defined as follows.

```
functions {
  void pretty_print_tri_lower(matrix x) {
    if (rows(x) == 0) {
      print("empty matrix");
      return;
    }
```

[2] A range of built-in validation routines is coming to Stan soon! Alternatively, the `reject` statement can be used to check constraints on the simplex.

```
    print("rows=", rows(x), " cols=", cols(x));
    for (m in 1:rows(x))
      for (n in 1:m)
        print("[", m, ",", n, "]=", x[m,n]);
  }
}
```

The special symbol `void` is used as the return type. This is not a type itself in that there are no values of type `void`; it merely indicates the lack of a value. As such, return statements for void functions are not allowed to have arguments, as in the return statement in the body of the previous example.

Void functions applied to appropriately typed arguments may be used on their own as statements. For example, the pretty-print function defined above may be applied to a covariance matrix being defined in the transformed parameters block.

```
transformed parameters {
  cov_matrix[K] Sigma;
  ... code to set Sigma ...
  pretty_print_tri_lower(Sigma);
  ...
```

16.3. Functions Accessing the Log Probability Accumulator

Functions whose names end in `_lp` are allowed to use sampling statements and `increment_log_prob()` statements; other functions are not. Because of this access, their use is restricted to the transformed parameters and model blocks.

Here is an example of a function to return the centered coefficients derived from a non-centered vector of parameters `beta_raw` along with the location `mu` of the center and the scale `sigma`; see Section 19.1 for more information on centering.

```
functions {
  vector center_lp(vector beta_raw, real mu, real sigma) {
    beta_raw ~ normal(0,1);
    sigma ~ cauchy(0,5);
    mu ~ cauchy(0,2.5);
    return sigma * beta_raw + mu;
  }
  ...
}
parameters {
  vector[K] beta_raw;
```

```
  real mu_beta;
  real<lower=0> sigma_beta;
  ...
transformed parameters {
  vector[K] beta;
  ...
  beta <- center_lp(beta_raw, mu_beta, sigma_beta);
  ...
```

16.4. Functions Acting as Random Number Generators

A user-specified function can be declared to act as a (pseudo) random number generator (PRNG) by giving it a name that ends in `_rng`. Giving a function a name that ends in `_rng` allows it to access built-in functions and user-defined functions that end in `_rng`, whihc includes all the built-in PRNG functions. Only functions ending in `_rng` are able access the built-in PRNG functions. The use of functions ending in `_rng` must therefore be restricted to the generated quantities block like other PRNG functions.

For example, the following function generates an $N \times K$ data matrix, the first column of which is filled with 1 values for the intercept and the remaining entries of which have values drawn from a unit normal PRNG.

```
matrix predictors_rng(int N, int K) {
  matrix[N,K] x;
  for (n in 1:N) {
    x[n,1] <- 1.0;  // intercept
    for (k in 2:K)
      x[n,k] <- normal_rng(0,1);
  }
  return x;
}
```

The following function defines a simulator for regression outcomes based on a data matrix `x`, coefficients `beta`, and noise scale `sigma`.

```
vector regression_rng(vector beta, matrix x, real sigma) {
  vector[rows(x)] y;
  vector[rows(x)] mu;
  mu <- x * beta;
  for (n in 1:rows(x))
    y[n] <- normal_rng(mu[n], sigma);
  return y;
}
```

These might be used in a generated quantity block to simulate some fake data from a fitted regression model as follows.

```
parameters {
  vector[K] beta;
  real<lower=0> sigma;
  ...
generated quantities {
  matrix[N_sim,K] x_sim;
  vector[N_sim] y_sim;
  x_sim <- predictors_rng(N_sim,K);
  y_sim <- regression_rng(beta,x_sim,sigma);
}
```

A more sophisticated simulation might fit a multivariate normal to the predictors `x` and use the resulting parameters to generate multivariate normal draws for `x_sim`.

16.5. User-Defined Probability Functions

Probability functions are distinguished in Stan by names ending in `_log` and `real` return types.

Suppose a model uses several unit normal distributions, for which there is not a specific overloaded density nor defaults in Stan. So rather than writing out the location of 0 and scale of 1 for all of them, a new density function may be defined and reused.

```
functions {
  real unit_normal_log(real y) {
    return normal_log(y,0,1);
  }
}
...
model {
  alpha ~ unit_normal();
  beta ~ unit_normal();
  ...
}
```

The ability to use the `unit_normal` function as a density is keyed off its name ending in `_log`.

In general, if `foo_log` is defined to consume $N + 1$ arguments, then

```
y ~ foo(theta1,...,thetaN);
```

can be used as shorthand for

```
increment_log_prob(foo_log(y,theta1,...,thetaN));
```

As with the built-in functions, the suffix _log is dropped and the first argument moves to the left of the sampling symbol (~) in the sampling statement.

16.6. Overloading Functions

Stan permits overloading (i.e., different functions with the same name). For example, the function unit_normal defined above could be named normal, thus overloading with the built-in function of the same name.

For example, built-in functions might be written to convert sequences of scalar arguments to vectors, leading to the following overloading of the name as_vector in four distinct functions.

```
vector as_vector();
vector as_vector(real a);
vector as_vector(real a, real b);
vector as_vector(real a, real b, real c);
```

A function's name combined with its argument type sequence must be unique. Therefore, it is not possible to have two functions of the same name and argument sequence with different return types. It is possible to have two functions of the same name and number of arguments as long as the arguments are of different types. For example, the following signatures are legal together.

```
real bar(matrix m);
real bar(vector v);
real bar(row_vector rv);
real bar(real[] a1);
real bar(real[,] a2);
```

They could be used to define an operation that can apply to any one- or two-dimensional container.

Overloaded functions only share names—their implementations are not intrinsically related. Nevertheless, it is good programming practice to only reuse names for related functions—otherwise, those reading the program will be confused.

16.7. Documenting Functions

Functions will ideally be documented at their interface level. The Stan style guide for function documentation follows the same format as used by the Doxygen (C++) and Javadoc (Java) automatic documentation systems. Such specifications indicate the variables and their types and the return value, prefaced with some descriptive text.

For example, here's some documentation for the prediction matrix generator.

```
/**
 * Return a data matrix of specified size with rows
 * correspdonding to items and the first column filled
 * with the value 1 to represent the intercept and the
 * remaining columns randomly filled with unit-normal draws.
 *
 * @param N Number of rows correspdong to data items
 * @param K Number of predictors, counting the intercept, per
 *          item.
 * @return Simulated predictor matrix.
 */
matrix predictors_rng(int N, int K) {
  ...
```

The comment begins with `/**`, ends with `*/`, and has an asterisk (`*`) on each line. It uses `@param` followed by the argument's identifier to document a function argument. The tag `@return` is used to indicate the return value. Stan does not (yet) have an automatic documentation generator like Javadoc or Doxygen, so this just looks like a big comment starting with `/*` and ending with `*/` to the Stan parser.

For functions that raise exceptions, exceptions can be documented using `@throws`.[3] For example,

```
 ...
 * @param theta
 * @throws If any of the entries of theta is negative.
 */
real entropy(vector theta) {
  ...
```

Usually an exception type would be provided, but these are not exposed as part of the Stan language, so there is no need to document them.

[3] As of Stan 2.5.0, the only way a user-defined producer will raise an exception is if a function it calls (including sampling statements) raises an exception via the reject statement.

16.8. Summary of Function Types

Functions may have a void or non-void return type and they may or may not have one of the special suffixes, `_log`, `_lp`, or `_rng`.

Void vs. Non-Void Return

Only functions declared to return `void` may be used as statements. These are also the only functions that use `return` statements with no arguments.

Only functions declared to return non-`void` values may be used as expressions. These functions require `return` statements with arguments of a type that matches the declared return type.

Suffixed or Non-Suffixed

Only functions ending in `_log` and with return type `real` may be used as probability functions in sampling statements.

Only functions ending in `_lp` may access the log probability accumulator through sampling statements or `increment_log_prob()` statements. Such functions may only be used in the transformed parameters or model blocks.

Only functions ending in `_rng` may access the built-in pseudo-random number generators. Such functions may only be used in the generated quantities block.

«««< HEAD

16.9. Recursive Functions

Stan supports recursive function definitions, which can be useful for some applications. For instance, consider the matrix power operation, A^n, which is defined for a square matrix A and positive integer n by

$$A^n = \begin{cases} \mathrm{I} & \text{if } n = 0, \text{ and} \\ A\,A^{n-1} & \text{if } n > 0. \end{cases}$$

where I is the identity matrix. This definition can be directly translated to a recursive function definition.

```
matrix matrix_pow(matrix a, int n);

matrix matrix_pow(matrix a, int n) {
  if (n == 0)
    return diag_matrix(rep_vector(1, rows(a)));
```

```
    else
      return a *  matrix_pow(a, n - 1);
  }
```

The forward declaration of the function signature before it is defined is necessary so that the embedded use of `matrix_pow` is well-defined when it is encountered.[4] It would be more efficient to not allow the recursion to go all the way to the base case, adding the following conditional clause.

```
    else if (n == 1)
      return a;
```

[4]A future version of Stan will allow self-recursive functions to skip the forward declaration.

17. Solving Differential Equations

Stan provides a built-in mechanism for solving systems of ordinary different equations (ODEs). The full specification can be found in Section 23.7.

17.1. Simple Harmonic Oscillator Example

This section details the simple harmonic oscillator, which is characterized by the following pair of differential equations.

$$\frac{d}{dt}y_1 = -y_2 \qquad \frac{d}{dt}y_2 = -y_1 - \theta y_2 \tag{17.1}$$

The values of $y = (y_1, y_2)$ are a function of both time (t) and the value of the system parameter (θ.

Solutions Given Initial Conditions

Given a value of the parameter θ and an initial state $y(t_0)$ at time t_0, it is possible to solve the equations numerically to calculate $y(t)$ for any given time t. The rest of this chapter shows how Stan is able to provide the functionalty to specify and solve a system of ODEs.

17.2. Coding an ODE System

A system of ODEs is coded directly in Stan as a function. For example, the simple harmonic oscillator given in Equation (17.1), can be coded using the following function in Stan (see Chapter 16 for more information on coding user-defined functions).

```
real[] sho(real t,
           real[] y,
           real[] theta,
           real[] x_r,
           int[] x_i) {
  real dydt[2];
  dydt[1] <- y[2];
  dydt[2] <- -y[1] - theta[1] * y[2];
  return dydt;
}
```

The function takes in a time `t`, system state `y`, system parameters `theta`, along with real data in variable `x_r` and integer data in variable `x_i`. It returns an array of derivatives of the system state with respect to time, evaluated at time `t` and state `y`.

Strict Signature

All of the arguments to such a function must be provided in exactly the order shown above. This may require passing in zero-length arrays for data or parameters if the system does not involve data or parameters. A full example for the simple harmonic oscillator, which does not depend on any constant data variables, is provided in in Figure 17.1.

Data versus Parameters

Unlike other functions, a function describing a system of ODEs is limited as to the origins of variables its arguments. In particular, the time `t`, real data `x`, and integer data `x_int` must be expressions that only involve data or transformed data variables. The initial state `y` or the parameters `theta` are the only arguments which may involve parameters.

17.3. Measurement Error Models

The point of doing statistical estimation for differential equations is to estimate the parameters and/or initial state given noisy measurements of the system variables at a finite number of time points.

For instance, suppose the simple harmonic oscillator has a parameter value of $\theta = 0.15$ and initial state $y(t = 0) = (1, 0)$. Now suppose the system is observed at 10 time points, say $t = 1, 2, ..., 10$, where each measurement of $y(t)$ has independent $\mathsf{Normal}(0, 0.1)$ error in both dimensions ($y_1(t)$ and $y_2(t)$). A plot of such measurements is shown in Figure 17.1.

Simulating Noisy Measurements

The data used to make this plot is derived from the Stan model to simulate noisy observations given in Figure 17.2.

This program illustrates the way in which the ODE solver is called in a Stan program,

```
y_hat <- integrate_ode(sho, y0, t0, ts, theta, x_r, x_i);
```

This assigns the solutions to the system defined by function `sho`, given initial state `y0`, initial time `t0`, requested solution times `ts`, parameters `theta`, real data `x`, and integer data `x_int`.

Here, the ODE solver is called in the generated quantities block to provide a 10×2 array of solutions `y_hat` to which measurement error is added using the normal

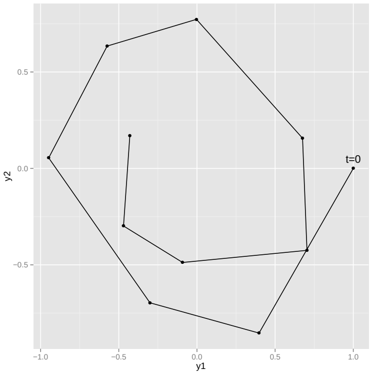

Figure 17.1: *Trajectory of the simple harmonic oscillator given parameter* $\theta = 0.15$ *and initial condition* $y(t = 0) = (1, 0)$ *with additional independent* $\mathsf{Normal}(0, 0.1)$ *measurement error in both dimensions.*

pseudo-random number generating function `normal_rng`. The number of rows in the solution array is the same as the size of `ts`, the requested solution times.

Estimating System Parameters and Initial State

Stan provides statistical inference for unknown initial states and/or parameters. The ODE solver will be used deterministically to produce predictions, much like the linear predictor does in a generalized linear model. These states will then be observed with measurement error.

A Stan program that can be used to estimate both the initial state and parameter value for the simple harmonic oscillator given noisy observations is given in Figure 17.3. Compared to the simulation model in Figure 17.2, the model to estimate parameters uses the `integrate_ode` function in the model block rather than the generated quantities block. There are Cauchy priors on the measurement error scales `sigma` and unit normal priors on the components of parameter array `theta` and initial state parameter array `y0`. The solutions to the ODE are then assigned to an array `y_hat`, which is then used as the location in the observation noise model as follows.

```
y_hat <- integrate_ode(sho, y0, t0, ts, theta, x_r, x_i);
for (t in 1:T)
  y[t] ~ normal(y_hat[t], sigma);
```

As with other regression-like models, it's easy to change the noise model to be robust

```
functions {
  real[] sho(real t,
             real[] y,
             real[] theta,
             real[] x_r,
             int[] x_i) {
    real dydt[2];
    dydt[1] <- y[2];
    dydt[2] <- -y[1] - theta[1] * y[2];
    return dydt;
  }
}
data {
  int<lower=1> T;
  real y0[2];
  real t0;
  real ts[T];
  real theta[1];
}
transformed data {
  real x_r[0];
  int x_i[0];
}
model {
}
generated quantities {
  real y_hat[T,2];
  y_hat <- integrate_ode(sho, y0, t0, ts, theta, x_r, x_i);

  // add measurement error
  for (t in 1:T) {
    y_hat[t,1] <- y_hat[t,1] + normal_rng(0,0.1);
    y_hat[t,2] <- y_hat[t,2] + normal_rng(0,0.1);
  }
}
```

Figure 17.2: *Stan program to simulate noisy measurements from a simple harmonic oscillator. The system of differential equations is coded as a function. The system parameters* `theta` *and initial state* `y0` *are read in as data along with the initial time* `t0` *and observation times* `ts`*. The generated quantities block is used to solve the ODE for the specified times and then add random measurement error, producing observations* `y_hat`*.*

```
functions {
  real[] sho(real t,
             real[] y,
             real[] theta,
             real[] x_r,
             int[] x_i) {
    real dydt[2];
    dydt[1] <- y[2];
    dydt[2] <- -y[1] - theta[1] * y[2];
    return dydt;
  }
}
data {
  int<lower=1> T;
  real y[T,2];
  real t0;
  real ts[T];
}
transformed data {
  real x_r[0];
  int x_i[0];
}
parameters {
  real y0[2];
  vector<lower=0>[2] sigma;
  real theta[1];
}
model {
  real y_hat[T,2];
  sigma ~ cauchy(0,2.5);
  theta ~ normal(0,1);
  y0 ~ normal(0,1);
  y_hat <- integrate_ode(sho, y0, t0, ts, theta, x_r, x_i);
  for (t in 1:T)
    y[t] ~ normal(y_hat[t], sigma);
}
```

Figure 17.3: *Stan program to estimate unknown initial conditions* `y0` *and system parameter* `theta` *for the simple harmonic oscillator with independent normal measurement error.*

(e.g., Student-t distributed), to be correlated in the state variables (e.g., with a multivariate normal distribution), or both (e.g., with a multivariate Student-t distribution).

In this simple model with independent noise scales of 0.10, 10 observed data points for times $t = 1, \ldots, 10$ is sufficient to reliably estimate the ODE parameter, initial state, and noise scales.

17.4. Stiff ODEs

Stan's approach will not be efficient for stiff systems of ODEs, roughly characterized as systems presenting numerical difficulties for gradient-based stepwise solvers. Stiffness typically arises due to high curvature in the solution space.[1] It will work, but can be slow enough to be impractical because the stepwise integrator used for the solutions will have to take very small steps to ensure adequate solution accuracy. In the future, we hope to roll out a more efficient approach to stiff ODEs in which automatic differentiation can be applied to compute the Jacobian of the final coupled system of state variables and partials with respect to parameters.

[1]Not coincidentally, high curvature in the posterior of a general Stan model poses the same kind of problem for Euclidean Hamiltonian Monte Carlo (HMC) sampling. The reason is that HMC is based on the leapfrog algorithm, a gradient-based, stepwise numerical differential equation solver specialized for Hamiltonian systems with separable potential and kinetic energy terms.

18. Problematic Posteriors

Mathematically speaking, with a proper posterior, one can do Bayesian inference and that's that. There is not even a need to require a finite variance or even a finite mean—all that's needed is a finite integral. Nevertheless, modeling is a tricky business and even experienced modelers sometimes code models that lead to improper priors. Furthermore, some posteriors are mathematically sound, but ill-behaved in practice. This chapter discusses issues in models that create problematic posterior inferences, either in general for Bayesian inference or in practice for Stan.

18.1. Collinearity of Predictors in Regressions

This section discusses problems related to the classical notion of identifiability, which lead to ridges in the posterior density and wreak havoc with both sampling and inference.

Examples of Collinearity

Redundant Intercepts

The first example of collinearity is an artificial example involving redundant intercept parameters.[1] Suppose there are observations y_n for $n \in 1{:}N$, two intercept parameters λ_1 and λ_2, a scale parameter $\sigma > 0$, and the sampling distribution

$$y_n \sim \mathsf{Normal}(\lambda_1 + \lambda_2, \sigma).$$

For any constant q, the sampling density for y does not change if we add q to λ_1 and subtract it from λ_2, i.e.,

$$p(y|\lambda_1, \lambda_2, \sigma) = p(y|\lambda_1 + q, \lambda_2 - q, \sigma).$$

The consequence is that an improper uniform prior $p(\mu, \sigma) \propto 1$ leads to an improper posterior. This impropriety arises because the neighborhoods around $\lambda_1 + q, \lambda_1 - q$ have the same mass no matter what q is. Therefore, a sampler would need to spend as much time in the neighborhood of $\lambda_1 = 1000000000$ and $\lambda_2 = -1000000000$ as it does in the neighborhood of $\lambda_1 = 0$ and $\lambda_2 = 0$, and so on for ever more far-ranging values.

[1]This example was raised by Richard McElreath on the Stan users group in a query about the difference in behavior between Gibbs sampling as used in BUGS and JAGS and the Hamiltonian Monte Carlo (HMC) and no-U-turn samplers (NUTS) used by Stan.

The maringal posterior $p(\lambda_1, \lambda_2|y)$ for this model is thus improper.[2] The impropriety shows up visually as a ridge in the posterior density, as illustrated in the left-hand figure of Figure 18.1. The ridge for this model is along the line where $\lambda_2 = \lambda_1 + c$ for some constant c.

Contrast this model with a simple regression with a single intercept parameter μ and sampling distribution

$$y_n \sim \mathsf{Normal}(\mu, \sigma).$$

Even with an improper prior, the posterior is proper as long as there are at least two data points y_n with distinct values.

Ability and Difficulty in IRT Models

Consider an item-response theory model for students $j \in 1{:}J$ with abilities α_j and test items $i \in 1{:}I$ with difficulties β_i. The observed data is an $I \times J$ array with entries $y_{i,j} \in \{0, 1\}$ coded such that $y_{i,j} = 1$ indicates that student j answered question i correctly. The sampling distribution for the data is

$$y_{i,j} \sim \mathsf{Bernoulli}(\operatorname{logit}^{-1}(\alpha_j - \beta_i)).$$

For any constant c, the probability of y is unchanged by adding a constant c to all the abilities and subtracting it from all the difficulties, i.e.,

$$p(y|\alpha, \beta) = p(y|\alpha + c, \beta - c).$$

This leads to a multivariate version of the ridge displayed by the regression with two intercepts discussed above.

General Collinear Regression Predictors

The general form of the collinearity problem arises when predictors for a regression are collinear. For example, consider a linear regression sampling distribution

$$y_n \sim \mathsf{Normal}(x_n\beta, \sigma)$$

for an N-dimensional observation vector y, an $N \times K$ predictor matrix x, and a K-dimensional coefficient vector β.

Now suppose that column k of the predictor matrix is a multiple of column k', i.e., there is some constant c such that $x_{n,k} = c\, x_{n,k'}$ for all n. In this case, the coefficients β_k and $\beta_{k'}$ can covary without changing the predictions, so that for any $d \neq 0$,

$$p(y| \ldots, \beta_k, \ldots, \beta_{k'}, \ldots, \sigma) = p(y| \ldots, d\beta_k, \ldots, \frac{d}{c}\, \beta_{k'}, \ldots, \sigma).$$

Even if columns of the predictor matrix are not exactly collinear as discussed above, they cause similar problems for inference if they are nearly collinear.

[2] The marginal posterior $p(\sigma|y)$ for σ is proper here as long as there are at least two distinct data points.

Multiplicative Issues with Discrimination in IRT

Consider adding a discrimination parameter δ_i for each question in an IRT model, with data sampling model

$$y_{i,j} \sim \mathsf{Bernoulli}(\mathrm{logit}^{-1}(\delta_i(\alpha_j - \beta_i))).$$

For any constant $c \neq 0$, multiplying δ by c and dividing α and β by c produces the same likelihood,

$$p(y|\delta, \alpha, \beta) = p(y|c\delta, \frac{1}{c}\alpha, \frac{1}{c}\beta).$$

If $c < 0$, this switches the signs of every component in α, β, and δ without changing the density.

Softmax with K vs. $K - 1$ Parameters

In order to parameterize a K-simplex (i.e., a K-vector with non-negative values that sum to one), only $K - 1$ parameters are necessary because the Kth is just one minus the sum of the first $K - 1$ parameters, so that if θ is a K-simplex,

$$\theta_K = 1 - \sum_{k=1}^{K} \theta_k.$$

The softmax function (see Section 33.6) maps a K-vector α of linear predictors to a K-simplex $\theta = \mathrm{softmax}(\alpha)$ by defining

$$\theta_k = \frac{\exp(\alpha_k)}{\sum_{k'=1}^{K} \exp(\alpha'_k)}.$$

The softmax function is many-to-one, which leads to a lack of identifiability of the unconstrained parameters α. In particular, adding or subtracting a constant from each α_k produces the same simplex θ.

Mitigating the Invariances

All of the examples discussed in the previous section allow translation or scaling of parameters while leaving the data probability density invariant. These problems can be mitigated in several ways.

Removing Redundant Parameters or Predictors

In the case of the multiple intercepts, λ_1 and λ_2, the simplest solution is to remove the redundant intercept, resulting in a model with a single intercept parameter μ and sampling distribution $y_n \sim \mathsf{Normal}(\mu, \sigma)$. The same solution works for solving the problem with collinearity—just remove one of the columns of the predictor matrix x.

Pinning Parameters

The IRT model without a discrimination parameter can be fixed by pinning one of its parameters to a fixed value, typically 0. For example, the first student ability α_1 can be fixed to 0. Now all other student ability parameters can be interpreted as being relative to student 1. Similarly, the difficulty parameters are interpretable relative to student 1's ability to answer them.

This solution is not sufficient to deal with the multiplicative invariance introduced by the question discrimination parameters δ_i. To solve this problem, one of the difficulty parameters, say δ_1, must also be constrained. Because it's a multiplicative and not an additive invariance, it must be constrained to a non-zero value, with 1 being a convenient choice. Now all of the discrimination parameters may be interpreted relative to item 1's discrimination.

The many-to-one nature of softmax(α) is typically mitigated by pinning a component of α, for instance fixing $\alpha_K = 0$. The resulting mapping is one-to-one from $K - 1$ unconstrained parameters to a K-simplex. This is roughly how simplex-constrained parameters are defined in Stan; see Section 53.6 for a precise definition. The Stan code for creating a simplex from a $K - 1$-vector can be written as

```
vector softmax_id(vector alpha) {
  vector[num_elements(alpha) + 1] alphac1;
  for (k in 1:num_elements(alpha))
    alphac1[k] <- alpha[k];
  alpha[num_elements(alphac)] <- 0;
  return softmax(alphac);
}
```

Adding Priors

So far, the models have been discussed as if the priors on the parameters were improper uniform priors.

A more general Bayesian solution to these invariance problems is to impose proper priors on the parameters. This approach can be used to solve problems arising from either additive or multiplicative invariance.

For example, normal priors on the multiple intercepts,

$$\lambda_1, \lambda_2 \sim \mathsf{Normal}(0, \tau),$$

with a constant scale τ, ensure that the posterior mode is located at a point where $\lambda_1 = \lambda_2$, because this minimizes $\log \mathsf{Normal}(\lambda_1|0,\tau) + \log \mathsf{Normal}(\lambda_2|0,\tau)$.[3] The addi-

[3]A Laplace prior (or an L1 regularizer for penalized maximum likelihood estimation) is not sufficient to remove this additive invariance. It provides shrinkage, but does not in and of itself identify the parameters because adding a constant to λ_1 and subtracting it from λ_2 results in the same value for the prior density.

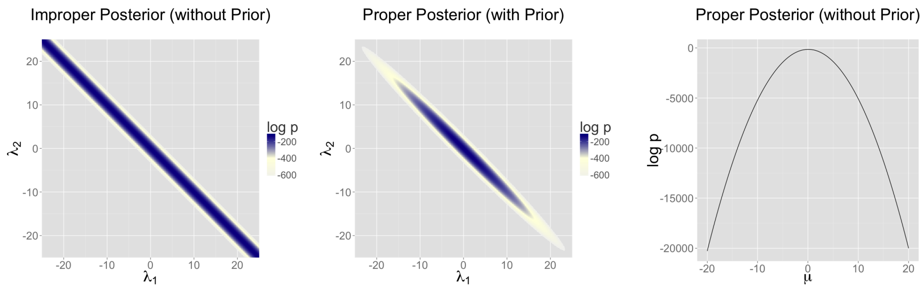

Figure 18.1: *Posteriors for two intercept parameterization without prior, two intercept parameterization with unit normal prior, and one intercept reparameterization without prior. For all three cases, the posterior is plotted for 100 data points drawn from a unit normal. Left) The two intercept parameterization leads to an improper prior with a ridge extending infinitely to the northwest and southeast. Middle) Adding a unit normal prior for the intercepts results in a proper posterior. Right) The single intercept parameterization with no prior also has a proper posterior.*

tion of a prior to the two intercepts model is shown in the middle plot in Figure 18.1. The plot on the right of Figure 18.1 shows the result of reparameterizing to a single intercept.

An alternative strategy for identifying a K-simplex parameterization $\theta = \text{softmax}(\alpha)$ in terms of an unconstrained K-vector α is to place a prior on the components of α with a fixed location (that is, specifically avoid hierarchical priors with varying location). Unlike the approaching of pinning $\alpha_K = 0$, the prior-based approach models the K outcomes symmetrically rather than modeling $K - 1$ outcomes relative to the K-th. The pinned parameterization, on the other hand, is usually more efficient statisticaly because it does not have the extra degree of (prior constrained) wiggle room.

Vague, Strongly Informative, and Weakly Informative Priors

Care must be used when adding a prior to resolve invariances. If the prior is taken to be too broad (i.e., too vague), the resolution is in theory only, and samplers will still struggle.

Ideally, a realistic prior will be formulated based on substantive knowledge of the problem being modeled. Such a prior can be chosen to have the appropriate strength based on prior knowledge. A strongly informative prior makes sense if there is strong prior information.

When there is not strong prior information, a weakly informative prior strikes the proper balance between controlling computational inference without dominating the data in the posterior. In most problems, the modeler will have at least some notion

of the expected scale of the estimates and be able to choose a prior for identification purposes that does not dominate the data, but provides sufficient computational control on the posterior.

Priors can also be used in the same way to control the additive invariance of the IRT model. A typical approach is to place a strong prior on student ability parameters α to control scale simply to control the additive invariance of the basic IRT model and the multiplicative invariance of the model extended with a item discrimination parameters; such a prior does not add any prior knowledge to the problem. Then a prior on item difficulty can be chosen that is either informative or weakly informative based on prior knowledge of the problem.

18.2. Label Switching in Mixture Models

Where collinearity in regression models can lead to infinitely many posterior maxima, swapping components in a mixture model leads to finitely many posterior maxima.

Mixture Models

Consider a normal mixture model with two location parameters μ_1 and μ_2, a shared scale $\sigma > 0$, a mixture ratio $\theta \in [0,1]$, and likelihood

$$p(y|\theta,\mu_1,\mu_2,\sigma) = \prod_{n=1}^{N} \big(\theta\,\mathsf{Normal}(y_n|\mu_1,\sigma) + (1-\theta)\,\mathsf{Normal}(y_n|\mu_2,\sigma)\big).$$

The issue here is exchangeability of the mixture components, because

$$p(\theta,\mu_1,\mu_2,\sigma|y) = p((1-\theta),\mu_2,\mu_1,\sigma|y).$$

The problem is exacerbated as the number of mixture components K grows, as in clustering models, leading to $K!$ identical posterior maxima.

Convergence Monitoring and Effective Sample Size

The analysis of posterior convergence and effective sample size is also difficult for mixture models. For example, the $\hat{R}$ convergence statistic reported by Stan and the computation of effective sample size are both compromised by label switching. The problem is that the posterior mean, a key ingredient in these computations, is affected by label switching, resulting in a posterior mean for μ_1 that is equal to that of μ_2, and a posterior mean for θ that is always 1/2, no matter what the data is.

Some Inferences are Invariant

In some sense, the index (or label) of a mixture component is irrelevant. Posterior predictive inferences can still be carried out without identifying mixture components. For example, the log probability of a new observation does not depend on the identities of the mixture components. The only sound Bayesian inferences in such models are those that are invariant to label switching. Posterior means for the parameters are meaningless because they are not invariant to label switching; for example, the posterior mean for θ in the two component mixture model will always be $1/2$.

Highly Multimodal Posteriors

Theoretically, this should not present a problem for inference because all of the integrals involved in posterior predictive inference will be well behaved. The problem in practice is computation.

Being able to carry out such invariant inferences in practice is an altogether different matter. It is almost always intractable to find even a single posterior mode, much less balance the exploration of the neighborhoods of multiple local maxima according to the probability masses. In Gibbs sampling, it is unlikely for μ_1 to move to a new mode when sampled conditioned on the current values of μ_2 and θ. For HMC and NUTS, the problem is that the sampler gets stuck in one of the two "bowls" arounds the modes and cannot gather enough energy from random momentum assignment to move from one mode to another.

Even with a proper posterior, all known sampling and inference techniques are notoriously ineffective when the number of modes grows super-exponentially as it does for mixture models with increasing numbers of components.

Hacks as Fixes

Several hacks (i.e., "tricks") have been suggested and employed to deal with the problems posed by label switching in practice.

Parameter Ordering Constraints

One common strategy is to impose a constraint on the parameters that identifies the components. For instance, we might consider constraining $\mu_1 < \mu_2$ in the two-component normal mixture model discussed above. A problem that can arise from such an approach is when there is substantial probabilty mass for the opposite ordering $\mu_1 > \mu_2$. In these cases, the the posteriors are affected by the constraint and true posterior uncertainty in μ_1 and μ_2 is not captured by the model with the constraint. In addition, standard approaches to posterior inference for event probabili-

ties is compromised. For instance, attempting to use M posterior samples to estimate $\mathrm{Pr}[\mu_1 > \mu_2]$, will fail, because the estimator

$$\mathrm{Pr}[\mu_1 > \mu_2] \approx \sum_{m=1}^{M} \mathrm{I}(\mu_1^{(m)} > \mu_2^{(m)})$$

will result in an estimate of 0 because the posterior respects the constraint in the model.

Initialization around a Single Mode

Another common approach is to run a single chain or to initialize the parameters near realistic values.[4] This can work better than the hard constraint approach if reasonable initial values can be found and the labels do not switch within a Markov chain. The result is that all chains are glued to a neighborhood of a particular mode in the posterior.

18.3. Posteriors with Unbounded Densities

In some cases, the posterior density grows without bounds as parameters approach certain poles or boundaries. In such, there are no posterior modes and numerical stability issues can arise as sampled parameters approach constraint boundaries.

Mixture Models with Varying Scales

One such example is a binary mixture model with scales varying by component, σ_1 and σ_2 for locations μ_1 and μ_2. In this situation, the density grows without bound as $\sigma_1 \rightarrow 0$ and $\mu_1 \rightarrow y_n$ for some n; that is, one of the mixture components concentrates all of its mass around a single data item y_n.

Beta-Binomial Models with Skewed Data and Weak Priors

Another example of unbounded densities arises with a posterior such as $\mathsf{Beta}(\phi|0.5, 0.5)$, which can arise if very "weak" beta priors are used for groups that have no data. This density is unbounded as $\phi \rightarrow 0$ and $\phi \rightarrow 1$. Similarly, a Bernoulli likelihood model coupled with a "weak" beta prior, leads to a posterior

$$\begin{array}{rcl} p(\phi|y) & \propto & \mathsf{Beta}(\phi|0.5, 0.5) \times \prod_{n=1}^{N} \mathsf{Bernoulli}(y_n|\phi) \\ & = & \mathsf{Beta}(\phi \mid 0.5 + \sum_{n=1}^{N} y_n, \;\; 0.5 + N - \sum_{n=1}^{N} y_n). \end{array}$$

[4] Tempering methods may be viewed as automated ways to carry out such a search for modes, though most MCMC tempering methods continue to search for modes on an ongoing basis; see (Swendsen and Wang, 1986; Neal, 1996b).

If $N = 9$ and each $y_n = 1$, the posterior is $\text{Beta}(\phi|9.5, 0, 5)$. This posterior is unbounded as $\phi \rightarrow 1$. Nevertheless, the posterior is proper, and although there is no posterior mode, the posterior mean is well-defined with a value of exactly 0.95.

Constrained vs. Unconstrained Scales

Stan does not sample directly on the constrained $(0, 1)$ space for this problem, so it doesn't directly deal with unconstrained density values. Rather, the probability values ϕ are logit-transformed to $(-\infty, \infty)$. The boundaries at 0 and 1 are pushed out to $-\infty$ and ∞ respectively. The Jacobian adjustment that Stan automatically applies ensures the unconstrained density is proper. The adjustment for the particular case of $(0, 1)$ is $\log \text{logit}^{-1}(\phi) + \log \text{logit}(1 - \phi)$; see Section 53.4 for the derivation.

There are two problems that still arise, though. The first is that if the posterior mass for ϕ is near one of the boundaries, the logit-transformed parameter will have to sweep out very long paths and thus can dominate the U-turn condition imposed by the no-U-turn sampler (NUTS). The second issue is that the inverse transform from the unconstrained space to the constrained space can underflow to 0 or overflow to 1, even when the unconstrained parameter is not infinite. Similar problems arise for the expectation terms in logistic regression, which is why the logit-scale parameterizations of the Bernoulli and binomial distributions are more stable.

18.4. Posteriors with Unbounded Parameters

In some cases, the posterior density will not grow without bound, but parameters will grow without bound with gradually increasing density values. Like the models discussed in the previous section that have densities that grow without bound, such models also have no posterior modes.

Separability in Logistic Regression

Consider a logistic regression model with N observed outcomes $y_n \in \{0, 1\}$, an $N \times K$ matrix x of predictors, a K-dimensional coefficient vector β, and sampling distribution

$$y_n \sim \text{Bernoulli}(\text{logit}^{-1}(x_n \beta)).$$

Now suppose that column k of the predictor matrix is such that $x_{n,k} > 0$ if and only if $y_n = 1$, a condition known as "separability." In this case, predictive accuracy on the observed data continue to improve as $\beta_k \rightarrow \infty$, because for cases with $y_n = 1$, $x_n \beta \rightarrow \infty$ and hence $\text{logit}^{-1}(x_n \beta) \rightarrow 1$.

With separability, there is no maximum to the likelihood and hence no maximum likelihood estimate. From the Bayesian perspective, the posterior is improper and

therefor the marginal posterior mean for β_k is also not defined. The usual solution to this problem in Bayesian models is to include a proper prior for β, which ensures a proper posterior.

18.5. Uniform Posteriors

Suppose your model includes a parameter ψ that is defined on $[0, 1]$ and is given a flat prior $\mathsf{Uniform}(\psi|0, 1)$. Now if the data don't tell us anything about ψ, the posterior is also $\mathsf{Uniform}(\psi|0, 1)$.

Although there is no maximum likelihood estimate for ψ, the posterior is uniform over a closed interval and hence proper. In the case of a uniform posterior on $[0, 1]$, the posterior mean for ψ is well-defined with value $1/2$. Although there is no posterior mode, posterior predictive inference may nevertheless do the right thing by simply integrating (i.e., averaging) over the predictions for ψ at all points in $[0, 1]$.

18.6. Sampling Difficulties with Problematic Priors

With an improper posterior, it is theoretically impossible to properly explore the posterior. However, Gibbs sampling as performed by BUGS and JAGS, although still ineable to properly sample from such an improper posterior, behaves quite differently in practice than the Hamiltonian Monte Carlo sampling performed by Stan when faced with an example such as the two intercept model discussed in Section 18.1 and illustrated in Figure 18.1.

Gibbs Sampling

Gibbs sampling, as performed by BUGS and JAGS, may appear to be efficient and well behaved for this unidentified model, but as discussed in the previous subsection, will not actually explore the posterior properly.

Consider what happens with initial values $\lambda_1^{(0)}, \lambda_2^{(0)}$. Gibbs sampling proceeds in iteration m by drawing

$$\begin{array}{rcl}
\lambda_1^{(m)} & \sim & p(\lambda_1 \mid \lambda_2^{(m-1)}, \sigma^{(m-1)}, y) \\
\lambda_2^{(m)} & \sim & p(\lambda_2 \mid \lambda_1^{(m)}, \sigma^{(m-1)}, y) \\
\sigma^{(m)} & \sim & p(\sigma \mid \lambda_1^{(m)}, \lambda_2^{(m)}, y).
\end{array}$$

Now consider the draw for λ_1 (the draw for λ_2 is symmetric), which is conjugate in this model and thus can be done very efficiently. In this model, the range from which the next λ_1 can be drawn is highly constrained by the current values of λ_2 and σ. Gibbs

will run very quickly and provide seemingly reasonable inferences for $\lambda_1 + \lambda_2$. But it will not explore the full range of the posterior; it will merely take a slow random walk from the initial values. This random walk behavior is typical of Gibbs sampling when posteriors are highly correlated and the primary reason to prefer Hamiltonian Monte Carlo to Gibbs sampling for models with parameters correlated in the posterior.

Hamiltonian Monte Carlo Sampling

Hamiltonian Monte Carlo (HMC), as performed by Stan, is much more efficient at exploring posteriors in models where parameters are correlated in the posterior. In this particular example, the Hamiltonian dynamics (i.e., the motion of a fictitious particle given random momentum in the field defined by the negative log posterior) is going to run up and down along the valley defined by the potential energy (ridges in log posteriors correspond to valleys in potential energy). In practice, even with a random momentum for λ_1 and λ_2, the gradient of the log posterior is going to adjust for the correlation and the simulation will run λ_1 and λ_2 in opposite directions along the valley corresponding to the ridge in the posterior log density (see Figure 18.1.

No-U-Turn Sampling

Stan's default no-U-turn sampler (NUTS), is even more efficient at exploring the posterior (see (Hoffman and Gelman, 2011, 2013)). NUTS simulates the motion of the fictitious particle representing the parameter values until it makes a U-turn, it will be defeated in most cases, as it will just move down the potential energy valley indefinitely without making a U-turn. What happens in practice is that the maximum number of leapfrog steps in the simulation will be hit in many of the iterations, causing a very large number of log probability and gradient evaluations (1000 if the max tree depth is set to 10, as in the default). Thus sampling will appear to be very slow. This is indicative of an improper posterior, not a bug in the NUTS algorithm or its implementation. It is simply not possible to sample from an improper posterior! Thus the behavior of HMC in general and NUTS in particular should be reassuring in that it will clearly fail in cases of improper posteriors, resulting in a clean diagnostic of sweeping out very large paths in the posterior.

Examples: Fits in Stan

To illustrate the issues with sampling from non-identified and only weakly identified models, we fit three models with increasing degrees of identification of their parameters. The posteriors for these models is illustrated in Figure 18.1. The first model is the unidentified model with two location parameters and no priors discussed in Section 18.1.

Two Scale Parameters, Improper Prior

```
Inference for Stan model: improper_stan
Warmup took (2.7, 2.6, 2.9, 2.9) seconds, 11 seconds total
Sampling took (3.4, 3.7, 3.6, 3.4) seconds, 14 seconds total
```

	Mean	MCSE	StdDev	5%	95%	N_Eff	N_Eff/s	R_hat
lp__	-5.3e+01	7.0e-02	8.5e-01	-5.5e+01	-5.3e+01	150	11	1.0
n_leapfrog__	1.4e+03	1.7e+01	9.2e+02	3.0e+00	2.0e+03	2987	212	1.0
lambda1	1.3e+03	1.9e+03	2.7e+03	-2.3e+03	6.0e+03	2.1	0.15	5.2
lambda2	-1.3e+03	1.9e+03	2.7e+03	-6.0e+03	2.3e+03	2.1	0.15	5.2
sigma	1.0e+00	8.5e-03	6.2e-02	9.5e-01	1.2e+00	54	3.9	1.1
mu	1.6e-01	1.9e-03	1.0e-01	-8.3e-03	3.3e-01	2966	211	1.0

Two Scale Parameters, Weak Prior

```
Warmup took (0.40, 0.44, 0.40, 0.36) seconds, 1.6 seconds total
Sampling took (0.47, 0.40, 0.47, 0.39) seconds, 1.7 seconds total
```

	Mean	MCSE	StdDev	5%	95%	N_Eff	N_Eff/s	R_hat
lp__	-54	4.9e-02	1.3e+00	-5.7e+01	-53	728	421	1.0
n_leapfrog__	157	2.8e+00	1.5e+02	3.0e+00	511	3085	1784	1.0
lambda1	0.31	2.8e-01	7.1e+00	-1.2e+01	12	638	369	1.0
lambda2	-0.14	2.8e-01	7.1e+00	-1.2e+01	12	638	369	1.0
sigma	1.0	2.6e-03	8.0e-02	9.2e-01	1.2	939	543	1.0
mu	0.16	1.8e-03	1.0e-01	-8.1e-03	0.33	3289	1902	1.0

One Scale Parameter, Improper Prior

```
Warmup took (0.011, 0.012, 0.011, 0.011) seconds, 0.044 seconds total
Sampling took (0.017, 0.020, 0.020, 0.019) seconds, 0.077 seconds total
```

	Mean	MCSE	StdDev	5%	50%	95%	N_Eff	N_Eff/s	R_hat
lp__	-54	2.5e-02	0.91	-5.5e+01	-53	-53	1318	17198	1.0
n_leapfrog__	3.2	2.7e-01	1.7	1.0e+00	3.0	7.0	39	507	1.0
mu	0.17	2.1e-03	0.10	-3.8e-03	0.17	0.33	2408	31417	1.0
sigma	1.0	1.6e-03	0.071	9.3e-01	1.0	1.2	2094	27321	1.0

Figure 18.2: *Results of Stan runs with default parameters fit to $N = 100$ data points generated from $y_n \sim \mathsf{Normal}(0, 1)$. On the top is the non-identified model with improper uniform priors and likelihood $y_n \sim \mathsf{Normal}(\lambda_1 + \lambda_2, \sigma)$. In the middle is the same likelihood as the middle plus priors $\lambda_k \sim \mathsf{Normal}(0, 10)$. On the bottom is an identified model with an improper prior, with likelihood $y_n \sim \mathsf{Normal}(\mu, \sigma)$. All models estimate μ at roughly 0.16 with very little Monte Carlo standard error, but a high posterior standard deviation of 0.1; the true value $\mu = 0$ is within the 90% posterior intervals in all three models.*

```
data {
  int N;
  real y[N];
}
parameters {
  real lambda1;
  real lambda2;
  real<lower=0> sigma;
}
transformed parameters {
  real mu;
  mu <- lambda1 + lambda2;
}
model {
  y ~ normal(mu, sigma);
}
```

The second adds priors to the model block for `lambda1` and `lambda2` to the previous model.

```
  lambda1 ~ normal(0,10);
  lambda2 ~ normal(0,10);
```

The third involves a single location parameter, but no priors.

```
data {
  int N;
  real y[N];
}
parameters {
  real mu;
  real<lower=0> sigma;
}
model {
  y ~ normal(mu, sigma);
}
```

All three of the example models were fit in Stan 2.1.0 with default parameters (1000 warmup iterations, 1000 sampling iterations, NUTS sampler with max tree depth of 10). The results are shown in Figure 18.2. The key statistics from these outputs are the following.

- As indicated by `R_hat` column, all parameters have converged other than λ_1 and λ_2 in the non-identified model.
- The average number of leapfrog steps is roughly 3 in the identified model, 150 in the model identified by a weak prior, and 1400 in the non-identified model.
- The number of effective samples per second for μ is roughly 31,000 in the identified model, 1900 in the model identified with weakly informative priors, and 200 in the non-identified model; the results are similar for σ.
- In the non-identified model, the 95% interval for λ_1 is (-2300,6000), whereas it is only (-12,12) in the model identified with weakly informative priors.
- In all three models, the simulated value of $\mu = 0$ and $\sigma = 1$ are well within the posterior 90% intervals.

The first two points, lack of convergence and hitting the maximum number of leapfrog steps (equivalently maximum tree depth) are indicative of improper posteriors. Thus rather than covering up the problem with poor sampling as may be done with Gibbs samplers, Hamiltonian Monte Carlo tries to explore the posterior and its failure is a clear indication that something is amiss in the model.

19. Optimizing Stan Code

This chapter provides a grab bag of techniques for optimizing Stan code, including vectorization, sufficient statistics, and conjugacy.

19.1. Reparameterization

Stan's sampler can be slow in sampling from distributions with difficult posterior geometries. One way to speed up such models is through reparameterization.

Example: Neal's Funnel

In this section, we discuss a general transform from a centered to a non-centered parameterization Papaspiliopoulos et al. (2007).[1] This reparameterization is helpful because it separates the hierarchical parameters and lower-level parameters in the prior.

(Neal, 2003) defines a distribution that exemplifies the difficulties of sampling from some hierarchical models. Neal's example is fairly extreme, but can be trivially reparameterized in such a way as to make sampling straightforward.

Neal's example has support for $y \in \mathbb{R}$ and $x \in \mathbb{R}^9$ with density

$$p(y, x) = \mathsf{Normal}(y|0, 3) \times \prod_{n=1}^{9} \mathsf{Normal}(x_n|0, \exp(y/2)).$$

The probability contours are shaped like ten-dimensional funnels. The funnel's neck is particularly sharp because of the exponential function applied to y. A plot of the log marginal density of y and the first dimension x_1 is shown in Figure 19.1.

The funnel can be implemented directly in Stan as follows.

```
parameters {
  real y;
  vector[9] x;
}
model {
  y ~ normal(0,3);
  x ~ normal(0,exp(y/2));
}
```

When the model is expressed this way, Stan has trouble sampling from the neck of the funnel, where y is small and thus x is constrained to be near 0. This is due to the

[1] This parameterization came to be known on our mailing lists as the "Matt trick" after Matt Hoffman, who independently came up with it while fitting hierarchical models in Stan.

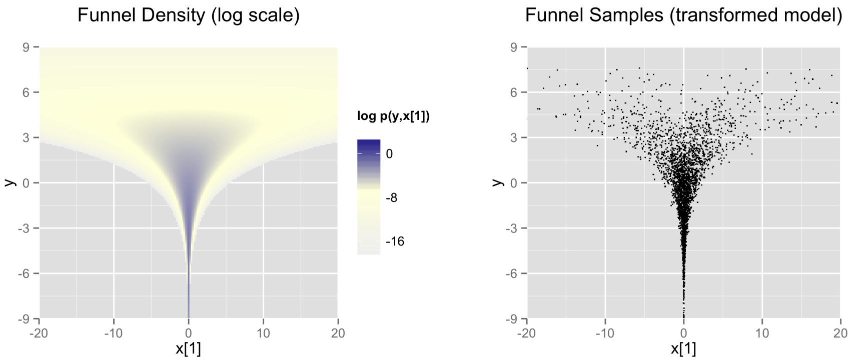

Figure 19.1: *Neal's Funnel. (Left) The marginal density of Neal's funnel for the upper-level variable y and one lower-level variable x_1 (see the text for the formula). The blue region has log density greater than -8, the yellow region density greater than -16, and the gray background a density less than -16. (Right) 4000 draws from a run of Stan's sampler with default settings. Both plots are restricted to the shown window of x_1 and y values; some draws fell outside of the displayed area as would be expected given the density. The samples are consistent with the marginal density $p(y) = \mathsf{Normal}(y|0,3)$, which has mean 0 and standard deviation 3.*

fact that the density's scale changes with y, so that a step size that works well in the body will be too large for the neck and a step size that works in the neck will be very inefficient in the body.

In this particular instance, because the analytic form of the density from which samples are drawn is known, the model can be converted to the following more efficient form.

```
parameters {
  real y_raw;
  vector[9] x_raw;
}
transformed parameters {
  real y;
  vector[9] x;

  y <- 3.0 * y_raw;
  x <- exp(y/2) * x_raw;
}
model {
  y_raw ~ normal(0,1); // implies y ~ normal(0,3)
  x_raw ~ normal(0,1); // implies x ~ normal(0,exp(y/2))
}
```

In this second model, the parameters x_raw and y_raw are sampled as independent unit normals, which is easy for Stan. These are then transformed into samples from the funnel. In this case, the same transform may be used to define Monte Carlo samples directly based on independent unit normal samples; Markov chain Monte Carlo methods are not necessary. If such a reparameterization were used in Stan code, it is useful to provide a comment indicating what the distribution for the parameter implies for the distribution of the transformed parameter.

Reparameterizing the Cauchy

Sampling from heavy tailed distributions such as the Cauchy is difficult for Hamiltonian Monte Carlo, which operates within a Euclidean geometry.[2] The practical problem is that tail of the Cauchy requires a relatively large step size compared to the trunk. With a small step size, the No-U-Turn sampler requires many steps when starting in the tail of the distribution; with a large step size, there will be too much rejection in the central portion of the distribution. This problem may be mitigated by defining the Cauchy-distributed variable as the transform of a uniformly distributed variable using the Cauchy inverse cumulative distribution function.

Suppose a random variable of interest X has a Cauchy distribution with location μ and scale τ, so that $X \sim \mathsf{Cauchy}(\mu, \tau)$. The variable X has a cumulative distribution function $F_X : \mathbb{R} \rightarrow (0, 1)$ defined by

$$F_X(x) = \frac{1}{\pi} \arctan\left(\frac{x - \mu}{\tau}\right) + \frac{1}{2}.$$

The inverse of the cumulative distribution function, $F_X^{-1} : (0, 1) \rightarrow \mathbb{R}$, is thus

$$F_X^{-1}(y) = \mu + \tau \tan\left(\pi\left(x - \frac{1}{2}\right)\right).$$

Thus if the random variable Y has a unit uniform distribution, $Y \sim \mathsf{Uniform}(0, 1)$, then $F_X^{-1}(Y)$ has a Cauchy distribution with location μ and scale τ, i.e., $F_X^{-1}(Y) \sim \mathsf{Cauchy}(\mu, \tau)$.

Consider a Stan program involving a Cauchy-distributed parameter beta.

```
parameters {
  real beta;
  ...
}
model {
```

[2]Riemannian Manifold Hamiltonian Monte Carlo (RMHMC) overcomes this difficulty by simulating the Hamiltonian dynamics in a a space with a position-dependent metric; see (Girolami and Calderhead, 2011) and (Betancourt, 2012).

```
    beta ~ cauchy(mu,tau);
    ...
  }
```

This declaration of beta as a parameter may be replaced with a transformed parameter beta defined in terms of a uniform-distributed parameter beta_unif.

```
  parameters {
    real<lower=-pi()/2, upper=pi()/2> beta_unif;
    ...
  }
  transformed parameters {
    real beta;
    beta <- mu + tau * tan(beta_unif);  // beta ~ cauchy(mu,tau)
  }
  model {
    beta_unif ~ uniform(-pi()/2, pi()/2);  // not necessary
    ...
  }
```

It is more convenient in Stan to transform a uniform variable on $(-\pi/2, \pi/2)$ than one on $(0, 1)$. The Cauchy location and scale parameters, mu and tau, may be defined as data or may themselves be parameters. The variable beta could also be defined as a local variable if it does not need to be included in the sampler's output.

The uniform distribution on beta_unif is defined explicitly in the model block, but it could be safely removed from the program without changing sampling behavior. This is because $\log \mathsf{Uniform}(\beta_{\text{unif}} | -\pi/2, \pi/2) = -\log \pi$ is a constant and Stan only needs the total log probability up to an additive constant. Stan will spend some time checking that that beta_unif is between -pi()/2 and pi()/2, but this condition is guaranteed by the constraints in the declaration of beta_unif.

Reparameterizing a Student-t Distribution

One thing that sometimes works when you're having trouble with the heavy-tailedness of Student-t distributions is to use the gamma-mixture representation, which says that you can generate a Student-t distributed variable β,

$$\beta \sim \mathsf{Student\text{-}t}(\nu, 0, 1),$$

by first generating a gamma-distributed precision (inverse variance) τ according to

$$\tau \sim \mathsf{Gamma}(\nu/2, \nu/2),$$

and then generating β from the normal distribution,

$$\beta \sim \mathsf{Normal}(0, \tau^{-\frac{1}{2}}).$$

Because τ is precision, $\tau^{-\frac{1}{2}}$ is the scale (standard deviation), which is the parameterization used by Stan.

The marginal distribution of β when you integrate out τ is $\mathsf{Student\text{-}t}(\nu, 0, 1)$, i.e.,

$$\mathsf{Student\text{-}t}(\beta|\nu, 0, 1). = \int_0^\infty \mathsf{Normal}(\beta|0, 1/\tau^{-\frac{1}{2}}) \times \mathsf{Gamma}(\tau|\nu/2, \nu/2)\, d\tau.$$

To go one step further, instead of defining a β drawn from a normal with precision τ, define α to be drawn from a unit normal,

$$\alpha \sim \mathsf{Normal}(0, 1)$$

and rescale by defining

$$\beta = \alpha\tau^{-\frac{1}{2}}.$$

Now suppose $\mu = \beta x$ is the product of β with a regression predictor x. Then the reparameterization $\mu = \alpha\tau^{-\frac{1}{2}}x$ has the same distribution, but in the original, direct parameterization, β has (potentially) heavy tails, whereas in the second, neither τ nor α have heavy tails.

To translate into Stan notation, this reparameterization replaces

```
parameters {
  real<lower=0> nu;
  real beta;
  ...
model {
  beta ~ student_t(nu,0,1);
  ...
```

with

```
parameters {
  real<lower=0> nu;
  real<lower=0> tau;
  ...
transformed parameters {
  real beta;
  beta <- alpha / sqrt(tau);
  ...
model {
```

```
    real half_nu;
    half_nu <- 0.5 * nu;
    tau ~ gamma(half_nu, half_nu);
    alpha ~ normal(0, 1);
    ...
```

Although set to 0 here, in most cases, the lower bound for the degrees of freedom parameter nu can be set to 1 or higher; when nu is 1, the result is a Cauchy distribution with very fat tails and as nu approaches infinity, the Student-t distribution approaches a normal distribution. Thus the parameter nu characterizes the heaviness of the tails of the model.

Hierarchical Models

Unfortunately, the usual situation in applied Bayesian modeling involves complex geometries and interactions that are not known analytically. Nevertheless, reparameterization can still be very effective for separating parameters. For example, a vectorized hierarchical model might draw a vector of coefficients β with definitions as follows.

```
parameters {
  real mu_beta;
  real<lower=0> sigma_beta;
  vector[K] beta;
  ...
model {
  beta ~ normal(mu_beta,sigma_beta);
  ...
```

Although not shown, a full model will have priors on both mu_beta and sigma_beta along with data modeled based on these coefficients. For instance, a standard binary logistic regression with data matrix x and binary outcome vector y would include a likelihood statement such as form y ~ bernoulli_logit(x * beta), leading to an analytically intractable posterior.

A hierarchical model such as the above will suffer from the same kind of inefficiencies as Neal's funnel, though typically not so extreme, because the values of beta, mu_beta and sigma_beta are highly correlated in the posterior. When there is a lot of data, such a hierarchical model can be made much more efficient by shifting the data's correlation with the parameters to the hyperparameters. Similar to the funnel example, this will be much more efficient in terms of effective sample size (Betancourt and Girolami, 2013).

```
parameters {
  vector[K] beta_raw;
```

```
  ...
transformed parameters {
  vector[K] beta;
  // implies: beta ~ normal(mu_beta,sigma_beta)
  beta <- mu_beta + sigma_beta * beta_raw;
model {
  beta_raw ~ normal(0,1);
  ...
```

Any priors defined for mu_beta and sigma_beta remain as defined in the original model.

Reparameterization of hierarchical models is not limited to the normal distribution, although the normal distribution is the best candidate for doing so. In general, any distribution of parameters in the location-scale family is a good candidate for reparameterization. Let $\beta = l + s\alpha$ where l is a location parameter and s is a scale parameter. Note that l need not be the mean, s need not be the standard deviation, and neither the mean nor the standard deviation need to exist. If α and β are from the same distributional family but α has location zero and unit scale, while β has location l and scale s, then that distribution is a location-scale distribution. Thus, if α were a parameter and β were a transformed parameter, then a prior distribution from the location-scale family on α with location zero and unit scale implies a prior distribution on β with location l and scale s. Doing so would reduce the dependence between α, l, and s.

There are several univariate distributions in the location-scale family, such as the Student t distribution, including its special cases of the Cauchy distribution (with one degree of freedom) and the normal distribution (with infinite degrees of freedom). As shown above, if α is distributed standard normal, then β is distributed normal with mean $\mu = l$ and standard deviation $\sigma = s$. The logistic, the double exponential, the generalized extreme value distributions, and the stable distribution are also in the location-scale family.

Also, if z is distributed standard normal, then z^2 is distributed chi-squared with one degree of freedom. By summing the squares of K independent standard normal variates, one can obtain a single variate that is distributed chi-squared with K degrees of freedom. However, for large K, the computational gains of this reparameterization may be overwhelmed by the computational cost of specifying K primitive parameters just to obtain one transformed parameter to use in a model.

Multivariate Reparameterizations

The benefits of reparameterization are not limited to univariate distributions. A parameter with a multivariate normal prior distribution is also an excellent candidate

for reparameterization. Suppose you intend the prior for β to be multivariate normal with mean vector μ and covariance matrix Σ. Such a belief is reflected by the following code.

```
data {
  int<lower=2> K;
  vector[K] mu;
  cov_matrix[K] Sigma;
  ...
parameters {
  vector[K] beta;
  ...
model {
  beta ~ multi_normal(mu,Sigma);
  ...
```

In this case mu and Sigma are fixed data, but they could be unknown parameters, in which case their priors would be unaffected by a reparameterization of beta.

If α has the same dimensions as β but the elements of α are independently and identically distributed standard normal such that $\beta = \mu + L\alpha$, where $LL^\top = \Sigma$, then β is distributed multivariate normal with mean vector μ and covariance matrix Σ. One choice for L is the Cholesky factor of Σ. Thus, the model above could be reparameterized as follows.

```
data {
  int<lower=2> K;
  vector[K] mu;
  cov_matrix[K] Sigma;
  ...
transformed data {
  matrix[K,K] L;
  L <- cholesky_decompose(Sigma);
}
parameters {
  vector[K] alpha;
  ...
transformed parameters {
  vector[K] beta;
  beta <- mu + L * alpha;
}
model {
  alpha ~ normal(0,1);
  // implies: beta ~ multi_normal(mu, Sigma)
  ...
```

This reparameterization is more efficient for two reasons. First, it reduces dependence among the elements of alpha and second, it avoids the need to invert Sigma every time multi_normal is evaluated.

The Cholesky factor is also useful when a covariance matrix is decomposed into a correlation matrix that is multiplied from both sides by a diagonal matrix of standard deviations, where either the standard deviations or the correlations are unknown parameters. The Cholesky factor of the covariance matrix is equal to the product of a diagonal matrix of standard deviations and the Cholesky factor of the correlation matrix. Furthermore, the product of a diagonal matrix of standard deviations and a vector is equal to the elementwise product between the standard deviations and that vector. Thus, if for example the correlation matrix Tau were fixed data but the vector of standard deviations sigma were unknown parameters, then a reparameterization of beta in terms of alpha could be implemented as follows.

```
data {
  int<lower=2> K;
  vector[K] mu;
  corr_matrix[K] Tau;
  ...
transformed data {
  matrix[K,K] L;
  L <- cholesky_decompose(Tau);
}
parameters {
  vector[K] alpha;
  vector<lower=0>[K] sigma;
  ...
transformed parameters {
  vector[K] beta;
  // This equals mu + diag_matrix(sigma) * L * alpha;
  beta <- mu + sigma .* (L * alpha);
}
model {
  sigma ~ cauchy(0,5);
  alpha ~ normal(0,1);
  // implies: beta ~ multi_normal(mu,
  //  diag_matrix(sigma) * L * L' * diag_matrix(sigma)))
  ...
```

This reparameterization of a multivariate normal distribution in terms of standard normal variates can be extended to other multivariate distributions that can be conceptualized as contaminations of the multivariate normal, such as the multivariate Student t and the skew multivariate normal distribution.

A Wishart distribution can also be reparameterized in terms of standard normal variates and chi-squared variates. Let L be the Cholesky factor of a $K \times K$ positive definite scale matrix S and let ν be the degrees of freedom. If

$$A = \begin{pmatrix} \sqrt{c_1} & 0 & \cdots & 0 \\ z_{21} & \sqrt{c_2} & \ddots & \vdots \\ \vdots & \ddots & \ddots & 0 \\ z_{K1} & \cdots & z_{K(K-1)} & \sqrt{c_K} \end{pmatrix},$$

where each c_i is distributed chi-squared with $\nu - i + 1$ degrees of freedom and each z_{ij} is distributed standard normal, then $W = LAA^\top L^\top$ is distributed Wishart with scale matrix $S = LL^\top$ and degrees of freedom ν. Such a reparameterization can be implemented by the following Stan code:

```
data {
  int<lower=1> N;
  int<lower=1> K;
  int<lower=K+2> nu
  matrix[K,K] L; // Cholesky factor of scale matrix
  vector[K] mu;
  matrix[N,K] y;
  ...
parameters {
  vector<lower=0>[K] c;
  vector[0.5 * K * (K - 1)] z;
  ...
model {
  matrix[K,K] A;
  int count;
  count <- 1;
  for (j in 1:(K-1)) {
    for (i in (j+1):K) {
      A[i,j] <- z[count];
      count <- count + 1;
    }
    for (i in 1:(j - 1)) {
      A[i,j] <- 0.0;
    }
    A[j,j] <- sqrt(c[j]);
  }

  for (i in 1:K) {
    c[i] ~ chi_square(nu - i + 1);
```

```
  }
  z ~ normal(0,1);
  // implies: L * A * A' * L' ~ wishart(nu, L * L')
  y ~ multi_normal_cholesky(mu, L * A);
  ...
```

This reparameterization is more efficient for three reasons. First, it reduces dependence among the elements of `z` and second, it avoids the need to invert the covariance matrix, W every time `wishart` is evaluated. Third, if W is to be used with a multivariate normal distribution, you can pass LA to the more efficient `multi_normal_cholesky` function, rather than passing W to `multi_normal`.

If W is distributed Wishart with scale matrix S and degrees of freedom ν, then W^{-1} is distributed inverse Wishart with inverse scale matrix S^{-1} and degrees of freedom ν. Thus, the previous result can be used to reparameterize the inverse Wishart distribution. Since $W = L * A * A^\top * L^\top$, $W^{-1} = L^{\top^{-1}} A^{\top^{-1}} A^{-1} L^{-1}$, where all four inverses exist, but $L^{-1^\top} = L^{\top^{-1}}$ and $A^{-1^\top} = A^{\top^{-1}}$. We can slightly modify the above Stan code for this case:

```
data {
  int<lower=1> K;
  int<lower=K+2> nu
  matrix[K,K] L; // Cholesky factor of scale matrix
  ...
transformed data {
  matrix[K,K] eye;
  matrix[K,K] L_inv;
  for (j in 1:K) {
    for (i in 1:K) {
      eye[i,j] <- 0.0;
    }
    eye[j,j] <- 1.0;
  }
  L_inv <- mdivide_left_tri_low(L, eye);
}
parameters {
  vector<lower=0>[K] c;
  vector[0.5 * K * (K - 1)] z;
  ...
model {
  matrix[K,K] A;
  matrix[K,K] A_inv_L_inv;
  int count;
  count <- 1;
  for (j in 1:(K-1)) {
```

```
    for (i in (j+1):K) {
      A[i,j] <- z[count];
      count <- count + 1;
    }
    for (i in 1:(j - 1)) {
      A[i,j] <- 0.0;
    }
    A[j,j] <- sqrt(c[j]);
  }
  A_inv_L_inv <- mdivide_left_tri_low(A, L_inv);
  for (i in 1:K) {
    c[i] ~ chi_square(nu - i + 1);
  }
  z ~ normal(0,1); // implies: crossprod(A_inv_L_inv) ~
  // inv_wishart(nu, L_inv' * L_inv)
  ...
```

Another candidate for reparameterization is the Dirichlet distribution with all K shape parameters equal. Zyczkowski and Sommers (2001) shows that if θ_i is equal to the sum of β independent squared standard normal variates and $\rho_i = \frac{\theta_i}{\sum \theta_i}$, then the K-vector ρ is distributed Dirichlet with all shape parameters equal to $\frac{\beta}{2}$. In particular, if $\beta = 2$, then ρ is uniformly distributed on the unit simplex. Thus, we can make ρ be a transformed parameter to reduce dependence, as in:

```
data {
  int<lower=1> beta;
  ...
parameters {
  vector[beta] z[K];
  ...
transformed parameters {
  simplex[K] rho;
  for (k in 1:K)
    rho[k] <- dot_self(z[k]); // sum-of-squares
  rho <- rho / sum(rho);
}
model {
  for (k in 1:K)
    z[k] ~ normal(0,1);
  // implies: rho ~ dirichlet(0.5 * beta * ones)
  ...
```

19.2. Vectorization

Gradient Bottleneck

Stan spends the vast majority of its time computing the gradient of the log probability function, making gradients the obvious target for optimization. Stan's gradient calculations with algorithmic differentiation require a template expression to be allocated[3] and constructed for each subexpression of a Stan program involving parameters or transformed parameters. This section defines optimization strategies based on vectorizing these subexpressions to reduce the work done during algorithmic differentiation.

Vectorizing Summations

Because of the gradient bottleneck described in the previous section, it is more efficient to collect a sequence of summands into a vector or array and then apply the `sum()` operation than it is to continually increment a variable by assignment and addition. For example, consider the following code snippet, where `foo()` is some operation that depends on `n`.

```
for (n in 1:N)
  total <- total + foo(n,...);
```

This code has to create intermediate representations for each of the `N` summands.

A faster alternative is to copy the values into a vector, then apply the `sum()` operator, as in the following refactoring.

```
{
  vector[N] summands;
  for (n in 1:N)
    summands[n] <- foo(n,...);
  total <- sum(summands);
}
```

Syntactically, the replacement is a statement block delineated by curly brackets ({, }), starting with the definition of the local variable `summands`.

Even though it involves extra work to allocate the `summands` vector and copy `N` values into it, the savings in differentiation more than make up for it. Perhaps surprisingly, it will also use substantially less memory overall than incrementing `total` within the loop.

[3] Stan uses its own arena-based allocation, so allocation and deallocation are faster than with a raw call to `new`.

Vectorization through Matrix Operations

The following program directly encodes a linear regression with fixed unit noise using a two-dimensional array `x` of predictors, an array `y` of outcomes, and an array `beta` of regression coefficients.

```
data {
  int<lower=1> K;
  int<lower=1> N;
  real x[K,N];
  real y[N];
}
parameters {
  real beta[K];
}
model {
  for (n in 1:N) {
    real gamma;
    gamma <- 0.0;
    for (k in 1:K)
      gamma <- gamma + x[n,k] * beta[k];
    y[n] ~ normal(gamma,1);
  }
}
```

The following model computes the same log probability function as the previous model, even supporting the same input files for data and initialization.

```
data {
  int<lower=1> K;
  int<lower=1> N;
  vector[K] x[N];
  real y[N];
}
parameters {
  vector[K] beta;
}
model {
  for (n in 1:N)
    y[n] ~ normal(dot_product(x[n],beta), 1);
}
```

Although it produces equivalent results, the dot product should not be replaced with a transpose and multiply, as in

```
y[n] ~ normal(x[n]' * beta, 1);
```

The relative inefficiency of the transpose and multiply approach is that the transposition operator allocates a new vector into which the result of the transposition is copied. This consumes both time and memory[4]. The inefficiency of transposition could itself be mitigated somewhat by reordering the product and pulling the transposition out of the loop, as follows.

```
...
transformed parameters {
  row_vector[K] beta_t;
  beta_t <- beta';
}
model {
  for (n in 1:N)
    y[n] ~ normal(beta_t * x[n], 1);
}
```

The problem with transposition could be completely solved by directly encoding the x as a row vector, as in the following example.

```
data {
  ...
  row_vector[K] x[N];
  ...
}
parameters {
  vector[K] beta;
}
model {
  for (n in 1:N)
    y[n] ~ normal(x[n] * beta, 1);
}
```

Declaring the data as a matrix and then computing all the predictors at once using matrix multiplication is more efficient still, as in the example discussed in the next section.

Vectorized Probability Functions

The final and most efficient version replaces the loops and transformed parameters by using the vectorized form of the normal probability function, as in the following example.

[4]Future versions of Stan may remove this inefficiency by more fully exploiting expression templates inside the Eigen C++ matrix library. This will require enhancing Eigen to deal with mixed-type arguments, such as the type `double` used for constants and the algorithmic differentiation type `stan::agrad::var` used for variables.

```
data {
  int<lower=1> K;
  int<lower=1> N;
  matrix[N,K] x;
  vector[N] y;
}
parameters {
  vector[K] beta;
}
model {
  y ~ normal(x * beta, 1);
}
```

The variables are all declared as either matrix or vector types. The result of the matrix-vector multiplication `x * beta` in the model block is a vector of the same length as `y`.

The probability function documentation in Part IV indicates which of Stan's probability functions support vectorization; see Section 28.1 for more information. Vectorized probability functions accept either vector or scalar inputs for all arguments, with the only restriction being that all vector arguments are the same dimensionality. In the example above, `y` is a vector of size `N`, `x * beta` is a vector of size `N`, and `1` is a scalar.

19.3. Exploiting Sufficient Statistics

In some cases, models can be recoded to exploit sufficient statistics in estimation. This can lead to large efficiency gains compared to an expanded model. For example, consider the following Bernoulli sampling model.

```
data {
  int<lower=0> N;
  int<lower=0,upper=1> y[N];
  real<lower=0> alpha;
  real<lower=0> beta;
}
parameters {
  real<lower=0,upper=1> theta;
}
model {
  theta ~ beta(alpha,beta);
  for (n in 1:N)
    y[n] ~ bernoulli(theta);
}
```

In this model, the sum of positive outcomes in y is a sufficient statistic for the chance of success theta. The model may be recoded using the binomial distribution as follows.

```
theta ~ beta(alpha,beta);
sum(y) ~ binomial(N,theta);
```

Because truth is represented as one and falsehood as zero, the sum sum(y) of a binary vector y is equal to the number of positive outcomes out of a total of N trials.

19.4. Exploiting Conjugacy

Continuing the model from the previous section, the conjugacy of the beta prior and binomial sampling distribution allow the model to be further optimized to the following equivalent form.

```
theta ~ beta(alpha + sum(y), beta + N - sum(y));
```

To make the model even more efficient, a transformed data variable defined to be sum(y) could be used in the place of sum(y).

19.5. Standardizing Predictors and Outputs

Stan programs will run faster if the input is standardized to have a zero sample mean and unit sample variance. This section illustrates the principle with a simple linear regression.

Suppose that $y = (y_1, \ldots, y_N)$ is a sequence of N outcomes and $x = (x_1, \ldots, x_N)$ a parallel sequence of N predictors. A simple linear regression involving an intercept coefficient α and slope coefficient β can be expressed as

$$y_n = \alpha + \beta x_n + \epsilon_n,$$

where

$$\epsilon_n \sim \mathsf{Normal}(0, \sigma).$$

If either vector x or y has very large or very small values or if the sample mean of the values is far away from 0 (on the scale of the values), then it can be more efficient to standardize the outputs y_n and predictors x_n. The data is first centered by subtracting the sample mean, and then scaled by dividing by the sample deviation. Thus a data point u is standardized is standardized with respect to a vector y by the function z_y, defined by

$$\mathrm{z}_y(u) = \frac{u - \bar{y}}{\mathrm{sd}(y)}$$

where the sample mean of y is

$$\bar{y} = \frac{1}{N} \sum_{n=1}^{N} y_n,$$

and the sample standard deviation of y is

$$\mathrm{sd}(y) = \left(\frac{1}{N} \sum_{n=1}^{N} (y_n - \bar{y})^2 \right)^{1/2}.$$

The inverse transform is defined by reversing the two normalization steps, first rescaling by the same deviation and relocating by the sample mean,

$$\mathrm{z}^{-1}(v) = \mathrm{sd}(y)v + \bar{y}.$$

To standardize a regression problem, the predictors and outcomes are standardized. This changes the scale of the variables, and hence changes the scale of the priors. Consider the following initial model.

```
data {
  int<lower=0> N;
  vector[N] y;
  vector[N] x;
}
parameters {
  real alpha;
  real beta;
  real<lower=0> sigma;
}
model {
  // priors
  alpha ~ normal(0,10);
  beta ~ normal(0,10);
  sigma ~ cauchy(0,5);
  // likelihood
  for (n in 1:N)
    y[n] ~ normal(alpha + beta * x[n], sigma);
}
```

The data block for the standardized model is identical. The standardized predictors and outputs are defined in the transformed data block.

```
data {
  int<lower=0> N;
  vector[N] y;
```

```
  vector[N] x;
}
transformed data {
  vector[N] x_std;
  vector[N] y_std;
  x_std <- (x - mean(x)) / sd(x);
  y_std <- (y - mean(y)) / sd(y);
}
parameters {
  real alpha_std;
  real beta_std;
  real<lower=0> sigma_std;
}
model {
  alpha_std ~ normal(0,10);
  beta_std ~ normal(0,10);
  sigma_std ~ cauchy(0,5);
  for (n in 1:N)
    y_std[n] ~ normal(alpha_std + beta_std * x_std[n],
                      sigma_std);
}
```

The parameters are renamed to indicate that they aren't the "natural" parameters, but the model is otherwise identical. In particular, the fairly diffuse priors on the coefficients and error scale are the same. These could have been transformed as well, but here they are left as is, because the scales make sense as very diffuse priors for standardized data; the priors could be made more informative. For instance, because the outputs y have been standardized, the error σ should not be greater than 1, because that's the scale of the noise for predictors $\alpha = \beta = 0$.

The original regression

$$y_n = \alpha + \beta x_n + \epsilon_n$$

has been transformed to a regression on the standardized variables,

$$\text{z}_y(y_n) = \alpha' + \beta' \text{z}_x(x_n) + \epsilon'_n.$$

The original parameters can be recovered with a little algebra,

$$\begin{aligned}
y_n &= \mathrm{z}_y^{-1}(\mathrm{z}_y(y_n)) \\
&= \mathrm{z}_y^{-1}\left(\alpha' + \beta' \mathrm{z}_x(x_n) + \epsilon'_n\right) \\
&= \mathrm{z}_y^{-1}\left(\alpha' + \beta' \left(\frac{x_n - \bar{x}}{\mathrm{sd}(x)}\right) + \epsilon'_n\right) \\
&= \mathrm{sd}(y)\left(\alpha' + \beta' \left(\frac{x_n - \bar{x}}{\mathrm{sd}(x)}\right) + \epsilon'_n\right) + \bar{y} \\
&= \left(\mathrm{sd}(y)\left(\alpha' - \beta' \frac{\bar{x}}{\mathrm{sd}(x)}\right) + \bar{y}\right) + \left(\beta' \frac{\mathrm{sd}(y)}{\mathrm{sd}(x)}\right) x_n + \mathrm{sd}(y)\epsilon'_n,
\end{aligned}$$

from which the original scale parameter values can be read off,

$$\alpha = \mathrm{sd}(y)\left(\alpha' - \beta' \frac{\bar{x}}{\mathrm{sd}(x)}\right) + \bar{y}; \qquad \beta = \beta' \frac{\mathrm{sd}(y)}{\mathrm{sd}(x)}; \qquad \sigma = \mathrm{sd}(y)\sigma'.$$

These recovered parameter values on the original scales can be calculated within Stan using a generated quantities block following the model block,

```
generated quantities {
  real alpha;
  real beta;
  real<lower=0> sigma;
  alpha <- sd(y) * (alpha_std - beta_std * mean(x) / sd(x))
           + mean(y);
  beta <- beta_std * sd(y) / sd(x);
  sigma <- sd(y) * sigma_std;
}
```

Of course, it is inefficient to compute all of the means and standard deviations every iteration; for more efficiency, these can be calculated once and stored as transformed data. Furthermore, the model sampling statement can be easily vectorized, for instance, in the transformed model, to

```
y_std ~ normal(alpha_std + beta_std * x_std, sigma_std);
```

20. Reproducibility

Floating point operations on modern computers are notoriously difficult to replicate because the fundamental arithmetic operations, right down to the IEEE 754 encoding level, are not fully specified. The primary problem is that the precision of operations varies across different hardware platforms and software implementations.

Stan is designed to allow full reproducibility. However, this is only possible up to the external constraints imposed by floating point arithmetic.

Stan results will only be exactly reproducible if *all* of the following components are *identical*:

- Stan version
- Stan interface (RStan, PyStan, CmdStan) and version, plus version of interface language (R, Python, shell)
- versions of included libraries (Boost and Eigen)
- operating system version
- computer hardware including CPU, motherboard and memory
- C++ compiler, including version, compiler flags, and linked libraries
- same configuration of call to Stan, including random seed, chain ID, initialization and data

It doesn't matter if you use a stable release version of Stan or the version with a particular Git hash tag. The same goes for all of the interfaces, compilers, and so on on. The point is that if any of these moving parts changes in some way, floating point results may change.

Concretely, if you compile a single Stan program using the same CmdStan code base, but changed the optimization flag (`-O3` vs. `-O2` or `-O0`), the two programs may not return the identical stream of results. Thus it is very hard to guarantee reproducibility on externally managed hardware, like in a cluster or even a desktop managed by an IT department or with automatic updates turned on.

If, however, you compiled a Stan program today using one set of flags, took the computer away from the internet and didn't allow it to update anything, then came back in a decade and recompiled the Stan program in the same way, you should get the same results.

The data needs to be the same down to the bit level. For example, if you are running in RStan, Rcpp handles the conversion between R's floating point numbers and C++ doubles. If Rcpp changes the conversion process or use different types, the results are not guaranteed to be the same down to the bit level.

Part III

Modeling Language Reference

21. Execution of a Stan Program

This chapter provides a sketch of how a compiled Stan model is executed using sampling. Optimization shares the same data reading and initialization steps, but then does optimization rather than sampling.

This sketch is elaborated in the following chapters of this part, which cover variable declarations, expressions, statements, and blocks in more detail.

21.1. Reading and Transforming Data

The reading and transforming data steps are the same for sampling, optimization and diagnostics.

Read Data

The first step of execution is to read data into memory. Data may be read in through file (in CmdStan) or through memory (RStan and PyStan); see their respective manuals for details. [1] All of the variables declared in the `data` block will be read. If a variable cannot be read, the program will halt with a message indicating which data variable is missing.

After each variable is read, if it has a declared constraint, the constraint is validated. For example, if a variable `N` is declared as `int<lower=0>`, after `N` is read, it will be tested to make sure it is greater than or equal to zero. If a variable violates its declared constraint, the program will halt with a warning message indicating which variable contains an illegal value, the value that was read, and the constraint that was declared.

Define Transformed Data

After data is read into the model, the transformed data variable statements are executed in order to define the transformed data variables. As the statements execute, declared constraints on variables are not enforced.

After the statements are executed, all declared constraints on transformed data variables are validated. If the validation fails, execution halts and the variable's name, value and constraints are displayed.

[1] The C++ code underlying Stan is flexible enough to allow data to be read from memory or file. Calls from R, for instance, can be configured to read data from file or directly from R's memory.

21.2. Initialization

Initialization is the same for sampling, optimization, and diagnosis

User-Supplied Initial Values

If there are user-supplied initial values for parameters, these are read using the same input mechanism and same file format as data reads. Any constraints declared on the parameters are validated for the initial values. If a variable's value violates its declared constraint, the program halts and a diagnostic message is printed.

After being read, initial values are transformed to unconstrained values that will be used to initialize the sampler.

Boundary Values are Problematic

Because of the way Stan defines its transforms from the constrained to the unconstrained space, initializing parameters on the boundaries of their constraints is usually problematic. For instance, with a constraint

```
parameters {
  real<lower=0,upper=1> theta;
  ...
}
```

an initial value of 0 for `theta` leads to an unconstrained value of $-\infty$, whereas a value of 1 leads to an unconstrained value of $+\infty$. While this will be inverse transformed back correctly given the behavior of floating point arithmetic, the Jacobian will be infinite and the log probability function will fail and raise an exception.

Random Initial Values

If there are no user-supplied initial values, the default initialization strategy is to initialize the unconstrained parameters directly with values drawn uniformly from the interval $(-2, 2)$. The bounds of this initialization can be changed but it is always symmetric around 0. The value of 0 is special in that it represents the median of the initialization. An unconstrained value of 0 corresponds to different parameter values depending on the constraints declared on the parameters.

An unconstrained real does not involve any transform, so an initial value of 0 for the unconstrained parameters is also a value of 0 for the constrained parameters.

For parameters that are bounded below at 0, the initial value of 0 on the unconstrained scale corresponds to $\exp(0) = 1$ on the constrained scale. A value of -2 corresponds to $\exp(-2) = .13$ and a value of 2 corresponds to $\exp(2) = 7.4$.

For parameters bounded above and below, the initial value of 0 on the unconstrained scale corresponds to a value at the midpoint of the constraint interval. For probability parameters, bounded below by 0 and above by 1, the transform is the inverse logit, so that an initial unconstrained value of 0 corresponds to a constrained value of 0.5, -2 corresponds to 0.12 and 2 to 0.88. Bounds other than 0 and 1 are just scaled and translated.

Simplexes with initial values of 0 on the unconstrained basis correspond to symmetric values on the constrained values (i.e., each value is $1/K$ in a K-simplex).

Cholesky factors for positive-definite matrices are initialized to 1 on the diagonal and 0 elsewhere; this is because the diagonal is log transformed and the below-diagonal values are unconstrained.

The initial values for other parameters can be determined from the transform that is applied. The transforms are all described in full detail in Chapter 53.

Zero Initial Values

The initial values may all be set to 0 on the unconstrained scale. This can be helpful for diagnosis, and may also be a good starting point for sampling. Once a model is running, multiple chains with more diffuse starting points can help diagnose problems with convergence; see Section 52.3 for more information on convergence monitoring.

21.3. Sampling

Sampling is based on simulating the Hamiltonian of a particle with a starting position equal to the current parameter values and an initial momentum (kinetic energy) generated randomly. The potential energy at work on the particle is taken to be the negative log (unnormalized) total probability function defined by the model. In the usual approach to implementing HMC, the Hamiltonian dynamics of the particle is simulated using the leapfrog integrator, which discretizes the smooth path of the particle into a number of small time steps called leapfrog steps.

Leapfrog Steps

For each leapfrog step, the negative log probability function and its gradient need to be evaluated at the position corresponding to the current parameter values (a more detailed sketch is provided in the next section). These are used to update the momentum based on the gradient and the position based on the momentum.

For simple models, only a few leapfrog steps with large step sizes are needed. For models with complex posterior geometries, many small leapfrog steps may be needed to accurately model the path of the parameters.

If the user specifies the number of leapfrog steps (i.e., chooses to use standard HMC), that number of leapfrog steps are simulated. If the user has not specified the number of leapfrog steps, the No-U-Turn sampler (NUTS) will determine the number of leapfrog steps adaptively (Hoffman and Gelman, 2011, 2013).

Log Probability and Gradient Calculation

During each leapfrog step, the log probability function and its gradient must be calculated. This is where most of the time in the Stan algorithm is spent. This log probability function, which is used by the sampling algorithm, is defined over the unconstrained parameters.

The first step of the calculation requires the inverse transform of the unconstrained parameter values back to the constrained parameters in terms of which the model is defined. There is no error checking required because the inverse transform is a total function on every point in whose range satisfies the constraints.

Because the probability statements in the model are defined in terms of constrained parameters, the log Jacobian of the inverse transform must be added to the accumulated log probability.

Next, the transformed parameter statements are executed. After they complete, any constraints declared for the transformed parameters are checked. If the constraints are violated, the model will halt with a diagnostic error message.

The final step in the log probability function calculation is to execute the statements defined in the model block.

As the log probability function executes, it accumulates an in-memory representation of the expression tree used to calculate the log probability. This includes all of the transformed parameter operations and all of the Jacobian adjustments. This tree is then used to evaluate the gradients by propagating partial derivatives backward along the expression graph. The gradient calculations account for the majority of the cycles consumed by a Stan program.

Metropolis Accept/Reject

A standard Metropolis accept/reject step is required to retain detailed balance and ensure samples are marginally distributed according to the probability function defined by the model. This Metropolis adjustment is based on comparing log probabilities, here defined by the Hamiltonian, which is the sum of the potential (negative log probability) and kinetic (squared momentum) energies. In theory, the Hamiltonian is invariant over the path of the particle and rejection should never occur. In practice, the probability of rejection is determined by the accuracy of the leapfrog approximation to the true trajectory of the parameters.

If step sizes are small, very few updates will be rejected, but many steps will be required to move the same distance. If step sizes are large, more updates will be rejected, but fewer steps will be required to move the same distance. Thus a balance between effort and rejection rate is required. If the user has not specified a step size, Stan will tune the step size during warmup sampling to achieve a desired rejection rate (thus balancing rejection versus number of steps).

If the proposal is accepted, the parameters are updated to their new values. Otherwise, the sample is the current set of parameter values.

21.4. Optimization

Optimization runs very much like sampling in that it starts by reading the data and then initializing parameters. Unlike sampling, it produces a deterministic output which requires no further analysis other than to verify that the optimizer itself converged to a posterior mode. The output for optimization is also similar to that for sampling.

21.5. Model Diagnostics

Model diagnostics are like sampling and optimization in that they depend on a model's data being read and its parameters being initialized. The user's guides for the interfaces (RStan, PyStan, CmdStan) provide more details on the diagnostics available; as of Stan 2.0, that's just gradients on the unconstrained scale and log probabilities.

21.6. Output

For each final sample (not counting samples during warmup or samples that are thinned), there is an output stage of writing the samples.

Generated Quantities

Before generating any output, the statements in the generated quantities block are executed. This can be used for any forward simulation based on parameters of the model. Or it may be used to transform parameters to an appropriate form for output.

After the generated quantities statements execute, the constraints declared on generated quantities variables are validated. If these constraints are violated, the program will terminate with a diagnostic message.

Write

The final step is to write the actual values. The values of all variables declared as parameters, transformed parameters, or generated quantities are written. Local variables are not written, nor is the data or transformed data. All values are written in their constrained forms, that is the form that is used in the model definitions.

In the executable form of a Stan models, parameters, transformed parameters, and generated quantities are written to a file in comma-separated value (csv) notation with a header defining the names of the parameters (including indices for multivariate parameters).[2]

[2] In the R version of Stan, the values may either be written to a csv file or directly back to R's memory.

22. Data Types and Variable Declarations

This chapter covers the data types for expressions in Stan. Every variable used in a Stan program must have a declared data type. Only values of that type will be assignable to the variable (except for temporary states of transformed data and transformed parameter values). This follows the convention of programming languages like C++, not the conventions of scripting languages like Python or statistical languages such as R or BUGS.

The motivation for strong, static typing is threefold.

- Strong typing forces the programmer's intent to be declared with the variable, making programs easier to comprehend and hence easier to debug and maintain.
- Strong typing allows programming errors relative to the declared intent to be caught sooner (at compile time) rather than later (at run time). The Stan compiler (called through an interface such as CmdStan, RStan, or PyStan) will flag any type errors and indicate the offending expressions quickly when the program is compiled.
- Constrained types will catch runtime data, initialization, and intermediate value errors as soon as they occur rather than allowing them to propagate and potentially pollute final results.

Strong typing disallows assigning the same variable to objects of different types at different points in the program or in different invocations of the program.

22.1. Overview of Data Types

Basic Data Types

The primitive Stan data types are `real` for continuous scalar quantities and `int` for integer values. The compound data types include `vector` (of real values), `row_vector` (of real values), and `matrix` (of real values).

Constrained Data Types

Integer or real types may be constrained with lower bounds, upper bounds, or both. There are four constrained vector data types, `simplex` for unit simplexes, `unit_vector` for unit-length vectors, `ordered` for ordered vectors of scalars and `positive_ordered` for vectors of positive ordered scalars. There are specialized matrix data types `corr_matrix` and `cov_matrix` for correlation matrices (symmetric,

positive definite, unit diagonal) and covariance matrices (symmetric, positive definite). The type `cholesky_factor_cov` is for Cholesky factors of covariance matrices (lower triangular, positive diagonal, product with own transpose is a covariance matrix). The type `cholesky_factor_corr` is for Cholesky factors of correlation matrices (lower triangular, positive diagonal, unit-length rows).

Arrays

Stan supports arrays of arbitrary order of any of the basic data types or constrained basic data types. This includes three-dimensional arrays of integers, one-dimensional arrays of positive reals, four-dimensional arrays of simplexes, one-dimensional arrays of row vectors, and so on.

22.2. Primitive Numerical Data Types

Unfortunately, the lovely mathematical abstraction of integers and real numbers is only partially supported by finite-precision computer arithmetic.

Integers

Stan uses 32-bit (4-byte) integers for all of its integer representations. The maximum value that can be represented as an integer is $2^{31} - 1$; the minimum value is $-(2^{31})$.

When integers overflow, their values wrap. Thus it is up to the Stan programmer to make sure the integer values in their programs stay in range. In particular, every intermediate expression must have an integer value that is in range.

Integer arithmetic works in the expected way for addition, subtraction, and multiplication, but rounds the result of division (see Section 30.1 for more information).

Reals

Stan uses 64-bit (8-byte) floating point representations of real numbers. Stan roughly[1] follows the IEEE 754 standard for floating-point computation. The range of a 64-bit number is roughly $\pm 2^{1022}$, which is slightly larger than $\pm 10^{307}$. It is a good idea to stay well away from such extreme values in Stan models as they are prone to cause overflow.

64-bit floating point representations have roughly 15 decimal digits of accuracy. But when they are combined, the result often has less accuracy. In some cases, the difference in accuracy between two operands and their result is large.

[1]Stan compiles integers to `int` and reals to `double` types in C++. Precise details of rounding will depend on the compiler and hardware architecture on which the code is run.

There are three special real values used to represent (1) error conditions, (2) positive infinity, and (3) negative infinity. The error value is referred to as "not a number."

Promoting Integers to Reals

Stan automatically promotes integer values to real values if necessary, but does not automatically demote real values to integers. For very large integers, this will cause a rounding error to fewer significant digits in the floating point representation than in the integer representation.

Unlike in C++, real values are never demoted to integers. Therefore, real values may only be assigned to real variables. Integer values may be assigned to either integer variables or real variables. Internally, the integer representation is cast to a floating-point representation. This operation is not without overhead and should thus be avoided where possible.

22.3. Univariate Data Types and Variable Declarations

All variables used in a Stan program must have an explicitly declared data type. The form of a declaration includes the type and the name of a variable. This section covers univariate types, the next section vector and matrix types, and the following section array types.

Unconstrained Integer

Unconstrained integers are declared using the `int` keyword. For example, the variable `N` is declared to be an integer as follows.

```
int N;
```

Constrained Integer

Integer data types may be constrained to allow values only in a specified interval by providing a lower bound, an upper bound, or both. For instance, to declare `N` to be a positive integer, use the following.

```
int<lower=1> N;
```

This illustrates that the bounds are inclusive for integers.

To declare an integer variable `cond` to take only binary values, that is zero or one, a lower and upper bound must be provided, as in the following example.

```
int<lower=0,upper=1> cond;
```

Unconstrained Real

Unconstrained real variables are declared using the keyword `real`, The following example declares `theta` to be an unconstrained continuous value.

```
real theta;
```

Constrained Real

Real variables may be bounded using the same syntax as integers. In theory (that is, with arbitrary-precision arithmetic), the bounds on real values would be exclusive. Unfortunately, finite-precision arithmetic rounding errors will often lead to values on the boundaries, so they are allowed in Stan.

The variable `sigma` may be declared to be non-negative as follows.

```
real<lower=0> sigma;
```

The following declares the variable `x` to be less than or equal to -1.

```
real<upper=-1> x;
```

To ensure `rho` takes on values between -1 and 1, use the following declaration.

```
real<lower=-1,upper=1> rho;
```

Infinite Constraints

Lower bounds that are negative infinity or upper bounds that are positive infinity are ignored. Stan provides constants `positive_infinity()` and `negative_infinity()` which may be used for this purpose, or they may be read as data in the dump format.

Expressions as Bounds

Bounds for integer or real variables may be arbitrary expressions. The only requirement is that they only include variables that have been defined before the declaration. If the bounds themselves are parameters, the behind-the-scenes variable transform accounts for them in the log Jacobian.

For example, it is acceptable to have the following declarations.

```
data {
 real lb;
}
parameters {
   real<lower=lb> phi;
}
```

This declares a real-valued parameter `phi` to take values greater than the value of the real-valued data variable `lb`. Constraints may be complex expressions, but must be of type `int` for integer variables and of type `real` for real variables (including constraints on vectors, row vectors, and matrices). Variables used in constraints can be any variable that has been defined at the point the constraint is used. For instance,

```
data {
   int<lower=1> N;
   real y[N];
}
parameters {
   real<lower=min(y),upper=max(y)> phi;
}
```

This declares a positive integer data variable `N`, an array `y` of real-valued data of length `N`, and then a parameter ranging between the minimum and maximum value of `y`. The functions `fmin()` and `fmax()` are minimum and maximum functions for floating point quantities.

22.4. Vector and Matrix Data Types

Values

Vectors, row vectors, and matrices contain real values. Arrays, on the other hand, may contain any kind of value, including integers and structured values like vectors.

Indexing

Vectors and matrices, as well as arrays, are indexed starting from one in Stan. This follows the convention in statistics and linear algebra as well as their implementations in the statistical software packages R, MATLAB, BUGS, and JAGS. General computer programming languages, on the other hand, such as C++ and Python, index arrays starting from zero.

Vectors

Vectors in Stan are column vectors; see the next subsection for information on row vectors. Vectors are declared with a size (i.e., a dimensionality). For example, a 3-dimensional vector is declared with the keyword `vector`, as follows.

```
vector[3] u;
```

Vectors may also be declared with constraints, as in the following declaration of a 3-vector of non-negative values.

```
vector<lower=0>[3] u;
```

Unit Simplexes

A unit simplex is a vector with non-negative values whose entries sum to 1. For instance, $(0.2, 0.3, 0.4, 0.1)^{\top}$ is a unit 4-simplex. Unit simplexes are most often used as parameters in categorical or multinomial distributions, and they are also the sampled variate in a Dirichlet distribution. Simplexes are declared with their full dimensionality. For instance, `theta` is declared to be a unit 5-simplex by

```
simplex[5] theta;
```

Unit simplexes are implemented as vectors and may be assigned to other vectors and vice-versa. Simplex variables, like other constrained variables, are validated to ensure they contain simplex values; for simplexes, this is only done up to a statically specified accuracy threshold ϵ to account for errors arising from floating-point imprecision.

Unit Vectors

A unit vector is a vector with a norm of one. For instance, $(0.5, 0.5, 0.5, 0.5)^{\top}$ is a unit 4-vector. Unit vectors are sometimes used in directional statistics. Unit vectors are declared with their full dimensionality. For instance, `theta` is declared to be a unit 5-vector by

```
unit_vector[5] theta;
```

Unit vectors are implemented as vectors and may be assigned to other vectors and vice-versa. Unit vector variables, like other constrained variables, are validated to ensure that they are indeed unit length; for unit vectors, this is only done up to a statically specified accuracy threshold ϵ to account for errors arising from floating-point imprecision.

Ordered Vectors

An ordered vector type in Stan represents a vector whose entries are sorted in ascending order. For instance, $(-1.3, 2.7, 2.71)^{\top}$ is an ordered 3-vector. Ordered vectors are most often employed as cut points in ordered logistic regression models (see Section 5.6).

The variable `c` is declared as an ordered 5-vector by

```
ordered[5] c;
```

After their declaration, ordered vectors, like unit simplexes, may be assigned to other vectors and other vectors may be assigned to them. Constraints will be checked after executing the block in which the variables were declared.

Positive, Ordered Vectors

There is also a positive, ordered vector type which operates similarly to ordered vectors, but all entries are constrained to be positive. For instance, $(2, 3.7, 4, 12.9)$ is a positive, ordered 4-vector.

The variable d is declared as a positive, ordered 5-vector by

```
positive_ordered[5] d;
```

Like ordered vectors, after their declaration positive ordered vectors assigned to other vectors and other vectors may be assigned to them. Constraints will be checked after executing the block in which the variables were declared.

Row Vectors

Row vectors are declared with the keyword row_vector. Like (column) vectors, they are declared with a size. For example, a 1093-dimensional row vector u would be declared as

```
row_vector[1093] u;
```

Constraints are declared as for vectors, as in the following example of a 10-vector with values between -1 and 1.

```
row_vector<lower=-1,upper=1>[10] u;
```

Row vectors may not be assigned to column vectors, nor may column vectors be assigned to row vectors. If assignments are required, they may be accommodated through the transposition operator.

Matrices

Matrices are declared with the keyword matrix along with a number of rows and number of columns. For example,

```
matrix[3,3] A;
matrix[M,N] B;
```

declares A to be a 3×3 matrix and B to be a $M \times N$ matrix. For the second declaration to be well formed, the variables M and N must be declared as integers in either the data or transformed data block and before the matrix declaration.

Matrices may also be declared with constraints, as in this ($3{\times}4$) matrix of non-positive values.

```
matrix<upper=0>[3,4] B;
```

Assigning to Rows of a Matrix

Rows of a matrix can be assigned by indexing the left-hand side of an assignment statement. For example, this is possible.

```
matrix[M,N] a;
row_vector[N] b;
...
a[1] <- b;
```

This copies the values from row vector b to a[1], which is the first row of the matrix a. If the number of columns in a is not the same as the size of b, a run-time error is raised; the number of rows of a is N, which is also the size of b.

Assignment works by copying values in Stan. That means any subsequent assignment to a[1] does not affect b, nor does an assignment to b affect a.

Correlation Matrices

Matrix variables may be constrained to represent correlation matrices. A matrix is a correlation matrix if it is symmetric and positive definite, has entries between -1 and 1, and has a unit diagonal. Because correlation matrices are square, only one dimension needs to be declared. For example,

```
corr_matrix[3] Sigma;
```

declares Sigma to be a 3×3 correlation matrix.

Correlation matrices may be assigned to other matrices, including unconstrained matrices, if their dimensions match, and vice-versa.

Cholesky Factors of Correlation Matrices

Matrix variables may be constrained to represent the Cholesky factors of a correlation matrix.

A Cholesky factor for a correlation matrix L is a $K \times K$ lower-triangular matrix with positive diagonal entries and rows that are of length 1 (i.e., $\sum_{n=1}^{K} L_{m,n}^2 = 1$). If

L is a Cholesky factor for a correlation matrix, then $L\,L^\top$ is a correlation matrix (i.e., symmetric postive definite with a unit diagonal).

A declaration such as follows.

```
cholesky_factor_corr[K] L;
```

declares `L` to be a Cholesky factor for a `K` by `K` correlation matrix.

Covariance Matrices

Matrix variables may be constrained to represent covariance matrices. A matrix is a covariance matrix if it is symmetric and positive definite. Like correlation matrices, covariance matrices only need a single dimension in their declaration. For instance,

```
cov_matrix[K] Omega;
```

declares `Omega` to be a $K \times K$ covariance matrix, where K is the value of the data variable `K`.

Cholesky Factors of Covariance Matrices

Matrix variables may be constrained to represent the Cholesky factors of a covariance matrix. This is often more convenient or more efficient than representing covariance matrices directly.

A Cholesky factor L is an $M \times N$ lower-triangular matrix (if $m < n$ then $L[m,n] = 0$) with a positive diagonal ($L[k,k] = 0$) and $M \geq N$. If L is a Cholesky factor, then $\Sigma = L\,L^\top$ is a covariance matrix. Furthermore, every covariance matrix has a Cholesky factorization.

The typical case of a square Cholesky factor may be declared with a single dimension,

```
cholesky_factor_cov[4] L;
```

In general, two dimensions may be declared, with the above being equal to `cholesky_factor_cov[4,4]`. The type `cholesky_factor_cov[M,N]` may be used for the general $M \times N$.

Assigning Constrained Variables

Constrained variables of all types may be assigned to other variables of the same unconstrained type and vice-versa. For instance, a variable declared to be

`real<lower=0,upper=1>` could be assigned to a variable declared as `real` and vice-versa. Similarly, a variable declared as `matrix[3,3]` may be assigned to a variable declared as `cov_matrix[3]` or `cholesky_factor_cov[3]`, and vice-versa.

Checks are carried out at the end of each relevant block of statements to ensure constraints are enforced. This includes run-time size checks. The Stan compiler isn't able to catch the fact that an attempt may be made to assign a matrix of one dimensionality to a matrix of mismatching dimensionality.

Expressions as Size Declarations

Variables may be declared with sizes given by expressions. Such expressions are constrained to only contain data or transformed data variables. This ensures that all sizes are determined once the data is read in and transformed data variables defined by their statements. For example, the following is legal.

```
data {
  int<lower=0> N_observed;    int<lower=0> N_missing;
  ...
transformed parameters {
  vector[N_observed + N_missing] y;
  ...
```

Accessing Vector and Matrix Elements

If `v` is a column vector or row vector, then `v[2]` is the second element in the vector. If `m` is a matrix, then `m[2,3]` is the value in the second row and third column.

Providing a matrix with a single index returns the specified row. For instance, if `m` is a matrix, then `m[2]` is the second row. This allows Stan blocks such as

```
matrix[M,N] m;
row_vector[N] v;
real x;
...
v <- m[2];
x <- v[3];   // x == m[2][3] == m[2,3]
```

The type of `m[2]` is `row_vector` because it is the second row of `m`. Thus it is possible to write `m[2][3]` instead of `m[2,3]` to access the third element in the second row. When given a choice, the form `m[2,3]` is preferred.[2]

[2] As of Stan version 1.0, the form `m[2,3]` is more efficient because it does not require the creation and use of an intermediate expression template for `m[2]`. In later versions, explicit calls to `m[2][3]` may be optimized to be as efficient as `m[2,3]` by the Stan compiler.

Size Declaration Restrictions

An integer expression is used to pick out the sizes of vectors, matrices, and arrays. For instance, we can declare a vector of size `M + N` using

```
vector[M + N] y;
```

Any integer-denoting expression may be used for the size declaration, providing all variables involved are either data, transformed data, or local variables. That is, expressions used for size declarations may not include parameters or transformed parameters or generated quantities.

22.5. Array Data Types

Stan supports arrays of arbitrary dimension. An array's elements may be any of the basic data types, that is univariate integers, univariate reals, vectors, row vectors matrices, including all of the constrained forms.

Declaring Array Variables

Arrays are declared by enclosing the dimensions in square brackets following the name of the variable.

The variable `n` is declared as an array of five integers as follows.

```
int n[5];
```

A two-dimensional array of real values with three rows and four columns is declared with the following.

```
real a[3,4];
```

A three-dimensional array `z` of positive reals with five rows, four columns, and two shelves can be declared as follows.

```
real<lower=0> z[5,4,2];
```

Arrays may also be declared to contain vectors. For example,

```
vector[7] mu[3];
```

declares `mu` to be a 3-dimensional array of 7-vectors. Arrays may also contain matrices. The example

```
matrix[7,2] mu[15,12];
```

declares a 15×12-dimensional array of 7×2 matrices. Any of the constrained types may also be used in arrays, as in the declaration

```
cholesky_factor_cov[5,6] mu[2,3,4];
```

of a $2 \times 3 \times 4$ array of 5×6 Cholesky factors of covariance matrices.

Accessing Array Elements and Subarrays

If x is a 1-dimensional array of length 5, then x[1] is the first element in the array and x[5] is the last. For a 3×4 array y of two dimensions, y[1,1] is the first element and y[3,4] the last element. For a three-dimensional array z, the first element is z[1,1,1], and so on.

Subarrays of arrays may be accessed by providing fewer than the full number of indexes. For example, suppose y is a two-dimensional array with three rows and four columns. Then y[3] is one-dimensional array of length four. This means that y[3][1] may be used instead of y[3,1] to access the value of the first column of the third row of y. The form y[3,1] is the preferred form (see Footnote 2 in this chapter).

Subarrays may be manipulated and assigned just like any other variables. Similar to the behavior of matrices, Stan allows blocks such as

```
real w[9,10,11];
real x[10,11];
real y[11];
real z;
...
x <- w[5];
y <- x[4];  // y == w[5][4] == w[5,4]
z <- y[3];  // z == w[5][4][3] == w[5,4,3]
```

Assigning

—fixme—

Arrays of Matrices and Vectors

Arrays of vectors and matrices are accessed in the same way as arrays of doubles. Consider the following vector and scalar declarations.

```
vector[5] a[4,3];
vector[5] b[4];
```

```
vector[5] c;
real x;
```

With these declarations, the following assignments are legal.

```
b <- a[1];      // result is array of vectors
c <- a[1,3];    // result is vector
c <- b[3];      //   same result as above
x <- a[1,3,5];  // result is scalar
x <- b[3,5];    //   same result as above
x <- c[5];      //   same result as above
```

Row vectors and other derived vector types (simplex and ordered) behave the same way in terms of indexing.

Consider the following matrix, vector and scalar declarations.

```
matrix[6,5] d[3,4];
matrix[6,5] e[4];
matrix[6,5] f;
row_vector[5] g;
real x;
```

With these declarations, the following definitions are legal.

```
e <- d[1];        // result is array of matrices
f <- d[1,3];      // result is matrix
f <- e[3];        //   same result as above
g <- d[1,3,2];    // result is row vector
g <- e[3,2];      //   same result as above
g <- f[2];        //   same result as above
x <- d[1,3,5,2];  // result is scalar
x <- e[3,5,2];    //   same result as above
x <- f[5,2];      //   same result as above
x <- g[2];        //   same result as above
```

As shown, the result `f[2]` of supplying a single index to a matrix is the indexed row, here row 2 of matrix `f`.

Partial Array Assignment

Subarrays of arrays may be assigned by indexing on the left-hand side of an assignment statement. For example, the following is legal.

```
real x[I,J,K];
real y[J,K];
real z[K];
...
x[1] <- y;
x[1,1] <- z;
```

The sizes must match. Here, `x[1]` is a `J` by `K` array, as is is `y`.

Partial array assignment also works for arrays of matrices, vectors, and row vectors.

Mixing Array, Vector, and Matrix Types

Arrays, row vectors, column vectors and matrices are not interchangeable in Stan. Thus a variable of any one of these fundamental types is not assignable to any of the others, nor may it be used as an argument where the other is required (use as arguments follows the assignment rules).

Mixing Vectors and Arrays

For example, vectors cannot be assigned to arrays or vice-versa.

```
real a[4];
vector b[4];
row_vector c[4];
...
a <- b; // illegal assignment of vector to array
b <- a; // illegal assignment of array to vector
a <- c; // illegal assignment of row vector to array
c <- a; // illegal assignment of array to row vector
```

Mixing Row and Column Vectors

It is not even legal to assign row vectors to column vectors or vice versa.

```
vector b[4];
row_vector c[4];
...
b <- c; // illegal assignment of row vector to column vector
c <- b; // illegal assignment of column vector to row vector
```

Mixing Matrices and Arrays

The same holds for matrices, where 2-dimensional arrays may not be assigned to matrices or vice-versa.

```
real a[3,4];
matrix[3,4] b;
...
a <- b;  // illegal assignment of matrix to array
b <- a;  // illegal assignment of array to matrix
```

Mixing Matrices and Vectors

A $1 \times N$ matrix cannot be assigned a row vector or vice versa.

```
matrix[1,4] a;
row_vector[4] b;
...
a <- b;  // illegal assignment of row vector to matrix
b <- a;  // illegal assignment of matrix to row vector
```

Similarly, an $M \times 1$ matrix may not be assigned to a column vector.

```
matrix[4,1] a;
vector[4] b;
...
a <- b;  // illegal assignment of column vector to matrix
b <- a;  // illegal assignment of matrix to column vector
```

Size Declaration Restrictions

An integer expression is used to pick out the sizes of arrays. The same restrictions as for vector and matrix sizes apply, namely that the size is declared with an integer-denoting expression that does not contain any parameters, transformed parameters, or generated quantities.

22.6. Variable Types vs. Constraints and Sizes

The type information associated with a variable only contains the underlying type and dimensionality of the variable.

Type Information Excludes Sizes

The size associated with a given variable is not part of its data type. For example, declaring a variable using

```
real a[3];
```

declares the variable `a` to be an array. The fact that it was declared to have size 3 is part of its declaration, but not part of its underlying type.

When are Sizes Checked?

Sizes are determined dynamically (at run time) and thus cannot be type-checked statically when the program is compiled. As a result, any conformance error on size will raise a run-time error. For example, trying to assign an array of size 5 to an array of size 6 will cause a run-time error. Similarly, multiplying an $N \times M$ by a $J \times K$ matrix will raise a run-time error if $M \neq J$.

Type Information Excludes Constraints

Like sizes, constraints are not treated as part of a variable's type in Stan when it comes to the compile-time check of operations it may participate in. Anywhere Stan accepts a matrix as an argument, it will syntactically accept a correlation matrix or covariance matrix or Cholesky factor. Thus a covariance matrix may be assigned to a matrix and vice-versa.

Similarly, a bounded real may be assigned to an unconstrained real and vice-versa.

When are Function Argument Constraints Checked?

For arguments to functions, constraints are sometimes, but not always checked when the function is called. Exclusions include C++ standard library functions. All probability functions and cumulative distribution functions check that their arguments are appropriate at run time as the function is called.

When are Declared Variable Constraints Checked?

For data variables, constraints are checked after the variable is read from a data file or other source. For transformed data variables, the check is done after the statements in the transformed data block have executed. Thus it is legal for intermediate values of variables to not satisfy declared constraints.

For parameters, constraints are enforced by the transform applied and do not need to be checked. For transformed parameters, the check is done after the statements in the transformed parameter block have executed.

For generated quantities, constraints are enforced after the statements in the generated quantities block have executed.

Type Naming Notation

In order to refer to data types, it is convenient to have a way to refer to them. The type naming notation outlined in this section is not part of the Stan programming language, but rather a convention adopted in this document to enable a concise description of a type.

Because size information is not part of a data type, data types will be written without size information. For instance, `real[]` is the type of one-dimensional array of reals and `matrix` is the type of matrices. The three-dimensional integer array type is written as `int[ ,  ,]`, indicating the number slots available for indexing. Similarly, `vector[,]` is the type of a two-dimensional array of vectors.

23. Expressions

An expression is the basic syntactic unit in a Stan program that denotes a value. Every expression in a well-formed Stan program has a type that is determined statically (at compile time). If an expressions type cannot be determined statically, the Stan compiler will report the location of the problem.

This chapter covers the syntax, typing, and usage of the various forms of expressions in Stan.

23.1. Numeric Literals

The simplest form of expression is a literal that denotes a primitive numerical value.

Integer Literals

Integer literals represent integers of type `int`. Integer literals are written in base 10 without any separators. Integer literals may contain a single negative sign. (The expression `--1` is interpreted as the negation of the literal `-1`.)

The following list contains well-formed integer literals.

```
0, 1, -1, 256, -127098, 24567898765
```

Integer literals must have values that fall within the bounds for integer values (see Section 22.2).

Integer literals may not contain decimal points (`.`). Thus the expressions `1.` and `1.0` are of type `real` and may not be used where a value of type `int` is required.

Real Literals

A number written with a period or with scientific notation is assigned to a the continuous numeric type `real`. Real literals are written in base 10 with a period (`.`) as a separator. Examples of well-formed real literals include the following.

```
0.0, 1.0, 3.14, -217.9387, 2.7e3, -2E-5
```

The notation `e` or `E` followed by a positive or negative integer denotes a power of 10 to multiply. For instance, `2.7e3` denotes 2.7×10^3 and `-2E-5` denotes -2×10^{-5}.

23.2. Variables

A variable by itself is a well-formed expression of the same type as the variable. Variables in Stan consist of ASCII strings containing only the basic lower-case and

upper-case Roman letters, digits, and the underscore (_) character. Variables must start with a letter (a-z and A-Z) and may not end with two underscores (__).

Examples of legal variable identifiers are as follows.

a, a3, a_3, Sigma, my_cpp_style_variable, myCamelCaseVariable

Unlike in R and BUGS, variable identifiers in Stan may not contain a period character.

Reserved Names

Stan reserves many strings for internal use and these may not be used as the name of a variable. An attempt to name a variable after an internal string results in the stanc translator halting with an error message indicating which reserved name was used and its location in the model code.

Model Name

The name of the model cannot be used as a variable within the model. This is usually not a problem because the default in bin/stanc is to append _model to the name of the file containing the model specification. For example, if the model is in file foo.stan, it would not be legal to have a variable named foo_model when using the default model name through bin/stanc. With user-specified model names, variables cannot match the model.

User-Defined Function Names

User-defined function names cannot be used as a variable within the model.

Reserved Words from Stan Language

The following list contains reserved words for Stan's programming language. Not all of these features are implemented in Stan yet, but the tokens are reserved for future use.

for, in, while, repeat, until, if, then, else, true, false

Variables should not be named after types, either, and thus may not be any of the following.

int, real, vector, simplex, unit_vector, ordered, positive_ordered, row_vector, matrix, cholesky_factor_corr, cholesky_factor_cov, corr_matrix, cov_matrix.

Variable names will *not* conflict with the following block identifiers,

functions, model, data, parameters, quantities, transformed, generated,

Reserved Names from Stan Implementation

Some variable names are reserved because they are used within Stan's C++ implementation. These are

var, fvar

Reserved Function and Distribution Names

Variable names will conflict with the names of predefined functions other than constants. Thus a variable may not be named logit or add, but it may be named pi or e.

Variable names will also conflict with the names of distributions suffixed with _log, _cdf, _cdf_log, and _ccdf_log, such as normal_cdf_log.

Using any of these variable names causes the stanc translator to halt and report the name and location of the variable causing the conflict.

Reserved Names from C++

Finally, variable names, including the names of models, should not conflict with any of the C++ keywords.

alignas, alignof, and, and_eq, asm, auto, bitand, bitor, bool, break, case, catch, char, char16_t, char32_t, class, compl, const, constexpr, const_cast, continue, decltype, default, delete, do, double, dynamic_cast, else, enum, explicit, export, extern, false, float, for, friend, goto, if, inline, int, long, mutable, namespace, new, noexcept, not, not_eq, nullptr, operator, or, or_eq, private, protected, public, register, reinterpret_cast, return, short, signed, sizeof, static, static_assert, static_cast, struct, switch, template, this, thread_local, throw, true, try, typedef, typeid, typename, union, unsigned, using, virtual, void, volatile, wchar_t, while, xor, xor_eq

Legal Characters

The legal variable characters have the same ASCII code points in the range 0–127 as in Unicode.

Characters	ASCII (Unicode) Code Points
a - z	97 - 122
A - Z	65 - 90
0 - 9	48 - 57
_	95

Although not the most expressive character set, ASCII is the most portable and least prone to corruption through improper character encodings or decodings.

Comments Allow ASCII-Compatible Encoding

Within comments, Stan can work with any ASCII-compatible character encoding, such as ASCII itself, UTF-8, or Latin1. It is up to user shells and editors to display them properly.

23.3. Parentheses for Grouping

Any expression wrapped in parentheses is also an expression. Like in C++, but unlike in R, only the round parentheses, `(` and `)`, are allowed. The square brackets `[` and `]` are reserved for array indexing and the curly braces `{` and `}` for grouping statements.

With parentheses it is possible to explicitly group subexpressions with operators. Without parentheses, the expression `1 + 2 * 3` has a subexpression `2 * 3` and evaluates to 7. With parentheses, this grouping may be made explicit with the expression `1 + (2 * 3)`. More importantly, the expression `(1 + 2) * 3` has `1 + 2` as a subexpression and evaluates to 9.

23.4. Arithmetic and Matrix Expressions

For integer and real-valued expressions, Stan supports the basic binary arithmetic operations of addition (+), subtraction (-), multiplication (*) and division (/) in the usual ways.

For integer expressions, Stan supports the modulus (%) binary arithmetic operation. Stan also supports the unary operation of negation for integer and real-valued expressions. For example, assuming `n` and `m` are integer variables and `x` and `y` real variables, the following expressions are legal.

```
3.0 + 0.14,  -15,  2 * 3 + 1,  (x - y) / 2.0,
(n * (n + 1)) / 2,  x / n,  m % n
```

The negation, addition, subtraction, and multiplication operations are extended to matrices, vectors, and row vectors. The transpose operation, written using an apostrophe (`'`) is also supported for vectors, row vectors, and matrices. Return types for

matrix operations are the smallest types that can be statically guaranteed to contain the result. The full set of allowable input types and corresponding return types is detailed in Chapter 33.

For example, if y and mu are variables of type vector and Sigma is a variable of type matrix, then

```
(y - mu)' * Sigma * (y - mu)
```

is a well-formed expression of type real. The type of the complete expression is inferred working outward from the subexpressions. The subexpression(s) y - mu are of type vector because the variables y and mu are of type vector. The transpose of this expression, the subexpression (y - mu)' is of type row_vector. Multiplication is left associative and transpose has higher precedence than multiplication, so the above expression is equivalent to the following well-formed, fully specified form.

```
(((y - mu)') * Sigma) * (y - mu)
```

The type of subexpression (y - mu)' * Sigma is inferred to be row_vector, being the result of multiplying a row vector by a matrix. The whole expression's type is thus the type of a row vector multiplied by a (column) vector, which produces a real value.

Stan provides elementwise matrix division and multiplication operations, a .* b and a ./b. These provide a shorthand to replace loops, but are not intrinsicially more efficient than a version programmed with an elementwise calculations and assignments in a loop. For example, given declarations

```
vector[N] a;
vector[N] b;
vector[N] c;
```

, the assignment

```
c <- a .* b;
```

produces the same result with roughly the same efficiency as the loop

```
for (n in 1:N)
  c[n] <- a[n] * b[n];
```

Stan supports exponentiation (^) of integer and real-valued expressions. The return type of exponentiation is always a real-value. For example, assuming n and m are integer variables and x and y real variables, the following expressions are legal.

```
3 ^ 2, 3.0 ^ -2, 3.0 ^ 0.14,
x ^ n, n ^ x, n ^ m, x ^ y
```

Exponentiation is right associative, so the expression

```
2 ^ 3 ^ 4
```

is equivalent to the following well-formed, fully specified form.

```
2 ^ (3 ^ 4)
```

Operator Precedence and Associativity

The precedence and associativity of operators, as well as built-in syntax such as array indexing and function application is given in tabular form in Figure 23.1. Other expression-forming operations, such as function application and subscripting bind more tightly than any of the arithmetic operations.

The precedence and associativity determine how expressions are interpreted. Because addition is left associative, the expression `a+b+c` is interpreted as `(a+b)+c`. Similarly, `a/b*c` is interpreted as `(a/b)*c`.

Because multiplication has higher precedence than addition, the expression `a*b+c` is interpreted as `(a*b)+c` and the expression `a+b*c` is interpreted as `a+(b*c)`. Similarly, `2*x+3*-y` is interpreted as `(2*x)+(3*(-y))`.

Transposition and exponentiation bind more tightly than any other arithmetic or logical operation. For vectors, row vectors, and matrices, `-u'` is interpreted as `-(u')`, `u*v'` as `u*(v')`, and `u'*v` as `(u')*v`. For integer and reals, `-n ^ 3` is interpreted as `-(n ^ 3)`.

23.5. Subscripting

Stan arrays, matrices, vectors, and row vectors are all accessed using the same array-like notation. For instance, if `x` is a variable of type `real[]` (a one-dimensional array of reals) then `x[1]` is the value of the first element of the array.

Subscripting has higher precedence than any of the arithmetic operations. For example, `alpha*x[1]` is equivalent to `alpha*(x[1])`.

Multiple subscripts may be provided within a single pair of square brackets. If `x` is of type `real[ , ]`, a two-dimensional array, then `x[2,501]` is of type `real`.

Accessing Subarrays

The subscripting operator also returns subarrays of arrays. For example, if `x` is of type `real[ , , ]`, then `x[2]` is of type `real[ , ]`, and `x[2,3]` is of type `real[]`. As a result, the expressions `x[2,3]` and `x[2][3]` have the same meaning.

Op.	*Prec.*	*Assoc.*	*Placement*	*Description*
`||`	9	left	binary infix	logical or
`&&`	8	left	binary infix	logical and
`==`	7	left	binary infix	equality
`!=`	7	left	binary infix	inequality
`<`	6	left	binary infix	less than
`<=`	6	left	binary infix	less than or equal
`>`	6	left	binary infix	greater than
`>=`	6	left	binary infix	greater than or equal
`+`	5	left	binary infix	addition
`-`	5	left	binary infix	subtraction
`*`	4	left	binary infix	multiplication
`/`	4	left	binary infix	(right) division
`%`	4	left	binary infix	modulus
`\`	3	left	binary infix	left division
`.*`	2	left	binary infix	elementwise multiplication
`./`	2	left	binary infix	elementwise division
`!`	1	n/a	unary prefix	logical negation
`-`	1	n/a	unary prefix	negation
`+`	1	n/a	unary prefix	promotion (no-op in Stan)
`^`	0.5	right	binary infix	exponentiation
`'`	0	n/a	unary postfix	transposition
`()`	0	n/a	prefix, wrap	function application
`[]`	0	left	prefix, wrap	array, matrix indexing

Figure 23.1: *Stan's unary and binary operators, with their precedences, associativities, place in an expression, and a description. The last two lines list the precedence of function application and array, matrix, and vector indexing. The operators are listed in order of precedence, from least tightly binding to most tightly binding. The full set of legal arguments and corresponding result types are provided in the function documentation in Part IV prefaced with* `operator` *(i.e.,* `operator*(int,int):int` *indicates the application of the multiplication operator to two integers, which returns an integer). Parentheses may be used to group expressions explicitly rather than relying on precedence and associativity.*

Accessing Matrix Rows

If Sigma is a variable of type matrix, then Sigma[1] denotes the first row of Sigma and has the type row_vector.

Mixing Array and Vector/Matrix Indexes

Stan supports mixed indexing of arrays and their vector, row vector or matrix values. For example, if m is of type matrix[,], a two-dimensional array of matrices, then m[1] refers to the first row of the array, which is a one-dimensional array of matrices. More than one index may be used, so that m[1,2] is of type matrix and denotes the matrix in the first row and second column of the array. Continuing to add indices, m[1,2,3] is of type row_vector and denotes the third row of the matrix denoted by m[1,2]. Finally, m[1,2,3,4] is of type real and denotes the value in the third row and fourth column of the matrix that is found at the first row and second column of the array m.

23.6. Function Application

Stan provides a range of built in mathematical and statistical functions, which are documented in Part IV.

Expressions in Stan may consist of the name of function followed by a sequence of zero or more argument expressions. For instance, log(2.0) is the expression of type real denoting the result of applying the natural logarithm to the value of the real literal 2.0.

Syntactically, function application has higher precedence than any of the other operators, so that y + log(x) is interpreted as y + (log(x)).

Type Signatures and Result Type Inference

Each function has a type signature which determines the allowable type of its arguments and its return type. For instance, the function signature for the logarithm function can be expressed as

```
real log(real);
```

and the signature for the multiply_log function is

```
real multiply_log(real,real);
```

A function is uniquely determined by its name and its sequence of argument types. For instance, the following two functions are different functions.

```
real mean(real[]);
real mean(vector);
```

The first applies to a one-dimensional array of real values and the second to a vector.

The identity conditions for functions explicitly forbids having two functions with the same name and argument types but different return types. This restriction also makes it possible to infer the type of a function expression compositionally by only examining the type of its subexpressions.

Constants

Constants in Stan are nothing more than nullary (no-argument) functions. For instance, the mathematical constants π and e are represented as nullary functions named `pi()` and `e()`. See Section 31.1 for a list of built-in constants.

Type Promotion and Function Resolution

Because of integer to real type promotion, rules must be established for which function is called given a sequence of argument types. The scheme employed by Stan is the same as that used by C++, which resolves a function call to the function requiring the minimum number of type promotions.

For example, consider a situation in which the following two function signatures have been registered for `foo`.

```
real foo(real,real);
int foo(int,int);
```

The use of `foo` in the expression `foo(1.0,1.0)` resolves to `foo(real,real)`, and thus the expression `foo(1.0,1.0)` itself is assigned a type of `real`.

Because integers may be promoted to real values, the expression `foo(1,1)` could potentially match either `foo(real,real)` or `foo(int,int)`. The former requires two type promotions and the latter requires none, so `foo(1,1)` is resolved to function `foo(int,int)` and is thus assigned the type `int`.

The expression `foo(1,1.0)` has argument types `(int,real)` and thus does not explicitly match either function signature. By promoting the integer expression `1` to type `real`, it is able to match `foo(real,real)`, and hence the type of the function expression `foo(1,1.0)` is `real`.

In some cases (though not for any built-in Stan functions), a situation may arise in which the function referred to by an expression remains ambiguous. For example, consider a situation in which there are exactly two functions named `bar` with the following signatures.

```
real bar(real,int);
real bar(int,real);
```

With these signatures, the expression `bar(1.0,1)` and `bar(1,1.0)` resolve to the first and second of the above functions, respectively. The expression `bar(1.0,1.0)` is illegal because real values may not be demoted to integers. The expression `bar(1,1)` is illegal for a different reason. If the first argument is promoted to a real value, it matches the first signature, whereas if the second argument is promoted to a real value, it matches the second signature. The problem is that these both require one promotion, so the function name `bar` is ambiguous. If there is not a unique function requiring fewer promotions than all others, as with `bar(1,1)` given the two declarations above, the Stan compiler will flag the expression as illegal.

Random-Number Generating Functions

For most of the distributions supported by Stan, there is a corresponding random-number generating function. These random number generators are named by the distribution with the suffix `_rng`. For example, a univariate normal random number can be generated by `normal_rng(0,1)`; only the parameters of the distribution, here a location (0) and scale (1) are specified because the variate is generated.

Random-Number Generators Restricted to Generated Quantities Block

The use of random-number generating functions is restricted to the generated quantities block; attempts to use them elsewhere will result in a parsing error with a diagnostic message.

This allows the random number generating functions to be used for simulation in general, and for Bayesian posterior predictive checking in particular.

Posterior Predictive Checking

Posterior predictive checks typically use the parameters of the model to generate simulated data (at the individual and optionally at the group level for hierarchical models), which can then be compared informally using plots and formally by means of test statistics, to the actual data in order to assess the suitability of the model; see (Gelman et al., 2013, Chapter 6) for more information on posterior predictive checks.

23.7. ODE Solver

Stan provides a built-in ordinary differential equation solver. Although it looks like a function application, it is special in two ways.

First, like PKBugs (Lunn et al., 1999), Stan's ODE solver takes a function specifying the system of equations as an argument. Ordinary Stan functions do not allow functions as arguments.

Second, the ODE solver expression restricts certain of its arguments to be data only expressions (i.e., they must not contain variables other than those declared in the data or transformed data block). Ordinary Stan functions place no restriction on the origin of variables in their argument expressions.

Arguments

The arguments to the ODE solver function `integrate_ode` are as follows:

1. Function literal referring to a function specifying the system of differential equations with signature

   (real,real[],real[],real[],int[]):real[]

   The arguments represent (1) time, (2) system state, (3) parameters, (4) real data, and (5) integer data, and the return value contains the derivatives with respect to time of the state,

2. initial state, type `real[]`,

3. initial time, type `int` or `real`, data only,

4. solution times, type `real[]`, data only,

5. parameters, type `real[]`,

6. real data, type `real[]`, data only, and

7. integer data, type `int[]`, data only.

Return Values

The return value for the ODE solver is an array of type `real[,]`, with values consisting of solutions at the specified times.

Sizes and Parallel Arrays

The sizes must match, and in particular, the following groups are of the same size:

- state variables passed into the system function, derivatives returned by the system function, initial state passed into the solver, and rows of the return value of the solver,

- solution times and number of rows of the return value of the solver,
- parameters, real data and integer data passed to the solver will be passed to the system function

Example

An example of a complete Stan program with a system definition and solver call is shown for data simulation in Figure 17.2 and estimation in Figure 17.3.

Gradients

Gradients are calculated by integrating the coupled system consisting of the original system state and derivatives of the original system with respect to the parameters and initial state. The additional derivatives required for the coupled system are calculated by automatically differentiating the system with respect to the parameters.

23.8. Type Inference

Stan is strongly statically typed, meaning that the implementation type of an expression can be resolved at compile time.

Implementation Types

The primitive implementation types for Stan are `int`, `real`, `vector`, `row_vector`, and `matrix`. Every basic declared type corresponds to a primitive type; see Figure 23.2 for the mapping from types to their primitive types. A full implementation type consists of a primitive implementation type and an integer array dimensionality greater than or equal to zero. These will be written to emphasize their array-like nature. For example, `int[]` has an array dimensionality of 1, `int` an array dimensionality of 0, and `int[,,]` an array dimensionality of 3. The implementation type `matrix[,,]` has a total of five dimensions and takes up to five indices, three from the array and two from the matrix.

Recall that the array dimensions come before the matrix or vector dimensions in an expression such as the following declaration of a three-dimensional array of matrices.

```
matrix[M,N] a[I,J,K];
```

The matrix `a` is indexed as `a[i,j,k,m,n]` with the array indices first, followed by the matrix indices, with `a[i,j,k]` being a matrix and `a[i,j,k,m]` being a row vector.

Type	*Primitive Type*
`int`	`int`
`real`	`real`
`matrix`	`matrix`
`cov_matrix`	`matrix`
`corr_matrix`	`matrix`
`cholesky_factor_cov`	`matrix`
`cholesky_factor_corr`	`matrix`

Type	*Primitive Type*
`vector`	`vector`
`simplex`	`vector`
`unit_vector`	`vector`
`ordered`	`vector`
`positive_ordered`	`vector`
`row_vector`	`row_vector`

Figure 23.2: *The table shows the variable declaration types of Stan and their corresponding primitive implementation type. Stan functions, operators and probability functions have argument and result types declared in terms of primitive types.*

Type Inference Rules

Stan's type inference rules define the implementation type of an expression based on a background set of variable declarations. The rules work bottom up from primitive literal and variable expressions to complex expressions.

Literals

An integer literal expression such as `42` is of type `int`. Real literals such as `42.0` are of type `real`.

Variables

The type of a variable declared locally or in a previous block is determined by its declaration. The type of a loop variable is `int`.

There is always a unique declaration for each variable in each scope because Stan prohibits the redeclaration of an already-declared variables.[1]

[1]Languages such as C++ and R allow the declaration of a variable of a given name in a narrower scope

Indexing

If `x` is an expression of total dimensionality greater than or equal to N, then the type of expression `e[i1,...,iN]` is the same as that of `e[i1]...[iN]`, so it suffices to define the type of a singly-indexed function. Suppose `e` is an expression and `i` is an expression of primitive type `int`. Then

- if `e` is an expression of array dimensionality $K > 0$, then `e[i]` has array dimensionality $K - 1$ and the same primitive implementation type as `e`,
- if `e` has implementation type `vector` or `row_vector` of array dimensionality 0, then `e[i]` has implementation type `real`, and
- if `e` has implementation type `matrix`, then `e[i]` has type `row_vector`.

Function Application

If `f` is the name of a function and `e1,...,eN` are expressions for $N \geq 0$, then `f(e1,...,eN)` is an expression whose type is determined by the return type in the function signature for `f` given `e1` through `eN`. Recall that a function signature is a declaration of the argument types and the result type.

In looking up functions, binary operators like `real * real` are defined as `operator*(real,real)` in the documentation and index.

In matching a function definition, arguments of type `int` may be promoted to type `real` if necessary (see the subsection on type promotion in Section 23.6 for an exact specification of Stan's integer-to-real type-promotion rule).

In general, matrix operations return the lowest inferrable type. For example, `row_vector * vector` returns a value of type `real`, which is declared in the function documentation and index as `real operator*(row_vector,vector)`.

23.9. Chain Rule and Derivatives

Derivatives of the log probability function defined by a model are used in several ways by Stan. The Hamiltonian Monte Carlo samplers, including NUTS, use gradients to guide updates. The BFGS optimizers also use gradients to guide search for posterior modes.

Errors Due to Chain Rule

Unlike evaluations in pure mathematics, evaluation of derivatives in Stan is done by applying the chain rule on an expression-by-expression basis, evaluating using

to hide (take precedence over for evaluation) a variable defined in a containing scope.

floating-point arithmetic. As a result, models such as the following are problematic for inference involving derivatives.

```
parameters {
  real x;
}
model {
  x ~ normal(sqrt(x - x), 1);
}
```

Algebraically, the sampling statement in the model could be reduced to

```
  x ~ normal(0, 1);
```

and it would seem the model should produce unit normal samples for `x`. But rather than cancelling, the expression `sqrt(x - x)` causes a problem for derivatives. The cause is the mechanistic evaluation of the chain rule,

$$
\begin{aligned}
\frac{d}{dx}\sqrt{x-x} &= \frac{1}{2\sqrt{x-x}} \times \frac{d}{dx}(x-x) \\
&= \frac{1}{0} \times (1-1) \\
&= \infty \times 0 \\
&= \text{NaN}.
\end{aligned}
$$

Rather than the $x - x$ cancelling out, it introduces a 0 into the numerator and denominator of the chain-rule evaluation.

The only way to avoid this kind problem is to be careful to do the necessary algebraic reductions as part of the model and not introduce expressions like `sqrt(x - x)` for which the chain rule produces not-a-number values.

Diagnosing Problems with Derivatives

The best way to diagnose whether something is going wrong with the derivatives is to use the test-gradient option to the sampler or optimizer inputs; this option is available in both Stan and RStan (though it may be slow, because it relies on finite differences to make a comparison to the built-in automatic differentiation).

For example, compiling the above model to an executable `sqrt-x-minus-x`, the test can be run as

```
> ./sqrt-x-minus-x diagnose test=gradient
```

```
...
TEST GRADIENT MODE

 Log probability=-0.393734

 param idx           value           model     finite diff           error
         0       -0.887393             nan               0             nan
```

Even though finite differences calculates the right gradient of 0, automatic differentiation follows the chain rule and produces a not-a-number output.

24. Statements

The blocks of a Stan program (see Chapter 26) are made up of variable declarations and statements. Unlike programs in BUGS, the declarations and statements making up a Stan program are executed in the order in which they are written. Variables must be defined to have some value (as well as declared to have some type) before they are used — if they do not, the behavior is undefined.

Like BUGS, Stan has two kinds of atomic statements, assignment statements and sampling statements. Also like BUGS, statements may be grouped into sequences and into for-each loops. In addition, Stan allows local variables to be declared in blocks and also allows an empty statement consisting only of a semicolon.

24.1. Assignment Statement

An assignment statement consists of a variable (possibly multivariate with indexing information) and an expression. Executing an assignment statement evaluates the expression on the right-hand side and assigns it to the (indexed) variable on the left-hand side. An example of a simple assignment is

```
n <- 0;
```

Executing this statement assigns the value of the expression `0`, which is the integer zero, to the variable `n`. For an assignment to be well formed, the type of the expression on the right-hand side should be compatible with the type of the (indexed) variable on the left-hand side. For the above example, because `0` is an expression of type `int`, the variable `n` must be declared as being of type `int` or of type `real`. If the variable is of type `real`, the integer zero is promoted to a floating-point zero and assigned to the variable. After the assignment statement executes, the variable `n` will have the value zero (either as an integer or a floating-point value, depending on its type).

Syntactically, every assignment statement must be followed by a semicolon. Otherwise, whitespace between the tokens does not matter (the tokens here being the left-hand-side (indexed) variable, the assignment operator, the right-hand-side expression and the semicolon).

Because the right-hand side is evaluated first, it is possible to increment a variable in Stan just as in C++ and other programming languages by writing

```
n <- n + 1;
```

Such self assignments are not allowed in BUGS, because they induce a cycle into the directed graphical model.

The left-hand side of an assignment may contain indices for array, matrix, or vector data structures. For instance, if `Sigma` is of type `matrix`, then

```
Sigma[1,1] <- 1.0;
```

sets the value in the first column of the first row of Sigma to one.

Assignments can involve complex objects of any type. If Sigma and Omega are matrices and sigma is a vector, then the following assignment statement, in which the expression and variable are both of type matrix, is well formed.

```
Sigma
  <- diag_matrix(sigma)
     * Omega
     * diag_matrix(sigma);
```

This example also illustrates the preferred form of splitting a complex assignment statement and its expression across lines.

Assignments to subcomponents of larger multi-variate data structures are supported by Stan. For example, a is an array of type real[,] and b is an array of type real[], then the following two statements are both well-formed.

```
a[3] <- b;
b <- a[4];
```

Similarly, if x is a variable declared to have type row_vector and Y is a variable declared as type matrix, then the following sequence of statements to swap the first two rows of Y is well formed.

```
x <- Y[1];
Y[1] <- Y[2];
Y[2] <- x;
```

Lvalue Summary

The expressions that are legal left-hand sides of assignment statements are known as "lvalues." In Stan, there are only two kinds of legal lvalues,

- a variable, or
- a variable with one or more indices.

To be used as an lvalue, an indexed variable must have at least as many dimensions as the number of indices provided. An array of real or integer types has as many dimensions as it is declared for. A matrix has two dimensions and a vector or row vector one dimension; this also holds for the constrained types, covariance and correlation matrices and their Cholesky factors and ordered, positive ordered, and simplex vectors. An array of matrices has two more dimensions than the array and an array of

vectors or row vectors has one more dimension than the array. Note that the number of indices can be less than the number of dimensions of the variable, meaning that the right hand side must itself be multidimensional to match the remaining dimensions.

24.2. Log Probability Increment Statement

The basis of Stan's execution is the evaluation of a log probability function for a given set of parameters. Data and transformed data are fixed before log probability is involved. Statements in the transformed parameter block and model block can have an effect on the log probability function defined by a model.

The total log probability is initialized to zero. Then statements in the transformed parameter block and model block may add to it. The most direct way this is done is through the log probability increment statement, which is of the following form.

```
increment_log_prob(-0.5 * y * y);
```

In this example, the unnormalized log probability of a unit normal variable y is added to the total log probability. In the general case, the argument can be any expression.[1]

An entire Stan model can be implemented this way. For instance, the following model will draw a single variable according to a unit normal probability.

```
parameters {
  real y;
}
model {
  increment_log_prob(-0.5 * y * y);
}
```

This model defines a log probability function

$$\log p(y) = -\frac{y^2}{2} - \log Z$$

where Z is a normalizing constant that does not depend on y. The constant Z is conventionally written this way because on the linear scale,

$$p(y) = \frac{1}{Z} \exp\left(-\frac{y^2}{2}\right).$$

[1] Writing this model with the expression `-0.5 * y * y` is more efficient than with the equivalent expression `y * y / -2` because multiplication is more efficient than division; in both cases, the negation is rolled into the numeric literal (`-0.5` and `-2`). Writing `square(y)` instead of `y * y` would be even more efficient because the derivatives can be precomputed, reducing the memory and number of operations required for automatic differentiation.

which is typically written without reference to Z as

$$p(y) \propto \exp\left(-\frac{y^2}{2}\right).$$

Stan only requires models to be defined up to a constant that does not depend on the parameters. This is convenient because often the normalizing constant Z is either time-consuming to compute or intractable to evaluate.

Vectorization

The `increment_log_prob` function accepts parameters of any expression type, including integers, reals, vectors, row vectors, matrices, and arrays of any dimensionality, including arrays of vectors and matrices.

Log Probability Variable `lp__`

Before version 2.0 of Stan, rather than writing

```
increment_log_prob(u);
```

it was necessary to manipulate a special variable `lp__` as follows

```
lp__ <- lp__ + u;
```

The special variable `lp__` refers to the log probability value that will be returned by Stan's log probability function.

Deprecation of `lp__`

As of Stan version 2.0, the use of `lp__` is deprecated. It will be removed altogether in a future release, but until that time, programs still work with `lp__`. A deprecation warning is now printed every time `lp__` is used.

To access the value of `lp__` for printing, use the `get_lp` function (see Section 31.3).

24.3. Sampling Statements

Like BUGS and JAGS, Stan supports probability statements in sampling notation, such as

```
y ~ normal(mu,sigma);
```

The name "sampling statement" is meant to be suggestive, not interpreted literally. Conceptually, the variable y, which may be an unknown parameter or known, modeled data, is being declared to have the distribution indicated by the right-hand side of the sampling statement.

Executing such a statement does not perform any sampling. In Stan, a sampling statement is merely a notational convenience. The above sampling statement could be expressed as a direct increment on the total log probability as

```
increment_log_prob(normal_log(y,mu,sigma));
```

See the subsection of Section 24.3 discussing log probability increments for a full explanation.

In general, a sampling statement of the form

```
ex0 ~ dist(ex1,...,exN);
```

involving subexpressions ex0 through exN (including the case where N is zero) will be well formed if and only if the corresponding assignment statement is well-formed,

```
increment_log_prob(dist_log(ex0,ex1,...,exN));
```

This will be well formed if and only if dist_log(ex0,ex1,...,exN) is a well-formed function expression of type real.

Log Probability Increment vs. Sampling Statement

Although both lead to the same sampling behavior in Stan, there is one critical difference between using the sampling statement, as in

```
y ~ normal(mu,sigma);
```

and explicitly incrementing the log probability function, as in

```
increment_log_prob(normal_log(y,mu,sigma));
```

The sampling statement drops all the terms in the log probability function that are constant, whereas the explicit call to normal_log adds all of the terms in the definition of the log normal probability function, including all of the constant normalizing terms. Therefore, the explicit increment form can be used to recreate the exact log probability values for the model. Otherwise, the sampling statement form will be faster if any of the input expressions, y, mu, or sigma, involve only constants, data variables or transformed data variables.

User-Transformed Variables

The left-hand side of a sampling statement may be a complex expression. For instance, it is legal syntactically to write

```
data {
  real<lower=0> y;
}
...
model {
  log(y) ~ normal(mu,sigma);
}
```

Unfortunately, this is not enough to properly model y as having a lognormal distribution. The log Jacobian of the transform must be added to the log probability accumulator to account for the differential change in scale (see Section 53.1 for full definitions). For the case above, the following adjustment will account for the log transform.[2]

```
increment_log_prob(- log(fabs(y)));
```

Truncated Distributions

A density function $p(x)$ may be truncated to an interval (a, b) to define a new density $p_{(a,b)}(x)$ by setting

$$p_{(a,b)}(x) = \frac{p(x)}{\int_a^b p(x')\,dx'}.$$

Given a probability function $p_X(x)$ for a random variable X, its cumulative distribution function (cdf) $F_X(x)$ is defined to be the probability that $X \leq a$. For continuous random variables, this is

$$F_X(x) = \int_{-\infty}^{x} p_X(x')\,dx',$$

whereas for discrete variables, it's

$$F_X(x) = \sum_{n \leq x} p_X(x).$$

The complementary cumulative distribution function (ccdf) is $1 - F_X(x)$.

Cumulative distribution functions are useful for defining truncated distributions, because

$$\int_a^b p(x')\,dx' = F(b) - F(a),$$

[2]Because $\log |\frac{d}{dy} \log y| = \log |1/y| = -\log |y|$; see Section 53.1.

so that

$$p_{(a,b)}(x) = \frac{p_X(x)}{F(b) - F(a)}.$$

On the log scale,

$$\log p_{(a,b)}(x) = \log p_X(x) - \log\left(F(b) - F(a)\right).$$

The denominator is more stably computed using Stan's log_diff_exp operation as

$$\begin{aligned}\log\left(F(b) - F(a)\right) &= \log\left(\exp(\log F(b)) - \exp(\log F(a))\right) \\ &= \text{log\_diff\_exp}\left(\log F(b), \log F(a)\right).\end{aligned}$$

As in BUGS and JAGS, Stan allows probability functions to be truncated. For example, a truncated unit normal distribution restricted to $(-0.5, 2.1)$ is encoded as follows.

```
y ~ normal(0,1) T[-0.5, 2.1];
```

Truncated distributions are translated as an addition summation for the accumulated log probability. For instance, this example has the same translation (up to arithmetic precision issues; see below) as

```
increment_log_prob(normal_log(y,0,1));
increment_log_prob(-(log(normal_cdf(2.1,0,1)
                         - normal_cdf(-0.5,0,1))));
```

The function `normal_cdf` represents the cumulative normal distribution function. For example, `normal_cdf(2.1,0,1)` evaluates to

$$\int_{-\infty}^{2.1} \mathsf{Normal}(x|0,1)\, dx,$$

which is the probability a unit normal variable takes on values less than 2.1, or about 0.98.

Arithmetic precision is handled by working on the log scale and using Stan's log scale cdf implementations. The logarithm of the cdf for the normal distribution is `normal_cdf_log` and the ccdf is `normal_ccdf_log`. Stan's translation for the denominator introduced by truncation is equivalent to

```
increment_log_prob(-log_diff_exp(normal_cdf_log(2.1,0,1),
                                 normal_cdf_log(-0.5,0,1)));
```

As with constrained variable declarations, truncation can be one sided. The density $p(x)$ can be truncated below by a to define a density $p_{(a,)}(x)$ with support (a, ∞) by setting

$$p_{(a,)}(x) = \frac{p(x)}{\int_a^\infty p(x')\,dx'}.$$

For example, the unit normal distribution truncated below at -0.5 would be represented as

```
y ~ normal(0,1) T[-0.5,];
```

The truncation has the same effect as the following direct update to the accumulated log probability (see the subsection of Section 24.3 contrasting log probability increment and sampling statements for more information).

```
increment_log_prob(normal_log(y, 0, 1));
increment_log_prob(-(1 - log(normal_cdf(-0.5, 0, 1))));
```

The denominator is actually implemented with the more efficient and stable version

```
increment_log_prob(-normal_ccdf_log(-0.5, 0, 1));
```

The density $p(x)$ can be truncated above by b to define a density $p_{(,b)}(x)$ with support $(-\infty, b)$ by setting

$$p_{(,b)}(x) = \frac{p(x)}{\int_{-\infty}^b p(x')\,dx'}.$$

For example, the unit normal distribution truncated above at 2.1 would be represented as

```
y ~ normal(0,1) T[,2.1];
```

The truncation has the same effect as the following direct update to the accumulated log probability.

```
increment_log_prob(normal_log(y, 0, 1));
increment_log_prob(-normal_cdf_log(2.1, 0, 1));
```

In all cases, the truncation is only well formed if the appropriate log cumulative distribution functions are defined.[3] Part V and Part VI document the available discrete and continuous cumulative distribution functions. Most distributions have cdf and ccdf functions.

[3] Although most distributions have cdfs and ccdfs implemented, some cumulative distribution functions and their gradients present computational challenges because they lack simple, analytic forms.

For continuous distributions, truncation points must be expressions of type `int` or `real`. For discrete distributions, truncation points must be expressions of type `int`.

For a truncated sampling statement, if the value sampled is not within the bounds specified by the truncation expression, the result is zero probability and the entire statement adds $-\infty$ to the total log probability, which in turn results in the sample being rejected; see the subsection of Section 8.2 discussing constraints and out-of-bounds returns for programming strategies to keep all values within bounds.

Vectorizing Truncated Distributions

Stan does not (yet) support vectorization of distribution functions with truncation.

24.4. For Loops

Suppose `N` is a variable of type `int`, `y` is a one-dimensional array of type `real[]`, and `mu` and `sigma` are variables of type `real`. Furthermore, suppose that `n` has not been defined as a variable. Then the following is a well-formed for-loop statement.

```
for (n in 1:N) {
  y[n] ~ normal(mu,sigma);
}
```

The loop variable is `n`, the loop bounds are the values in the range `1:N`, and the body is the statement following the loop bounds.

Loop Variable Typing and Scope

The bounds in a for loop must be integers. Unlike in R, the loop is always interpreted as an upward counting loop. The range `L:H` will cause the loop to execute the loop with the loop variable taking on all integer values greater than or equal to `L` and less than or equal to `H`. For example, the loop `for (n in 2:5)` will cause the body of the for loop to be executed with `n` equal to 2, 3, 4, and 5, in order. The variable and bound `for (n in 5:2)` will not execute anything because there are no integers greater than or equal to 5 and less than or equal to 2.

Order Sensitivity and Repeated Variables

Unlike in BUGS, Stan allows variables to be reassigned. For example, the variable `theta` in the following program is reassigned in each iteration of the loop.

```
for (n in 1:N) {
  theta <- inv_logit(alpha + x[n] * beta);
  y[n] ~ bernoulli(theta);
}
```

Such reassignment is not permitted in BUGS. In BUGS, for loops are declarative, defining plates in directed graphical model notation, which can be thought of as repeated substructures in the graphical model. Therefore, it is illegal in BUGS or JAGS to have a for loop that repeatedly reassigns a value to a variable.[4]

In Stan, assignments are executed in the order they are encountered. As a consequence, the following Stan program has a very different interpretation than the previous one.

```
for (n in 1:N) {
  y[n] ~ bernoulli(theta);
  theta <- inv_logit(alpha + x[n] * beta);
}
```

In this program, `theta` is assigned after it is used in the probability statement. This presupposes it was defined before the first loop iteration (otherwise behavior is undefined), and then each loop uses the assignment from the previous iteration.

Stan loops may be used to accumulate values. Thus it is possible to sum the values of an array directly using code such as the following.

```
total <- 0.0;
for (n in 1:N)
  total <- total + x[n];
```

After the for loop is executed, the variable `total` will hold the sum of the elements in the array `x`. This example was purely pedagogical; it is easier and more efficient to write

```
total <- sum(x);
```

A variable inside (or outside) a loop may even be reassigned multiple times, as in the following legal code.

```
for (n in 1:100) {
  y <- y + y * epsilon;
  epsilon <- epsilon / 2.0;
  y <- y + y * epsilon;
}
```

[4]A programming idiom in BUGS code simulates a local variable by replacing `theta` in the above example with `theta[n]`, effectively creating `N` different variables, `theta[1]`, ..., `theta[N]`. Of course, this is not a hack if the value of `theta[n]` is required for all `n`.

24.5. Conditional Statements

Stan supports full conditional statements using the same if-then-else syntax as C++. The general format is

```
if (condition1)
  statement1
else if (condition2)
  statement2
...
else if (conditionN-1)
  statementN-1
else
  statementN
```

There must be a single leading `if` clause, which may be followed by any number of `else if` clauses, all of which may be optionally followed by an `else` clause. Each condition must be a real or integer value, with non-zero values interpreted as true and the zero value as false.

The entire sequence of if-then-else clauses forms a single conditional statement for evaluation. The conditions are evaluated in order until one of the conditions evaluates to a non-zero value, at which point its corresponding statement is executed and the conditional statement finishes execution. If none of the conditions evaluates to a non-zero value and there is a final else clause, its statement is executed.

24.6. While Statements

Stan supports standard while loops using the same syntax as C++. The general format is as follows.

```
while (condition)
  body
```

The condition must be an integer or real expression and the body can be any statement (or sequence of statements in curly braces).

Evaluation of a while loop starts by evaluating the condition. If the condition evaluates to a false (zero) value, the execution of the loop terminates and control moves to the position after the loop. If the loop's condition evaluates to a true (non-zero) value, the body statement is executed, then the whole loop is executed again. Thus the loop is continually executed as long as the condition evaluates to a true value.

24.7. Statement Blocks and Local Variable Declarations

Just as parentheses may be used to group expressions, curly brackets may be used to group a sequence of zero or more statements into a statement block. At the beginning of each block, local variables may be declared that are scoped over the rest of the statements in the block.

Blocks in For Loops

Blocks are often used to group a sequence of statements together to be used in the body of a for loop. Because the body of a for loop can be any statement, for loops with bodies consisting of a single statement can be written as follows.

```
for (n in 1:N)
  y[n] ~ normal(mu,sigma);
```

To put multiple statements inside the body of a for loop, a block is used, as in the following example.

```
for (n in 1:N) {
  lambda[n] ~ gamma(alpha,beta);
  y[n] ~ poisson(lambda[n]);
}
```

The open curly bracket ({) is the first character of the block and the close curly bracket (}) is the last character.

Because whitespace is ignored in Stan, the following program will not compile.

```
for (n in 1:N)
  y[n] ~ normal(mu,sigma);
  z[n] ~ normal(mu,sigma); // ERROR!
```

The problem is that the body of the for loop is taken to be the statement directly following it, which is `y[n] ~ normal(mu,sigma)`. This leaves the probability statement for `z[n]` hanging, as is clear from the following equivalent program.

```
for (n in 1:N) {
  y[n] ~ normal(mu,sigma);
}
z[n] ~ normal(mu,sigma); // ERROR!
```

Neither of these programs will compile. If the loop variable `n` was defined before the for loop, the for-loop declaration will raise an error. If the loop variable `n` was not defined before the for loop, then the use of the expression `z[n]` will raise an error.

Local Variable Declarations

A for loop has a statement as a body. It is often convenient in writing programs to be able to define a local variable that will be used temporarily and then forgotten. For instance, the for loop example of repeated assignment should use a local variable for maximum clarity and efficiency, as in the following example.

```
for (n in 1:N) {
  real theta;
  theta <- inv_logit(alpha + x[n] * beta);
  y[n] ~ bernoulli(theta);
}
```

The local variable theta is declared here inside the for loop. The scope of a local variable is just the block in which it is defined. Thus theta is available for use inside the for loop, but not outside of it. As in other situations, Stan does not allow variable hiding. So it is illegal to declare a local variable theta if the variable theta is already defined in the scope of the for loop. For instance, the following is not legal.

```
for (m in 1:M) {
  real theta;
  for (n in 1:N) {
    real theta; // ERROR!
    theta <- inv_logit(alpha + x[m,n] * beta);
    y[m,n] ~ bernoulli(theta);
...
```

The compiler will flag the second declaration of theta with a message that it is already defined.

No Constraints on Local Variables

Local variables may not have constraints on their declaration. The only types that may be used are

int, real, vector[K], row_vector[K], and matrix[M,N].

Blocks within Blocks

A block is itself a statement, so anywhere a sequence of statements is allowed, one or more of the statements may be a block. For instance, in a for loop, it is legal to have the following

```
for (m in 1:M) {
  {
     int n;
     n <- 2 * m;
     sum <- sum + n
  }
  for (n in 1:N)
    sum <- sum + x[m,n];
}
```

The variable declaration `int n;` is the first element of an embedded block and so has scope within that block. The for loop defines its own local block implicitly over the statement following it in which the loop variable is defined. As far as Stan is concerned, these two uses of `n` are unrelated.

24.8. Print Statements

Stan provides print statements that can print literal strings and the values of expressions. Print statements accept any number of arguments. Consider the following for-each statement with a print statement in its body.

```
for (n in 1:N) { print("loop iteration: ", n); ... }
```

The print statement will execute every time the body of the loop does. Each time the loop body is executed, it will print the string “loop iteration: ” (with the trailing space), followed by the value of the expression `n`, followed by a new line.

Print Content

The text printed by a print statement varies based on its content. A literal (i.e., quoted) string in a print statement always prints exactly that string (without the quotes). Expressions in print statements result in the value of the expression being printed. But how the value of the expression is formatted will depend on its type.

Printing a simple `real` or `int` typed variable always prints the variable’s value.[5] For array, vector, and matrix variables, the print format uses brackets. For example, a 3-vector will print as

```
[1,2,3]
```

and a 2×3-matrix as

[5] The adjoint component is always zero during execution for the algorithmic differentiation variables used to implement parameters, transformed parameters, and local variables in the model.

```
[[1,2,3],[4,5,6]]
```

Printing a more readable version of arrays or matrices can be done with loops. An example is the print statement in the following transformed data block.

```
transformed data {
  matrix[2,2] u;
  u[1,1] <- 1.0;  u[1,2] <- 4.0;
  u[2,1] <- 9.0;  u[2,2] <- 16.0;
  for (n in 1:2)
    print("u[", n, "] = ", u[n]);
}
```

This print statement executes twice, printing the following two lines of output.

```
u[1] = [1,4]
u[2] = [9,16]
```

Print Frequency

Printing for a print statement happens every time it is executed. The `transformed data` block is executed once per chain, the `transformed parameter` and `model` blocks once per leapfrog step, and the `generated quantities` block once per iteration.

String Literals

String literals begin and end with a double quote character ("). The characters between the double quote characters may be the space character or any visible ASCII character, with the exception of the backslash character (\) and double quote character ("). The full list of visible ASCII characters is as follows.

```
a b c d e f g h i j k l m n o p q r s t u v w x y z
A B C D E F G H I J K L M N O P Q R S T U V W X Y Z
0 1 2 3 4 5 6 7 8 9 0 ~ @ # $ % ^ & * _ ' ` - + = {
} [ ] ( ) < > | / ! ? . , ; :
```

Debug by `print`

Because Stan is an imperative language, print statements can be very useful for debugging. They can be used to display the values of variables or expressions at various points in the execution of a program. They are particularly useful for spotting problematic not-a-number of infinite values, both of which will be printed.

It is particularly useful to print the value of the log probability accumulator (see Section 31.3), as in the following example.

```
vector[2] y;
y[1] <- 1;
print("lp before =",get_lp());
y ~ normal(0,1); // bug!  y[2] not defined
print("lp after =",get_lp());
```

The example has a bug in that `y[2]` is not defined before the vector `y` is used in the sampling statement. By printing the value of the log probability accumulator before and after each sampling statement, it's possible to isolate where the log probability becomes ill-defined (i.e., becomes not-a-number).

24.9. Reject Statements

The Stan `reject` statement provides a mechanism to report errors or problematic values encountered during program execution and either halt processing or reject samples or optimization iterations.

Like the `print` statement, the reject statement accepts any number of quoted string literals or Stan expressions as arguments.

Reject statements are typically embedded in a conditional statement in order to detect variables in illegal states. For example, the following code handles the case where a variable `x`'s value is negative.

```
if (x < 0)
  reject("x must not be negative; found x=", x);
```

Behavior of Reject Statements

Reject statements have the same behavior as exceptions thrown by built-in Stan functions. For example, the `normal_log` function raises an exception if the input scale is not positive and finite. The effect of a reject statement depends on the program block in which the rejection occurs.

In all cases of rejection, the interface accessing the Stan program should print the arguments to the reject statement.

Rejections in Functions

Rejections in user-defined functions are just passed to the calling function or program block. Reject statements can be used in functions to validate the function arguments,

allowing user-defined functions to fully emulate built-in function behavior. It is better to find out earlier rather than later when there is a problem.

Fatal Exception Contexts

In both the transformed data block and generated quantities block, rejections are fatal. This is because if initialization fails or if generating output fails, there is no way to recover values.

Reject statements placed in the transformed data block can be used to validate both the data and transformed data (if any). This allows more complicated constraints to be enforced that can be specified with Stan's constrained variable declarations.

Recoverable Rejection Contexts

Rejections in the transformed parameters and model blocks are not in and of themselves instantly fatal. The result has the same effect as assigning a $-\infty$ log probability, which causes rejection of the current proposal in MCMC samplers and adjustment of search parameters in optimization.

If the log probability function results in a rejection every time it is called, the containing application (MCMC sampler or optimization) should diagnose this problem and terminate with an appropriate error message. To aid in diagnosing problems, the message for each reject statement will be printed as a result of executing it.

25. User-Defined Functions

Stan allows users to define their own functions. The basic syntax is a simplified version of that used in C and C++. This chapter specifies how functions are declare, defined, and used in Stan; see Chapter 16 for a more programming-oriented perspective.

25.1. Function-Definition Block

User-defined functions appear in a special function-definition block before all of the other program blocks.

```
functions {
   ... function declarations and definitions ...
}
data {
  ...
```

Function definitions and declarations may appear in any order, subject to the condition that a function must be declared before it is used. Forward declarations are allowed in order to support recursive functions; see Section 25.9 for the precise rules governing declarations and definitions.

25.2. Function Names

The rules for function naming and function-argument naming are the same as for other variables; see Section 23.2 for more information on valid identifiers. For example,

```
real foo(real mu, real sigma);
```

declares a function named `foo` with two argument variables of types `real` and `real`. The arguments are named `mu` and `sigma`, but that is not part of the declaration. Two functions may have the same name if they have different sequences of argument types; see Section 25.9 for details. A function may not have the same name and argument type sequence as another function, including a built-in function.

25.3. Calling Functions

All function arguments are mandatory—there are no default values (though see Section 25.9 for alternatives based on overloading).

Functions as Expressions

Functions with non-void return types are called just like any other built-in function in Stan—they are applied to appropriately typed arguments to produce an expression, which has a value when executed.

Functions as Statements

Functions with void return types may be applied to arguments and used as statements. These act like sampling statements or print statements. Such uses are only appropriate for functions that act through side effects, such as incrementing the log probability accumulator, printing, or raising exceptions.

Probability Functions in Sampling Statements

Functions whose name ends in `_log` may be used as probability functions and may be used in place of parameterized distributions on the right-hand-side of sampling statements. There is no restriction on where such functions may be used.

Restritions on Placement

Functions of certain types are restricted on scope of usage. Functions whose names end in `_lp` assume access to the log probability accumulator and are only available in the transformed parameter and model blocks. Functions whose names end in `_rng` assume access to the random number generator and may only be used within the generated quantities block. See Section 25.5 for more information on these two special types of function.

25.4. Unsized Argument Types

Stan's functions all have declared types for both arguments and returned value. As with built-in functions, user-defined functions are only declared for base argument type and dimensionality. This requires a different syntax than for declaring other variables. The choice of language was made so that return types and argument types could use the same declaration syntax.

The type `void` may not be used as an argument type, only a return type for a function with side effects.

Base Variable Type Declaration

The base variable types are `integer`, `real`, `vector`, `row_vector`, and `matrix`. No lower-bound or upper-bound constraints are allowed (e.g., `real<lower=0>` is illegal). Specialized types are also not allowed (e.g., `simplex` is illegal) .

Dimensionality Declaration

Arguments and return types may be arrays, and these are indicated with optional brackets and commas as would be used for indexing. For example, `int` denotes a single integer argument or return, whereas `real[ ]` indicates a one-dimensional array of reals, `real[ , ]` a two-dimensional array and `real[ , , ]` a three-dimensional array; whitespace is optional, as usual.

The dimensions for vectors and matrices are not included, so that `matrix` is the type of a single matrix argument or return type. Thus if a variable is declared as `matrix a`, then `a` has two indexing dimensions, so that `a[1]` is a row vector and `a[1,1]` a real value. Matrices implicitly have two indexing dimensions. The type declaration `matrix[,] b` specifies that `b` is a two-dimensional array of matrices, for a total of four indexing dimensions, with `b[1,1,1,1]` picking out a real value.

Dimensionality Checks and Exceptions

Function argument and return types are not themselves checked for dimensionality. A matrix of any size may be passed in as a matrix argument. Nevertheless, a user-defined function might call a function (such as a multivariate normal density) that itself does dimensionality checks.

Dimensions of function return values will be checked if they're assigned to a previously declared variable. They may also be checked if they are used as the argument to a function.

Any errors raised by calls to functions inside user functions or return type mismatches are simply passed on; this typically results in a warning message and rejection of a proposal during sampling or optimization.

25.5. Function Bodies

The body of a function is bounded by curly braces (`{` and `}`). The body may contain local variable declarations at the top of the function body's block and these scope the same way as local variables used in any other statement block.

The only restrictions on statements in function bodies are external, and determine whether the log probability accumulator or random number generators are available; see the rest of this section for details.

Random Number Generating Functions

Functions that call random number generating functions in their bodies must have a name that ends in `_rng`; attempts to use random-number generators in other functions leads to a compile-time error.

Like other random number generating functions, user-defined functions with names that end in `_rng` may be used only in the generated quantities block. An attempt to use such a function elsewhere results in a compile-time error.

Log Probability Access in Functions

Functions that include sampling statements or log probability increment statements must have a name that ends in `_lp`. Attempts to use sampling statements or increment log probability statements in other functions leads to a compile-time error.

Like the `increment_log_prob` statement and sampling statements, user-defined functions with names that end in `_lp` may only be used in blocks where the log probability accumulator is accessible, namely the transformed parameters and model blocks. An attempt to use such a function elsewhere results in a compile-time error.

Defining Probability Functions for Sampling Statements

Functions whose names end in `_log` can be used as probability functions in sampling statements. As with the built-in functions, the first argument will appear on the left of the sampling statement operator (~) in the sampling statement and the other arguments follow. For example, with a function declared with signature

```
real foo_log(real y, vector theta);
```

allows the use of

```
real z;
vector[K] phi;
...
z ~ foo(phi);
```

to have the exact same effect as

```
increment_log_prob(foo_log(z,phi);
```

so that functions that are going to be accessed as distributions should be defined on the log scale.

Unlike built-in probability functions, user-defined probability functions like the example `foo` above will not automatically drop constant terms.

25.6. Parameters are Constant

Within function definition bodies, the parameters may be used like any other variable. But the parameters are constant in the sense that they can't be assigned to (i.e., can't appear on the left side of an assignment (`<-`) statement. In other words, their value remains constant throughout the function body. Attempting to assign a value to a function parameter value will raise a compile-time error.[1]

Local variables may be declared at the top of the function block and scope as usual.

25.7. Return Value

Non-void functions must have a return statement that returns an appropriately typed expression. If the expression in a return statement does not have the same type as the return type declared for the function, a compile-time error is raised.

Void functions may use `return` only without an argument, but return statements are not mandatory.

Return Guarantee Required

Unlike C++, Stan enforces a syntactic guarantee for non-void functions that ensures control will leave a non-void function through an appropriately typed return statement or because an exception is raised in the execution of the function. To enforce this condition, functions must have a return statement as the last statement in their body. This notion of last is defined recursively in terms of statements that qualify as bodies for functions. The base case is that

- a return statement qualifies,

and the recursive cases are that

- a sequence of statements qualifies if its last statement qualifies,
- a for loop or while loop qualifies if its body qualifies, and
- a conditional statement qualifies if it has a default else clause and all of its body statements qualify.

These rules disqualify

[1]Despite being declared constant and appearing to have a pass-by-value syntax in Stan, the implementation of the language passes function arguments by constant reference in C++.

```
real foo(real x) {
  if (x > 2) return 1.0;
  else if (x <= 2) return -1.0;
}
```

because there is no default `else` clause, and disqualify

```
real foo(real x) {
  real y;
  y <- x;
  while (x < 10) {
    if (x > 0) return x;
    y <- x / 2;
  }
}
```

beause the return statement is not the last statement in the while loop. A bogus dummy return could be placed after the while loop in this case. The rules for returns allow

```
real fancy_log(real x) {
  if (x < 1e-30)
    return x;
  else if (x < 1e-14)
    return x * x;
  else
    return log(x);
}
```

because there's a default else clause and each condition body has return as its final statement.

25.8. Void Functions as Statements

Void Functions

A function can be declared without a return value by using `void` in place of a return type. Note that the type `void` may only be used as a return type—arguments may not be declared to be of type `void`.

Usage as Statement

A void function may be used as a statement after the function is declared; see Section 25.10 for rules on declaration.

Because there is no return, such a usage is only for side effects, such as incrementing the log probability function, printing, or raising an error.

Special Return Statements

In a return statement within a void function's definition, the `return` keyword is followed immediately by a semicolon (`;`) rather than by the expression whose value is returned.

25.9. Overloading

Stan allows function overloading, where two functions have the same name. For example, an overloaded unit-normal log density function might be declared with an overload of the function `unit_normal_log`, giving it three signatures with one argument each.

```
real unit_normal_log(real y);
real unit_normal_log(vector y);
real unit_normal_log(row_vector y);
```

The sequence of argument types must be distinct for each distinct function; attempting to declare two functions with the same name and argument type sequence will cause a compile-time error, even if the return types or argument names are different. For instance, it would not be possible to add the following declaration to those above.

```
vector unit_normal_log(vector y);
```

Overloading for Defaults

In order to write functions with default values, the function must be overloaded; see Section 25.9 for more on overloading. Overloading comes with no guarantees that the overloaded functions all share an underlying implementation. For example, the function `unit_normal_log` above could've just been named `normal_log` and used to overload the regular normal function with default parameter values (0 location and 1 scale).

Functions are not called using named arguments, the options for defaults are more limited than with named arguments in languages such as R. For example, it would not be possible to define two two-argument normal density functions, one of which specified a location and one of which specified a scale,

```
real normal_log(real y, real mu);
real normal_log(real y, real sigma); // illegal
```

because they have the same argument-type sequence, (`real`, `real`).

25.10. Declarations

In general, functions must be declared before they are used. Stan supports forward declarations, which look like function definitions without bodies. For example,

```
real unit_normal_log(real y);
```

declares a function named `unit_normal_log` that consumes a single real-valued input and produces a real-valued output. A function definition with a body simultaneously declares and defines the named function, as in

```
real unit_normal_log(real y) {
  return -0.5 * square(y);
}
```

A user-defined Stan function may be declared and then later defined, or just defined without being declared. No other combination of declaration and definition is legal, so that, for instance, a function may not be declared more than once, nor may it be defined more than once. If there is a declaration, there must be a definition. These rules together ensure that all the declared functions are eventually defined.

Recursive Functions

Forward declarations allow the definition of self-recursive or mutually recursive functions. For instance, consider the following code to compute Fibonacci numbers.

```
int fib(int n);

int fib(int n) {
  if (n < 2) return n;
  else return fib(n-1) + fib(n-2);
}
```

Without the forward declaration in the first line, the body of the definition would not compile.

26. Program Blocks

A Stan program is organized into a sequence of named blocks, the bodies of which consist of variable declarations, followed in the case of some blocks with statements.

26.1. Comments

Stan supports C++-style line-based and bracketed comments. Comments may be used anywhere whitespace is allowed in a Stan program.

Line-Based Comments

In line-based comments, any text on a line following two forward slashes (//) or the pound sign (#) is ignored (along with the slashes or pound sign).

Bracketed Comments

For bracketed comments, any text between a forward-slash and asterisk pair (/*) and an asterisk and forward-slash pair (*/) is ignored.

Character Encoding

Comments may be in ASCII, UTF-8, Latin1, or any other character encoding that is byte-wise compatible with ASCII. This excludes encodings like UTF-16, Big5, etc.[1]

26.2. Overview of Stan's Program Blocks

The full set of named program blocks is exemplified in the following skeletal Stan program.

```
functions {
  ... function declarations and definitions ...
}
data {
  ... declarations ...
}
transformed data {
   ... declarations ... statements ...
}
```

[1] The issue is that they will separate the characters in */ and */.

```
parameters {
   ... declarations ...
}
transformed parameters {
   ... declarations ... statements ...
}
model {
   ... declarations ... statements ...
}
generated quantities {
   ... declarations ... statements ...
}
```

The function-definition block contains user-defined functions. The data block declares the required data for the model. The transformed data block allows the definition of constants and transforms of the data. The parameters block declares the model's parameters — the unconstrained version of the parameters is what's sampled or optimized. The transformed parameters block allows variables to be defined in terms of data and parameters that may be used later and will be saved. The model block is where the log probability function is defined. The generated quantities block allows derived quantities based on parameters, data, and optionally (pseudo) random number generation.

Optionality and Ordering

All of the blocks other than the `model` block are optional. The blocks that occur must occur in the order presented in the skeletal program above. Within each block, both declarations and statements are optional, subject to the restriction that the declarations come before the statements.

Variable Scope

The variables declared in each block have scope over all subsequent statements. Thus a variable declared in the transformed data block may be used in the model block. But a variable declared in the generated quantities block may not be used in any earlier block, including the model block. The exception to this rule is that variables declared in the model block are always local to the model block and may not be accessed in the generated quantities block; to make a variable accessible in the model and generated quantities block, it must be declared as a transformed parameter.

Variables declared as function parameters have scope only within that function definition's body, and may not be assigned to (they are constant).

Function Scope

Functions defined in the function block may be used in any appropriate block. Most functions can be used in any block and applied to a mixture of parameters and data (including constants or program literals).

Random-number-generating functions are restricted to the generated quantities block; such functions are suffixed with `_rng`. Log-probability modifying functions to blocks where the log probability accumulator is in scope (transformed parameters and model); such functions are suffixed with `_lp`.

Density functions defined in the program may be used in sampling statements.

Automatic Variable Definitions

The variables declared in the `data` and `parameters` block are treated differently than other variables in that they are automatically defined by the context in which they are used. This is why there are no statements allowed in the data or parameters block.

The variables in the `data` block are read from an external input source such as a file or a designated R data structure. The variables in the `parameters` block are read from the sampler's current parameter values (either standard HMC or NUTS). The initial values may be provided through an external input source, which is also typically a file or a designated R data structure. In each case, the parameters are instantiated to the values for which the model defines a log probability function.

Transformed Variables

The `transformed data` and `transformed parameters` block behave similarly to each other. Both allow new variables to be declared and then defined through a sequence of statements. Because variables scope over every statement that follows them, transformed data variables may be defined in terms of the data variables.

Before generating any samples, data variables are read in, then the transformed data variables are declared and the associated statements executed to define them. This means the statements in the transformed data block are only ever evaluated once.[2] Transformed parameters work the same way, being defined in terms of the parameters, transformed data, and data variables. The difference is the frequency of evaluation. Parameters are read in and (inverse) transformed to constrained representations on their natural scales once per log probability and gradient evaluation. This means the inverse transforms and their log absolute Jacobian determinants are evaluated once per leapfrog step. Transformed parameters are then declared and their defining statements executed once per leapfrog step.

[2]If the C++ code is configured for concurrent threads, the data and transformed data blocks can be executed once and reused for multiple chains.

Block	*Stmt*	*Action / Period*
`data`	no	read / chain
`transformed data`	yes	evaluate / chain
`parameters`	no	inv. transform, Jacobian / leapfrog<br>inv. transform, write / sample
`transformed parameters`	yes	evaluate / leapfrog<br>write / sample
`model`	yes	evaluate / leapfrog step
`generated quantities`	yes	eval / sample<br>write / sample
(initialization)	n/a	read, transform / chain

Figure 26.1: *The read, write, transform, and evaluate actions and periodicities listed in the last column correspond to the Stan program blocks in the first column. The middle column indicates whether the block allows statements. The last row indicates that parameter initialization requires a read and transform operation applied once per chain.*

Generated Quantities

The generated quantity variables are defined once per sample after all the leapfrog steps have been completed. These may be random quantities, so the block must be rerun even if the Metropolis adjustment of HMC or NUTS rejects the update proposal.

Variable Read, Write, and Definition Summary

A table summarizing the point at which variables are read, written, and defined is given in Figure 26.1. Another way to look at the variables is in terms of their function. To decide which variable to use, consult the charts in Figure 26.2. The last line has no corresponding location, as there is no need to print a variable every iteration that does not depend on parameters.[3] The rest of this chapter provides full details on when and how the variables and statements in each block are executed.

26.3. Statistical Variable Taxonomy

(Gelman and Hill, 2007, p. 366) provides a taxonomy of the kinds of variables used in Bayesian models. Figure 26.3 contains Gelman and Hill's taxonomy along with a

[3]It is possible to print a variable every iteration that does not depend on parameters — just define it (or redefine it if it is transformed data) in the `generated quantities` block.

Params	*Log Prob*	*Print*	*Declare In*
+	+	+	`transformed parameters`
+	+	−	*local in* `model`
+	−	−	*local in* `generated quantities`
+	−	+	`generated quantities`
−	−	+	`generated quantities`*
−	±	−	*local in* `transformed data`
−	+	+	`transformed data` *and* `generated quantities`*

Figure 26.2: *This table indicates where variables that are not basic data or parameters should be declared, based on whether it is defined in terms of parameters, whether it is used in the log probability function defined in the model block, and whether it is printed. The two lines marked with asterisks (∗) should not be used as there is no need to print a variable every iteration that does not depend on the value of any parameters (for information on how to print these if necessary, see Footnote 3 in this chapter).*

Variable Kind	*Declaration Block*
unmodeled data	`data`, `transformed data`
modeled data	`data`, `transformed data`
missing data	`parameters`, `transformed parameters`
modeled parameters	`parameters`, `transformed parameters`
unmodeled parameters	`data`, `transformed data`
generated quantities	`transformed data`, `transformed parameters`, `generated quantities`
loop indices	loop statement

Figure 26.3: *Variables of the kind indicated in the left column must be declared in one of the blocks declared in the right column.*

missing-data kind along with the corresponding locations of declarations and definitions in Stan.

Constants can be built into a model as literals, data variables, or as transformed data variables. If specified as variables, their definition must be included in data files. If they are specified as transformed data variables, they cannot be used to specify the sizes of elements in the `data` block.

The following program illustrates various variables kinds, listing the kind of each variable next to its declaration.

```
data {
```

```
  int<lower=0> N;           // unmodeled data
  real y[N];                // modeled data
  real mu_mu;               // config. unmodeled param
  real<lower=0> sigma_mu;   // config. unmodeled param
}
transformed data {
  real<lower=0> alpha;      // const. unmodeled param
  real<lower=0> beta;       // const. unmodeled param
  alpha <- 0.1;
  beta <- 0.1;
}
parameters {
  real mu_y;                // modeled param
  real<lower=0> tau_y;      // modeled param
}
transformed parameters {
  real<lower=0> sigma_y;    // derived quantity (param)
  sigma_y <- pow(tau_y,-0.5);
}
model {
  tau_y ~ gamma(alpha,beta);
  mu_y ~ normal(mu_mu,sigma_mu);
  for (n in 1:N)
    y[n] ~ normal(mu_y,sigma_y);
}
generated quantities {
  real variance_y;       // derived quantity (transform)
  variance_y <- sigma_y * sigma_y;
}
```

In this example, `y[N]` is a modeled data vector. Although it is specified in the `data` block, and thus must have a known value before the program may be run, it is modeled as if it were generated randomly as described by the model.

The variable `N` is a typical example of unmodeled data. It is used to indicate a size that is not part of the model itself.

The other variables declared in the data and transformed data block are examples of unmodeled parameters, also known as hyperparameters. Unmodeled parameters are parameters to probability densities that are not themselves modeled probabilistically. In Stan, unmodeled parameters that appear in the `data` block may be specified on a per-model execution basis as part of the data read. In the above model, `mu_mu` and `sigma_mu` are configurable unmodeled parameters.

Unmodeled parameters that are hard coded in the model must be declared in the `transformed data` block. For example, the unmodeled parameters `alpha` and `beta`

are both hard coded to the value 0.1. To allow such variables to be configurable based on data supplied to the program at run time, they must be declared in the `data` block, like the variables `mu_mu` and `sigma_mu`.

This program declares two modeled parameters, `mu` and `tau_y`. These are the location and precision used in the normal model of the values in `y`. The heart of the model will be sampling the values of these parameters from their posterior distribution.

The modeled parameter `tau_y` is transformed from a precision to a scale parameter and assigned to the variable `sigma_y` in the `transformed parameters` block. Thus the variable `sigma_y` is considered a derived quantity — its value is entirely determined by the values of other variables.

The `generated quantities` block defines a value `variance_y`, which is defined as a transform of the scale or deviation parameter `sigma_y`. It is defined in the generated quantities block because it is not used in the model. Making it a generated quantity allows it to be monitored for convergence (being a non-linear transform, it will have different autocorrelation and hence convergence properties than the deviation itself).

In later versions of Stan which have random number generators for the distributions, the `generated quantities` block will be usable to generate replicated data for model checking.

Finally, the variable `n` is used as a loop index in the `model` block.

26.4. Program Block: `data`

The rest of this chapter will lay out the details of each block in order, starting with the `data` block in this section.

Variable Reads and Transformations

The `data` block is for the declaration of variables that are read in as data. With the current model executable, each Markov chain of samples will be executed in a different process, and each such process will read the data exactly once.[4]

Data variables are not transformed in any way. The format for data files or data in memory depends on the interface; see the user's guides and interface documentation for PyStan, RStan, and CmdStan for details.

[4]With multiple threads, or even running chains sequentially in a single thread, data could be read only once per set of chains. Stan was designed to be thread safe and future versions will provide a multithreading option for Markov chains.

Statements

The `data` block does not allow statements.

Variable Constraint Checking

Each variable's value is validated against its declaration as it is read. For example, if a variable `sigma` is declared as `real<lower=0>`, then trying to assign it a negative value will raise an error. As a result, data type errors will be caught as early as possible. Similarly, attempts to provide data of the wrong size for a compound data structure will also raise an error.

26.5. Program Block: `transformed data`

The `transformed data` block is for declaring and defining variables that do not need to be changed when running the program.

Variable Reads and Transformations

For the `transformed data` block, variables are all declared in the variable declarations and defined in the statements. There is no reading from external sources and no transformations performed.

Variables declared in the `data` block may be used to declare transformed variables.

Statements

The statements in a `transformed data` block are used to define (provide values for) variables declared in the `transformed data` block. Assignments are only allowed to variables declared in the `transformed data` block.

These statements are executed once, in order, right after the data is read into the data variables. This means they are executed once per chain (though see Footnote 4 in this chapter).

Variables declared in the `data` block may be used in statements in the `transformed data` block.

Restriction on Operations in `transformed data`

The statements in the transformed data block are designed to be executed once and have a deterministic result. Therefore, log probability is not accumulated and sampling statements may not be used. Random number generating functions are also prohibited.

Variable Constraint Checking

Any constraints on variables declared in the `transformed data` block are checked after the statements are executed. If any defined variable violates its constraints, Stan will halt with a diagnostic error message.

26.6. Program Block: `parameters`

The variables declared in the `parameters` program block correspond directly to the variables being sampled by Stan's samplers (HMC and NUTS). From a user's perspective, the parameters in the program block *are* the parameters being sampled by Stan.

Variables declared as parameters cannot be directly assigned values. So there is no block of statements in the `parameters` program block. Variable quantities derived from parameters may be declared in the `transformed parameters` or `generated quantities` blocks, or may be defined as local variables in any statement blocks following their declaration.

There is a substantial amount of computation involved for parameter variables in a Stan program at each leapfrog step within the HMC or NUTS samplers, and a bit more computation along with writes involved for saving the parameter values corresponding to a sample.

Constraining Inverse Transform

Stan's two samplers, standard Hamiltonian Monte Carlo (HMC) and the adaptive No-U-Turn sampler (NUTS), are most easily (and often most effectively) implemented over a multivariate probability density that has support on all of $\mathbb{R}^n$. To do this, the parameters defined in the `parameters` block must be transformed so they are unconstrained.

In practice, the samplers keep an unconstrained parameter vector in memory representing the current state of the sampler. The model defined by the compiled Stan program defines an (unnormalized) log probability function over the unconstrained parameters. In order to do this, the log probability function must apply the inverse transform to the unconstrained parameters to calculate the constrained parameters defined in Stan's `parameters` program block. The log Jacobian of the inverse transform is then added to the accumulated log probability function. This then allows the Stan model to be defined in terms of the constrained parameters.

In some cases, the number of parameters is reduced in the unconstrained space. For instance, a K-simplex only requires $K - 1$ unconstrained parameters, and a K-correlation matrix only requires $\binom{K}{2}$ unconstrained parameters. This means that the probability function defined by the compiled Stan program may have fewer parameters than it would appear from looking at the declarations in the `parameters` program block.

The probability function on the unconstrained parameters is defined in such a way that the order of the parameters in the vector corresponds to the order of the variables defined in the `parameters` program block. The details of the specific transformations are provided in Chapter 53.

Gradient Calculation

Hamiltonian Monte Carlo requires the gradient of the (unnormalized) log probability function with respect to the unconstrained parameters to be evaluated during every leapfrog step. There may be one leapfrog step per sample or hundreds, with more being required for models with complex posterior distribution geometries.

Gradients are calculated behind the scenes using Stan's algorithmic differentiation library. The time to compute the gradient does not depend directly on the number of parameters, only on the number of subexpressions in the calculation of the log probability. This includes the expressions added from the transforms' Jacobians.

The amount of work done by the sampler does depend on the number of unconstrained parameters, but this is usually dwarfed by the gradient calculations.

Writing Samples

In the basic Stan compiled program, the values of variables are written to a file for each sample. The constrained versions of the variables are written, again in the order they are defined in the `parameters` block. In order to do this, the transformed parameter, model, and generated quantities statements must be executed.

26.7. Program Block: `transformed parameters`

The `transformed parameters` program block consists of optional variable declarations followed by statements. After the statements are executed, the constraints on the transformed parameters are validated. Any variable declared as a transformed parameter is part of the output produced for samples.

Any variable that is defined wholly in terms of data or transformed data should be declared and defined in the transformed data block. Defining such quantities in the transformed parameters block is legal, but much less efficient than defining them as transformed data.

26.8. Program Block: `model`

The `model` program block consists of optional variable declarations followed by statements. The variables in the model block are local variables and are not written as part

of the output.

Local variables may not be defined with constraints because there is no well-defined way to have them be both flexible and easy to validate.

The statements in the model block typically define the model. This is the block in which probability (sampling notation) statements are allowed. These are typically used when programming in the BUGS idiom to define the probability model.

26.9. Program Block: `generated quantities`

The `generated quantities` program block is rather different than the other blocks. Nothing in the generated quantities block affects the sampled parameter values. The block is executed only after a sample has been generated.

Among the applications of posterior inference that can be coded in the generated quantities block are

- forward sampling to generate simulated data for model testing,
- generating predictions for new data,
- calculating posterior event probabilities, including multiple comparisons, sign tests, etc.,
- calculating posterior expectations,
- transforming parameters for reporting,
- applying full Bayesian decision theory,
- calculating log likelihoods, deviances, etc. for model comparison.

Forward samples, event probabilities and statistics may all be calculated directly using plug-in estimates. Stan automatically provides full Bayesian inference by producing samples from the posterior distribution of any calculated event probabilities, predictions, or statistics. See Chapter 51 for more information on Bayesian inference.

Within the generated quantities block, the values of all other variables declared in earlier program blocks (other than local variables) are available for use in the generated quantities block.

It is more efficient to define a variable in the generated quantities block instead of the transformed parameters block. Therefore, if a quantity does not play a role in the model, it should be defined in the generated quantities block.

After the generated quantities statements are executed, the constraints on the declared generated quantity variables are validated.

All variables declared as generated quantities are printed as part of the output.

27. Modeling Language Syntax

This chapter defines the basic syntax of the Stan modeling language using a Backus-Naur form (BNF) grammar plus extra-grammatical constraints on function typing and operator precedence and associativity.

27.1. BNF Grammars

Syntactic Conventions

In the following BNF grammars, literal strings are indicated in single quotes ('). Grammar non-terminals are unquoted strings. A prefix question mark (?A) indicates optionality of A. A postfixed Kleene star (A*) indicates zero or more occurrences of A. The notation A % B, following the Boost Spirit parser library's notation, is shorthand for ?(A (B A)*), i.e., any number of A (including zero), separated by B. A postfixed, curly-braced number indicates a fixed number of repetions; e.g., A{6} is equivalent to a sequence of six copies of A.

Programs

```
program ::= ?functions ?data ?tdata ?params ?tparams model ?generated

functions ::= 'functions' function_decls
data ::= 'data' var_decls
tdata ::= 'transformed data' var_decls_statements
params ::= 'parameters' var_decls
tparams ::= 'transformed parameters' var_decls_statements
model ::= 'model' statement
generated ::= 'generated quantities' var_decls_statements

function_decls ::= '{' function_decl* '}'
var_decls ::= '{' var_decl* '}'
var_decls_statements ::= '{' var_decl* statement* '}'
```

Function Declarations

```
funtion_decl ::= unsized_return_type identifier '(' unsized_types ')'
                 statement

unsized_return_type ::= 'void' | unsized_type
unsized_type ::= (basic_type ?unsized_dims)
unsized_types ::= unsized_type % ','
```

```
basic_type ::= 'int' | 'real' | 'vector' | 'row_vector' | 'matrix'
unsized_dims ::= '['  ','*  ']'
```

Variable Declarations

```
var_decl ::= var_type variable ?dims

var_type ::= 'int' range_constraint
           | 'real' range_constraint
           | 'vector' range_constraint '[' expression ']'
           | 'ordered' '[' expression ']'
           | 'positive_ordered' '[' expression ']'
           | 'simplex' '[' expression ']'
           | 'unit_vector' '[' expression ']'
           | 'row_vector' range_constraint '[' expression ']'
           | 'matrix' range_constraint '[' expression ',' expression ']'
           | 'cholesky_factor_corr' '[' expression ']'
           | 'cholesky_factor_cov' '[' expression ?(',' expression) ']'
           | 'corr_matrix' '[' expression ']'
           | 'cov_matrix' '[' expression ']'

range_constraint ::= ?('<' range '>')

range ::= 'lower' '=' expression ',' 'upper' = expression
        | 'lower' '=' expression
        | 'upper' '=' expression

dims ::= '['  expressions ']'

variable ::= identifier

identifier ::= [a-zA-Z] [a-zA-Z0-9_]*
```

Expressions

```
expressions ::= expression % ','
expression ::= numeric_literal
             | variable
             | expression infixOp expression
             | prefixOp expression
             | expression postfixOp
             | expression '[' expressions ']'
             | function_literal '(' ?expressions ')'
             | ode_solve '(' function_literal (',' expression){6} ')'
```

```
            | '(' expression ')'

expressions ::= expression % ','

numeric_literal ::= int_literal | real_literal

integer_literal ::= [0-9]*

real_literal ::= [0-9]* ?('.' [0-9]*) ?exp_literal

exp_literal ::= ('e' | 'E') integer_literal

function_literal ::= identifier
```

Statements

```
statement ::= atomic_statement | nested_statement

atomic_statement ::= atomic_statement_body ';'
atomic_statement_body
::=  lhs '<-' expression
   | expression '~' identifier '(' expressions ')' ?truncation
   | function_literal '(' expressions ')'
   | 'increment_log_prob' '(' expression ')'
   | 'print' '(' (expression | string_literal)* ')'
   | 'reject' '(' (expression | string_literal)* ')'
   | 'return' expression
   | ''

string_literal ::= '"' char* '"'

truncation ::= 'T' '[' ?expression ',' ?expression ']'

lhs ::= identifier ?indices
indices ::= '[' expressions ']'

nested_statement
::=
  | 'if' '(' expression ')' statement
    ('else' 'if' '(' expression ')' statement)*
    ?('else' statement)
  | 'while' '(' expression ')' statement
  | 'for' '(' identifier 'in' expression ':' expression ')' statement
  | '{' var_decl* statement+ '}'
```

27.2. Extra-Grammatical Constraints

Type Constraints

A well-formed Stan program must satisfy the type constraints imposed by functions and distributions. For example, the binomial distribution requires an integer total count parameter and integer variate and when truncated would require integer truncation points. If these constraints are violated, the program will be rejected during parsing with an error message indicating the location of the problem. For information on argument types, see Part IV.

Operator Precedence and Associativity

In the Stan grammar provided in this chapter, the expression `1 + 2 * 3` has two parses. As described in Section 23.4, Stan disambiguates between the meaning $1 + (2 \times 3)$ and the meaning $(1 + 2) \times 3$ based on operator precedences and associativities.

Forms of Numbers

Integer literals longer than one digit may not start with 0 and real literals cannot consist of only a period or only an exponent.

Conditional Arguments

Both the conditional if-then-else statement and while-loop statement require the expression denoting the condition to be a primitive type, integer or real.

ODE Solver Argument Types and Origins

The `integrate_ode` expression requires

- its first argument to refer to a function with signature

  `(real,real[],real[],real[],int[]):real[]`,

- the remaining six arguments must assignable to types

  `real[]`, `real`, `real[]`, `real[]`, `real[]`, and `int[]`

  respectively, and

- the third, fourth, and sixth arguments must be expressions not containing any variables not originating in the data or transformed data blocks.

Part IV

Built-In Functions

28. Vectorization

Stan's scalar log probability functions all support vectorized function application, with results defined to be the sum of the elementwise application of the function. In all cases, matrix operations are faster than loops and vectorized log probability functions are faster than their equivalent form defined with loops.

Stan also overloads the multivariate normal distribution, allowing arrays of row vectors or vectors for the variate and location parameter.

Stan also overloads some scalar functions, such as `log` and `exp`, to apply to vectors (arrays) and return vectors (arrays). These vectorizations are defined elementwise and unlike the probability functions, provide only minimal efficiency speedups over repeated application and assignment in a loop.

28.1. Vectorized Function Signatures

Vectorized Scalar Arguments

The normal probability function is specified with the signature

```
normal_log(reals,reals,reals);
```

The pseudo-type `reals` is used to indicate that an argument position may be vectorized. Argument positions declared as `reals` may be filled with a real, a one-dimensional array, a vector, or a row-vector. If there is more than one array or vector argument, their types can be anything but their size must match. For instance, it is legal to use `normal_log(row_vector,vector,real)` as long as the vector and row vector have the same size.

Vectorized Vector and Row Vector Arguments

The multivariate normal distribution accepting vector or array of vector arguments is written as

```
multi_normal_log(vectors,vectors,matrix);
```

Vectorized Integer Arguments

The pseudo-type `ints` is used for vectorized integer arguments.

28.2. Evaluating Vectorized Functions

The result of a vectorized log probability function is equivalent to the sum of the evaluations on each element. Any non-vector argument, namely `real` or `int`, is repeated. For instance, if `y` is a vector of size `N`, `mu` is a vector of size `N`, and `sigma` is a scalar, then

```
ll <- normal_log(y, mu, sigma);
```

is just a more efficient way to write

```
ll <- 0;
for (n in 1:N)
  ll <- ll + normal_log(y[n], mu[n], sigma);
```

With the same arguments, the vectorized sampling statement

```
y ~ normal(mu, sigma);
```

has the same effect on the total log probability as

```
for (n in 1:N)
  y[n] ~ normal(mu[n], sigma);
```

29. Void Functions

Stan does not technically support functions that do not return values. It does support two types of statements that look like functions, one for incrementing log probabilities and one for printing. Documentation on these functions is included here for completeness.

Although it's not part of Stan's type language, in this chapter, `void` will be used for the return type.

29.1. Increment Log Probability

There is a special function `increment_log_prob` takes a single argument of any expression type `T`.

`void` **`increment_log_prob`**`(T` *`lp`*`)`

> Add *`lp`* (or elements of *`lp`* if `T` is a container type) to the total log probability accumulator returned by the log probability function defined by a Stan model.

The expression `lp` can be of any expression type. Specifically, it can be an integer or real primitive, a vector, row vector, or matrix, or an array of any type, including multidimensional arrays and arrays of matrices, vectors, or row vectors. Vector arguments are included for convenience and have no speed advantage over adding values in a loop.

The full behavior of the `increment_log_prob` statement and its relation to sampling statements is described in Section 24.2.

29.2. Print

The `print` statement is unique among Stan's syntactic constructs in two ways. First, it is the only function-like construct that allows a variable number of arguments. Second, it is the only function-like construct to accept string literals (e.g., `"hello world"`) as arguments.

Printing has no effect on the model's log probability function. Its sole purpose is the side effect (i.e., an effect not represented in a return value) of arguments being printed to whatever the standard output stream is connected to (e.g., the terminal in command-line Stan or the R console in RStan).

`void` **`print`**`(T1` *`x1`*`,..., TN` *`xN`*`)`

> Print the values denoted by the arguments *`x1`* through *`xN`* on the standard output stream. There are no spaces between items in the print, but a line feed (LF; Unicode U+000A; C++ literal `'\n'`) is inserted at the end of the printed line.

The types `T1` through `TN` can be any of Stan's built-in numerical types or double quoted strings of ASCII characters.

The full behavior of the `print` statement with examples is documented in Section 24.8.

30. Integer-Valued Basic Functions

This chapter describes Stan's built-in function that take various types of arguments and return results of type integer.

30.1. Integer-Valued Arithmetic Operators

Stan's arithmetic is based on standard double-precision C++ integer and floating-point arithmetic. If the arguments to an arithmetic operator are both integers, as in `2 + 2`, integer arithmetic is used. If one argument is an integer and the other a floating-point value, as in `2.0 + 2` and `2 + 2.0`, then the integer is promoted to a floating point value and floating-point arithmetic is used.

Integer arithmetic behaves slightly differently than floating point arithmetic. The first difference is how overflow is treated. If the sum or product of two integers overflows the maximum integer representable, the result is an undesirable wraparound behavior at the bit level. If the integers were first promoted to real numbers, they would not overflow a floating-point representation. There are no extra checks in Stan to flag overflows, so it is up to the user to make sure it does not occur.

Secondly, because the set of integers is not closed under division and there is no special infinite value for integers, integer division implicitly rounds the result. If both arguments are positive, the result is rounded down. For example, `1 / 2` evaluates to 0 and `5 / 3` evaluates to 1.

If one of the integer arguments to division is negative, the latest C++ specification (C++11), requires rounding toward zero. This would have `-1 / 2` evaluate to 0 and `-7 / 2` evaluate to 3. Before the C++11 specification, the behavior was platform dependent, allowing rounding up or down. All compilers recent enough to be able to deal with Stan's templating should follow the C++11 specification, but it may be worth testing if you are not sure and plan to use integer division with negative values.

Unlike floating point division, where `1.0 / 0.0` produces the special positive infinite value, integer division by zero, as in `1 / 0`, has undefined behavior in the C++ standard. For example, the `clang`++ compiler on Mac OS X returns 3764, whereas the `g`++ compiler throws an exception and aborts the program with a warning. As with overflow, it is up to the user to make sure integer divide-by-zero does not occur.

Binary Infix Operators

Operators are described using the C++ syntax. For instance, the binary operator of addition, written `X + Y`, would have the Stan signature `int operator+(int,int)` indicating it takes two real arguments and returns a real value.

`int` **`operator+`**`(int x, int y)`

The sum of the addends *x* and *y*

`int` **`operator-`**`(int x, int y)`

The difference between the minuend *x* and subtrahend *y*

`int` **`operator*`**`(int x, int y)`

The product of the factors *x* and *y*

`int` **`operator/`**`(int x, int y)`

The integer quotient of the dividend *x* and divisor *y*

Unary Prefix Operators

`int` **`operator-`**`(int x)`

The negation of the subtrahend *x*

`int` **`operator+`**`(int x)`

This is a no-op.

30.2. Absolute Functions

`int` **`abs`**`(int x)`

Returns the absolute value of *x*.

$$\text{abs}(x) = |x|$$

`int` **`int_step`**`(int x)`

`int` **`int_step`**`(real x)`

Returns the integer step, or Heaviside, function of *x*.

$$\text{int\_step}(x) = \begin{cases} 0 & \text{if } x \leq 0 \\ 1 & \text{if } x > 0 \end{cases}$$

See the warning in Section 31.6 about the dangers of step functions applied to anything other than data.

30.3. Bound Functions

`int **min**(int *x*, int *y*)`

Returns the minimum of x and y.

$$\min(x, y) = \begin{cases} x & \text{if } x < y \\ y & \text{otherwise} \end{cases}$$

`int **max**(int *x*, int *y*)`

Returns the maximum of x and y.

$$\max(x, y) = \begin{cases} x & \text{if } x > y \\ y & \text{otherwise} \end{cases}$$

31. Real-Valued Basic Functions

This chapter describes built-in functions that take zero or more real or integer arguments and return real values.

31.1. Mathematical Constants

Constants are represented as functions with no arguments and must be called as such. For instance, the mathematical constant π must be written in a Stan program as `pi()`.

`real` **`pi`**`()`

π, the ratio of a circle's circumference to its diameter

`real` **`e`**`()`

e, the base of the natural logarithm

`real` **`sqrt2`**`()`

The square root of 2

`real` **`log2`**`()`

The natural logarithm of 2

`real` **`log10`**`()`

The natural logarithm of 10

31.2. Special Values

`real` **`not_a_number`**`()`

Not-a-number, a special non-finite real value returned to signal an error

`real` **`positive_infinity`**`()`

Positive infinity, a special non-finite real value larger than all finite numbers

`real` **`negative_infinity`**`()`

Negative infinity, a special non-finite real value smaller than all finite numbers

`real` **`machine_precision`**`()`

The smallest number x such that $(x + 1) \neq 1$ in floating-point arithmetic on the current hardware platform

31.3. Log Probability Function

The basic purpose of a Stan program is to compute a log probability function and its derivatives. The log probability function in a Stan model outputs the log density on the unconstrained scale. A log probabilty accumulator starts at zero and is then incremented in various ways by a Stan program. The variables are first transformed from unconstrained to constrained, and the log Jacobian determinant added to the log probability accumulator. Then the model block is executed on the constrained parmeters, with each sampling statement (~) and log probability increment statement (`increment_log_prob`) adding to the accumulator. At the end of the model block execution, the value of the log probability accumulator is the log probability value returned by the Stan program.

Stan provides a special built-in function `get_lp` that takes no arguments and returns the current value of the log probability accumulator. This function is primarily useful for debugging purposes, where for instance, it may be used with a print statement to display the log probability accumulator at various stages of execution to see where it becomes ill defined.

`real` **`get_lp`**`()`

Returns the current value of the log probability accumulator.

Like user-defined functions that end in `_lp`, the log probability get function may only be used in the transformed parameter and model blocks, as well as in the bodies of functions with names that also end in `_lp`.

31.4. Logical Functions

Like C++, BUGS, and R, Stan uses 0 to encode false, and 1 to encode true. Stan supports the usual boolean comparison operations and boolean operators. These all have the same syntax and precedence as in C++; for the full list of operators and precedences, see Figure 23.1.

Comparison Operators

All comparison operators return boolean values, either 0 or 1. Each operator has two signatures, one for integer comparisons and one for floating-point comparisons. Comparing an integer and real value is carried out by first promoting the integer value.

`int` **`operator<`**`(int` *`x`*`, int` *`y`*`)`

int **operator<**(real *x*, real *y*)

Returns 1 if *x* is less than *y* and 0 otherwise.

$$(x{<}y) = \text{operator<}(x, y) = \begin{cases} 1 & \text{if } x < y \\ 0 & \text{otherwise} \end{cases}$$

int **operator<=**(int *x*, int *y*)

int **operator<=**(real *x*, real *y*)

Returns 1 if *x* is less than or equal *y* and 0 otherwise.

$$(x{<=}y) = \text{operator<=}(x, y) = \begin{cases} 1 & \text{if } x \leq y \\ 0 & \text{otherwise} \end{cases}$$

int **operator>**(int *x*, int *y*)

int **operator>**(real *x*, real *y*)

Returns 1 if *x* is greater than *y* and 0 otherwise.

$$(x{>}y) = \text{operator>}(x, y) = \begin{cases} 1 & \text{if } x > y \\ 0 & \text{otherwise} \end{cases}$$

int **operator>=**(int *x*, int *y*)

int **operator>=**(real *x*, real *y*)

Returns 1 if *x* is greater than or equal to *y* and 0 otherwise.

$$(x{>=}y) = \text{operator>=}(x, y) = \begin{cases} 1 & \text{if } x \geq y \\ 0 & \text{otherwise} \end{cases}$$

int **operator==**(int *x*, int *y*)

int **operator==**(real *x*, real *y*)

Returns 1 if *x* is equal to *y* and 0 otherwise.

$$(x{==}y) = \text{operator==}(x, y) = \begin{cases} 1 & \text{if } x = y \\ 0 & \text{otherwise} \end{cases}$$

int **operator!=**(int *x*, int *y*)

`int` **`operator!=`**`(real` *x*`, real` *y*`)`

Returns 1 if *x* is not equal to *y* and 0 otherwise.

$$(x!\!=\!y) = \text{operator!=}(x, y) = \begin{cases} 1 & \text{if } x \neq y \\ 0 & \text{otherwise} \end{cases}$$

Boolean Operators

Boolean operators return either 0 for false or 1 for true. Inputs may be any real or integer values, with non-zero values being treated as true and zero values treated as false. These operators have the usual precedences, with negation (not) binding the most tightly, conjunction the next and disjunction the weakest; all of the operators bind more tightly than the comparisons. Thus an expression such as `!a && b` is interpreted as `(!a) && b`, and `a < b || c >= d && e != f` as `(a < b) || (((c >= d) && (e != f)))`.

`int` **`operator!`**`(int` *x*`)`

`int` **`operator!`**`(real` *x*`)`

Returns 1 if *x* is zero and 0 otherwise.

$$(!x) = \text{operator!}(x) = \begin{cases} 0 & \text{if } x \neq 0 \\ 1 & \text{if } x = 0 \end{cases}$$

`int` **`operator&&`**`(int` *x*`, int` *y*`)`

`int` **`operator&&`**`(real` *x*`, real` *y*`)`

Returns 1 if *x* is unequal to 0 and *y* is unequal to 0.

$$(x\&\&y) = \text{operator\&\&}(x, y) = \begin{cases} 1 & \text{if } x \neq 0 \text{ and } y \neq 0 \\ 0 & \text{otherwise} \end{cases}$$

`int` **`operator||`**`(int` *x*`, int` *y*`)`

`int` **`operator||`**`(real` *x*`, real` *y*`)`

Returns 1 if *x* is unequal to 0 or *y* is unequal to 0.

$$(x||y) = \text{operator||}(x, y) = \begin{cases} 1 & \text{if } x \neq 0 \text{ or } y \neq 0 \\ 0 & \text{otherwise} \end{cases}$$

Boolean Operator Short Circuiting

Like in C++, the boolean operators `&&` and `||` and are implemented to short circuit directly to a return value after evaluating the first argument if it is sufficient to resolve the result. In evaluating `a || b`, if `a` evaluates to a value other than zero, the expression returns the value 1 without evaluating the expression `b`. Similarly, evaluating `a && b` first evaluates `a`, and if the result is zero, returns 0 without evaluating `b`.

Logical Functions

The logical functions introduce conditional behavior functionally and are primarily provided for compatibility with BUGS and JAGS.

`real` **`if_else`**`(int` *`cond`*`, real` *`x`*`, real` *`y`*`)`

Returns x if *cond* is non-zero and y otherwise. Stan's `if_else` function does not short circuit—it always evaluates both arguments x and y.

$$\text{if\_else}(cond, x, y) = \begin{cases} x & \text{if } cond \neq 0 \\ y & \text{otherwise} \end{cases}$$

`real` **`step`**`(real` *`x`*`)`

Returns 1 if x is positive and 0 otherwise.

$$\text{step}(x) = \begin{cases} 1 & \text{if } x > 0 \\ 0 & \text{otherwise} \end{cases}$$

Both of these are step-like functions; see the warning in Section 31.6 applied to expressions dependent on parameters.

The step function is often used in BUGS to perform conditional operations. For instance, `step(a - b)` evaluates to 1 if `a` is greater than `b` and evaluates to 0 otherwise. In Stan, the expression `step(a - b)` is more naturally coded directly in terms of the comparison operator, `(a > b)`.

The log probability function and gradient evaluations are more efficient in Stan when implemented using conditional statements than with mulitplications by step functions. For example, if `y` is a `real` variable, and `c`, `x1`, and `x2` are scalar expressions (type `real` or `int`), then the assignment statements

```
y <- x1 * step(c) + x2 * (1 - step(c));
```

and

```
y <- if_else(c > 0, x1, x2);
```

are more efficiently written with the conditional statement

```
if (c > 0)
  y <- x1;
else
  y <- x2;
```

The reason the functional versions are slower is that they evaluate all of their arguments; the step function approach is particularly slow as it also introduces arithmetic operations. The overhead will be more noticeable if `c`, `x1` or `x2` are parameters (including transformed parameters and local variables that depend on parameters) or if `x1` and `x2` are complicated expressions rather than constants or simple variables.

`int` **`is_inf`**`(real` *x*`)`

Returns *1* if x is infinite (positive or negative) and *0* otherwise.

`int` **`is_nan`**`(real` *x*`)`

Returns *1* if x is NaN and *0* otherwise.

Care must be taken because both of these indicator functions are step-like and thus can cause discontinuities in gradients when applied to parameters; see Section 31.6 for details.

31.5. Real-Valued Arithmetic Operators

The arithmetic operators are presented using C++ notation. For instance `operator+(x,y)` refers to the binary addition operator and `operator-(x)` to the unary negation operator. In Stan programs, these are written using the usual infix and prefix notations as `x + y` and `-x`, respectively.

Binary Infix Operators

`real` **`operator+`**`(real` *x*`, real` *y*`)`

Returns the sum of x and y.

$$(x + y) = \text{operator+}(x, y) = x + y$$

`real` **`operator-`**`(real x, real y)`

Returns the difference between *x* and *y*.

$$(x - y) = \text{operator-}(x, y) = x - y$$

`real` **`operator*`**`(real x, real y)`

Returns the product of *x* and *y*.

$$(x * y) = \text{operator*}(x, y) = xy$$

`real` **`operator/`**`(real x, real y)`

Returns the quotient of *x* and *y*.

$$(x/y) = \text{operator/}(x, y) = \frac{x}{y}$$

`real` **`operator^`**`(real x, real y)`

Return *x* raised to the power of *y*.

$$(x\hat{}y) = \text{operator}\hat{}(x, y) = x^y$$

Unary Prefix Operators

`real` **`operator-`**`(real x)`

Returns the negation of the subtrahend *x*.

$$(-x) = \text{operator-}(x) = -x$$

`real` **`operator+`**`(real x)`

Returns the value of *x*.

$$(+x) = \text{operator+}(x) = x$$

31.6. Step-like Functions

Warning: *These functions can seriously hinder sampling and optimization efficiency for gradient-based methods (e.g., NUTS, HMC, BFGS) if applied to parameters (including transformed parameters and local variables in the transformed parameters or model block). The problem is that they break gradients due to discontinuities coupled with zero gradients elsewhere. They do not hinder sampling when used in the data, transformed data, or generated quantities blocks.*

Absolute Value Functions

`real` **`abs`**`(real x)`

Returns the absolute value of *x*; this function is deprecated and will be removed in the future; please use `fabs` instead.

$$\mathrm{abs}(x) = |x|$$

`real` **`fabs`**`(real x)`

Returns the absolute value of *x*; see warning at start of Section 31.6.

$$\mathrm{fabs}(x) = |x|$$

`real` **`fdim`**`(real x, real y)`

Returns the positive difference between *x* and *y*, which is *x* - *y* if *x* is greater than *y* and 0 otherwise; see warning at start of Section 31.6.

$$\mathrm{fdim}(x, y) = \begin{cases} x - y & \text{if } x \geq y \\ 0 & \text{otherwise} \end{cases}$$

Bounds Functions

`real` **`fmin`**`(real x, real y)`

Returns the minimum of *x* and *y*; see warning at start of Section 31.6.

$$\mathrm{fmin}(x, y) = \begin{cases} x & \text{if } x \leq y \\ y & \text{otherwise} \end{cases}$$

`real` **`fmax`**`(real x, real y)`

Returns the maximum of x and y; see warning at start of Section 31.6.

$$\text{fmax}(x, y) = \begin{cases} x & \text{if } x \geq y \\ y & \text{otherwise} \end{cases}$$

Arithmetic Functions

`real` **`fmod`**`(real x, real y)`

Returns the real value remainder after dividing x by y; see warning at start of Section 31.6.

$$\text{fmod}(x, y) = x - \left\lfloor \frac{x}{y} \right\rfloor y$$

The operator $\lfloor u \rfloor$ is the floor operation; see below.

Rounding Functions

Warning: Rounding functions convert real values to integers. Because the output is an integer, any gradient information resulting from functions applied to the integer is not passed to the real value it was derived from. With MCMC sampling using HMC or NUTS, the Metropolis/slice procedure will correct for any error due to poor gradient calculations, but the result is likely to be reduced acceptance probabilities and less efficient sampling.

The rounding functions cannot be used as indices to arrays because they return real values. Stan may introduce integer-valued versions of these in the future, but as of now, there is no good workaround.

`real` **`floor`**`(real x)`

Returns the floor of x, which is the largest integer less than or equal to x, converted to a real value; see warning at start of Section 31.6.

$$\text{floor}(x) = \lfloor x \rfloor$$

`real` **`ceil`**`(real x)`

Returns the ceiling of x, which is the smallest integer greater than or equal to x, converted to a real value; see warning at start of Section 31.6.

$$\text{ceil}(x) = \lceil x \rceil$$

`real` **`round`**`(real x)`

Returns the nearest integer to x, converted to a real value; see warning at start of Section 31.6.

$$\text{round}(x) = \begin{cases} \lceil x \rceil & \text{if } x - \lfloor x \rfloor \geq 0.5 \\ \lfloor x \rfloor & \text{otherwise} \end{cases}$$

`real` **`trunc`**`(real x)`

Returns the integer nearest to but no larger in magnitude than x, converted to a double value; see warning at start of Section 31.6.

$$\text{trunc}(x) = \lfloor x \rfloor$$

Note that this function is redundant with `floor`.

31.7. Power and Logarithm Functions

`real` **`sqrt`**`(real x)`

Returns the square root of x.

$$\text{sqrt}(x) = \begin{cases} \sqrt{x} & \text{if } x \geq 0 \\ \text{NaN} & \text{otherwise} \end{cases}$$

`real` **`cbrt`**`(real x)`

Returns the cube root of x

$$\text{cbrt}(x) = \sqrt[3]{x}$$

`real` **`square`**`(real x)`

Returns the square of x.

$$\text{square}(x) = x^2$$

`real` **`exp`**`(real x)`

Returns the natural exponential of x.

$$\exp(x) = e^x$$

`real exp2(real x)`

Returns the base-2 exponential of *x*.

$$\text{exp2}(x) = 2^x$$

`real log(real x)`

Returns the natural logarithm of *x*.

$$\log(x) = \begin{cases} \log_e(x) & \text{if } x \geq 0 \\ \text{NaN} & \text{otherwise} \end{cases}$$

`real log2(real x)`

Returns the base-2 logarithm of *x*.

$$\text{log2}(x) = \begin{cases} \log_2(x) & \text{if } x \geq 0 \\ \text{NaN} & \text{otherwise} \end{cases}$$

`real log10(real x)`

Returns the base-10 logarithm of *x*.

$$\text{log10}(x) = \begin{cases} \log_{10}(x) & \text{if } x \geq 0 \\ \text{NaN} & \text{otherwise} \end{cases}$$

`real pow(real x, real y)`

Returns *x* raised to the power of *y*.

$$\text{pow}(x, y) = x^y$$

`real inv(real x)`

Returns the inverse of *x*.

$$\text{inv}(x) = \frac{1}{x}$$

`real` **`inv_sqrt`**`(real` *`x`*`)`

Returns the inverse of the square root of *x*.

$$\text{inv\_sqrt}(x) = \begin{cases} \frac{1}{\sqrt{x}} & \text{if } x \geq 0 \\ \text{NaN} & \text{otherwise} \end{cases}$$

`real` **`inv_square`**`(real` *`x`*`)`

Returns the inverse of the square of *x*.

$$\text{inv\_square}(x) = \frac{1}{x^2}$$

31.8. Trigonometric Functions

`real` **`hypot`**`(real` *`x`*`, real` *`y`*`)`

Returns the length of the hypotenuse of a right triangle with sides of length *x* and *y*.

$$\text{hypot}(x, y) = \begin{cases} \sqrt{x^2 + y^2} & \text{if } x, y \geq 0 \\ \text{NaN} & \text{otherwise} \end{cases}$$

`real` **`cos`**`(real` *`x`*`)`

Returns the cosine of the angle *x* (in radians).

$$\cos(x) = \cos(x)$$

`real` **`sin`**`(real` *`x`*`)`

Returns the sine of the angle *x* (in radians).

$$\sin(x) = \sin(x)$$

`real` **`tan`**`(real` *`x`*`)`

Returns the tangent of the angle *x* (in radians).

$$\tan(x) = \tan(x)$$

`real `**`acos`**`(real x)`

Returns the principal arc (inverse) cosine (in radians) of *x*.

$$\text{acos}(x) = \begin{cases} \arccos(x) & \text{if } -1 \leq x \leq 1 \\ \text{NaN} & \text{otherwise} \end{cases}$$

`real `**`asin`**`(real x)`

Returns the principal arc (inverse) sine (in radians) of *x*.

$$\text{asin}(x) = \begin{cases} \arcsin(x) & \text{if } -1 \leq x \leq 1 \\ \text{NaN} & \text{otherwise} \end{cases}$$

`real `**`atan`**`(real x)`

Returns the principal arc (inverse) tangent (in radians) of *x*.

$$\text{atan}(x) = \arctan(x)$$

`real `**`atan2`**`(real x, real y)`

Returns the principal arc (inverse) tangent (in radians) of *x* divided by *y*.

$$\text{atan2}(x, y) = \arctan\left(\frac{x}{y}\right)$$

31.9. Hyperbolic Trigonometric Functions

`real `**`cosh`**`(real x)`

Returns the hyperbolic cosine of *x* (in radians).

$$\cosh(x) = \cosh(x)$$

`real `**`sinh`**`(real x)`

Returns the hyperbolic sine of *x* (in radians).

$$\sinh(x) = \sinh(x)$$

`real` **`tanh`**`(real x)`

Returns the hyperbolic tangent of `x` (in radians).

$$\text{tanh}(x) = \tanh(x)$$

`real` **`acosh`**`(real x)`

Returns the inverse hyperbolic cosine (in radians) of `x`.

$$\text{acosh}(x) = \begin{cases} \cosh^{-1}(x) & \text{if } x \geq 1 \\ \text{NaN} & \text{otherwise} \end{cases}$$

`real` **`asinh`**`(real x)`

Returns the inverse hyperbolic cosine (in radians) of `x`.

$$\text{asinh}(x) = \sinh^{-1}(x)$$

`real` **`atanh`**`(real x)`

Returns the inverse hyperbolic tangent (in radians) of `x`.

$$\text{atanh}(x) = \begin{cases} \tanh^{-1}(x) & \text{if } -1 \leq x \leq 1 \\ \text{NaN} & \text{otherwise} \end{cases}$$

31.10. Link Functions

The following functions are commonly used as link functions in generalized linear models (see Section 5.4). The function Φ is also commonly used as a link function (see Section 31.11).

`real` **`logit`**`(real x)`

Returns the log odds, or logit, function applied to `x`.

$$\text{logit}(x) = \begin{cases} \log \frac{x}{1-x} & \text{if } 0 \leq x \leq 1 \\ \text{NaN} & \text{otherwise} \end{cases}$$

`real` **`inv_logit`**`(real y)`

Returns the logistic sigmoid function applied to *y*.

$$\text{inv\_logit}(y) = \frac{1}{1 + \exp(-y)}$$

`real` **`inv_cloglog`**`(real y)`

Returns the inverse of the complementary log-log function applied to *y*.

$$\text{inv\_cloglog}(y) = 1 - \exp(-\exp(y))$$

31.11. Probability-Related Functions

Normal Cumulative Distribution Functions

The error function erf is related to the unit normal cumulative distribution function Φ by scaling. See Section 39.1 for the general normal cumulative distribution function (and its complement).

`real` **`erf`**`(real x)`

Returns the error function, also known as the Gauss error function, of *x*.

$$\text{erf}(x) = \frac{2}{\sqrt{\pi}} \int_0^x e^{-t^2} dt$$

`real` **`erfc`**`(real x)`

Returns the complementary error function of *x*.

$$\text{erfc}(x) = \frac{2}{\sqrt{\pi}} \int_x^\infty e^{-t^2} dt$$

`real` **`Phi`**`(real x)`

Returns the unit normal cumulative distribution function of *x*.

$$\text{Phi}(x) = \frac{1}{\sqrt{2\pi}} \int_0^x e^{-t^2/2} dt$$

`real` **`Phi_approx`**`(real x)`

Returns a fast approximation of the unit normal cumulative distribution function of `x`. This approximation has a maximum absolute error of 0.00014 and may be used instead of `Phi` for probit regression. See (Bowling et al., 2009) for details.

$$\text{Phi\_approx}(x) = \text{logit}^{-1}(0.07056\,x^3 + 1.5976\,x)$$

Other Probability-Related Functions

`real` **`binary_log_loss`**`(int y, real y_hat)`

Returns the log loss function for for predicting $\hat{y} \in [0,1]$ for boolean outcome $y \in \{0,1\}$.

$$\text{binary\_log\_loss}(y,\hat{y}) = \begin{cases} -\log \hat{y} & \text{if } y = 0 \\ -\log(1-\hat{y}) & \text{otherwise} \end{cases}$$

`real` **`owens_t`**`(real h, real a)`

Returns the Owen's T function for the probability of the event $X > h$ and $0 < Y < aX$ where X and Y are independent standard normal random variables.

$$\text{owens\_t}(h,a) = \frac{1}{2\pi} \int_0^a \frac{\exp(-\frac{1}{2}h^2(1+x^2))}{1+x^2} dx$$

31.12. Combinatorial Functions

`real` **`lbeta`**`(real alpha, real beta)`

Returns the natural logarithm of the beta function applied to `alpha` and `beta`. The beta function, $\mathrm{B}(\alpha,\beta)$, computes the normalizing constant for the beta distribution, and is defined for $\alpha > 0$ and $\beta > 0$.

$$\text{lbeta}(\alpha,\beta) = \log\Gamma(a) + \log\Gamma(b) - \log\Gamma(a+b)$$

See Section E.1 for definition of $\mathrm{B}(\alpha,\beta)$.

`real` **`tgamma`**`(real x)`

Returns the gamma function applied to `x`. The gamma function is the generalization of the factorial function to continuous variables, defined so that

$\Gamma(n+1) = n!$. The function is defined for positive numbers and non-integral negative numbers.

$$\text{tgamma}(x) = \begin{cases} \Gamma(x) & \text{if } x \notin \{\dots,-3,-2,-1,0\} \\ \text{error} & \text{otherwise} \end{cases}$$

See Section E.2 for definition of $\Gamma(x)$.

`real` **`lgamma`**`(real` *`x`*`)`

Returns the natural logarithm of the gamma function applied to *x*.

$$\text{lgamma}(x) = \begin{cases} \log\Gamma(x) & \text{if } x \notin \{\dots,-3,-2,-1,0\} \\ \text{error} & \text{otherwise} \end{cases}$$

See Section E.2 for definition of $\Gamma(x)$.

`real` **`digamma`**`(real` *`x`*`)`

Returns the digamma function applied to *x*. The digamma function is the derivative of the natural logarithm of the Gamma function. The function is defined for positive numbers and non-integral negative numbers.

$$\text{digamma}(x) = \begin{cases} \frac{\Gamma'(x)}{\Gamma(x)} & \text{if } x \notin \{\dots,-3,-2,-1,0\} \\ \text{error} & \text{otherwise} \end{cases}$$

See Section E.2 for definition of $\Gamma(x)$.

`real` **`trigamma`**`(real` *`x`*`)`

Returns the trigamma function applied to *x*. The trigamma function is the second derivative of the natural logarithm of the Gamma function.

$$\text{trigamma}(x) = \begin{cases} \sum_{n=0}^{\infty} \frac{1}{(x+n)^2} & \text{if } x \notin \{\dots,-3,-2,-1,0\} \\ \text{error} & \text{otherwise} \end{cases}$$

`real` **`lmgamma`**`(int` *`n`*`, real` *`x`*`)`

Returns the natural logarithm of the multinomial gamma function with *n* dimensions applied to *x*.

$$\text{lmgamma}(n,x) = \begin{cases} \pi^{n(n-1)/4} \prod_{j=1}^{n} \Gamma(x + (1-j)/2) & \text{if } x \notin \{\dots,-3,-2,-1,0\} \\ \text{error} & \text{otherwise} \end{cases}$$

`real` **`gamma_p`**`(real` *a*`, real` *z*`)`

Returns the normalized lower incomplete gamma function of *a* and *z* defined for positive *a* and nonnegative *z*.

$$\text{gamma\_p}(a, z) = \begin{cases} \frac{1}{\Gamma(a)} \int_0^z t^{a-1} e^{-t} dt & \text{if } a > 0, z \geq 0 \\ \text{error} & \text{otherwise} \end{cases}$$

`real` **`gamma_q`**`(real` *a*`, real` *z*`)`

Returns the normalized upper incomplete gamma function of *a* and *z* defined for positive *a* and nonnegative *z*.

$$\text{gamma\_q}(a, z) = \begin{cases} \frac{1}{\Gamma(a)} \int_z^\infty t^{a-1} e^{-t} dt & \text{if } a > 0, z \geq 0 \\ \\ \text{error} & \text{otherwise} \end{cases}$$

`real` **`binomial_coefficient_log`**`(real` *x*`, real` *y*`)`

Returns the natural logarithm of the binomial coefficient of *x* and *y*. For non-negative integer inputs, the binomial coefficient function is written as $\binom{x}{y}$ and pronounced "*x* choose *y*." This function generalizes to real numbers using the gamma function.

For $0 \leq y \leq x$,

$$\text{binomial\_coefficient\_log}(x, y) = \log \Gamma(x + 1) - \log \Gamma(y + 1) - \log \Gamma(x - y + 1)$$

`real` **`bessel_first_kind`**`(int` *v*`, real` *x*`)`

Returns the Bessel function of the first kind with order *v* applied to *x*.

$$\text{bessel\_first\_kind}(v, x) = J_v(x)$$

where

$$J_v(x) = \left(\frac{1}{2}x\right)^v \sum_{k=0}^{\infty} \frac{\left(-\frac{1}{4}x^2\right)^k}{k!\,\Gamma(v + k + 1)}$$

`real` **`bessel_second_kind`**`(int` *v*`, real` *x*`)`

Returns the Bessel function of the second kind with order *v* applied to *x* defined for positive *x* and *v*.

For $x, \nu > 0$,

$$\text{bessel\_second\_kind}(\nu, x) = \begin{cases} Y_\nu(x) & \text{if } x > 0 \\ \text{error} & \text{otherwise} \end{cases}$$

where

$$Y_\nu(x) = \frac{J_\nu(x)\cos(\nu\pi) - J_{-\nu}(x)}{\sin(\nu\pi)}$$

`real` **`modified_bessel_first_kind`**`(int` *`v`*`, real` *`z`*`)`

Returns the modified Bessel function of the first kind with order *v* applied to *z* defined for all *z* and *v*.

$$\text{modified\_bessel\_first\_kind}(\nu, z) = I_\nu(z)$$

where

$$I_\nu(z) = \left(\frac{1}{2}z\right)^\nu \sum_{k=0}^{\infty} \frac{\left(\frac{1}{4}z^2\right)^k}{k!\Gamma(\nu + k + 1)}$$

`real` **`modified_bessel_second_kind`**`(int` *`v`*`, real` *`z`*`)`

Returns the modified Bessel function of the second kind with order *v* applied to *z* defined for positive *z* and *v*.

$$\text{modified\_bessel\_second\_kind}(\nu, z) = \begin{cases} K_\nu(z) & \text{if } z > 0 \\ \text{error} & \text{if } z \leq 0 \end{cases}$$

where

$$K_\nu(z) = \frac{\pi}{2} \cdot \frac{I_{-\nu}(z) - I_\nu(z)}{\sin(\nu\pi)}$$

`real` **`falling_factorial`**`(real` *`x`*`, real` *`n`*`)`

Returns the falling factorial of *x* with power *n* defined for positive *x* and real *n*.

$$\text{falling\_factorial}(x, n) = \begin{cases} (x)_n & \text{if } x > 0 \\ \text{error} & \text{if } x \leq 0 \end{cases}$$

where

$$(x)_n = \frac{\Gamma(x+1)}{\Gamma(x - n + 1)}$$

`real` **`log_falling_factorial`**`(real x, real n)`

Returns the log of the falling factorial of *x* with power *n* defined for positive *x* and real *n*.

$$\text{log\_falling\_factorial}(x, n) = \begin{cases} \log(x)_n & \text{if } x > 0 \\ \text{error} & \text{if } x \leq 0 \end{cases}$$

`real` **`rising_factorial`**`(real x, real n)`

Returns the rising factorial of *x* with power *n* defined for positive *x* and real *n*.

$$\text{rising\_factorial}(x, n) = \begin{cases} x^{(n)} & \text{if } x > 0 \\ \text{error} & \text{if } x \leq 0 \end{cases}$$

where

$$x^{(n)} = \frac{\Gamma(x+n)}{\Gamma(x)}$$

`real` **`log_rising_factorial`**`(real x, real n)`

Returns the log of the rising factorial of *x* with power *n* defined for positive *x* and real *n*.

$$\text{log\_rising\_factorial}(x, n) = \begin{cases} \log x^{(n)} & \text{if } x > 0 \\ \text{error} & \text{if } x \leq 0 \end{cases}$$

31.13. Composed Functions

The functions in this section are equivalent in theory to combinations of other functions. In practice, they are implemented to be more efficient and more numerically stable than defining them directly using more basic Stan functions.

`real` **`expm1`**`(real x)`

Returns the natural exponential of *x* minus 1.

$$\text{expm1}(x) = e^x - 1$$

`real` **`fma`**`(real x, real y, real z)`

Returns *z* plus the result of *x* multiplied by *y*.

$$\text{fma}(x, y, z) = x \cdot y + z$$

`real` **`multiply_log`**`(real x, real y)`

Returns the product of *x* and the natural logarithm of *y*.

$$\text{multiply\_log}(x, y) = \begin{cases} 0 & \text{if } x = y = 0 \\ x \log y & \text{if } x, y \neq 0 \\ \text{NaN} & \text{otherwise} \end{cases}$$

`real` **`log1p`**`(real x)`

Returns the natural logarithm of 1 plus *x*.

$$\text{log1p}(x) = \begin{cases} \log(1 + x) & \text{if } x \geq -1 \\ \text{NaN} & \text{otherwise} \end{cases}$$

`real` **`log1m`**`(real x)`

Returns the natural logarithm of 1 minus *x*.

$$\text{log1m}(x) = \begin{cases} \log(1 - x) & \text{if } x \leq 1 \\ \text{NaN} & \text{otherwise} \end{cases}$$

`real` **`log1p_exp`**`(real x)`

Returns the natural logarithm of one plus the natural exponentiation of *x*.

$$\text{log1p\_exp}(x) = \log(1 + \exp(x))$$

`real` **`log1m_exp`**`(real x)`

Returns the natural logarithm of one minus the natural exponentiation of *x*.

$$\text{log1m\_exp}(x) = \begin{cases} \log(1 - \exp(x)) & \text{if } x < 0 \\ \text{NaN} & \text{if } x \geq 0 \end{cases}$$

`real` **`log_diff_exp`**`(real x, real y)`

Returns the natural logarithm of the difference of the natural exponentiation of *x* and the natural exponentiation of *y*.

$$\text{log\_diff\_exp}(x, y) = \begin{cases} \log(\exp(x) - \exp(y)) & \text{if } x > y \\ \\ \text{NaN} & \text{otherwise} \end{cases}$$

`real` **`log_sum_exp`**`(real x, real y)`

Returns the natural logarithm of the sum of the natural exponentiation of *x* and the natural exponentiation of *y*.

$$\text{log\_sum\_exp}(x, y) = \log(\exp(x) + \exp(y))$$

`real` **`log_inv_logit`**`(real x)`

Returns the natural logarithm of the inverse logit function of *x*.

$$\text{log\_inv\_logit}(x) = \log \text{logit}^{-1}(x)$$

See Section 31.10 for a definition of inverse logit.

`real` **`log1m_inv_logit`**`(real x)`

Returns the natural logarithm of 1 minus the inverse logit function of *x*.

$$\text{log1m\_inv\_logit}(x) = \log(1 - \text{logit}^{-1}(x))$$

See Section 31.10 for a definition of inverse logit.

32. Array Operations

32.1. Reductions

The following operations take arrays as input and produce single output values. The boundary values for size 0 arrays are the unit with respect to the combination operation (min, max, sum, or product).

Minimum and Maximum

`real min(real x[])`

The minimum value in x, or $+\infty$ if x is empty

`int min(int x[])`

The minimum value in x, or raise exception if x is empty

`real max(real x[])`

The maximum value in x, or $-\infty$ if x is empty

`int max(int x[])`

The maximum value in x, or raise exception if x is empty

Sum, Product, and Log Sum of Exp

`int sum(int x[])`

The sum of the elements in x, or 0 if x is empty.

`real sum(real x[])`

The sum of the elements in x, or 0 if x is empty.

`real prod(real x[])`

The product of the elements in x, or 1 if x is empty.

`real prod(int x[])`

The product of the elements in x, or 1 if x is empty.

`real log_sum_exp(real x[])`

The natural logarithm of the sum of the exponentials of the elements in x, or $-\infty$ if the array is empty.

Moments

Moments are only defined for arrays x of size $N \geq 1$; it is an error to call them with arrays of size $N = 0$. The sample mean is defined by

$$\text{mean}(x) = \frac{1}{N} \sum_{n=1}^{N} x_n.$$

For $N \geq 2$, sample variance is defined for $N \geq 1$ by

$$\text{variance}(x) = \frac{1}{N-1} \sum_{n=1}^{N} (x_n - \text{mean}(x))^2$$

and sample standard deviation by

$$\text{sd}(x) = \sqrt{\text{variance}(x)},$$

For convenience, when $N = 1$, variance(x) and $\text{sd}(x)$ are defined to be 0.

`real` **`mean`**`(real` *`x`*`[])`

The sample mean of the elements in *x*

`real` **`variance`**`(real` *`x`*`[])`

The sample variance of the elements in *x*

`real` **`sd`**`(real` *`x`*`[])`

The sample standard deviation of elements in *x*

Distance and Squared Distance

`real` **`distance`**`(vector` *`x`*`, vector` *`y`*`)`

The Euclidean distance between *x* and *y*, defined by

$$\text{distance}(x, y) = \sqrt{\textstyle\sum_{n=1}^{N} (x[n] - y[n])^2},$$

where `N` is the size of *x* and *y*.

`real` **`distance`**`(vector` *`x`*`, row_vector` *`y`*`)`

The Euclidean distance between *x* and *y*

`real` **`distance`**`(row_vector` *`x`*`, vector` *`y`*`)`

The Euclidean distance between *x* and *y*

`real` **`distance`**`(row_vector x, row_vector y)`

The Euclidean distance between x and y

`real` **`squared_distance`**`(vector x, vector y[])`

The Euclidean distance between x and y, defined by

$$\text{squared\_distance}(x, y) = \sum_{n=1}^{N} (x[n] - y[n])^2,$$

where `N` is the size of x and y.

`real` **`squared_distance`**`(vector x, row_vector y[])`

The Euclidean distance between x and y

`real` **`squared_distance`**`(row_vector x, vector y[])`

The Euclidean distance between x and y

`real` **`squared_distance`**`(row_vector x, row_vector y[])`

The Euclidean distance between x and y

32.2. Array Size and Dimension Function

The size of an array or matrix can be obtained using the `dims()` function. The `dims()` function is defined to take an argument consisting of any variable with up to 8 array dimensions (and up to 2 additional matrix dimensions) and returns an array of integers with the dimensions. For example, if two variables are declared as follows,

```
real x[7,8,9];
matrix[8,9] y[7];
```

then calling `dims(x)` or `dims(y)` returns an integer array of size 3 containing the elements 7, 8, and 9 in that order.

The `size()` function extracts the number of elements in an array. The function is overloaded to apply to arrays of integers, reals, matrices, vectors, and row vectors.

`int[]` **`dims`**`(T x)`

Returns an integer array containing the dimensions of x; the type of the argument T can be any Stan type with up to 8 array dimensions.

`int` **`num_elements`**`(T[] x)`

Returns the total number of elements in the array x including all elements in contained arrays, vectors, and matrices. T can be any array type. For example, if `x` is of type `real[4,3]` then `num_elements(x)` is 12, and if `y` is declared as `matrix[3,4] y[5]`, then `size(y)` evaluates to 60.

`int` **`size`**`(`*T*`[] `*x*`)`

Returns the number of elements in the array *x*; the type of the array *T* can be any type, but the size is just the size of the top level array, not the total number of elements contained. For example, if `x` is of type `real[4,3]` then `size(x)` is 4.

32.3. Array Broadcasting

The following operations create arrays by repeating elements to fill an array of a specified size. These operations work for all input types *T*, including reals, integers, vectors, row vectors, matrices, or arrays.

T`[]` **`rep_array`**`(`*T* *x*`, int `*n*`)`

Return the *n* array with every entry assigned to *x*.

T`[ , ]` **`rep_array`**`(`*T* *x*`, int `*m*`, int `*n*`)`

Return the *m* by *n* array with every entry assigned to *x*.

T`[ , , ]` **`rep_array`**`(`*T* *x*`, int `*k*`, int `*m*`, int `*n*`)`

Return the *k* by *m* by *n* array with every entry assigned to *x*.

For example, `rep_array(1.0,5)` produces a real array (type `real[]`) of size 5 with all values set to 1.0. On the other hand, `rep_array(1,5)` produces an integer array (type `int[]`) of size 5 with all values set to 1. This distinction is important because it is not possible to assign an integer array to a real array. For example, the following example contrasts legal with illegal array creation and assignment

```
real y[5];
int x[5];

x <- rep_array(1,5);     // ok
y <- rep_array(1.0,5);   // ok

x <- rep_array(1.0,5);   // illegal
y <- rep_array(1,5);     // illegal

x <- y;                  // illegal
y <- x;                  // illegal
```

If the value being repeated `v` is a vector (i.e., `T` is `vector`), then `rep_array(v,27)` is a size 27 array consisting of 27 copies of the vector `v`.

```
vector[5] v;
vector[5] a[3];
...
a <- rep_array(v,3);  // fill a with copies of v
a[2,4] <- 9.0;        // v[4], a[1,4], a[2,4] unchanged
```

If the type T of x is itself an array type, then the result will be an array with one, two, or three added dimensions, depending on which of the rep_array functions is called. For instance, consider the following legal code snippet.

```
real a[5,6];
real b[3,4,5,6];
...
b <- rep_array(a,3,4); //  make (3 x 4) copies of a
b[1,1,1,1] <- 27.9;    //  a[1,1] unchanged
```

After the assignment to b, the value for b[j,k,m,n] is equal to a[m,n] where it is defined, for j in 1:3, k in 1:4, m in 1:5, and n in 1:6.

32.4. Sorting functions

Sorting can be used to sort values or the indices of those values in either ascending or descending order. For example, if v is declared as a real array of size 3, with values

$$\texttt{v} = (1, -10.3, 20.987),$$

then the various sort routines produce

$$\begin{aligned} \texttt{sort\_asc(v)} &= (-10.3, 1, 20.987) \\ \texttt{sort\_desc(v)} &= (20.987, 1, -10.3) \\ \texttt{sort\_indices\_asc(v)} &= (2, 1, 3) \\ \texttt{sort\_indices\_desc(v)} &= (3, 1, 2) \end{aligned}$$

real[] **sort_asc**(real[] *v*)

Sort the elements of v in ascending order

int[] **sort_asc**(int[] *v*)

Sort the elements of v in ascending order

real[] **sort_desc**(real[] *v*)

Sort the elements of v in descending order

`int[]` **`sort_desc`**`(int[]` *v*`)`

Sort the elements of *v* in descending order

`int[]` **`sort_indices_asc`**`(real[]` *v*`)`

Return an array of indices between 1 and the size of *v*, sorted to index *v* in ascending order.

`int[]` **`sort_indices_asc`**`(int[]` *v*`)`

Return an array of indices between 1 and the size of *v*, sorted to index *v* in ascending order.

`int[]` **`sort_indices_desc`**`(real[]` *v*`)`

Return an array of indices between 1 and the size of *v*, sorted to index *v* in descending order.

`int[]` **`sort_indices_desc`**`(int[]` *v*`)`

Return an array of indices between 1 and the size of *v*, sorted to index *v* in descending order.

`int` **`rank`**`(real[]` *v*`, int` *s*`)`

Number of components of *v* less than *v[s]*

`int` **`rank`**`(int[]` *v*`, int` *s*`)`

Number of components of *v* less than *v[s]*

33. Matrix Operations

33.1. Integer-Valued Matrix Size Functions

`int` **`num_elements`**`(vector` *x*`)`

The total number of elements in the vector *x* (same as function `rows`)

`int` **`num_elements`**`(row_vector` *x*`)`

The total number of elements in the vector *x* (same as function `cols`)

`int` **`num_elements`**`(matrix` *x*`)`

The total number of elements in the matrix *x*. For example, if `x` is a 5×3 matrix, then `num_elements(x)` is 15

`int` **`rows`**`(vector` *x*`)`

The number of rows in the vector *x*

`int` **`rows`**`(row_vector` *x*`)`

The number of rows in the row vector *x*, namely 1

`int` **`rows`**`(matrix` *x*`)`

The number of rows in the matrix *x*

`int` **`cols`**`(vector` *x*`)`

The number of columns in the vector *x*, namely 1

`int` **`cols`**`(row_vector` *x*`)`

The number of columns in the row vector *x*

`int` **`cols`**`(matrix` *x*`)`

The number of columns in the matrix *x*

33.2. Matrix Arithmetic Operators

Stan supports the basic matrix operations using infix, prefix and postfix operations. This section lists the operations supported by Stan along with their argument and result types.

Negation Prefix Operators

`vector` **`operator-`**`(vector` *x*`)`

The negation of the vector *x*

`row_vector` **`operator-`**`(row_vector` *x*`)`

The negation of the row vector *x*

`matrix` **`operator-`**`(matrix` *x*`)`

The negation of the matrix *x*

Infix Matrix Operators

`vector` **`operator+`**`(vector` *x*`, vector` *y*`)`

The sum of the vectors *x* and *y*

`row_vector` **`operator+`**`(row_vector` *x*`, row_vector` *y*`)`

The sum of the row vectors *x* and *y*

`matrix` **`operator+`**`(matrix` *x*`, matrix` *y*`)`

The sum of the matrices *x* and *y*

`vector` **`operator-`**`(vector` *x*`, vector` *y*`)`

The difference between the vectors *x* and *y*

`row_vector` **`operator-`**`(row_vector` *x*`, row_vector` *y*`)`

The difference between the row vectors *x* and *y*

`matrix` **`operator-`**`(matrix` *x*`, matrix` *y*`)`

The difference between the matrices *x* and *y*

`vector` **`operator*`**`(real` *x*`, vector` *y*`)`

The product of the scalar *x* and vector *y*

`row_vector` **`operator*`**`(real` *x*`, row_vector` *y*`)`

The product of the scalar *x* and the row vector *y*

`matrix` **`operator*`**`(real` *x*`, matrix` *y*`)`

The product of the scalar *x* and the matrix *y*

`vector` **`operator*`**`(vector` *x*`, real` *y*`)`

The product of the scalar *y* and vector *x*

`matrix` **`operator*`**`(vector` *x*`, row_vector` *y*`)`

The product of the vector *x* and row vector *y*

`row_vector` **`operator*`**`(row_vector` *x*`, real` *y*`)`

The product of the scalar *y* and row vector *x*

`real` **`operator*`**`(row_vector` *x*`, vector` *y*`)`

The product of the row vector *x* and vector *y*

`row_vector` **`operator*`**`(row_vector` *x*`, matrix` *y*`)`

The product of the row vector *x* and matrix *y*

`matrix` **`operator*`**`(matrix` *x*`, real` *y*`)`

The product of the scalar *y* and matrix *x*

`vector` **`operator*`**`(matrix` *x*`, vector` *y*`)`

The product of the matrix *x* and vector *y*

`matrix` **`operator*`**`(matrix` *x*`, matrix` *y*`)`

The product of the matrices *x* and *y*

Broadcast Infix Operators

`vector` **`operator+`**`(vector` *x*`, real` *y*`)`

The result of adding *y* to every entry in the vector *x*

`vector` **`operator+`**`(real` *x*`, vector` *y*`)`

The result of adding *x* to every entry in the vector *y*

`row_vector` **`operator+`**`(row_vector` *x*`, real` *y*`)`

The result of adding *y* to every entry in the row vector *x*

`row_vector` **`operator+`**`(real` *x*`, row_vector` *y*`)`

The result of adding *x* to every entry in the row vector *y*

`matrix` **`operator+`**`(matrix` *x*`, real` *y*`)`

The result of adding *y* to every entry in the matrix *x*

`matrix` **`operator+`**`(real` *x*`, matrix` *y*`)`

The result of adding *x* to every entry in the matrix *y*

`vector` **`operator-`**`(vector` *x*`, real` *y*`)`

The result of subtracting *y* from every entry in the vector *x*

`vector` **`operator-`**`(real` *x*`, vector` *y*`)`

The result of adding *x* to every entry in the negation of the vector *y*

`row_vector` **`operator-`**`(row_vector` *x*`, real` *y*`)`

The result of subtracting *y* from every entry in the row vector *x*

`row_vector` **`operator-`**`(real` *x*`, row_vector` *y*`)`

The result of adding *x* to every entry in the negation of the row vector *y*

`matrix` **`operator-`**`(matrix` *x*`, real` *y*`)`

The result of subtracting *y* from every entry in the matrix *x*

`matrix` **`operator-`**`(real` *x*`, matrix` *y*`)`

The result of adding *x* to every entry in negation of the matrix *y*

`vector` **`operator/`**`(vector` *x*`, real` *y*`)`

The result of dividing each entry in the vector *x* by *y*

`row_vector` **`operator/`**`(row_vector` *x*`, real` *y*`)`

The result of dividing each entry in the row vector *x* by *y*

`matrix` **`operator/`**`(matrix` *x*`, real` *y*`)`

The result of dividing each entry in the matrix *x* by *y*

Elementwise Products

`vector` **`operator.*`**`(vector` *x*`, vector` *y*`)`

The elementwise product of *y* and *x*

`row_vector` **`operator.*`**`(row_vector` *x*`, row_vector` *y*`)`

The elementwise product of *y* and *x*

`matrix` **`operator.*`**`(matrix` *x*`, matrix` *y*`)`

The elementwise product of *y* and *x*

`vector` **`operator./`**`(vector` *x*`, vector` *y*`)`

The elementwise quotient of *y* and *x*

`vector` **`operator./`**`(vector` *x*`, real` *y*`)`

The elementwise quotient of *y* and *x*

`vector` **`operator./`**`(real` *x*`, vector` *y*`)`

The elementwise quotient of *y* and *x*

`row_vector` **`operator./`**`(row_vector` *x*`, row_vector` *y*`)`

The elementwise quotient of *y* and *x*

`row_vector` **`operator./`**`(row_vector` *x*`, real` *y*`)`

The elementwise quotient of *y* and *x*

`row_vector` **`operator./`**`(real` *x*`, row_vector` *y*`)`

The elementwise quotient of *y* and *x*

`matrix` **`operator./`**`(matrix` *x*`, matrix` *y*`)`

The elementwise quotient of *y* and *x*

`matrix` **`operator./`**`(matrix` *x*`, real` *y*`)`

The elementwise quotient of *y* and *x*

`matrix` **`operator./`**`(real` *x*`, matrix` *y*`)`

The elementwise quotient of *y* and *x*

Elementwise Logarithms

`vector` **`log`**`(vector` *x*`)`

The elementwise natural logarithm of *x*

`row_vector` **`log`**`(row_vector` *x*`)`

The elementwise natural logarithm of *x*

`matrix` **`log`**`(matrix` *x*`)`

The elementwise natural logarithm of *x*

`vector` **`exp`**`(vector` *x*`)`

The elementwise exponential of *x*

`row_vector` **`exp`**`(row_vector` *x*`)`

The elementwise exponential of *x*

`matrix` **`exp`**`(matrix` *x*`)`

The elementwise exponential of *x*

Cumulative Sums

The cumulative sum of a sequence $x_1, \ldots, x_N$ is the sequence $y_1, \ldots, y_N$, where

$$y_n = \sum_{m=1}^{n} x_n.$$

`real[]` **`cumulative_sum`**`(real[]` *x*`)`
The cumulative sum of *x*

`vector` **`cumulative_sum`**`(vector` *v*`)`
The cumulative sum of *v*

`row_vector` **`cumulative_sum`**`(row_vector` *rv*`)`
The cumulative sum of *rv*

Dot Products

`real` **`dot_product`**`(vector` *x*`, vector` *y*`)`
The dot product of *x* and *y*

`real` **`dot_product`**`(vector` *x*`, row_vector` *y*`)`
The dot product of *x* and *y*

`real` **`dot_product`**`(row_vector` *x*`, vector` *y*`)`
The dot product of *x* and *y*

`real` **`dot_product`**`(row_vector` *x*`, row_vector` *y*`)`
The dot product of *x* and *y*

`row_vector` **`columns_dot_product`**`(vector` *x*`, vector` *y*`)`
The dot product of the columns of *x* and *y*

`row_vector` **`columns_dot_product`**`(row_vector` *x*`, row_vector` *y*`)`
The dot product of the columns of *x* and *y*

`row_vector` **`columns_dot_product`**`(matrix` *x*`, matrix` *y*`)`
The dot product of the columns of *x* and *y*

`vector` **`rows_dot_product`**`(vector` *x*`, vector` *y*`)`
The dot product of the rows of *x* and *y*

`vector` **`rows_dot_product`**`(row_vector` *x*`, row_vector` *y*`)`
The dot product of the rows of *x* and *y*

`vector` **`rows_dot_product`**`(matrix` *x*`, matrix` *y*`)`

The dot product of the rows of *x* and *y*

`real` **`dot_self`**`(vector` *x*`)`

The dot product of the vector *x* with itself

`real` **`dot_self`**`(row_vector` *x*`)`

The dot product of the row vector *x* with itself

`row_vector` **`columns_dot_self`**`(vector` *x*`)`

The dot product of the columns of *x* with themselves

`row_vector` **`columns_dot_self`**`(row_vector` *x*`)`

The dot product of the columns of *x* with themselves

`row_vector` **`columns_dot_self`**`(matrix` *x*`)`

The dot product of the columns of *x* with themselves

`vector` **`rows_dot_self`**`(vector` *x*`)`

The dot product of the rows of *x* with themselves

`vector` **`rows_dot_self`**`(row_vector` *x*`)`

The dot product of the rows of *x* with themselves

`vector` **`rows_dot_self`**`(matrix` *x*`)`

The dot product of the rows of *x* with themselves

Specialized Products

`matrix` **`tcrossprod`**`(matrix` *x*`)`

The product of *x* postmultiplied by its own transpose, similar to the tcrossprod(x) function in R. The result is a symmetric matrix $\mathrm{x}\,\mathrm{x}^\top$.

`matrix` **`crossprod`**`(matrix` *x*`)`

The product of *x* premultiplied by its own transpose, similar to the crossprod(x) function in R. The result is a symmetric matrix $\mathrm{x}^\top\,\mathrm{x}$.

The following functions all provide shorthand forms for common expressions, which are also much more efficient.

`matrix` **`quad_form`**`(matrix` *A*`, matrix` *B*`)`

The quadratic form, i.e., `B' * A * B`.

`real` **`quad_form`**`(matrix` *A*`, vector` *B*`)`

The quadratic form, i.e., `B' * A * B`.

`matrix` **`quad_form_diag`**`(matrix` *m*`, vector` *v*`)`

The quadratic form using the column vector *v* as a diagonal matrix, i.e., `diag_matrix(`*v*`) *` *m* `* diag_matrix(`*v*`)`.

`matrix` **`quad_form_diag`**`(matrix` *m*`, row_vector` *rv*`)`

The quadratic form using the row vector *rv* as a diagonal matrix, i.e., `diag_matrix(`*rv*`) *` *m* `* diag_matrix(`*rv*`)`.

`matrix` **`quad_form_sym`**`(matrix` *A*`, matrix` *B*`)`

Similarly to quad_form, gives `B' * A * B`, but additionally checks if A is symmetric and ensures that the result is also symmetric.

`real` **`quad_form_sym`**`(matrix` *A*`, vector` *B*`)`

Similarly to quad_form, gives `B' * A * B`, but additionally checks if A is symmetric and ensures that the result is also symmetric.

`real` **`trace_quad_form`**`(matrix` *A*`, matrix` *B*`)`

The trace of the quadratic form, i.e., `trace(B' * A * B)`.

`real` **`trace_gen_quad_form`**`(matrix` *D*`,matrix` *A*`, matrix` *B*`)`

The trace of a generalized quadratic form, i.e., `trace(D * B' * A * B).`

`matrix` **`multiply_lower_tri_self_transpose`**`(matrix` *x*`)`

The product of the lower triangular portion of *x* (including the diagonal) times its own transpose; that is, if `L` is a matrix of the same dimensions as *x* with `L(m,n)` equal to *x*`(m,n)` for $n \leq m$ and `L(m,n)` equal to 0 if $n > m$, the result is the symmetric matrix $L L^{\top}$. This is a specialization of tcrossprod(x) for lower-triangular matrices. The input matrix does not need to be square.

`matrix` **`diag_pre_multiply`**`(vector` *v*`, matrix` *m*`)`

Return the product of the diagonal matrix formed from the vector *v* and the matrix *m*, i.e., `diag_matrix(`*v*`) *` *m*.

`matrix` **`diag_pre_multiply`**`(row_vector` *rv*`, matrix` *m*`)`

Return the product of the diagonal matrix formed from the vector *rv* and the matrix *m*, i.e., `diag_matrix(`*rv*`) *` *m*.

`matrix` **`diag_post_multiply`**`(matrix` *m*`, vector` *v*`)`

Return the product of the matrix *m* and the diagonal matrix formed from the vector *v*, i.e., *m* `* diag_matrix(`*v*`)`.

`matrix` **`diag_post_multiply`**`(matrix` *m*`, row_vector` *rv*`)`

Return the product of the matrix *m* and the diagonal matrix formed from the the row vector *rv*, i.e., *m* `* diag_matrix(`*rv*`)`.

33.3. Reductions

Log Sum of Exponents

`real` **`log_sum_exp`**`(vector` *x*`)`

The natural logarithm of the sum of the exponentials of the elements in *x*

`real` **`log_sum_exp`**`(row_vector` *x*`)`

The natural logarithm of the sum of the exponentials of the elements in *x*

`real` **`log_sum_exp`**`(matrix` *x*`)`

The natural logarithm of the sum of the exponentials of the elements in *x*

Minimum and Maximum

`real` **`min`**`(vector` *x*`)`

The minimum value in *x*, or $+\infty$ if *x* is empty

`real` **`min`**`(row_vector` *x*`)`

The minimum value in *x*, or $+\infty$ if *x* is empty

`real` **`min`**`(matrix` *x*`)`

The minimum value in *x*, or $+\infty$ if *x* is empty

`real` **`max`**`(vector` *x*`)`

The maximum value in *x*, or $-\infty$ if *x* is empty

`real` **`max`**`(row_vector` *x*`)`

The maximum value in *x*, or $-\infty$ if *x* is empty

`real` **`max`**`(matrix` *x*`)`

The maximum value in *x*, or $-\infty$ if *x* is empty

Sums and Products

`real` **`sum`**`(vector` *x*`)`

The sum of the values in *x*, or 0 if *x* is empty

`real` **`sum`**`(row_vector x)`
The sum of the values in x, or 0 if x is empty

`real` **`sum`**`(matrix x)`
The sum of the values in x, or 0 if x is empty

`real` **`prod`**`(vector x)`
The product of the values in x, or 1 if x is empty

`real` **`prod`**`(row_vector x)`
The product of the values in x, or 1 if x is empty

`real` **`prod`**`(matrix x)`
The product of the values in x, or 1 if x is empty

Sample Moments

Full definitions are provided for sample moments in Section 32.1.

`real` **`mean`**`(vector x)`
The sample mean of the values in x; see Section 32.1 for details.

`real` **`mean`**`(row_vector x)`
The sample mean of the values in x; see Section 32.1 for details.

`real` **`mean`**`(matrix x)`
The sample mean of the values in x; see Section 32.1 for details.

`real` **`variance`**`(vector x)`
The sample variance of the values in x; see Section 32.1 for details.

`real` **`variance`**`(row_vector x)`
The sample variance of the values in x; see Section 32.1 for details.

`real` **`variance`**`(matrix x)`
The sample variance of the values in x; see Section 32.1 for details.

`real` **`sd`**`(vector x)`
The sample standard deviation of the values in x; see Section 32.1 for details.

`real` **`sd`**`(row_vector x)`
The sample standard deviation of the values in x; see Section 32.1 for details.

`real sd(matrix x)`

The sample standard deviation of the values in *x*; see Section 32.1 for details.

33.4. Broadcast Functions

The following broadcast functions allow vectors, row vectors and matrices to be created by copying a single element into all of their cells. Matrices may also be created by stacking copies of row vectors vertically or stacking copies of column vectors horizontally.

`vector rep_vector(real x, int m)`

Return the size *m* (column) vector consisting of copies of *x*.

`row_vector rep_row_vector(real x, int n)`

Return the size *n* row vector consisting of copies of *x*.

`matrix rep_matrix(real x, int m, int n)`

Return the *m* by *n* matrix consisting of copies of *x*.

`matrix rep_matrix(vector v, int n)`

Return the *m* by *n* matrix consisting of *n* copies of the (column) vector *v* of size *m*.

`matrix rep_matrix(row_vector rv, int m)`

Return the *m* by *n* matrix consisting of *m* copies of the row vector *rv* of size *n*.

Unlike the situation with array broadcasting (see Section 32.3), where there is a distinction between integer and real arguments, the following two statements produce the same result for vector broadcasting; row vector and matrix broadcasting behave similarly.

```
vector[3] x;
x <- rep_vector(1, 3);
x <- rep_vector(1.0, 3);
```

There are no integer vector or matrix types, so integer values are automatically promoted.

33.5. Slice and Package Functions

Diagonal Matrices

`vector` **`diagonal`**`(matrix` *x*`)`

The diagonal of the matrix *x*

`matrix` **`diag_matrix`**`(vector` *x*`)`

The diagonal matrix with diagonal *x*

Columns and Rows

`vector` **`col`**`(matrix` *x*`, int` *n*`)`

The *n*-th column of matrix *x*

`row_vector` **`row`**`(matrix` *x*`, int` *m*`)`

The *m*-th row of matrix *x*

The `row` function is special in that it may be used as an lvalue in an assignment statement; for more information on assignment, see Section 24.1. The row function is also special in that the indexing notation `x[m]` is just an alternative way of writing `row(x,m)`. The `col` function may *not* be used as an lvalue, nor is there an indexing based shorthand for it.

Block Operations

Matrix Slicing Operations

Block operations may be used to extract a sub-block of a matrix.

`matrix` **`block`**`(matrix` *x*`, int` *i*`, int` *j*`, int` *n_rows*`, int` *n_cols*`)`

Return the submatrix of *x* that starts at row *i* and column *j* and extends *n_rows* rows and *n_cols* columns.

The sub-row and sub-column operations may be used to extract a slice of row or column from a matrix

`vector` **`sub_col`**`(matrix` *x*`, int` *i*`, int` *j*`, int` *n_rows*`)`

Return the sub-column of *x* that starts at row *i* and column *j* and extends *n_rows* rows and 1 column.

`row_vector` **`sub_row`**`(matrix` *x*`, int` *i*`, int` *j*`, int` *n_cols*`)`

Return the sub-row of *x* that starts at row *i* and column *j* and extends 1 row and *n_cols* columns.

Vector and Array Slicing Operations

The head operation extracts the first *n* elements of a vector and the tail operation the last. The segment operation extracts an arbitrary subvector.

`vector` **`head`**`(vector` *`v`*`, int` *`n`*`)`

Return the vector consisting of the first *n* elements of *v*.

`row_vector` **`head`**`(row_vector` *`rv`*`, int` *`n`*`)`

Return the row vector consisting of the first *n* elements of *rv*.

`T[]` **`head`**`(T[]` *`sv`*`, int` *`n`*`)`

Return the standard vector consisting of the first *n* elements of *sv*; applies to up to three-dimensional arrays containing any type of elements T.

`vector` **`tail`**`(vector` *`v`*`, int` *`n`*`)`

Return the vector consisting of the last *n* elements of *v*.

`row_vector` **`tail`**`(row_vector` *`rv`*`, int` *`n`*`)`

Return the row vector consisting of the last *n* elements of *rv*.

`T[]` **`tail`**`(T[]` *`sv`*`, int` *`n`*`)`

Return the standard vector consisting of the last *n* elements of *sv*; applies to up to three-dimensional arrays containing any type of elements T.

`vector` **`segment`**`(vector` *`v`*`, int` *`i`*`, int` *`n`*`)`

Return the vector consisting of the *n* elements of *v* starting at *i*; i.e., elements *i* through through $i + n - 1$.

`row_vector` **`segment`**`(row_vector` *`v`*`, int` *`i`*`, int` *`n`*`)`

Return the row vector consisting of the *n* elements of *rv* starting at *i*; i.e., elements *i* through through $i + n - 1$.

`T[]` **`segment`**`(T[]` *`sv`*`, int` *`i`*`, int` *`n`*`)`

Return the standard vector consisting of the *n* elements of *sv* starting at *i*; i.e., elements *i* through through $i + n - 1$. Applies to up to three-dimensional arrays containing any type of elements T.

Concatenation Operations

Horizontal concatenation

`matrix` **`append_col`**`(matrix` *`x`*`, matrix` *`y`*`)`

Combine matrices *x* and *y* by columns. The matrices must have the same number of rows.

`matrix` **`append_col`**`(matrix` *x*`, vector` *y*`)`

Combine matrix *x* and vector *y* by columns. The matrix and the vector must have the same number of rows.

`matrix` **`append_col`**`(vector` *x*`, matrix` *y*`)`

Combine vector *x* and matrix *y* by columns. The vector and the matrix must have the same number of rows.

`matrix` **`append_col`**`(vector` *x*`, vector` *y*`)`

Combine vectors *x* and *y* by columns. The vectors must have the same number of rows.

`row_vector` **`append_col`**`(row_vector` *x*`, row_vector` *y*`)`

Combine row vectors *x* and *y* of any size into another row vector.

Vertical concatenation

`matrix` **`append_row`**`(matrix` *x*`, matrix` *y*`)`

Combine matrices *x* and *y* by columns. The matrices must have the same number of rows.

`matrix` **`append_row`**`(matrix` *x*`, row_vector` *y*`)`

Combine matrix *x* and row vector *y* by columns. The matrix and the row vector must have the same number of rows.

`matrix` **`append_row`**`(row_vector` *x*`, matrix` *y*`)`

Combine row vector *x* and matrix *y* by columns. The row vector and the matrix must have the same number of rows.

`matrix` **`append_row`**`(row_vector` *x*`, row_vector` *y*`)`

Combine row vectors *x* and *y* by columns. The row vectors must have the same number of rows.

`vector` **`append_row`**`(vector` *x*`, vector` *y*`)`

Combine vectors *x* and *y* of any size into another vector.

Transposition Postfix Operator

`matrix` **`operator'`**`(matrix` *x*`)`

The transpose of the matrix *x*, written as `x'`

`row_vector` **`operator'`**`(vector` *x*`)`

The transpose of the vector *x*, written as `x'`

`vector` **`operator'`**`(row_vector` *x*`)`

The transpose of the row vector *x*, written as `x'`

33.6. Special Matrix Functions

The softmax function maps $y \in \mathbb{R}^K$ to the K-simplex by

$$\text{softmax}(y) = \frac{\exp(y)}{\sum_{k=1}^{K} \exp(y_k)},$$

where $\exp(y)$ is the componentwise exponentiation of y. Softmax is usually calculated on the log scale,

$$\log \text{softmax}(y) \; = \; y - \log \sum_{k=1}^{K} \exp(y_k) \; = \; y - \text{log\_sum\_exp}(y).$$

Stan provides the following functions for softmax and its log.

`vector` **`softmax`**`(vector` *x*`)`

The softmax of *x*

`vector` **`log_softmax`**`(vector` *x*`)`

The natural logarithm of the softmax of *x*

33.7. Linear Algebra Functions and Solvers

Matrix Division Infix Operators

`row_vector` **`operator/`**`(row_vector` *b*`, matrix` *A*`)`

The right division of *b* by *A*; equivalently *b* `* inverse(`*A*`)`

`matrix` **`operator/`**`(matrix` *B*`, matrix` *A*`)`

The right division of *B* by *A*; equivalently *B* `* inverse(`*A*`)`

`vector` **`operator\`**`(matrix A, vector b)`

T he left division of *b* by *A*; equivalently `inverse(A) * b`

`matrix` **`operator\`**`(matrix A, matrix B)`

T he left division of *B* by *A*; equivalently `inverse(A) * B`

Lower-Triangular Matrix-Division Functions

There are four division functions which use lower triangular views of a matrix. The lower triangular view of a matrix $\text{tri}(A)$ is used in the definitions and defined by

$$\text{tri}(A)[m,n] = \begin{cases} A[m,n] & \text{if } m \geq n, \text{ and} \\ 0 & \text{otherwise.} \end{cases}$$

When a lower triangular view of a matrix is used, the elements above the diagonal are ignored.

`row_vector` **`mdivide_right_tri_low`**`(row_vector B, matrix A)`

The right division of *B* by `tri(A)`, a lower triangular view of *A*; equivalently `B * inverse(tri(A))`

`matrix` **`mdivide_right_tri_low`**`(matrix B, matrix A)`

The right division of *B* by `tri(A)`, a lower triangular view of *A*; equivalently `B * inverse(tri(A))`

`vector` **`mdivide_left_tri_low`**`(matrix A, vector B)`

T he left division of *B* by a triangular view of `tri(A)`, a lower triangular view of *A*; equivalently `inverse(tri(A)) * B`

`matrix` **`mdivide_left_tri_low`**`(matrix A, matrix B)`

T he left division of *B* by a triangular view of `tri(A)`, a lower triangular view of *A*; equivalently `inverse(tri(A)) * B`

Linear Algebra Functions

Trace

`real` **`trace`**`(matrix A)`

The trace of *A*, or 0 if *A* is empty; *A* is not required to be diagonal

Determinants

`real` **`determinant`**`(matrix A)`

The determinant of A

`real` **`log_determinant`**`(matrix A)`

The log of the absolute value of the determinant of A

Inverses

`matrix` **`inverse`**`(matrix A)`

The inverse of A

`matrix` **`inverse_spd`**`(matrix A)`

The inverse of A where A is symmetric, positive definite. This version is faster and more arithmetically stable when the input is symmetric and positive definite.

Eigendecomposition

`vector` **`eigenvalues_sym`**`(matrix A)`

The vector of eigenvalues of a symmetric matrix A in ascending order

`matrix` **`eigenvectors_sym`**`(matrix A)`

The matrix with the (column) eigenvectors of symmetric matrix A in the same order as returned by the function `eigenvalues_sym`

Because multiplying an eigenvector by -1 results in an eigenvector, eigenvectors returned by a decomposition are only identified up to a sign change. In order to compare the eigenvectors produced by Stan's eigendecomposition to others, signs may need to be normalized in some way, such as by fixing the sign of a component, or doing comparisons allowing a multiplication by -1.

The condition number of a symmetric matrix is defined to be the ratio of the largest eigenvalue to the smallest eigenvalue. Large condition numbers lead to difficulty in numerical algorithms such as computing inverses, and thus known as "ill conditioned." The ratio can even be infinite in the case of singular matrices (i.e., those with eigenvalues of 0).

QR Decomposition

`matrix` **`qr_Q`**`(matrix A)`

The orthogonal matrix in the QR decomposition of A

`matrix` **`qr_R`**`(matrix A)`

The upper triangular matrix in the QR decomposition of A

Multiplying a column of an orthogonal matrix by -1 still results in an orthogonal matrix, and you can multiply the corresponding row of the upper triangular matrix by -1 without changing the product. Thus, Stan adopts the normalization that the diagonal elements of the upper triangular matrix are strictly positive and the columns of the orthogonal matrix are rescaled if necessary. The input matrix A need not be square but must have at least as many rows as it has columns. Also, this QR decomposition algorithm does not utilize pivoting and thus is faster but may be numerically unstable.

Cholesky Decomposition

Every symmetric, positive-definite matrix (such as a correlation or covariance matrix) has a Cholesky decomposition. If Σ is a symmetric, positive-definite matrix, its Cholesky decomposition is the lower-triangular vector L such that

$$\Sigma = L\,L^{\top}.$$

`matrix` **`cholesky_decompose`**`(matrix A)`

The lower-triangular Cholesky factor of the symmetric positive-definite matrix A

Singular Value Decomposition

Stan only provides functions for the singular values, not for the singular vectors involved in a singular value decomposition (SVD).

`vector` **`singular_values`**`(matrix A)`

The singular values of A in descending order

33.8. Sort Functions

See Section 32.4 for examples of how the functions work.

`vector` **`sort_asc`**`(vector v)`

Sort the elements of v in ascending order

`row_vector` **`sort_asc`**`(row_vector v)`

Sort the elements of v in ascending order

`vector` **`sort_desc`**`(vector` *v*`)`

Sort the elements of *v* in descending order

`row_vector` **`sort_desc`**`(row_vector` *v*`)`

Sort the elements of *v* in descending order

`int[]` **`sort_indices_asc`**`(vector` *v*`)`

Return an array of indices between 1 and the size of *v*, sorted to index *v* in ascending order.

`int[]` **`sort_indices_asc`**`(row_vector` *v*`)`

Return an array of indices between 1 and the size of *v*, sorted to index *v* in ascending order.

`int[]` **`sort_indices_desc`**`(vector` *v*`)`

Return an array of indices between 1 and the size of *v*, sorted to index *v* in descending order.

`int[]` **`sort_indices_desc`**`(row_vector` *v*`)`

Return an array of indices between 1 and the size of *v*, sorted to index *v* in descending order.

`int` **`rank`**`(vector` *v*`, int` *s*`)`

Number of components of *v* less than *v[s]*

`int` **`rank`**`(row_vector` *v*`, int` *s*`)`

Number of components of *v* less than *v[s]*

34. Mixed Operations

These functions perform conversions between Stan containers matrix, vector, row vector and arrays.

Whenever a conversion implies reduction of dimensionality (like converting a matrix to a vector or a two dimensional array to a one dimensional array), the conversion is proceed in row-major order when the input is an array and in column-major order when the input is a vector, a row vector or a matrix.

If the dimensionality is preserved (like when converting a matrix to a two dimensional array), then the indexes are also fully preserved which implies easy reversibility of the operation.

`matrix` **`to_matrix`**`(matrix` *`m`*`)`

Return the matrix *m* itself.

`matrix` **`to_matrix`**`(vector` *`v`*`)`

Convert the column vector *v* to a `size(`*`v`*`)` by 1 matrix.

`matrix` **`to_matrix`**`(row_vector` *`v`*`)`

Convert the row vector *v* to a 1 by `size(`*`v`*`)` matrix.

`matrix` **`to_matrix`**`(real[,]` *`a`*`)`

Convert the two dimensional array *a* to a matrix with the same dimensions and indexing order.

`matrix` **`to_matrix`**`(int[,]` *`a`*`)`

Convert the two dimensional array *a* to a matrix with the same dimensions and indexing order.

`vector` **`to_vector`**`(matrix` *`m`*`)`

Convert the matrix *m* to a column vector in column-major order.

`vector` **`to_vector`**`(vector` *`v`*`)`

Return the column vector *v* itself.

`vector` **`to_vector`**`(row_vector` *`v`*`)`

Convert the row vector *v* to a column vector.

`vector` **`to_vector`**`(real[]` *`a`*`)`

Convert the one-dimensional array *a* to a column vector.

`vector` **`to_vector`**`(int[]` *`a`*`)`

Convert the one-dimensional integer array *a* to a column vector.

`row_vector` **`to_row_vector`**`(matrix` *m*`)`

Convert the matrix *m* to a row vector in column-major order.

`row_vector` **`to_row_vector`**`(vector` *v*`)`

Convert the column vector *v* to a row vector.

`row_vector` **`to_row_vector`**`(row_vector` *v*`)`

Return the row vector *v* itself.

`row_vector` **`to_row_vector`**`(real[]` *a*`)`

Convert the one-dimensional array *a* to a row vector.

`row_vector` **`to_row_vector`**`(int[]` *a*`)`

Convert the one-dimensional array *a* to a row vector.

`real[,]` **`to_array_2d`**`(matrix` *m*`)`

Convert the matrix *m* to a two dimensional array with the same dimensions and indexing order.

`real[]` **`to_array_1d`**`(vector` *v*`)`

Convert the column vector *v* to a one-dimensional array.

`real[]` **`to_array_1d`**`(row_vector` *v*`)`

Convert the row vector *v* to a one-dimensional array.

`real[]` **`to_array_1d`**`(matrix` *m*`)`

Convert the matrix *m* to a one-dimensional array in column-major order.

`real[]` **`to_array_1d`**`(real[...]` *a*`)`

Convert the array *a* (of any dimension up to 10) to a one-dimensional array in row-major order.

`int[]` **`to_array_1d`**`(int[...]` *a*`)`

Convert the array *a* (of any dimension up to 10) to a one-dimensional array in row-major order.

Part V

Discrete Distributions

35. Binary Distributions

Binary probability distributions have support on $\{0, 1\}$, where 1 represents the value true and 0 the value false.

35.1. Bernoulli Distribution

Probability Mass Function

If $\theta \in [0, 1]$, then for $y \in \{0, 1\}$,

$$\text{Bernoulli}(y|\theta) = \begin{cases} \theta & \text{if } y = 1, \text{ and} \\ 1 - \theta & \text{if } y = 0. \end{cases}$$

Sampling Statement

`y ~ bernoulli(theta);`

Increment log probability with `bernoulli_log(y,theta)`, dropping constant additive terms; Section 24.3 explains sampling statements.

Stan Functions

`real bernoulli_log(ints y, reals theta)`

The log Bernoulli probability mass of *y* given chance of success *theta*

`real bernoulli_cdf(ints y, reals theta)`

The Bernoulli cumulative distribution function of *y* given chance of success *theta*

`real bernoulli_cdf_log(ints y, reals theta)`

The log of the Bernoulli cumulative distribution function of *y* given chance of success *theta*

`real bernoulli_ccdf_log(ints y, reals theta)`

The log of the Bernoulli complementary cumulative distribution function of *y* given chance of success *theta*

`int bernoulli_rng(real theta)`

Generate a Bernoulli variate with chance of success *theta*; may only be used in generated quantities block

35.2. Bernoulli Distribution, Logit Parameterization

Stan also supplies a direct parameterization in terms of a logit-transformed chance-of-success parameter. This parameterization is more numerically stable if the chance-of-success parameter is on the logit scale, as with the linear predictor in a logistic regression.

Probability Mass Function

If $\alpha \in \mathbb{R}$, then for $c \in \{0, 1\}$,

$$\text{BernoulliLogit}(c|\alpha) = \text{Bernoulli}(c|\text{logit}^{-1}(\alpha)) = \begin{cases} \text{logit}^{-1}(\alpha) & \text{if } y = 1, \text{ and} \\ 1 - \text{logit}^{-1}(\alpha) & \text{if } y = 0. \end{cases}$$

Sampling Statement

`y ~ ` **`bernoulli_logit`**`(alpha);`

Increment log probability with `bernoulli_logit_log(y,alpha)`, dropping constant additive terms; Section 24.3 explains sampling statements.

Stan Functions

`real ` **`bernoulli_logit_log`**`(ints y, reals alpha)`

The log Bernoulli probability mass of *y* given chance of success `inv_logit(alpha)`

36. Bounded Discrete Distributions

Bounded discrete probability functions have support on $\{0, \ldots, N\}$ for some upper bound N.

36.1. Binomial Distribution

Probability Mass Function

Suppose $N \in \mathbb{N}$ and $\theta \in [0,1]$, and $n \in \{0, \ldots, N\}$.

$$\text{Binomial}(n|N,\theta) = \binom{N}{n} \theta^n (1-\theta)^{N-n}.$$

Log Probability Mass Function

$$\begin{aligned} \log \text{Binomial}(n|N,\theta) &= \log \Gamma(N+1) - \log \Gamma(n+1) - \log \Gamma(N-n+1) \\ &\quad + n \log \theta + (N-n) \log(1-\theta), \end{aligned}$$

Gradient of Log Probability Mass Function

$$\frac{\partial}{\partial \theta} \log \text{Binomial}(n|N,\theta) = \frac{n}{\theta} - \frac{N-n}{1-\theta}$$

Sampling Statement

`n ~ binomial(N,theta);`

Increment log probability with `binomial_log(n,N,theta)`, dropping constant additive terms; Section 24.3 explains sampling statements.

Stan Functions

`real binomial_log(ints n, ints N, reals theta)`

The log binomial probability mass of *n* successes in *N* trials given chance of success *theta*

`real binomial_cdf(ints n, ints N, reals theta)`

The binomial cumulative distribution function of *n* successes in *N* trials given chance of success *theta*

`real` **`binomial_cdf_log`**`(ints` *`n`*`, ints` *`N`*`, reals` *`theta`*`)`

The log of the binomial cumulative distribution function of n successes in N trials given chance of success *theta*

`real` **`binomial_ccdf_log`**`(ints` *`n`*`, ints` *`N`*`, reals` *`theta`*`)`

The log of the binomial complementary cumulative distribution function of n successes in N trials given chance of success *theta*

`int` **`binomial_rng`**`(int` *`N`*`, real` *`theta`*`)`

Generate a binomial variate with N trials and chance of success *theta*; may only be used in generated quantities block

36.2. Binomial Distribution, Logit Parameterization

Stan also provides a version of the binomial probability mass function distribution with the chance of success parameterized on the unconstrained logistic scale.

Probability Mass Function

Suppose $N \in \mathbb{N}$, $\alpha \in \mathbb{R}$, and $n \in \{0, \ldots, N\}$.

$$\begin{aligned} \mathsf{BinomialLogit}(n|N,\alpha) &= \mathsf{BinomialLogit}(n|N,\operatorname{logit}^{-1}(\alpha)) \\ &= \binom{N}{n} \left(\operatorname{logit}^{-1}(\alpha)\right)^{n} \left(1 - \operatorname{logit}^{-1}(\alpha)\right)^{N-n}. \end{aligned}$$

Log Probability Mass Function

$$\begin{aligned} \log \mathsf{BinomialLogit}(n|N,\alpha) &= \log\Gamma(N+1) - \log\Gamma(n+1) - \log\Gamma(N-n+1) \\ &\quad + n \log \operatorname{logit}^{-1}(\alpha) + (N-n)\log\left(1 - \operatorname{logit}^{-1}(\alpha)\right), \end{aligned}$$

Gradient of Log Probability Mass Function

$$\frac{\partial}{\partial\alpha} \log \mathsf{BinomialLogit}(n|N,\alpha) = \frac{n}{\operatorname{logit}^{-1}(-\alpha)} - \frac{N-n}{\operatorname{logit}^{-1}(\alpha)}$$

Sampling Statement

`n ~ binomial_logit(N,alpha);`
Increment log probability with `binomial_logit_log(n,N,alpha)`, dropping constant additive terms; Section 24.3 explains sampling statements.

Stan Functions

`real binomial_logit_log(ints n, ints N, reals alpha)`
The log binomial probability mass of *n* successes in *N* trials given logit-scaled chance of success *alpha*

36.3. Beta-Binomial Distribution

Probability Mass Function

If $N \in \mathbb{N}$, $\alpha \in \mathbb{R}^+$, and $\beta \in \mathbb{R}^+$, then for $n \in \{0, \ldots, N\}$,

$$\text{BetaBinomial}(n|N, \alpha, \beta) = \binom{N}{n} \frac{\mathrm{B}(n + \alpha, N - n + \beta)}{\mathrm{B}(\alpha, \beta)},$$

where the beta function $\mathrm{B}(u, v)$ is defined for $u \in \mathbb{R}^+$ and $v \in \mathbb{R}^+$ by

$$\mathrm{B}(u, v) = \frac{\Gamma(u)\,\Gamma(v)}{\Gamma(u + v)}.$$

Sampling Statement

`n ~ beta_binomial(N,alpha,beta);`
Increment log probability with `beta_binomial_log(n,N,alpha,beta)`, dropping constant additive terms; Section 24.3 explains sampling statements.

Stan Functions

`real beta_binomial_log(ints n, ints N, reals alpha, reals beta)`
The log beta-binomial probability mass of *n* successes in *N* trials given prior success count (plus one) of *alpha* and prior failure count (plus one) of *beta*

`real beta_binomial_cdf(ints n, ints N, reals alpha, reals beta)`
The beta-binomial cumulative distribution function of *n* successes in *N* trials given prior success count (plus one) of *alpha* and prior failure count (plus one) of *beta*

`real` **`beta_binomial_cdf_log`**`(ints n, ints N, reals alpha, reals beta)`

The log of the beta-binomial cumulative distribution function of *n* successes in *N* trials given prior success count (plus one) of *alpha* and prior failure count (plus one) of *beta*

`real` **`beta_binomial_ccdf_log`**`(ints n, ints N, reals alpha, reals beta)`

The log of the beta-binomial complementary cumulative distribution function of *n* successes in *N* trials given prior success count (plus one) of *alpha* and prior failure count (plus one) of *beta*

`int` **`beta_binomial_rng`**`(int N, real alpha, real beta)`

Generate a beta-binomial variate with *N* trials, prior success count (plus one) of *alpha*, and prior failure count (plus one) of *beta*; may only be used in generated quantities block

36.4. Hypergeometric Distribution

Probability Mass Function

If $a \in \mathbb{N}$, $b \in \mathbb{N}$, and $N \in \{0, \ldots, a+b\}$, then for $n \in \{\max(0, N-b), \ldots, \min(a, N)\}$,

$$\text{Hypergeometric}(n|N, a, b) = \frac{\binom{a}{n}\binom{b}{N-n}}{\binom{a+b}{N}}.$$

Sampling Statement

`n ~` **`hypergeometric`**`(N,a,b);`

Increment log probability with `hypergeometric_log(n,N,a,b)`, dropping constant additive terms; Section 24.3 explains sampling statements.

Stan Functions

`real` **`hypergeometric_log`**`(int n, int N, int a, int b)`

The log hypergeometric probability mass of *n* successes in *N* trials given total success count of *a* and total failure count of *b*

`int` **`hypergeometric_rng`**`(int N, real a, real b)`

Generate a hypergeometric variate with *N* trials, total success count of *a*, and total failure count of *b*; may only be used in generated quantities block

36.5. Categorical Distribution

Probability Mass Functions

If $N \in \mathbb{N}$, $N > 0$, and if $\theta \in \mathbb{R}^N$ forms an N-simplex (i.e., has nonnegative entries summing to one), then for $y \in \{1, \ldots, N\}$,

$$\text{Categorical}(y|\theta) = \theta_y.$$

In addition, Stan provides a log-odds scaled categorical distribution,

$$\text{CategoricalLogit}(y|\beta) = \text{Categorical}(y|\text{softmax}(\beta)).$$

See Section 33.6 for the definition of the softmax function.

Sampling Statement

`y ~ categorical(theta);`

Increment log probability with `categorical_log(y,theta)`, dropping constant additive terms; Section 24.3 explains sampling statements.

Sampling Statement

`y ~ categorical_logit(beta);`

Increment log probability with `categorical_logit_log(y,beta)`, dropping constant additive terms; Section 24.3 explains sampling statements.

Stan Functions

All of the categorical distributions are vectorized so that the outcome *y* can be a single integer (type `int`) or an array of integers (type `int[]`).

`real` **`categorical_log`**`(ints y, vector theta)`

The log categorical probability mass function with outcome(s) *y* in $1 : N$ given N-vector of outcome probabilities *theta*. The parameter *theta* must have non-negative entries that sum to one, but it need not be a variable declared as a simplex.

`real` **`categorical_logit_log`**`(ints y, vector beta)`

The log categorical probability mass function with outcome(s) *y* in $1 : N$ given log-odds of outcomes *beta*.

`int` **`categorical_rng`**`(vector theta)`

Generate a categorical variate with N-simplex distribution parameter *theta*; may only be used in generated quantities block

36.6. Ordered Logistic Distribution

Probability Mass Function

If $K \in \mathbb{N}$ with $K > 2$, $c \in \mathbb{R}^{K-1}$ such that $c_k < c_{k+1}$ for $k \in \{1, \ldots, K-2\}$, and $\eta \in \mathbb{R}$, then for $k \in \{1, \ldots, K\}$,

$$\mathsf{OrderedLogistic}(k|\eta, c) = \begin{cases} 1 - \mathrm{logit}^{-1}(\eta - c_1) & \text{if } k = 1, \\ \mathrm{logit}^{-1}(\eta - c_{k-1}) - \mathrm{logit}^{-1}(\eta - c_k) & \text{if } 1 < k < K, \text{and} \\ \mathrm{logit}^{-1}(\eta - c_{K-1}) - 0 & \text{if } k = K. \end{cases}$$

The $k = 1$ and $k = K$ edge cases can be subsumed into the general definition by setting $c_0 = -\infty$ and $c_K = +\infty$ with $\mathrm{logit}^{-1}(-\infty) = 0$ and $\mathrm{logit}^{-1}(\infty) = 1$.

Sampling Statement

`k ~ ordered_logistic(eta,c);`

Increment log probability with `ordered_logistic_log(k,eta,c)`, dropping constant additive terms; Section 24.3 explains sampling statements.

Stan Functions

`real ordered_logistic_log(int k, real eta, vector c)`

The log ordered logistic probability mass of `k` given linear predictor `eta` and cutpoints `c`.

`int ordered_logistic_rng(real eta, vector c)`

Generate an ordered logistic variate with linear predictor `eta` and cutpoints `c`; may only be used in generated quantities block

37. Unbounded Discrete Distributions

The unbounded discrete distributions have support over the natural numbers (i.e., the non-negative integers).

37.1. Negative Binomial Distribution

For the negative binomial distribution Stan uses the parameterization described in Gelman et al. (2013). For an alternative parametrization, see section 37.2

Probability Mass Function

If $\alpha \in \mathbb{R}^+$ and $\beta \in \mathbb{R}^+$, then for $y \in \mathbb{N}$,

$$\text{NegativeBinomial}(y|\alpha,\beta) = \binom{y+\alpha-1}{\alpha-1} \left(\frac{\beta}{\beta+1}\right)^{\alpha} \left(\frac{1}{\beta+1}\right)^{y}.$$

The mean and variance of a random variable $y \sim \text{NegativeBinomial}(\alpha,\beta)$ are given by

$$\mathbb{E}[y] = \frac{\alpha}{\beta} \quad \text{and} \quad \text{Var}[Y] = \frac{\alpha}{\beta^2}(\beta+1).$$

Sampling Statement

`n ~ neg_binomial(alpha,beta);`

Increment log probability with `neg_binomial_log(n,alpha,beta)`, dropping constant additive terms; Section 24.3 explains sampling statements.

Stan Functions

`real neg_binomial_log(ints n, reals alpha, reals beta)`

The log negative binomial probability mass of *n* given shape *alpha* and inverse scale *beta*

`real neg_binomial_cdf(ints n, reals alpha, reals beta)`

The negative binomial cumulative distribution function of *n* given shape *alpha* and inverse scale *beta*

`real neg_binomial_cdf_log(ints n, reals alpha, reals beta)`

The log of the negative binomial cumulative distribution function of *n* given shape *alpha* and inverse scale *beta*

`real` **`neg_binomial_ccdf_log`**`(ints n, reals alpha, reals beta)`
The log of the negative binomial complementary cumulative distribution function of *n* given shape *alpha* and inverse scale *beta*

`int` **`neg_binomial_rng`**`(real alpha, real beta)`
Generate a negative binomial variate with shape *alpha* and inverse scale *beta*; may only be used in generated quantities block

37.2. Negative Binomial Distribution, alternative parameterization

Stan also provides an alternative parameterization of the negative binomial distribution using the mean and inverse scale (overdispersion) as parameters.

Probability Mass Function

If $\eta \in \mathbb{R}$, $\mu \in \mathbb{R}^+$ and $\phi \in \mathbb{R}^+$, then for $y \in \mathbb{N}$,

$$\text{NegativeBinomial2}(y|\mu,\phi) = \binom{y+\phi-1}{y} \left(\frac{\mu}{\mu+\phi}\right)^{y} \left(\frac{\phi}{\mu+\phi}\right)^{\phi}.$$

The mean and variance of a random variable $y \sim \text{NegativeBinomial2}(y|\mu,\phi)$ are

$$\mathbb{E}[Y] = \mu = e^{\eta} \quad \text{and} \quad \text{Var}[Y] = \mu + \frac{\mu^2}{\phi}.$$

The parameter ϕ represents precision, whereas its inverse, ϕ^{-1}, represents overdispersion.

Sampling Statement

`y ~` **`neg_binomial_2`**`(mu, phi);`
Increment log probability with `neg_binomial_2_log(y,mu, phi)`, dropping constant additive terms; Section 24.3 explains sampling statements.

Sampling Statement

`y ~` **`neg_binomial_2_log`**`(eta, phi);`
Increment log probability with `neg_binomial_2_log_log(y,eta, phi)`, dropping constant additive terms; Section 24.3 explains sampling statements.

Stan Functions

`real` **`neg_binomial_2_log`**`(ints y, reals mu, reals phi)`

The negative binomial probability mass of *n* given location *mu* and precision *phi*.

`real` **`neg_binomial_2_log_log`**`(ints y, reals eta, reals phi)`

The log negative binomial probability mass of *n* given log-location *eta* and precision *phi*. This is useful for log-linear negative binomial regressions.

`int` **`neg_binomial_2_rng`**`(real mu, real phi)`

Generate a negative binomial variate with location *mu* and precision *phi*; may only be used in generated quantities block

`int` **`neg_binomial_2_log_rng`**`(real eta, real phi)`

Generate a negative binomial variate with log-location *eta* and precision *phi*; may only be used in generated quantities block

37.3. Poisson Distribution

Probability Mass Function

If $\lambda \in \mathbb{R}^+$, then for $n \in \mathbb{N}$,

$$\text{Poisson}(n|\lambda) = \frac{1}{n!}\,\lambda^n \exp(-\lambda).$$

Sampling Statement

`n ~` **`poisson`**`(lambda);`

Increment log probability with `poisson_log(n,lambda)`, dropping constant additive terms; Section 24.3 explains sampling statements.

Stan Functions

`real` **`poisson_log`**`(ints n, reals lambda)`

The log Poisson probability mass of *n* given rate *lambda*

`real` **`poisson_cdf`**`(ints n, reals lambda)`

The Poisson cumulative distribution function of *n* given rate *lambda*

`real` **`poisson_cdf_log`**`(ints n, reals lambda)`

The log of the Poisson cumulative distribution function of *n* given rate *lambda*

`real` **`poisson_ccdf_log`**`(ints` *`n`*`, reals` *`lambda`*`)`

The log of the Poisson complementary cumulative distribution function of *n* given rate *lambda*

`int` **`poisson_rng`**`(real` *`lambda`*`)`

Generate a Poisson variate with rate *lambda*; may only be used in generated quantities block

37.4. Poisson Distribution, Log Parameterization

Stan also provides a parameterization of the Poisson using the log rate $\alpha = \log \lambda$ as a parameter. This is useful for log-linear Poisson regressions so that the predictor does not need to be exponentiated and passed into the standard Poisson probability function.

Probability Mass Function

If $\alpha \in \mathbb{R}$, then for $n \in \mathbb{N}$,

$$\text{PoissonLog}(n|\alpha) = \frac{1}{n!} \exp\left(n\alpha - \exp(\alpha)\right).$$

Sampling Statement

`n` `~` **`poisson_log`**`(`*`alpha`*`);`

Increment log probability with `poisson_log_log(`*`n`*`,`*`alpha`*`)`, dropping constant additive terms; Section 24.3 explains sampling statements.

Stan Functions

`real` **`poisson_log_log`**`(ints` *`n`*`, reals` *`alpha`*`)`

The log Poisson probability mass of *n* given log rate *alpha*

38. Multivariate Discrete Distributions

The multivariate discrete distributions are over multiple integer values, which are expressed in Stan as arrays.

38.1. Multinomial Distribution

Probability Mass Function

If $K \in \mathbb{N}$, $N \in \mathbb{N}$, and $\theta \in K$-simplex, then for $y \in \mathbb{N}^K$ such that $\sum_{k=1}^{K} y_k = N$,

$$\text{Multinomial}(y|\theta) = \binom{N}{y_1, \ldots, y_K} \prod_{k=1}^{K} \theta_k^{y_k},$$

where the multinomial coefficient is defined by

$$\binom{N}{y_1, \ldots, y_k} = \frac{N!}{\prod_{k=1}^{K} y_k!}.$$

Sampling Statement

`y ~ multinomial(theta);`

Increment log probability with `multinomial_log(y,theta)`, dropping constant additive terms; Section 24.3 explains sampling statements.

Stan Functions

`real multinomial_log(int[] y, vector theta)`

The log multinomial probability mass function with outcome array `y` of size K given the K-simplex distribution parameter *theta* and (implicit) total count `N = sum(y)`

`vector multinomial_rng(vector theta, int N)`

Generate a multinomial variate with simplex distribution parameter *theta* and total count *N*; may only be used in generated quantities block

Part VI

Continuous Distributions

39. Unbounded Continuous Distributions

The unbounded univariate continuous probability distributions have support on all real numbers.

39.1. Normal Distribution

Probability Density Function

If $\mu \in \mathbb{R}$ and $\sigma \in \mathbb{R}^+$, then for $y \in \mathbb{R}$,

$$\text{Normal}(y|\mu,\sigma) = \frac{1}{\sqrt{2\pi}\,\sigma} \exp\left(-\frac{1}{2}\left(\frac{y-\mu}{\sigma}\right)^2\right).$$

Sampling Statement

`y ~ normal(mu,sigma);`

Increment log probability with `normal_log(y,mu,sigma)`, dropping constant additive terms; Section 24.3 explains sampling statements.

Stan Functions

`real` **`normal_log`**`(reals y, reals mu, reals sigma)`

The log of the normal density of *y* given location `mu` and scale `sigma`

`real` **`normal_cdf`**`(reals y, reals mu, reals sigma)`

The cumulative normal distribution of *y* given location `mu` and scale `sigma`; normal_cdf will underflow to 0 for $\frac{y-\mu}{\sigma}$ below -37.5 and overflow to 1 for $\frac{y-\mu}{\sigma}$ above 8.25

`real` **`normal_cdf_log`**`(reals y, reals mu, reals sigma)`

The log of the cumulative normal distribution of *y* given location `mu` and scale `sigma`; normal_cdf_log will underflow to $-\infty$ for $\frac{y-\mu}{\sigma}$ below -37.5 and overflow to 0 for $\frac{y-\mu}{\sigma}$ above 8.25

`real` **`normal_ccdf_log`**`(reals y, reals mu, reals sigma)`

The log of the complementary cumulative normal distribution of *y* given location `mu` and scale `sigma`; normal_ccdf_log will overflow to 0 for $\frac{y-\mu}{\sigma}$ below -37.5 and underflow to $-\infty$ for $\frac{y-\mu}{\sigma}$ above 8.25

`real` **`normal_rng`**`(real mu, real sigma)`

Generate a normal variate with location *mu* and scale *sigma*; may only be used in generated quantities block

39.2. Exponentially Modified Normal Distribution

Probability Density Function

If $\mu \in \mathbb{R}$, $\sigma \in \mathbb{R}^+$, and $\lambda \in \mathbb{R}^+$, then for $y \in \mathbb{R}$,

$$\text{ExpModNormal}(y|\mu,\sigma,\lambda) = \frac{\lambda}{\sqrt{\pi}}\,\exp\left(\frac{\lambda}{2}\left(2\mu + \lambda\sigma^2 - 2y\right)\right)\text{erfc}\left(\frac{\mu + \lambda\sigma^2 - y}{\sqrt{2}\sigma}\right).$$

Sampling Statement

`y ~` **`exp_mod_normal`**`(mu,sigma,lambda);`

Increment log probability with `exp_mod_normal_log(y,mu,sigma,lambda)`, dropping constant additive terms; Section 24.3 explains sampling statements.

Stan Functions

`real` **`exp_mod_normal_log`**`(reals y, reals mu, reals sigma, reals lambda)`

The log of the exponentially modified normal density of *y* given location *mu*, scale *sigma*, and shape *lambda*

`real` **`exp_mod_normal_cdf`**`(reals y, reals mu, reals sigma, reals lambda)`

The exponentially modified normal cumulative distribution function of *y* given location *mu*, scale *sigma*, and shape *lambda*

`real` **`exp_mod_normal_cdf_log`**`(reals y, reals mu, reals sigma, reals lambda)`

The log of the exponentially modified normal cumulative distribution function of *y* given location *mu*, scale *sigma*, and shape *lambda*

`real` **`exp_mod_normal_ccdf_log`**`(reals y, reals mu, reals sigma, reals lambda)`

The log of the exponentially modified normal complementary cumulative distribution function of *y* given location *mu*, scale *sigma*, and shape *lambda*

`real` **`exp_mod_normal_rng`**`(real mu, real sigma, real lambda)`

Generate a exponentially modified normal variate with location *mu*, scale *sigma*, and shape *lambda*; may only be used in generated quantities block

39.3. Skew Normal Distribution

Probability Density Function

If $\mu \in \mathbb{R}$, $\sigma \in \mathbb{R}^+$, and $\alpha \in \mathbb{R}$, then for $y \in \mathbb{R}$,

$$\text{SkewNormal}(y|\mu,\sigma,\alpha) = \frac{1}{\sigma\sqrt{2\pi}} \exp\left(-\frac{1}{2}\left(\frac{y-\mu}{\sigma}\right)^2\right) \left(1 + \text{erf}\left(\alpha\left(\frac{y-\mu}{\sigma\sqrt{2}}\right)\right)\right).$$

Sampling Statement

`y ~ skew_normal(mu,sigma,alpha);`

Increment log probability with `skew_normal_log(y,mu,sigma,alpha)`, dropping constant additive terms; Section 24.3 explains sampling statements.

Stan Functions

`real skew_normal_log(reals y, reals mu, reals sigma, reals alpha)`

The log of the skew normal density of *y* given location *mu*, scale *sigma*, and shape *alpha*

`real skew_normal_cdf(reals y, reals mu, reals sigma, reals alpha)`

The skew normal distribution function of *y* given location *mu*, scale *sigma*, and shape *alpha*

`real skew_normal_cdf_log(reals y, reals mu, reals sigma, reals alpha)`

The log of the skew normal cumulative distribution function of *y* given location *mu*, scale *sigma*, and shape *alpha*

`real skew_normal_ccdf_log(reals y, reals mu, reals sigma, reals alpha)`

The log of the skew normal complementary cumulative distribution function of *y* given location *mu*, scale *sigma*, and shape *alpha*

`real skew_normal_rng(real mu, real sigma, real alpha)`

Generate a skew normal variate with location *mu*, scale *sigma*, and shape *alpha*; may only be used in generated quantities block

39.4. Student-t Distribution

Probability Density Function

If $\nu \in \mathbb{R}^+$, $\mu \in \mathbb{R}$, and $\sigma \in \mathbb{R}^+$, then for $y \in \mathbb{R}$,

$$\text{StudentT}(y|\nu,\mu,\sigma) = \frac{\Gamma\left((\nu+1)/2\right)}{\Gamma(\nu/2)} \frac{1}{\sqrt{\nu\pi}\,\sigma} \left(1 + \frac{1}{\nu}\left(\frac{y-\mu}{\sigma}\right)^2\right)^{-(\nu+1)/2}.$$

Sampling Statement

`y ~ student_t(nu,mu,sigma);`

Increment log probability with `student_t_log(y,nu,mu,sigma)`, dropping constant additive terms; Section 24.3 explains sampling statements.

Stan Functions

`real student_t_log(reals y, reals nu, reals mu, reals sigma)`

The log of the Student-t density of *y* given degrees of freedom *nu*, location *mu*, and scale *sigma*

`real student_t_cdf(reals y, reals nu, reals mu, reals sigma)`

The Student-t cumulative distribution function of *y* given degrees of freedom *nu*, location *mu*, and scale *sigma*

`real student_t_cdf_log(reals y, reals nu, reals mu, reals sigma)`

The log of the Student-t cumulative distribution function of *y* given degrees of freedom *nu*, location *mu*, and scale *sigma*

`real student_t_ccdf_log(reals y, reals nu, reals mu, reals sigma)`

The log of the Student-t complementary cumulative distribution function of *y* given degrees of freedom *nu*, location *mu*, and scale *sigma*

`real student_t_rng(real nu, real mu, real sigma)`

Generate a Student-t variate with degrees of freedom *nu*, location *mu*, and scale *sigma*; may only be used in generated quantities block

39.5. Cauchy Distribution

Probability Density Function

If $\mu \in \mathbb{R}$ and $\sigma \in \mathbb{R}^+$, then for $y \in \mathbb{R}$,

$$\text{Cauchy}(y|\mu,\sigma) = \frac{1}{\pi\sigma} \, \frac{1}{1 + ((y-\mu)/\sigma)^2}.$$

Sampling Statement

`y ~ cauchy(mu,sigma);`

Increment log probability with `cauchy_log(y,mu,sigma)`, dropping constant additive terms; Section 24.3 explains sampling statements.

Stan Functions

`real cauchy_log(reals y, reals mu, reals sigma)`

The log of the Cauchy density of *y* given location *mu* and scale *sigma*

`real cauchy_cdf(reals y, reals mu, reals sigma)`

The Cauchy cumulative distribution function of *y* given location *mu* and scale *sigma*

`real cauchy_cdf_log(reals y, reals mu, reals sigma)`

The log of the Cauchy cumulative distribution function of *y* given location *mu* and scale *sigma*

`real cauchy_ccdf_log(reals y, reals mu, reals sigma)`

The log of the Cauchy complementary cumulative distribution function of *y* given location *mu* and scale *sigma*

`real cauchy_rng(real mu, real sigma)`

Generate a Cauchy variate with location *mu* and scale *sigma*; may only be used in generated quantities block

39.6. Double Exponential (Laplace) Distribution

Probability Density Function

If $\mu \in \mathbb{R}$ and $\sigma \in \mathbb{R}^+$, then for $y \in \mathbb{R}$,

$$\text{DoubleExponential}(y|\mu,\sigma) = \frac{1}{2\sigma} \exp\left(-\frac{|y-\mu|}{\sigma}\right).$$

Sampling Statement

`y ~ double_exponential(mu,sigma);`

Increment log probability with `double_exponential_log(y,mu,sigma)`, dropping constant additive terms; Section 24.3 explains sampling statements.

Stan Functions

`real` **`double_exponential_log`**`(reals y, reals mu, reals sigma)`

The log of the double exponential density of *y* given location *mu* and scale *sigma*

`real` **`double_exponential_cdf`**`(reals y, reals mu, reals sigma)`

The double exponential cumulative distribution function of *y* given location *mu* and scale *sigma*

`real` **`double_exponential_cdf_log`**`(reals y, reals mu, reals sigma)`

The log of the double exponential cumulative distribution function of *y* given location *mu* and scale *sigma*

`real` **`double_exponential_ccdf_log`**`(reals y, reals mu, reals sigma)`

The log of the double exponential complementary cumulative distribution function of *y* given location *mu* and scale *sigma*

`real` **`double_exponential_rng`**`(real mu, real sigma)`

Generate a double exponential variate with location *mu* and scale *sigma*; may only be used in generated quantities block

39.7. Logistic Distribution

Probability Density Function

If $\mu \in \mathbb{R}$ and $\sigma \in \mathbb{R}^+$, then for $y \in \mathbb{R}$,

$$\text{Logistic}(y|\mu,\sigma) = \frac{1}{\sigma}\,\exp\!\left(-\,\frac{y-\mu}{\sigma}\right)\,\left(1+\exp\!\left(-\,\frac{y-\mu}{\sigma}\right)\right)^{-2}.$$

Sampling Statement

`y ~ logistic(mu,sigma);`

Increment log probability with `logistic_log(y,mu,sigma)`, dropping constant additive terms; Section 24.3 explains sampling statements.

Stan Functions

real **logistic_log**(reals *y*, reals *mu*, reals *sigma*)
The log of the logistic density of *y* given location *mu* and scale *sigma*

real **logistic_cdf**(reals *y*, reals *mu*, reals *sigma*)
The logistic cumulative distribution function of *y* given location *mu* and scale *sigma*

real **logistic_cdf_log**(reals *y*, reals *mu*, reals *sigma*)
The log of the logistic cumulative distribution function of *y* given location *mu* and scale *sigma*

real **logistic_ccdf_log**(reals *y*, reals *mu*, reals *sigma*)
The log of the logistic complementary cumulative distribution function of *y* given location *mu* and scale *sigma*

real **logistic_rng**(real *mu*, real *sigma*)
Generate a logistic variate with location *mu* and scale *sigma*; may only be used in generated quantities block

39.8. Gumbel Distribution

Probability Density Function

If $\mu \in \mathbb{R}$ and $\beta \in \mathbb{R}^+$, then for $y \in \mathbb{R}$,

$$\text{Gumbel}(y|\mu,\beta) = \frac{1}{\beta}\,\exp\left(-\frac{y-\mu}{\beta} - \exp\left(-\frac{y-\mu}{\beta}\right)\right).$$

Sampling Statement

y ~ **gumbel**(*mu*,*beta*);
Increment log probability with gumbel_log(*y*,*mu*,*beta*), dropping constant additive terms; Section 24.3 explains sampling statements.

Stan Functions

real **gumbel_log**(reals *y*, reals *mu*, reals *beta*)
The log of the gumbel density of *y* given location *mu* and scale *beta*

`real` **`gumbel_cdf`**`(reals` *`y`*`, reals` *`mu`*`, reals` *`beta`*`)`

The gumbel cumulative distribution function of *y* given location *mu* and scale *beta*

`real` **`gumbel_cdf_log`**`(reals` *`y`*`, reals` *`mu`*`, reals` *`beta`*`)`

The log of the gumbel cumulative distribution function of *y* given location *mu* and scale *beta*

`real` **`gumbel_ccdf_log`**`(reals` *`y`*`, reals` *`mu`*`, reals` *`beta`*`)`

The log of the gumbel complementary cumulative distribution function of *y* given location *mu* and scale *beta*

`real` **`gumbel_rng`**`(real` *`mu`*`, real` *`beta`*`)`

Generate a gumbel variate with location *mu* and scale *beta*; may only be used in generated quantities block

40. Positive Continuous Distributions

The positive continuous probability functions have support on the positive real numbers.

40.1. Lognormal Distribution

Probability Density Function

If $\mu \in \mathbb{R}$ and $\sigma \in \mathbb{R}^+$, then for $y \in \mathbb{R}^+$,

$$\text{LogNormal}(y|\mu,\sigma) = \frac{1}{\sqrt{2\pi}\ \sigma}\ \frac{1}{y}\ \exp\left(-\frac{1}{2}\left(\frac{\log y - \mu}{\sigma}\right)^2\right).$$

Sampling Statement

`y ~ lognormal(mu,sigma);`

Increment log probability with `lognormal_log(y,mu,sigma)`, dropping constant additive terms; Section 24.3 explains sampling statements.

Stan Functions

`real lognormal_log(reals y, reals mu, reals sigma)`

The log of the lognormal density of *y* given location *mu* and scale *sigma*

`real lognormal_cdf(reals y, reals mu, reals sigma)`

The cumulative lognormal distribution function of *y* given location *mu* and scale *sigma*

`real lognormal_cdf_log(reals y, reals mu, reals sigma)`

The log of the lognormal cumulative distribution fucntion of *y* given location *mu* and scale *sigma*

`real lognormal_ccdf_log(reals y, reals mu, reals sigma)`

The log of the lognormal complementary cumulative distribution function of *y* given location *mu* and scale *sigma*

`real lognormal_rng(real mu, real beta)`

Generate a lognormal variate with location *mu* and scale *sigma*; may only be used in generated quantities block

40.2. Chi-Square Distribution

Probability Density Function

If $\nu \in \mathbb{R}^+$, then for $y \in \mathbb{R}^+$,

$$\text{ChiSquare}(y|\nu) = \frac{2^{-\nu/2}}{\Gamma(\nu/2)}\, y^{\nu/2-1} \exp\!\left(-\,\frac{1}{2}\, y\right).$$

Sampling Statement

`y ~ `**`chi_square`**`(nu);`

Increment log probability with `chi_square_log(y,nu)`, dropping constant additive terms; Section 24.3 explains sampling statements.

Stan Functions

`real `**`chi_square_log`**`(reals y, reals nu)`

The log of the Chi-square density of *y* given degrees of freedom *nu*

`real `**`chi_square_cdf`**`(reals y, reals nu)`

The Chi-square cumulative distribution function of *y* given degrees of freedom *nu*

`real `**`chi_square_cdf_log`**`(reals y, reals nu)`

The log of the Chi-square cumulative distribution function of *y* given degrees of freedom *nu*

`real `**`chi_square_ccdf_log`**`(reals y, reals nu)`

The log of the complementary Chi-square cumulative distribution function of *y* given degrees of freedom *nu*

`real `**`chi_square_rng`**`(real nu)`

Generate a Chi-square variate with degrees of freedom *nu*; may only be used in generated quantities block

40.3. Inverse Chi-Square Distribution

Probability Density Function

If $\nu \in \mathbb{R}^+$, then for $y \in \mathbb{R}^+$,

$$\text{InvChiSquare}(y|\nu) = \frac{2^{-\nu/2}}{\Gamma(\nu/2)}\, y^{-(\nu/2-1)} \exp\!\left(-\,\frac{1}{2}\,\frac{1}{y}\right).$$

Sampling Statement

`y ~ inv_chi_square(nu);`

Increment log probability with `inv_chi_square_log(y,nu)`, dropping constant additive terms; Section 24.3 explains sampling statements.

Stan Functions

`real inv_chi_square_log(reals y, reals nu)`

The log of the inverse Chi-square density of *y* given degrees of freedom *nu*

`real inv_chi_square_cdf(reals y, reals nu)`

The inverse Chi-squared cumulative distribution function of *y* given degrees of freedom *nu*

`real inv_chi_square_cdf_log(reals y, reals nu)`

The log of the inverse Chi-squared cumulative distribution function of *y* given degrees of freedom *nu*

`real inv_chi_square_ccdf_log(reals y, reals nu)`

The log of the inverse Chi-squared complementary cumulative distribution function of *y* given degrees of freedom *nu*

`real inv_chi_square_rng(real nu)`

Generate an inverse Chi-squared variate with degrees of freedom *nu*; may only be used in generated quantities block

40.4. Scaled Inverse Chi-Square Distribution

Probability Density Function

If $\nu \in \mathbb{R}^+$ and $\sigma \in \mathbb{R}^+$, then for $y \in \mathbb{R}^+$,

$$\text{ScaledInvChiSquare}(y|\nu,\sigma) = \frac{(\nu/2)^{\nu/2}}{\Gamma(\nu/2)}\,\sigma^{\nu}\,y^{-(\nu/2+1)}\,\exp\!\left(-\frac{1}{2}\,\nu\,\sigma^2\,\frac{1}{y}\right).$$

Sampling Statement

`y ~ scaled_inv_chi_square(nu,sigma);`

Increment log probability with `scaled_inv_chi_square_log(y,nu,sigma)`, dropping constant additive terms; Section 24.3 explains sampling statements.

Stan Functions

`real` **`scaled_inv_chi_square_log`**`(reals y, reals nu, reals sigma)`

The log of the scaled inverse Chi-square density of *y* given degrees of freedom *nu* and scale *sigma*

`real` **`scaled_inv_chi_square_cdf`**`(reals y, reals nu, reals sigma)`

The scaled inverse Chi-square cumulative distribution function of *y* given degrees of freedom *nu* and scale *sigma*

`real` **`scaled_inv_chi_square_cdf_log`**`(reals y, reals nu, reals sigma)`

The log of the scaled inverse Chi-square cumulative distribution function of *y* given degrees of freedom *nu* and scale *sigma*

`real` **`scaled_inv_chi_square_ccdf_log`**`(reals y, reals nu, reals sigma)`

The log of the scaled inverse Chi-square complementary cumulative distribution function of *y* given degrees of freedom *nu* and scale *sigma*

`real` **`scaled_inv_chi_square_rng`**`(real nu, real sigma)`

Generate a scaled inverse Chi-squared variate with degrees of freedom *nu* and scale *sigma*; may only be used in generated quantities block

40.5. Exponential Distribution

Probability Density Function

If $\beta \in \mathbb{R}^+$, then for $y \in \mathbb{R}^+$,

$$\text{Exponential}(y|\beta) = \beta \exp(-\beta\, y).$$

Sampling Statement

`y ~` **`exponential`**`(beta);`

Increment log probability with `exponential_log(y,beta)`, dropping constant additive terms; Section 24.3 explains sampling statements.

Stan Functions

`real` **`exponential_log`**`(reals y, reals beta)`

The log of the exponential density of *y* given inverse scale *beta*

`real` **`exponential_cdf`**`(reals y, reals beta)`
The exponential cumulative distribution function of *y* given inverse scale *beta*

`real` **`exponential_cdf_log`**`(reals y, reals beta)`
The log of the exponential cumulative distribution function of *y* given inverse scale *beta*

`real` **`exponential_ccdf_log`**`(reals y, reals beta)`
The log of the exponential complementary cumulative distribution function of *y* given inverse scale *beta*

`real` **`exponential_rng`**`(real beta)`
Generate an exponential variate with inverse scale *beta*; may only be used in generated quantities block

40.6. Gamma Distribution

Probability Density Function

If $\alpha \in \mathbb{R}^+$ and $\beta \in \mathbb{R}^+$, then for $y \in \mathbb{R}^+$,

$$\text{Gamma}(y|\alpha,\beta) = \frac{\beta^\alpha}{\Gamma(\alpha)}\, y^{\alpha-1} \exp(-\beta\, y).$$

Sampling Statement

`y ~` **`gamma`**`(alpha,beta);`
Increment log probability with `gamma_log(y,alpha,beta)`, dropping constant additive terms; Section 24.3 explains sampling statements.

Stan Functions

`real` **`gamma_log`**`(reals y, reals alpha, reals beta)`
The log of the gamma density of *y* given shape *alpha* and inverse scale *beta*

`real` **`gamma_cdf`**`(reals y, reals alpha, reals beta)`
The cumulative gamma distribution function of *y* given shape *alpha* and inverse scale *beta*

`real` **`gamma_cdf_log`**`(reals y, reals alpha, reals beta)`
The log of the cumulative gamma distribution function of *y* given shape *alpha* and inverse scale *beta*

`real` **`gamma_ccdf_log`**`(reals y, reals alpha, reals beta)`

The log of the complementary cumulative gamma distribution function of *y* given shape *alpha* and inverse scale *beta*

`real` **`gamma_rng`**`(real alpha, real beta)`

Generate a gamma variate with shape *alpha* and inverse scale *beta*; may only be used in generated quantities block

40.7. Inverse Gamma Distribution

Probability Density Function

If $\alpha \in \mathbb{R}^+$ and $\beta \in \mathbb{R}^+$, then for $y \in \mathbb{R}^+$,

$$\text{InvGamma}(y|\alpha,\beta) = \frac{\beta^\alpha}{\Gamma(\alpha)}\, y^{-(\alpha+1)} \exp\left(-\beta\,\frac{1}{y}\right).$$

Sampling Statement

`y ~` **`inv_gamma`**`(alpha,beta);`

Increment log probability with `inv_gamma_log(y,alpha,beta)`, dropping constant additive terms; Section 24.3 explains sampling statements.

Stan Functions

`real` **`inv_gamma_log`**`(reals y, reals alpha, reals beta)`

The log of the inverse gamma density of *y* given shape *alpha* and scale *beta*

`real` **`inv_gamma_cdf`**`(reals y, reals alpha, reals beta)`

The inverse gamma cumulative distribution function of *y* given shape *alpha* and scale *beta*

`real` **`inv_gamma_cdf_log`**`(reals y, reals alpha, reals beta)`

The log of the inverse gamma cumulative distribution function of *y* given shape *alpha* and scale *beta*

`real` **`inv_gamma_ccdf_log`**`(reals y, reals alpha, reals beta)`

The log of the inverse gamma complementary cumulative distribution function of *y* given shape *alpha* and scale *beta*

`real` **`inv_gamma_rng`**`(real alpha, real beta)`

Generate an inverse gamma variate with shape *alpha* and scale *beta*; may only be used in generated quantities block

40.8. Weibull Distribution

Probability Density Function

If $\alpha \in \mathbb{R}^+$ and $\sigma \in \mathbb{R}^+$, then for $y \in [0, \infty)$,

$$\text{Weibull}(y|\alpha, \sigma) = \frac{\alpha}{\sigma} \left(\frac{y}{\sigma}\right)^{\alpha-1} \exp\left(-\left(\frac{y}{\sigma}\right)^{\alpha}\right).$$

Note that if $Y \propto \text{Weibull}(\alpha, \sigma)$, then $Y^{-1} \propto \text{Frechet}(\alpha, \sigma^{-1})$.

Sampling Statement

`y ~ weibull(alpha,sigma);`

Increment log probability with `weibull_log(y,alpha,sigma)`, dropping constant additive terms; Section 24.3 explains sampling statements.

Stan Functions

`real weibull_log(reals y, reals alpha, reals sigma)`

The log of the Weibull density of *y* given shape *alpha* and scale *sigma*

`real weibull_cdf(reals y, reals alpha, reals sigma)`

The Weibull cumulative distribution function of *y* given shape *alpha* and scale *sigma*

`real weibull_cdf_log(reals y, reals alpha, reals sigma)`

The log of the Weibull cumulative distribution function of *y* given shape *alpha* and scale *sigma*

`real weibull_ccdf_log(reals y, reals alpha, reals sigma)`

The log of the Weibull complementary cumulative distribution function of *y* given shape *alpha* and scale *sigma*

`real weibull_rng(real alpha, real sigma)`

Generate a weibull variate with shape *alpha* and scale *sigma*; may only be used in generated quantities block

40.9. Fréchet Distribution

Probability Density Function

If $\alpha \in \mathbb{R}^+$ and $\sigma \in \mathbb{R}^+$, then for $y \in \mathbb{R}^+$,

$$\text{Frechet}(y|\alpha,\sigma) = \frac{\alpha}{\sigma} \left(\frac{y}{\sigma}\right)^{-\alpha-1} \exp\left(-\left(\frac{y}{\sigma}\right)^{-\alpha}\right).$$

Note that if $Y \propto \text{Frechet}(\alpha, \sigma)$, then $Y^{-1} \propto \text{Weibull}(\alpha, \sigma^{-1})$.

Sampling Statement

`y ~ frechet(alpha,sigma);`

Increment log probability with `frechet_log(y,alpha,sigma)`, dropping constant additive terms; Section 24.3 explains sampling statements.

Stan Functions

`real frechet_log(reals y, reals alpha, reals sigma)`

The log of the Fréchet density of *y* given shape *alpha* and scale *sigma*

`real frechet_cdf(reals y, reals alpha, reals sigma)`

The Fréchet cumulative distribution function of *y* given shape *alpha* and scale *sigma*

`real frechet_cdf_log(reals y, reals alpha, reals sigma)`

The log of the Fréchet cumulative distribution function of *y* given shape *alpha* and scale *sigma*

`real frechet_ccdf_log(reals y, reals alpha, reals sigma)`

The log of the Fréchet complementary cumulative distribution function of *y* given shape *alpha* and scale *sigma*

`real frechet_rng(real alpha, real sigma)`

Generate an Fréchet variate with shape *alpha* and scale *sigma*; may only be used in generated quantities block

41. Non-negative Continuous Distributions

The non-negative continuous probability functions have support on the non-negative real numbers.

41.1. Rayleigh Distribution

Probability Density Function

If $\sigma \in \mathbb{R}^+$, then for $y \in [0, \infty)$,

$$\text{Rayleigh}(y|\sigma) = \frac{y}{\sigma^2} \exp(-y^2/2\sigma^2).$$

Sampling Statement

`y ~ rayleigh(sigma);`

Increment log probability with `rayleigh_log(y,sigma)`, dropping constant additive terms; Section 24.3 explains sampling statements.

Stan Functions

`real rayleigh_log(reals y, reals sigma)`

The log of the Rayleigh ensity of *y* given scale `sigma`

`real rayleigh_cdf(real y, real sigma)`

The Rayleigh cumulative distribution of *y* given scale `sigma`

`real rayleigh_cdf_log(real y, real sigma)`

The log of the Rayleigh cumulative distribution of *y* given scale `sigma`

`real rayleigh_ccdf_log(real y, real sigma)`

The log of the Rayleigh complementary cumulative distribution of *y* given scale `sigma`

`real rayleigh_rng(real sigma)`

Generate a Rayleigh variate with scale `sigma`; may only be used in generated quantities block

42. Positive Lower-Bounded Probabilities

The positive lower-bounded probabilities have support on real values above some positive minimum value.

42.1. Pareto Distribution

Probability Density Function

If $y_{\text{min}} \in \mathbb{R}^+$ and $\alpha \in \mathbb{R}^+$, then for $y \in \mathbb{R}^+$ with $y \geq y_{\text{min}}$,

$$\text{Pareto}(y|y_{\text{min}}, \alpha) = \frac{\alpha\, y_{\text{min}}^{\alpha}}{y^{\alpha+1}}.$$

Sampling Statement

`y ~ pareto(y_min,alpha);`

Increment log probability with `pareto_log(y,y_min,alpha)`, dropping constant additive terms; Section 24.3 explains sampling statements.

Stan Functions

`real pareto_log(reals y, reals y_min, reals alpha)`

The log of the Pareto density of `y` given positive minimum value `y_min` and shape `alpha`

`real pareto_cdf(reals y, reals y_min, reals alpha)`

The Pareto cumulative distribution function of `y` given positive minimum value `y_min` and shape `alpha`

`real pareto_cdf_log(reals y, reals y_min, reals alpha)`

The log of the Pareto cumulative distribution function of `y` given positive minimum value `y_min` and shape `alpha`

`real pareto_ccdf_log(reals y, reals y_min, reals alpha)`

The log of the Pareto complementary cumulative distribution function of `y` given positive minimum value `y_min` and shape `alpha`

`real pareto_rng(real y_min, real alpha)`

Generate a Pareto variate with positive minimum value `y_min` and shape `alpha`; may only be used in generated quantities block

42.2. Pareto Type 2 Distribution

Probability Density Function

If $\mu \in \mathbb{R}$, $\lambda \in \mathbb{R}^+$, and $\alpha \in \mathbb{R}^+$, then for $y \geq \mu$,

$$\text{Pareto\_Type\_2}(y|\mu, \lambda, \alpha) = \frac{\alpha}{\lambda} \left(1 + \frac{y - \mu}{\lambda}\right)^{-(\alpha+1)}.$$

Note that the Lomax distribution is a Pareto Type 2 distribution with $\mu = 0$.

Sampling Statement

`y ~ pareto_type_2(mu,lambda,alpha);`
Increment log probability with `pareto_type_2_log(y,mu,lambda,alpha)`, dropping constant additive terms; Section 24.3 explains sampling statements.

Stan Functions

`real pareto_type_2_log(reals y, reals mu, reals lambda, reals alpha)`
The log of the Pareto Type 2 density of *y* given location *mu*, scale *lambda*, and shape *alpha*

`real pareto_type_2_cdf(reals y, reals mu, reals lambda, reals alpha)`
The Pareto Type 2 cumulative distribution function of *y* given location *mu*, scale *lambda*, and shape *alpha*

`real pareto_type_2_cdf_log(reals y, reals mu, reals lambda, reals alpha)`
The log of the Pareto Type 2 cumulative distribution function of *y* given location *mu*, scale *lambda*, and shape *alpha*

`real pareto_type_2_ccdf_log(reals y, reals mu, reals lambda, reals alpha)`
The log of the Pareto Type 2 complementary cumulative distribution function of *y* given location *mu*, scale *lambda*, and shape *alpha*

`real pareto_type_2_rng(real mu, real lambda, real alpha)`
Generate a Pareto Type 2 variate with location *mu*, scale *lambda*, and shape *alpha*; may only be used in generated quantities block

43. Continuous Distributions on [0, 1]

The continuous distributions with outcomes in the interval $[0,1]$ are used to characterized bounded quantities, including probabilities.

43.1. Beta Distribution

Probability Density Function

If $\alpha \in \mathbb{R}^+$ and $\beta \in \mathbb{R}^+$, then for $\theta \in (0,1)$,

$$\text{Beta}(\theta|\alpha,\beta) = \frac{1}{\mathrm{B}(\alpha,\beta)}\, \theta^{\alpha-1}\,(1-\theta)^{\beta-1},$$

where the beta function $\mathrm{B}()$ is as defined in Section 31.12.

Warning: If $\theta = 0$ or $\theta = 1$, then the probability is 0 and the log probability is $-\infty$. Similarly, the distribution requires strictly positive parameters, $\alpha, \beta > 0$.

Sampling Statement

`theta ~ beta(alpha,beta);`

Increment log probability with `beta_log(theta,alpha,beta)`, dropping constant additive terms; Section 24.3 explains sampling statements.

Stan Functions

`real beta_log(reals theta, reals alpha, reals beta)`

The log of the beta density of `theta` in $[0,1]$ given positive prior successes (plus one) `alpha` and prior failures (plus one) `beta`

`real beta_cdf(reals theta, reals alpha, reals beta)`

The beta cumulative distribution function of `theta` in $[0,1]$ given positive prior successes (plus one) `alpha` and prior failures (plus one) `beta`

`real beta_cdf_log(reals theta, reals alpha, reals beta)`

The log of the beta cumulative distribution function of `theta` in $[0,1]$ given positive prior successes (plus one) `alpha` and prior failures (plus one) `beta`

`real beta_ccdf_log(reals theta, reals alpha, reals beta)`

The log of the beta complementary cumulative distribution function of `theta` in $[0,1]$ given positive prior successes (plus one) `alpha` and prior failures (plus one) `beta`

`real` **`beta_rng`**`(real` *`alpha`*`, real` *`beta`*`)`

Generate a beta variate with positive prior successes (plus one) *`alpha`* and prior failures (plus one) *`beta`*; may only be used in generated quantities block

44. Circular Distributions

Circular distributions are defined for finite values y in any interval of length 2π.

44.1. Von Mises Distribution

Probability Density Function

If $\mu \in \mathbb{R}$ and $\kappa \in \mathbb{R}^+$, then for $y \in \mathbb{R}$,

$$\text{VonMises}(y|\mu,\kappa) = \frac{\exp(\kappa\cos(y-\mu))}{2\pi I_0(\kappa)}.$$

In order for this density to properly normalize, y must be restricted to some interval $(c, c+2\pi)$ of length 2π, because

$$\int_c^{c+2\pi} \text{VonMises}(y|\mu,\kappa)dy = 1.$$

Similarly, if μ is a parameter, it will typically be restricted to the same range as y.

A von Mises distribution with its 2π interval of support centered around its location μ will have a single mode at μ; for example, restricting y to $(-\pi,\pi)$ and taking $\mu = 0$ leads to a single local optimum at the model μ. If the location μ is not in the center of the support, the density is circularly translated and there will be a second local maximum at the boundary furthest from the mode. Ideally, the parameterization and support will be set up so that the bulk of the probability mass is in a continuous interval around the mean μ.

Sampling Statement

`y ~ von_mises(mu,kappa);`

Increment log probability with `von_mises_log(y,mu,kappa)`, dropping constant additive terms; Section 24.3 explains sampling statements.

Stan Functions

`real von_mises_log(reals y, reals mu, reals kappa)`

The log of the von mises density of *y* given location *mu* and scale *kappa*

`real von_mises_rng(reals mu, reals kappa)`

Generate a Von Mises variate with location *mu* and scale *kappa* (i.e. returns values in the interval $[(\mu \mod 2\pi) - \pi, (\mu \mod 2\pi) + \pi]$); may only be used in generated quantities block

Numerical Stability

Evaluating the Von Mises distribution for $\kappa > 100$ is numerically unstable in the current implementation. Nathanael I. Lichti suggested the following workaround on the Stan users group, based on the fact that as $\kappa \rightarrow \infty$,

$$\text{VonMises}(y|\mu, \kappa) \rightarrow \text{Normal}(\mu, \sqrt{1/\kappa}).$$

The workaround is to replace `y ~ von_mises(mu,kappa)` with

```
if (kappa < 100)
  y ~ von_mises(mu, kappa);
else
  y ~ normal(mu, sqrt(1 / kappa));
```

45. Bounded Continuous Probabilities

The bounded continuous probabilities have support on a finite interval of real numbers.

45.1. Uniform Distribution

Probability Density Function

If $\alpha \in \mathbb{R}$ and $\beta \in (\alpha, \infty)$, then for $y \in [\alpha, \beta]$,

$$\mathsf{Uniform}(y|\alpha, \beta) = \frac{1}{\beta - \alpha}.$$

Sampling Statement

`y ~ uniform(alpha,beta);`

Increment log probability with `uniform_log(y,alpha,beta)`, dropping constant additive terms; Section 24.3 explains sampling statements.

Stan Functions

`real uniform_log(reals y, reals alpha, reals beta)`

The log of the uniform density of *y* given lower bound *alpha* and upper bound *beta*

`real uniform_cdf(reals y, reals alpha, reals beta)`

The uniform cumulative distribution function of *y* given lower bound *alpha* and upper bound *beta*

`real uniform_cdf_log(reals y, reals alpha, reals beta)`

The log of the uniform cumulative distribution function of *y* given lower bound *alpha* and upper bound *beta*

`real uniform_ccdf_log(reals y, reals alpha, reals beta)`

The log of the uniform complementary cumulative distribution function of *y* given lower bound *alpha* and upper bound *beta*

`real uniform_rng(real alpha, real beta)`

Generate a uniform variate with lower bound *alpha* and upper bound *beta*; may only be used in generated quantities block

46. Distributions over Unbounded Vectors

The unbounded vector probability distributions have support on all of $\mathbb{R}^K$ for some fixed K.

46.1. Multivariate Normal Distribution

Probability Density Function

If $K \in \mathbb{N}$, $\mu \in \mathbb{R}^K$, and $\Sigma \in \mathbb{R}^{K \times K}$ is symmetric and positive definite, then for $y \in \mathbb{R}^K$,

$$\text{MultiNormal}(y|\mu, \Sigma) = \frac{1}{(2\pi)^{K/2}} \frac{1}{\sqrt{|\Sigma|}} \exp\left(-\frac{1}{2}(y - \mu)^\top \Sigma^{-1} (y - \mu)\right),$$

where $|\Sigma|$ is the absolute determinant of Σ.

Sampling Statement

`y ~ multi_normal(mu,Sigma);`

Increment log probability with `multi_normal_log(y,mu,Sigma)`, dropping constant additive terms; Section 24.3 explains sampling statements.

Stan Functions

The multivariate normal probability function is overloaded to allow the variate vector y and location vector μ to be vectors or row vectors (or to mix the two types). The density function is also vectorized, so it allows arrays of row vectors or vectors as arguments; see Section 28.1 for a description of vectorization.

`real multi_normal_log(vectors y, vectors mu, matrix Sigma)`

The log of the multivariate normal density of vector(s) *y* given location vector(s) *mu* and covariance matrix *Sigma*

`real multi_normal_log(vectors y, row_vectors mu, matrix Sigma)`

The log of the multivariate normal density of vector(s) *y* given location row vector(s) *mu* and covariance matrix *Sigma*

`real multi_normal_log(row_vectors y, vectors mu, matrix Sigma)`

The log of the multivariate normal density of row vector(s) *y* given location vector(s) *mu* and covariance matrix *Sigma*

`real` **`multi_normal_log`**`(row_vectors` *`y`*`, row_vectors` *`mu`*`, matrix` *`Sigma`*`)`

The log of the multivariate normal density of row vector(s) *y* given location row vector(s) *mu* and covariance matrix *Sigma*

`vector` **`multi_normal_rng`**`(vector` *`mu`*`, matrix` *`Sigma`*`)`

Generate a multivariate normal variate with location *mu* and covariance matrix *Sigma*; may only be used in generated quantities block

46.2. Multivariate Normal Distribution, Precision Parameterization

Probability Density Function

If $K \in \mathbb{N}$, $\mu \in \mathbb{R}^K$, and $\Omega \in \mathbb{R}^{K \times K}$ is symmetric and positive definite, then for $y \in \mathbb{R}^K$,

$$\text{MultiNormalPrecision}(y|\mu, \Omega) = \text{MultiNormal}(y|\mu, \Sigma^{-1})$$

Sampling Statement

`y` `~` **`multi_normal_prec`**`(`*`mu,Omega`*`);`

Increment log probability with `multi_normal_prec_log(`*`y,mu,Omega`*`)`, dropping constant additive terms; Section 24.3 explains sampling statements.

Stan Functions

`real` **`multi_normal_prec_log`**`(vectors` *`y`*`, vectors` *`mu`*`, matrix` *`Omega`*`)`

The log of the multivariate normal density of vector(s) *y* given location vector(s) *mu* and positive definite precision matrix *Omega*

`real` **`multi_normal_prec_log`**`(vectors` *`y`*`, row_vectors` *`mu`*`, matrix` *`Omega`*`)`

The log of the multivariate normal density of vector(s) *y* given location row vector(s) *mu* and positive definite precision matrix *Omega*

`real` **`multi_normal_prec_log`**`(row_vectors` *`y`*`, vectors` *`mu`*`, matrix` *`Omega`*`)`

The log of the multivariate normal density of row vector(s) *y* given location vector(s) *mu* and positive definite precision matrix *Omega*

`real` **`multi_normal_prec_log`**`(row_vectors` *`y`*`, row_vectors` *`mu`*`, matrix` *`Omega`*`)`

The log of the multivariate normal density of row vector(s) *y* given location row vector(s) *mu* and positive definite precision matrix *Omega*

46.3. Multivariate Normal Distribution, Cholesky Parameterization

Probability Density Function

If $K \in \mathbb{N}$, $\mu \in \mathbb{R}^K$, and $L \in \mathbb{R}^{K \times K}$ is lower triangular and such that $LL^\top$ is positive definite, then for $y \in \mathbb{R}^K$,

$$\text{MultiNormalCholesky}(y|\mu, L) = \text{MultiNormal}(y|\mu, LL^\top).$$

Sampling Statement

`y ~ multi_normal_cholesky(mu,L);`

Increment log probability with `multi_normal_cholesky_log(y,mu,L)`, dropping constant additive terms; Section 24.3 explains sampling statements.

Stan Functions

`real multi_normal_cholesky_log(vectors y, vectors mu, matrix L)`

The log of the multivariate normal density of vector(s) *y* given location vector(s) *mu* and lower-triangular Cholesky factor of the covariance matrix *L*

`real multi_normal_cholesky_log(vectors y, row_vectors mu, matrix L)`

The log of the multivariate normal density of vector(s) *y* given location row vector(s) *mu* and lower-triangular Cholesky factor of the covariance matrix *L*

`real multi_normal_cholesky_log(row_vectors y, vectors mu, matrix L)`

The log of the multivariate normal density of row vector(s) *y* given location vector(s) *mu* and lower-triangular Cholesky factor of the covariance matrix *L*

`real multi_normal_cholesky_log(row_vectors y, row_vectors mu, matrix L)`

The log of the multivariate normal density of row vector(s) *y* given location row vector(s) *mu* and lower-triangular Cholesky factor of the covariance matrix *L*

`vector multi_normal_cholesky_rng(vector mu, matrix L)`

Generate a multivariate normal variate with location *mu* and lower-triangular Cholesky factor of the covariance matrix *L*; may only be used in generated quantities block

46.4. Multivariate Gaussian Process Distribution

Probability Density Function

If $K, N \in \mathbb{N}$, $\Sigma \in \mathbb{R}^{N\times N}$ is symmetric, positive definite kernel matrix and $w \in \mathbb{R}^K$ is a vector of positive inverse scales, then for $y \in \mathbb{R}^{K\times N}$,

$$\text{MultiGP}(y|\Sigma, w) = \prod_{i=1}^{K} \text{MultiNormal}(y_i|0, w_i^{-1}\Sigma),$$

where y_i is the ith row of y. This is used to efficiently handle Gaussian Processes with multi-variate outputs where only the output dimensions share a kernel function but vary based on their scale. Note that this function does not take into account the mean prediction.

Sampling Statement

`y ~ multi_gp(Sigma,w);`

Increment log probability with `multi_gp_log(y,Sigma,w)`, dropping constant additive terms; Section 24.3 explains sampling statements.

Stan Functions

`real multi_gp_log(matrix y, matrix Sigma, vector w)`

The log of the multivariate GP density of matrix `y` given kernel matrix `Sigma` and inverses scales `w`

46.5. Multivariate Gaussian Process Distribution, Cholesky parameterization

Probability Density Function

If $K, N \in \mathbb{N}$, $L \in \mathbb{R}^{N\times N}$ is lower triangular and such that $LL^\top$ is positive definite kernel matrix and $w \in \mathbb{R}^K$ is a vector of positive inverse scales, then for $y \in \mathbb{R}^{K\times N}$,

$$\text{MultiGPCholesky}(y|L, w) = \prod_{i=1}^{K} \text{MultiNormal}(y_i|0, w_i^{-1}LL^\top),$$

where y_i is the ith row of y. This is used to efficiently handle Gaussian Processes with multi-variate outputs where only the output dimensions share a kernel function but vary based on their scale. If the model allows parametrization in terms of Cholesky factor of the kernel matrix, this distribution is also more efficient than MultiGP(). Note that this function does not take into account the mean prediction.

Sampling Statement

`y ~ multi_gp_cholesky(L,w);`

Increment log probability with `multi_gp_cholesky_log(y,L,w)`, dropping constant additive terms; Section 24.3 explains sampling statements.

Stan Functions

`real multi_gp_cholesky_log(matrix y, matrix L, vector w)`

The log of the multivariate GP density of matrix *y* given lower-triangular Cholesky factor of the kernel matrix *L* and inverses scales *w*

46.6. Multivariate Student-*t* Distribution

Probability Density Function

If $K \in \mathbb{N}$, $\nu \in \mathbb{R}^+$, $\mu \in \mathbb{R}^K$, and $\Sigma \in \mathbb{R}^{K\times K}$ is symmetric and positive definite, then for $y \in \mathbb{R}^K$,

$$\text{MultiStudentT}(y|\nu,\mu,\Sigma) = \frac{1}{\pi^{K/2}}\ \frac{1}{\nu^{K/2}}\ \frac{\Gamma x\left((\nu+K)/2\right)}{\Gamma(\nu/2)}\ \frac{1}{\sqrt{|\Sigma|}}\ \left(1+\frac{1}{\nu}\ (y-\mu)^{\top}\ \Sigma^{-1}\ (y-\mu)\right)^{-(\nu+K)/2}.$$

Sampling Statement

`y ~ multi_student_t(nu,mu,Sigma);`

Increment log probability with `multi_student_t_log(y,nu,mu,Sigma)`, dropping constant additive terms; Section 24.3 explains sampling statements.

Stan Functions

`real multi_student_t_log(vectors y, real nu, vectors mu, matrix Sigma)`

The log of the multivariate Student-*t* density of vector(s) *y* given degrees of freedom *nu*, location vector(s) *mu*, and scale matrix *Sigma*

`real multi_student_t_log(vectors y, real nu, row_vectors mu, matrix Sigma)`

The log of the multivariate Student-*t* density of vector(s) *y* given degrees of freedom *nu*, location row vector(s) *mu*, and scale matrix *Sigma*

`real` **`multi_student_t_log`**`(row_vectors y, real nu, vectors mu, matrix Sigma)`
The log of the multivariate Student-t density of row vector(s) `y` given degrees of freedom `nu`, location vector(s) `mu`, and scale matrix `Sigma`

`real` **`multi_student_t_log`**`(row_vectors y, real nu, row_vectors mu, matrix Sigma)`
The log of the multivariate Student-t density of row vector(s) `y` given degrees of freedom `nu`, location row vector(s) `mu`, and scale matrix `Sigma`

`vector` **`multi_student_t_rng`**`(real nu, vector mu, matrix Sigma)`

Generate a multivariate Student-t variate with degrees of freedom `nu`, location `mu`, and scale matrix `Sigma`; may only be used in generated quantities block

46.7. Gaussian Dynamic Linear Models

A Gaussian Dynamic Linear model is defined as follows, For $t \in 1, \ldots, T$,

$$
\begin{aligned}
y_t &\sim N(F'\theta_t, V) \\
\theta_t &\sim N(G\theta_{t-1}, W) \\
\theta_0 &\sim N(m_0, C_0)
\end{aligned}
$$

where y is $n \times T$ matrix where rows are variables and columns are observations. These functions calculate the log-likelihood of the observations marginalizing over the latent states ($p(y|F, G, V, W, m_0, C_0)$). This log-likelihood is a system that is calculated using the Kalman Filter. If V is diagonal, then a more efficient algorithm which sequentially processes observations and avoids a matrix inversions can be used (Durbin and Koopman, 2001, Sec 6.4).

Sampling Statement

`y ~` **`gaussian_dlm_obs`**`(F,G,V,W,m0,C0);`
Increment log probability with `gaussian_dlm_obs_log(y,F,G,V,W,m0,C0)`, dropping constant additive terms; Section 24.3 explains sampling statements.

Stan Functions

The following two functions differ in the type of their `V`, the first taking a full observation covariance matrix `V` and the second a vector `V` representing the diagonal of the observation covariance matrix. The sampling statement defined in the previous section works with either type of observation `V`.

`real` **`gaussian_dlm_obs_log`**`(matrix` *`y`*`, matrix` *`F`*`, matrix` *`G`*`, matrix` *`V`*`,`
`matrix` *`W`*`, vector` *`m0`*`, matrix` *`C0`*`)`

The log of the density of the Gaussian Dynamic Linear model with observation matrix *y* in which rows are variables and columns are observations, design matrix *F*, transition matrix *G*, observation covariance matrix *V*, system covariance matrix *W*, and the initial state is distributed normal with mean *m0* and covariance *C0*.

`real` **`gaussian_dlm_obs_log`**`(matrix` *`y`*`, matrix` *`F`*`, matrix` *`G`*`, vector` *`V`*`,`
`matrix` *`W`*`, vector` *`m0`*`, matrix` *`C0`*`)`

The log of the density of the Gaussian Dynamic Linear model with observation matrix *y* in which rows are variables and columns are observations, design matrix *F*, transition matrix *G*, observation covariance matrix with diagonal *V*, system covariance matrix *W*, and the initial state is distributed normal with mean *m0* and covariance *C0*.

47. Simplex Distributions

The simplex probabilities have support on the unit K-simplex for a specified K. A K-dimensional vector θ is a unit K-simplex if $\theta_k \geq 0$ for $k \in \{1, \ldots, K\}$ and $\sum_{k=1}^{K} \theta_k = 1$.

47.1. Dirichlet Distribution

Probability Density Function

If $K \in \mathbb{N}$ and $\alpha \in (\mathbb{R}^+)^K$, then for $\theta \in K$-simplex,

$$\text{Dirichlet}(\theta|\alpha) = \frac{\Gamma\left(\sum_{k=1}^{K} \alpha_k\right)}{\prod_{k=1}^{K} \Gamma(\alpha_k)} \prod_{k=1}^{K} \theta_k^{\alpha_k - 1}.$$

Warning: If any of the components of θ satisfies $\theta_i = 0$ or $\theta_i = 1$, then the probability is 0 and the log probability is $-\infty$. Similarly, the distribution requires strictly positive parameters, with $\alpha_i > 0$ for each i.

Sampling Statement

`theta ~ dirichlet(alpha);`

Increment log probability with `dirichlet_log(theta,alpha)`, dropping constant additive terms; Section 24.3 explains sampling statements.

Stan Functions

`real dirichlet_log(vector theta, vector alpha)`

The log of the Dirichlet density for simplex *theta* given prior counts (plus one) *alpha*

`vector dirichlet_rng(vector alpha)`

Generate a Dirichlet variate with prior counts (plus one) *alpha*; may only be used in generated quantities block

48. Correlation Matrix Distributions

The correlation matrix distributions have support on the (Cholesky factors of) correlation matrices. A Cholesky factor L for a $K \times K$ correlation matrix Σ of dimension K has rows of unit length so that the diagonal of $LL^\top$ is the unit K-vector. Even though models are usually conceptualized in terms of correlation matrices, it is better to operationalize them in terms of their Cholesky factors. If you are interested in the posterior distribution of the correlations, you can recover them in the generated quantities block via

```
generated quantities {
  corr_matrix[K] Sigma;
  Sigma <- multiply_lower_tri_self_transpose(L);
}
```

48.1. LKJ Correlation Distribution

Probability Density Function

For $\eta > 0$, if Σ a positive-definite, symmetric matrix with unit diagonal (i.e., a correlation matrix), then

$$\text{LkjCorr}(\Sigma|\eta) \propto \det(\Sigma)^{(\eta-1)}.$$

The expectation is the identity matrix for any positive value of the shape parameter η, which can be interpreted like the shape parameter of a symmetric beta distribution:

- if $\eta = 1$, then the density is uniform over all correlation matrices of a given order;
- if $\eta > 1$, the identity matrix is the modal correlation matrix, with a sharper peak in the density at the identity matrix for larger η; and
- for $0 < \eta < 1$, the density has a trough at the identity matrix.
- if η were an unknown parameter, the Jeffreys prior is proportional to $\sqrt{2\sum_{k=1}^{K-1}\left(\psi_1\left(\eta + \frac{K-k-1}{2}\right) - 2\psi_1(2\eta + K - k - 1)\right)}$, where $\psi_1()$ is the trigamma function

See (Lewandowski et al., 2009) for definitions. However, it is much better computationally to work directly with the Cholesky factor of Σ, so this distribution should never be explicitly used in practice.

Sampling Statement

`y ~ lkj_corr(eta);`

Increment log probability with `lkj_corr_log(y,eta)`, dropping constant additive terms; Section 24.3 explains sampling statements.

Stan Functions

`real lkj_corr_log(matrix y, real eta)`

The log of the LKJ density for the correlation matrix *y* given nonnegative shape *eta*. The only reason to use this density function is if you want the code to run slower and consume more memory with more risk of numerical errors. Use its Cholesky factor as described in the next section.

`matrix lkj_corr_rng(int K, real eta)`

Generate a LKJ random correlation matrix of order *K* with shape *eta*; may only be used in generated quantities block

48.2. Cholesky LKJ Correlation Distribution

Stan provides an implicit parameterization of the LKJ correlation matrix density in terms of its Cholesky factor, which you should use rather than the explicit parameterization in the previous section. For example, if L is a Cholesky factor of a correlation matrix, then

```
L ~ lkj_corr_cholesky(2.0);
# implies L * L' ~ lkj_corr(2.0);
```

Because Stan requires models to have support on all valid constrained parameters, L will almost always [1] be a parameter declared with the type of a Cholesky factor for a correlation matrix; for example,

```
parameters {
  cholesky_factor_corr[K] L;
  # rather than corr_matrix[K] Sigma;
  ...
```

[1]It is possible to build up a valid L within Stan, but that would then require Jacobian adjustments to imply the intended posterior.

Probability Density Function

For $\eta > 0$, if L is the Cholesky factor of a symmetric positive-definite matrix with unit diagonal (i.e., a correlation matrix), then

$$\text{LkjCholesky}(L|\eta) \propto |J| \det(LL^\top)^{(\eta-1)} = \prod_{k=2}^{K} L_{kk}^{K-k+2\eta-2}.$$

See the previous section for details on interpreting the shape parameter η. Note that even if $\eta = 1$, it is still essential to evaluate the density function because the density of L is not constant, regardless of the value of η, even though the density of $LL^\top$ is constant iff $\eta = 1$.

Sampling Statement

`L ~ `**`lkj_corr_cholesky`**`(eta);`

Increment log probability with `lkj_corr_cholesky_log(L,eta)`, dropping constant additive terms; Section 24.3 explains sampling statements.

Stan Functions

`real `**`lkj_corr_cholesky_log`**`(matrix L, real eta)`

The log of the LKJ density for the lower-triangular Cholesky factor `L` of a correlation matrix given shape `eta`.

`matrix `**`lkj_corr_cholesky_rng`**`(int K, real eta)`

Generate a random Cholesky factor of a correlation matrix of order `K` that is distributed LKJ with shape `eta`; may only be used in generated quantities block

49. Covariance Matrix Distributions

The covariance matrix distributions have support on symmetric, positive-definite $K \times K$ matrices.

49.1. Wishart Distribution

Probability Density Function

If $K \in \mathbb{N}$, $\nu \in (K-1, \infty)$, and $S \in \mathbb{R}^{K \times K}$ is symmetric and positive definite, then for symmetric and positive-definite $W \in \mathbb{R}^{K \times K}$,

$$\text{Wishart}(W|\nu, S) = \frac{1}{2^{\nu K/2}} \frac{1}{\Gamma_K\left(\frac{\nu}{2}\right)} |S|^{-\nu/2} |W|^{(\nu-K-1)/2} \exp\left(-\frac{1}{2} \operatorname{tr}\left(S^{-1}W\right)\right),$$

where tr() is the matrix trace function, and $\Gamma_K()$ is the multivariate Gamma function,

$$\Gamma_K(x) = \frac{1}{\pi^{K(K-1)/4}} \prod_{k=1}^{K} \Gamma\left(x + \frac{1-k}{2}\right).$$

Sampling Statement

`W ~ wishart(nu,Sigma);`

Increment log probability with `wishart_log(W,nu,Sigma)`, dropping constant additive terms; Section 24.3 explains sampling statements.

Stan Functions

`real wishart_log(matrix W, real nu, matrix Sigma)`

The log of the Wishart density for symmetric and positive-definite matrix `W` given degrees of freedom `nu` and symmetric and positive-definite scale matrix `Sigma`

`matrix wishart_rng(real nu, matrix Sigma)`

Generate a Wishart variate with degrees of freedom `nu` and symmetric and positive-definite scale matrix `Sigma`; may only be used in generated quantities block

49.2. Inverse Wishart Distribution

Probability Density Function

If $K \in \mathbb{N}$, $\nu \in (K-1, \infty)$, and $S \in \mathbb{R}^{K \times K}$ is symmetric and positive definite, then for symmetric and positive-definite $W \in \mathbb{R}^{K \times K}$,

$$\text{InvWishart}(W|\nu, S) = \frac{1}{2^{\nu K/2}} \frac{1}{\Gamma_K\left(\frac{\nu}{2}\right)} |S|^{\nu/2} |W|^{-(\nu+K+1)/2} \exp\left(-\frac{1}{2} \text{tr}(SW^{-1})\right).$$

Sampling Statement

`W ~ inv_wishart(nu,Sigma);`

Increment log probability with `inv_wishart_log(W,nu,Sigma)`, dropping constant additive terms; Section 24.3 explains sampling statements.

Stan Functions

`real inv_wishart_log(matrix W, real nu, matrix Sigma)`

The log of the inverse Wishart density for symmetric and positive-definite matrix `W` given degrees of freedom `nu` and symmetric and positive-definite scale matrix `Sigma`

`matrix inv_wishart_rng(real nu, matrix Sigma)`

Generate an inverse Wishart variate with degrees of freedom `nu` and symmetric and positive-definite scale matrix `Sigma`; may only be used in generated quantities block

Part VII

Additional Topics

50. Point Estimation

This chapter defines the workhorses of non-Bayesian estimation, maximum likelihood and penalized maximum likelihood, and relates them to Bayesian point estimation based on posterior means, medians, and modes. Such estimates are called "point estimates" because they are composed of a single value for the model parameters θ rather than a posterior distribution.

Stan's optimizer can be used to implement (penalized) maximum likelihood estimation for any likelihood function and penalty function that can be coded in Stan's modeling language. Stan's optimizer can also be used for point estimation in Bayesian settings based on posterior modes. Stan's Markov chain Monte Carlo samplers can be used to implement point inference in Bayesian models based on posterior means or medians.

50.1. Maximum Likelihood Estimation

Given a likelihood function $p(y|\theta)$ and a fixed data vector y, the maximum likelihood estimate (MLE) is the parameter vector $\hat{\theta}$ that maximizes the likelihood, i.e.,

$$\hat{\theta} = \text{argmax}_{\theta}\ p(y|\theta).$$

It is usually more convenient to work on the log scale. An equivalent[1] formulation of the MLE is

$$\hat{\theta} = \text{argmax}_{\theta}\ \log p(y|\theta).$$

Existence of Maximum Likelihood Estimates

Because not all functions have unique maximum values, maximum likelihood estimates are not guaranteed to exist. As discussed in Chapter 18, this situation can arise when

- there is more than one point that maximizes the likelihood function,
- the likelihood function is unbounded, or
- the likelihood function is bounded by an asymptote that is never reached for legal parameter values.

These problems persist with the penalized maximum likelihood estimates discussed in the next section, and Bayesian posterior modes as discussed in the following section.

[1] The equivalence follows from the fact that densities are positive and the log function is strictly monotonic, i.e., $p(y|\theta) \geq 0$ and for all $a, b > 0$, $\log a > \log b$ if and only if $a > b$.

Example: Linear Regression

Consider an ordinary linear regression problem with an N-dimensional vector of observations y, an $(N \times K)$-dimensional data matrix x of predictors, a K-dimensional parameter vector β of regression coefficients, and a real-valued noise scale $\sigma > 0$, with log likelihood function

$$\log p(y|\beta, x) = \sum_{n=1}^{N} \log \mathsf{Normal}(y_n | x_n\beta, \sigma).$$

The maximum likelihood estimate for $\theta = (\beta, \sigma)$ is just

$$(\hat{\beta}, \hat{\sigma}) \;=\; \text{argmax}_{\beta,\sigma} \log p(y|\beta, \sigma, x) = \sum_{n=1}^{N} \log \mathsf{Normal}(y_n | x_n\beta, \sigma).$$

Squared Error

A little algebra on the log likelihood function shows that the marginal maximum likelihood estimate $\hat{\theta} = (\hat{\beta}, \hat{\sigma})$ can be equivalently formulated for $\hat{\beta}$ in terms of least squares. That is, $\hat{\beta}$ is the value for the coefficient vector that minimizes the sum of squared prediction errors,

$$\hat{\beta} \;=\; \text{argmin}_{\beta} \sum_{n=1}^{N} (y_n - x_n\beta)^2 \;=\; \text{argmin}_{\beta} (y - x\beta)^{\top}(y - x\beta).$$

The residual error for data item n is the difference between the actual value and predicted value, $y_n - x_n\hat{\beta}$. The maximum likelihood estimate for the noise scale, $\hat{\sigma}$ is just the square root of the average squared residual,

$$\hat{\sigma}^2 \;=\; \frac{1}{N} \sum_{n=1}^{N} \left(y_n - x_n\hat{\beta}\right)^2 \;=\; \frac{1}{N}(y - x\hat{\beta})^{\top}(y - x\hat{\beta}).$$

Minimizing Squared Error in Stan

The squared error approach to linear regression can be directly coded in Stan with the following model.

```
data {
  int<lower=0> N;
  int<lower=1> K;
  vector[N] y;
  matrix[N,K] x;
```

```
}
parameters {
  vector[K] beta;
}
transformed parameters {
  real<lower=0> squared_error;
  squared_error <- dot_self(y - x * beta);
}
model {
  increment_log_prob(-squared_error);
}
generated quantities {
  real<lower=0> sigma_squared;
  sigma_squared <- squared_error / N;
}
```

Running Stan's optimizer on this model produces the MLE for the linear regression by directly minimizing the sum of squared errors and using that to define the noise scale as a generated quantity.

By replacing `N` with `N-1` in the denominator of the definition of `sigma_squared`, the more commonly supplied unbiased estimate of σ^2 can be calculated; see Section 50.6 for a definition of estimation bias and a discussion of estimating variance.

50.2. Penalized Maximum Likelihood Estimation

There is nothing special about a likelihood function as far as the ability to perform optimization is concerned. It is common among non-Bayesian statisticians to add so-called "penalty" functions to log likelihoods and optimize the new function. The penalized maximum likelihood estimator for a log likelihood function $\log p(y|\theta)$ and penalty function $r(\theta)$ is defined to be

$$\hat{\theta} = \text{argmax}_{\theta} \log p(y|\theta) - r(\theta).$$

The penalty function $r(\theta)$ is negated in the maximization so that the estimate $\hat{\theta}$ balances maximizing the log likelihood and minimizing the penalty. Penalization is sometimes called "regularization."

Examples

Ridge Regression

Ridge regression (Hoerl and Kennard, 1970) is based on penalizing the Euclidean length of the coefficient vector β. The ridge penalty function is

$$r(\beta) \; = \; \lambda \sum_{k=1}^{K} \beta_k^2 \; = \; \lambda \, \beta^\top \beta,$$

where λ is a constant tuning parameter that determines the magnitude of the penalty.

Therefore, the penalized maximum likelihood estimate for ridge regression is just

$$(\hat{\beta}, \hat{\sigma}) \; = \; \text{argmax}_{\beta,\sigma} \sum_{n=1}^{N} \log \mathsf{Normal}(y_n | x_n \beta, \sigma) - \lambda \sum_{k=1}^{K} \beta_k^2$$

The ridge penalty is sometimes called L2 regularization or shrinkage, because of its relation to the L2 norm.

Like the basic MLE for linear regression, the ridge regression estimate for the coefficients β can also be formulated in terms of least squares,

$$\hat{\beta} \; = \; \text{argmin}_{\beta} \sum_{n=1}^{N} (y_n - x_n \beta)^2 + \sum_{k=1}^{K} \beta_k^2 \; = \; \text{argmin}_{\beta} \, (y - x\beta)^\top (y - x\beta) + \lambda \beta^\top \beta.$$

The effect of adding the ridge penalty function is that the ridge regression estimate for β is a vector of shorter length, or in other words, $\hat{\beta}$ is shrunk. The ridge estimate does not necessarily have a smaller absolute β_k for each k, nor does the coefficient vector necessarily point in the same direction as the maximum likelihood estimate.

In Stan, adding the ridge penalty involves adding its magnitude as a data variable and the penalty itself to the model block,

```
data {
  ...
  real<lower=0> lambda;
}
...
model {
  ...
  increment_log_prob(- lambda * dot_self(beta));
}
```

The noise term calculation remains the same.

The Lasso

The lasso (Tibshirani, 1996) is an alternative to ridge regression that applies a penalty based on the sum of the absolute coefficients, rather than the sum of their squares,

$$r(\beta) = \lambda \sum_{k=1}^{K} |\beta_k|.$$

The lasso is also called L1 shrinkage due to its relation to the L1 norm, which is also known as taxicab distance or Manattan distance.

Because the derivative of the penalty does not depend on the value of the β_k,

$$\frac{d}{d\beta_k} \lambda \sum_{k=1}^{K} |\beta_k| = \text{signum}(\beta_k),$$

it has the effect of shrinking parameters all the way to 0 in maximum likelihood estimates. Thus it can be used for variable selection as well as just shrinkage.

But there is one drawback. Stan's optimizers are not set up to deal with the absolute value in the formula, so they are unlikely to drive estimates all the way to 0.

The lasso can be implemented in Stan just as easily as ridge regression, with the magnitude declared as data and the penalty added to the model block,

```
data {
  ...
  real<lower=0> lambda;
}
...
model {
  ...
  for (k in 1:K)
    increment_log_prob(- lambda * abs(beta[k]));
}
```

The Elastic Net

The naive elastic net (Zou and Hastie, 2005) involves a weighted average of ridge and lasso penalties, with a penalty function

$$r(\beta) = \lambda_1 \sum_{k=1}^{K} \beta_k^2 + \lambda_2 \sum_{k=1}^{K} |\beta_k|.$$

The naive elastic net combines properties of both ridge regression and the lasso, providing both identification and variable selection.

The naive elastic net can be implemented directly in Stan by combining implementations of ridge regression and the lasso, as

```
data {
  real<lower=0> lambda1;
  real<lower=0> lambda2;
  ...
}
...
model {
  ...
  increment_log_prob(lambda2 * dot_self(beta));
  for (k in 1:K)
    increment_log_prob(lambda1 * abs(beta[k]));
}
```

The elastic net (Zou and Hastie, 2005) involves adjusting the final estimate for β based on the fit $\hat{\beta}$ produced by the naive elastic net. The elastic net estimate is

$$\hat{\beta} = (1 + \lambda_2)\beta^*$$

where β^* is the naive elastic net estimate.

To implement the elastic net in Stan, the data, parameter, and model blocks are the same as for the naive elastic net. In addition, the elastic net estimate is calculated in the generated quantities block.

```
generated quantities {
  vector[K] beta_elastic_net;
  ...
  beta_elastic_net <- (1 + lambda2) * beta;
}
```

The error scale also needs to be calculated in the generated quantities block based on the elastic net coefficients `beta_elastic_net`.

Other Penalized Regressions

It is also common to use penalty functions that bias the coefficient estimates toward values other than 0, as in the estimators of James and Stein (1961). Penalty functions can also be used to bias estimates toward population means; see (Efron and Morris, 1975; Efron, 2012). This latter approach is similar to the hierarchical models commonly employed in Bayesian statistics.

50.3. Posterior Mode Estimation

There are three common approaches to Bayesian point estimation based on the posterior $p(\theta|y)$ of parameters θ given observed data y: the mode (maximum), the mean, and the median. This section covers estimates based on the parameters θ that maximize the posterior density, and the next sections continue with discussions of the mean and median.

An estimate based on a model's posterior mode can be defined by

$$\hat{\theta} = \text{argmax}_{\theta}\, p(\theta|y).$$

When it exists, $\hat{\theta}$ maximizes the posterior density of the parameters given the data. The posterior mode is sometimes called the "maximum a posteriori" (MAP) estimate.

As discussed in Chapter 18 and Section 50.1, a unique posterior mode might not exist—there may be no value that maximizes the posterior mode or there may be more than one. In these cases, the posterior mode estimate is undefined. Stan's optimizer, like most optimizers, will have problems in these situations. It may also return a locally maximal value that is not the global maximum.

In cases where there is a posterior mode, it will correspond to a penalized maximum likelihood estimate with a penalty function equal to the negation of the log prior. This is because Bayes's rule,

$$p(\theta|y) = \frac{p(y|\theta)\, p(\theta)}{p(y)},$$

ensures that

$$\begin{aligned} \text{argmax}_{\theta}\; p(\theta|y) &= \text{argmax}_{\theta}\; \frac{p(y|\theta)\, p(\theta)}{p(y)} \\ &= \text{argmax}_{\theta}\; p(y|\theta)\, p(\theta), \end{aligned}$$

and the positiveness of densities and the strict monotonicity of log ensure that

$$\text{argmax}_{\theta}\; p(y|\theta)\, p(\theta) \;=\; \text{argmax}_{\theta}\; \log p(y|\theta) + \log p(\theta).$$

In the case where the prior (proper or improper) is uniform, the posterior mode is equivalent to the maximum likelihood estimate.

For most commonly used penalty functions, there are probabilistic equivalents. For example, the ridge penalty function corresponds to a normal prior on coefficients and the lasso to a Laplace prior. The reverse is always true—a negative prior can always be treated as a penalty function.

50.4. Posterior Mean Estimation

A standard Bayesian approach to point estimation is to use the posterior mean (assuming it exists), defined by

$$\hat{\theta} = \int \theta \, p(\theta|y) \, d\theta.$$

The posterior mean is often called *the* Bayesian estimator, because it's the estimator that minimizes the expected square error of the estimate.

An estimate of the posterior mean for each parameter is returned by Stan's interfaces; see the RStan, CmdStan, and PyStan user's guides for details on the interfaces and data formats.

Posterior means exist in many situations where posterior modes do not exist. For example, in the $\mathsf{Beta}(0.1, 0.1)$ case, there is no posterior mode, but posterior mean is well defined with value 0.5.

A situation where posterior means fail to exist but posterior modes do exist is with a posterior with a Cauchy distribution $\mathsf{Cauchy}(\mu, \tau)$. The posterior mode is μ, but the integral expressing the posterior mean diverges. Such diffuse priors rarely arise in practical modeling applications; even with a Cauchy Cauchy prior for some parameters, data will provide enough constraints that the posterior is better behaved and means exist.

Sometimes when posterior means exist, they are not meaningful, as in the case of a multimodal posterior arising from a mixture model or in the case of a uniform distribution on a closed interval.

50.5. Posterior Median Estimation

The posterior median (i.e., 50th percentile or 0.5 quantile) is another popular point estimate reported for Bayesian models. The posterior median minimizes the expected absolute error of estimates. These estimates are returned in the various Stan interfaces; see the RStan, PyStan and CmdStan user's guides for more information on format.

Although posterior medians may fail to be meaningful, they often exist even where posterior means do not, as in the Cauchy distribution.

50.6. Estimation Error, Bias, and Variance

An estimate $\hat{\theta}$ depends on the particular data y and either the log likelihood function, $\log p(y|\theta)$, penalized log likelihood function $\log p(y|\theta) - r(\theta)$, or log probability function $\log p(y, \theta) = \log p(y, \theta) + \log p(\theta)$. In this section, the notation $\hat{\theta}$ is overloaded

to indicate the estimator, which is an implicit function of the data and (penalized) likelihood or probability function.

Estimation Error

For a particular observed data set y generated according to true parameters θ, the estimation error is the difference between the estimated value and true value of the parameter,

$$\text{err}(\hat{\theta}) = \hat{\theta} - \theta.$$

Estimation Bias

For a particular true parameter value θ and a likelihood function $p(y|\theta)$, the expected estimation error averaged over possible data sets y according to their density under the likelihood is

$$\mathbb{E}_{p(y|\theta)}[\hat{\theta}] = \int (\text{argmax}_{\theta'} p(y|\theta'))\, p(y|\theta) dy.$$

An estimator's bias is the expected estimation error,

$$\mathbb{E}_{p(y|\theta)}[\hat{\theta} - \theta] = \mathbb{E}_{p(y|\theta)}[\hat{\theta}] - \theta$$

The bias is a multivariate quantity with the same dimensions as θ. An estimator is unbiased if its expected estimation error is zero and biased otherwise.

Example: Estimating a Normal Distribution

Suppose a data set of observations y_n for $n \in 1{:}N$ drawn from a normal distribution. This presupposes a model $y_n \sim \mathsf{Normal}(\mu, \sigma)$, where both μ and $\sigma > 0$ are parameters. The log likelihood is just

$$\log p(y|\mu, \sigma) = \sum_{n=1}^{N} \log \mathsf{Normal}(y_n|\mu, \sigma).$$

The maximum likelihood estimator for μ is just the sample mean, i.e., the average of the samples,

$$\hat{\mu} = \frac{1}{N} \sum_{n=1}^{N} y_n.$$

The maximum likelihood estimate for the mean is unbiased.

The maximum likelihood estimator for the variance σ^2 is the average of the squared difference from the mean,

$$\hat{\sigma}^2 = \frac{1}{N} \sum_{n=1}^{N} (y_n - \hat{\mu})^2.$$

The maximum likelihood for the variance is biased on the low side, i.e.,

$$\mathbb{E}_{p(y|\mu,\sigma)}[\hat{\sigma}^2] < \sigma.$$

The reason for this bias is that the maximum likelihood estimate is based on the difference from the estimated mean $\hat{\mu}$. Plugging in the actual mean can lead to larger sum of squared differences; if $\mu \neq \hat{\mu}$, then

$$\frac{1}{N} \sum_{n=1}^{N} (y_n - \mu)^2 > \frac{1}{N} \sum_{n=1}^{N} (y_n - \hat{\mu})^2.$$

An alternative estimate for the variance is the sample variance, which is defined by

$$\hat{\mu} = \frac{1}{N-1} \sum_{n=1}^{N} (y_n - \hat{\mu})^2.$$

This value is larger than the maximum likelihood estimate by a factor of $N/(N-1)$.

Estimation Variance

The variance of component k of an estimator $\hat{\theta}$ is computed like any other variance, as the expected squared difference from its expectation,

$$\mathrm{var}_{p(y|\theta)}[\hat{\theta}_k] \;=\; \mathbb{E}_{p(y|\theta)}[\,(\hat{\theta}_k - \mathbb{E}_{p(y|\theta)}[\hat{\theta}_k])^2\,].$$

The full $K \times K$ covariance matrix for the estimator is thus defined, as usual, by

$$\mathrm{covar}_{p(y|\theta)}[\hat{\theta}] \;=\; \mathbb{E}_{p(y|\theta)}[\,(\hat{\theta} - \mathbb{E}[\hat{\theta}])\,(\hat{\theta} - \mathbb{E}[\hat{\theta}])^{\top}\,].$$

Continuing the example of estimating the mean and variance of a normal distribution based on sample data, the maximum likelihood estimator (i.e., the sample mean) is the unbiased estimator for the mean μ with the lowest variance; the Gauss-Markov theorem establishes this result in some generality for least-squares estimation, or equivalently, maximum likelihood estimation under an assumption of normal noise; see (Hastie et al., 2009, Section 3.2.2).

51. Bayesian Data Analysis

Gelman et al. (2013) provide the following characterization of Bayesian data analysis.

> By Bayesian data analysis, we mean practical methods for making inferences from data using probability models for quantities we observe and about which we wish to learn.

They go on to describe how Bayesian statistics differs from frequentist approaches.

> The essential characteristic of Bayesian methods is their explicit use of probability for quantifying uncertainty in inferences based on statistical analysis.

Because they view probability as the limit of relative frequencies of observations, strict frequentists forbid probability statements about parameters. Parameters are considered fixed, not random.

Bayesians also treat parameters as fixed but unknown. But unlike frequentists, they make use of both prior distributions over parameters and posterior distributions over parameters. These prior and posterior probabilities and posterior predictive probabilities are intended to characterize knowledge about the parameters and future observables. Posterior distributions form the basis of Bayesian inference, as described below.

51.1. Bayesian Modeling

(Gelman et al., 2013) break applied Bayesian modeling into the following three steps.

1. Set up a full probability model for all observable and unobservable quantities. This model should be consistent with existing knowledge of the data being modeled and how it was collected.

2. Calculate the posterior probability of unknown quantities conditioned on observed quantities. The unknowns may include unobservable quantities such as parameters and potentially observable quantities such as predictions for future observations.

3. Evaluate the model fit to the data. This includes evaluating the implications of the posterior.

Typically, this cycle will be repeated until a sufficient fit is achieved in the third step. Stan automates the calculations involved in the second and third steps.

51.2. Bayesian Inference

Basic Quantities

The mechanics of Bayesian inference follow directly from Bayes's rule. To fix notation, let y represent observed quantities such as data and let θ represent unknown quantities such as parameters and future observations. Both y and θ will be modeled as random. Let x represent known, but unmodeled quantities such as constants, hyperparameters, and predictors.

Probability Functions

The probability function $p(y, \theta)$ is the joint probability function of the data y and parameters θ. The constants and predictors x are implicitly understood as being part of the conditioning. The conditional probability function $p(y|\theta)$ of the data y given parameters θ and constants x is called the sampling probability function; it is also called the likelihood function when viewed as a function of θ for fixed y and x.

The probability function $p(\theta)$ over the parameters given the constants x is called the prior because it characterizes the probability of the parameters before any data is observed. The conditional probability function $p(\theta|y)$ is called the posterior because it characterizes the probability of parameters given observed data y and constants x.

Bayes's Rule

The technical apparatus of Bayesian inference hinges on the following chain of equations, known in various forms as Bayes's rule (where again, the constants x are implicit).

$$
\begin{aligned}
p(\theta|y) &= \frac{p(\theta, y)}{p(y)} && \text{[definition of conditional probability]} \\
&= \frac{p(y|\theta)\, p(\theta)}{p(y)} && \text{[chain rule]} \\
&= \frac{p(y|\theta)\, p(\theta)}{\int_\Theta p(y, \theta)\, d\theta} && \text{[law of total probability]} \\
&= \frac{p(y|\theta)\, p(\theta)}{\int_\Theta p(y|\theta)\, p(\theta)\, d\theta} && \text{[chain rule]} \\
&\propto p(y|\theta)\, p(\theta) && \text{[}y\text{ is fixed]}
\end{aligned}
$$

Bayes's rule "inverts" the probability of the posterior $p(\theta|y)$, expressing it solely in terms of the likelihood $p(y|\theta)$ and prior $p(\theta)$ (again, with constants and predictors

x implicit). The last step is important for Stan, which only requires probability functions to be characterized up to a constant multiplier.

Predictive Inference

The uncertainty in the estimation of parameters θ from the data y (given the model) is characterized by the posterior $p(\theta|y)$. The posterior is thus crucial for Bayesian predictive inference.

If $\tilde{y}$ is taken to represent new, perhaps as yet unknown, observations, along with corresponding constants and predictors $\tilde{x}$, then the posterior predictive probability function is given by

$$p(\tilde{y}|y) = \int_{\Theta} p(\tilde{y}|\theta)\, p(\theta|y)\, d\theta.$$

Here, both the original constants and predictors x and the new constants and predictors $\tilde{x}$ are implicit. Like the posterior itself, predictive inference is characterized probabilistically. Rather than using a point estimate of the parameters θ, predictions are made based on averaging the predictions over a range of θ weighted by the posterior probability $p(\theta|y)$ of θ given data y (and constants x).

The posterior may also be used to estimate event probabilities. For instance, the probability that a parameter θ_k is greater than zero is characterized probabilistically by

$$\Pr[\theta_k > 0] = \int_{\Theta} \mathrm{I}(\theta_k > 0)\, p(\theta|y)\, d\theta.$$

The indicator function, $\mathrm{I}(\phi)$, evaluates to one if the proposition ϕ is true and evaluates to zero otherwise.

Comparisons involving future observables may be carried out in the same way. For example, the probability that $\tilde{y}_n > \tilde{y}_{n'}$ can be characterized using the posterior predictive probability function as

$$\Pr[\tilde{y}_n > \tilde{y}_{n'}] = \int_{\Theta} \int_{Y} \mathrm{I}(\tilde{y}_n > \tilde{y}_{n'})\, p(\tilde{y}|\theta) p(\theta|y)\, d\tilde{y}\, d\theta.$$

Posterior Predictive Checking

After the parameters are fit to data, they can be used to simulate a new data set by running the model inferences in the forward direction. These replicated data sets can then be compared to the original data either visually or statistically to assess model fit (Gelman et al., 2013, Chapter 6).

In Stan, posterior simulations can be generated in two ways. The first approach is to treat the predicted variables as parameters and then define their distributions in the model block. The second approach, which also works for discrete variables, is to

generate replicated data using random-number generators in the generated quantities block.

52. Markov Chain Monte Carlo Sampling

Like BUGS, Stan uses Markov chain Monte Carlo (MCMC) techniques to generate samples from the posterior distribution for inference.

52.1. Monte Carlo Sampling

Monte Carlo methods were developed to numerically approximate integrals that are not tractable analytically but for which evaluation of the function being integrated is tractable (Metropolis and Ulam, 1949).

For example, the mean μ of a probability density $p(\theta)$ is defined by the integral

$$\mu = \int_{\Theta} \theta \times p(\theta)\, d\theta.$$

For even a moderately complex Bayesian model, the posterior density $p(\theta|y)$ leads to an integral that is impossible to evaluate analytically. The posterior also depends on the constants and predictors x, but from here, they will just be elided and taken as given.

Now suppose it is possible to draw independent samples from $p(\theta)$ and let $\theta^{(1)}, \theta^{(2)}, \ldots, \theta^{(N)}$ be N such samples. A Monte Carlo estimate $\hat{\mu}$ of the mean μ of $p(\theta)$ is given by the sample average,

$$\hat{\mu} = \frac{1}{N} \sum_{n=1}^{N} \theta^{(n)}.$$

If the probability function $p(\theta)$ has a finite mean and variance, the law of large numbers ensures the Monte Carlo estimate converges to the correct value as the number of samples increases,

$$\lim_{N \rightarrow \infty} \hat{\mu} = \mu.$$

Assuming finite mean and variance, estimation error is governed by the central limit theorem, so that estimation error decreases as the square root of N,

$$|\mu - \hat{\mu}| \propto \frac{1}{\sqrt{N}}.$$

Therefore, estimating a mean to an extra decimal place of accuracy requires one hundred times more samples; adding two decimal places means ten thousand times as many samples. This makes Monte Carlo methods more useful for rough estimates to within a few decimal places than highly precise estimates. In practical applications, there is no point estimating a quantity beyond the uncertainty of the data sample on which it is based, so this lack of many decimal places of accuracy is rarely a problem in practice for statistical models.

52.2. Markov Chain Monte Carlo Sampling

Markov chain Monte Carlo (MCMC) methods were developed for situations in which it is not straightforward to draw independent samples (Metropolis et al., 1953).

A Markov chain is a sequence of random variables $\theta^{(1)}, \theta^{(2)}, \ldots$ where each variable is conditionally independent of all other variables given the value of the previous value. Thus if $\theta = \theta^{(1)}, \theta^{(2)}, \ldots, \theta^{(N)}$, then

$$p(\theta) = p(\theta^{(1)}) \prod_{n=2}^{N} p(\theta^{(n)}|\theta^{(n-1)}).$$

Stan uses Hamiltonian Monte Carlo to generate a next state in a manner described in Chapter 54.

The Markov chains Stan and other MCMC samplers generate are ergodic in the sense required by the Markov chain central limit theorem, meaning roughly that there is there is a reasonable chance of reaching one value of θ from another. The Markov chains are also stationary, meaning that the transition probabilities do not change at different positions in the chain, so that for $n, n' \geq 0$, the probability function $p(\theta^{(n+1)}|\theta^{(n)})$ is the same as $p(\theta^{(n'+1)}|\theta^{(n')})$ (following the convention of overloading random and bound variables and picking out a probability function by its arguments).

Stationary Markov chains have an equilibrium distribution on states in which each has the same marginal probability function, so that $p(\theta^{(n)})$ is the same probability function as $p(\theta^{(n+1)})$. In Stan, this equilibrium distribution $p(\theta^{(n)})$ is the probability function $p(\theta)$ being sampled, typically a Bayesian posterior density.

Using MCMC methods introduces two difficulties that are not faced by independent sample Monte Carlo methods. The first problem is determining when a randomly initialized Markov chain has converged to its equilibrium distribution. The second problem is that the draws from a Markov chain are correlated, and thus the central limit theorem's bound on estimation error no longer applies. These problems are addressed in the next two sections.

52.3. Initialization and Convergence Monitoring

A Markov chain generates samples from the target distribution only after it has converged to equilibrium. Unfortunately, this is only guaranteed in the limit in theory. In practice, diagnostics must be applied to monitor whether the Markov chain(s) have converged.

Potential Scale Reduction

One way to monitor whether a chain has converged to the equilibrium distribution is to compare its behavior to other randomly initialized chains. This is the motivation for the Gelman and Rubin (1992) potential scale reduction statistic, $\hat{R}$. The $\hat{R}$ statistic measures the ratio of the average variance of samples within each chain to the variance of the pooled samples across chains; if all chains are at equilibrium, these will be the same and $\hat{R}$ will be one. If the chains have not converged to a common distribution, the $\hat{R}$ statistic will be greater than one.

Gelman and Rubin's recommendation is that the independent Markov chains be initialized with diffuse starting values for the parameters and sampled until all values for $\hat{R}$ are below 1.1. Stan allows users to specify initial values for parameters and it is also able to draw diffuse random initializations itself.

The $\hat{R}$ statistic is defined for a set of M Markov chains, θ_m, each of which has N samples $\theta_m^{(n)}$. The between-sample variance estimate is

$$B = \frac{N}{M-1} \sum_{m=1}^{M} (\bar{\theta}_m^{(\bullet)} - \bar{\theta}_\bullet^{(\bullet)})^2,$$

where

$$\bar{\theta}_m^{(\bullet)} = \frac{1}{N} \sum_{n=1}^{N} \theta_m^{(n)} \quad \text{and} \quad \bar{\theta}_\bullet^{(\bullet)} = \frac{1}{M} \sum_{m=1}^{M} \bar{\theta}_m^{(\bullet)}.$$

The within-sample variance is

$$W = \frac{1}{M} \sum_{m=1}^{M} s_m^2,$$

where

$$s_m^2 = \frac{1}{N-1} \sum_{n=1}^{N} (\theta_m^{(n)} - \bar{\theta}_m^{(\bullet)})^2.$$

The variance estimator is

$$\widehat{\text{var}}^{+}(\theta|y) = \frac{N-1}{N} W + \frac{1}{N} B.$$

Finally, the potential scale reduction statistic is defined by

$$\hat{R} = \sqrt{\frac{\widehat{\text{var}}^{+}(\theta|y)}{W}}.$$

Generalized $\hat{R}$ for Ragged Chains

Now suppose that each chain may have a different number of samples. Let N_m be the number of samples in chain m. Now the formula for the within-chain mean for chain

m uses the size of the chain, N_m,

$$\bar{\theta}_m^{(\bullet)} = \frac{1}{N_m} \sum_{n=1}^{N} \theta_n^{(m)},$$

as does the within-chain variance estimate,

$$s_m^2 = \frac{1}{N_m - 1} \sum_{n=1}^{N_m} (\theta_m^{(n)} - \bar{\theta}_m^{(\bullet)})^2.$$

The terms that average over chains, such as $\bar{\theta}_\bullet^{(\bullet)}$, B, and W, have the same definition as before to ensure that each chain has the same effect on the estimate. If the averages were weighted by size, a single long chain would dominate the statistics and defeat the purpose of monitoring convergence with multiple chains.

Because it contains the term N, the estimate $\widehat{var}^+$ must be generalized. By expanding the first term,

$$\frac{N-1}{N} W = \frac{N-1}{N} \frac{1}{M} \sum_{m=1}^{M} \frac{1}{N-1} \sum_{n=1}^{N} (\theta_m^{(n)} - \bar{\theta}_m^{(\bullet)})^2 = \frac{1}{M} \sum_{m=1}^{M} \frac{1}{N} \sum_{n=1}^{N} (\theta_m^{(n)} - \bar{\theta}_m^{(\bullet)})^2,$$

and the second term,

$$\frac{1}{N} B = \frac{1}{M-1} \sum_{m=1}^{M} (\bar{\theta}_m^{(\bullet)} - \bar{\theta}_\bullet^{(\bullet)})^2.$$

the variance estimator naturally generalizes to

$$\widehat{\text{var}}^+(\theta|y) = \frac{1}{M} \sum_{m=1}^{M} \frac{1}{N_m} \sum_{n=1}^{N_m} (\theta_m^{(n)} - \bar{\theta}_m^{(\bullet)})^2 + \frac{1}{M-1} \sum_{m=1}^{M} (\bar{\theta}_m^{(\bullet)} - \bar{\theta}_\bullet^{(\bullet)})^2.$$

If the chains are all the same length, this definition is equivalent to the one in the last section. This generalized variance estimator and the within-chains variance estimates may be plugged directly into the formula for $\hat{R}$ from the previous section.

Split $\hat{R}$ for Detecting Non-Stationarity

Before calculating the potential-scale-reduction statistic $\hat{R}$, each chain may be split into two halves. This provides an additional means to detect non-stationarity in the chains. If one chain involves gradually increasing values and one involves gradually decreasing values, they have not mixed well, but they can have $\hat{R}$ values near unity. In this case, splitting each chain into two parts leads to $\hat{R}$ values substantially greater than 1 because the first half of each chain has not mixed with the second half.

Converence is Global

A question that often arises is whether it is acceptable to monitor convergence of only a subset of the parameters or generated quantities. The short answer is "no," but this is elaborated further in this section.

For example, consider the value `lp__`, which is the log posterior density (up to a constant) It is a mistake to declare convergence in any practical sense if `lp__` has not converged, because different chains are really in different parts of the space. Yet measuring convergence for `lp__` is particularly tricky, as noted below.

Asymptotics and transience vs. equilibrium

Markov chain convergence is a global property in the sense that it does not depend on the choice of function of the parameters that is monitored. There is no hard cut-off between pre-convergence "transience" and post-convergence "equilibrium." What happens is that as the number of states in the chain approaches infinity, the distribution of possible states in the chain approaches the target distribution and in that limit the expected value of the Monte Carlo estimator of any integrable function converges to the true expectation. There is nothing like warmup here, because in the limit, the effects of initial state are completely washed out.

Multivariate convergence of functions

The $\hat{R}$ statistic considers the composition of a Markov chain and a function, and if the Markov chain has converged then each Markov chain and function composition will have converged. Multivariate functions converge when all of their margins have converged by the Cramer-Wold theorem.

The transformation from unconstrained space to constrained space is just another function, so does not effect convergence.

Different functions may have different autocorrelations, but if the Markov chain has equilibrated then all Markov chain plus function compositions should be consistent with convergence. Formally, any function that appears inconsistent is of concern and although it would be unreasonable to test every function, `lp__` and other measured quantities should at least be consistent.

The obvious difference in `lp__` is that it tends to vary quickly with position and is consequently susceptible to outliers.

Finite numbers of states

The question is what happens for finite numbers of states? If we can prove a strong geometric ergodicity property (which depends on the sampler and the target distribution), then one can show that there exists a finite time after which the chain forgets

its initial state with a large probability. This is both the autocorrelation time and the warmup time. But even if you can show it exists and is finite (which is nigh impossible) you can't compute an actual value analytically.

So what we do in practice is hope that the finite number of draws is large enough for the expectations to be reasonably accurate. Removing warmup iterations improves the accuracy of the expectations but there is no guarantee that removing any finite number of samples will be enough.

Why inconsistent $\hat{R}$?

There are two things to worry about here.

Firstly, as noted above, for any finite number of draws, there will always be some residual effect of the initial state, which typically manifests as some small (or large if the autocorrelation time is huge) probability of having a large outlier. Functions robust to such outliers (say, quantiles) will appear more stable and have better $\hat{R}$. Functions vulnerable to such outliers may show fragility.

Secondly, use of the $\hat{R}$ statistic makes very strong assumptions. In particular, it assumes that the functions being considered are Gaussian or it only uses the first two moments and assumes some kind of independence. The point is that strong assumptions are made that do not always hold. In particular, the distribution for the log posterior density (`lp__`) almost never looks Gaussian, instead it features long tails that can lead to large $\hat{R}$ even in the large N limit. Tweaks to $\hat{R}$, such as using quantiles in place of raw values, have the flavor of making the samples of interest more Gaussian and hence the $\hat{R}$ statistic more accurate.

Final words on convergence monitoring

"Convergence" is a global property and holds for all integrable functions at once, but employing the $\hat{R}$ statistic requires additional assumptions and thus may not work for all functions equally well.

Note that if you just compare the expectations between chains then we can rely on the Markov chain asymptotics for Gaussian distributions and can apply the standard tests.

52.4. Effective Sample Size

The second technical difficulty posed by MCMC methods is that the samples will typically be autocorrelated within a chain. This increases the uncertainty of the estimation of posterior quantities of interest, such as means, variances or quantiles.

Definition of Effective Sample Size

The amount by which autocorrelation within the chains increases uncertainty in estimates can be measured by effective sample size (ESS). Given independent samples, the central limit theorem bounds uncertainty in estimates based on the number of samples N. Given dependent samples, the number of independent samples is replaced with the effective sample size N_{eff}, which is the number of independent samples with the same estimation power as the N autocorrelated samples. For example, estimation error is proportional to $1/\sqrt{N_{\text{eff}}}$ rather than $1/\sqrt{N}$.

The effective sample size of a sequence is defined in terms of the autocorrelations within the sequence at different lags. The autocorrelation ρ_t at lag $t \geq 0$ for a chain with joint probability function $p(\theta)$ with mean μ and variance σ^2 is defined to be

$$\rho_t = \frac{1}{\sigma^2} \int_{\Theta} (\theta^{(n)} - \mu)(\theta^{(n+t)} - \mu)\, p(\theta)\, d\theta.$$

This is just the correlation between the two chains offset by t positions. Because we know $\theta^{(n)}$ and $\theta^{(n+t)}$ have the same marginal distribution in an MCMC setting, multiplying the two difference terms and reducing yields

$$\rho_t = \frac{1}{\sigma^2} \int_{\Theta} \theta^{(n)}\, \theta^{(n+t)}\, p(\theta)\, d\theta.$$

The effective sample size of N samples generated by a process with autocorrelations ρ_t is defined by

$$N_{\text{eff}} = \frac{N}{\sum_{t=-\infty}^{\infty} \rho_t} = \frac{N}{1 + 2\sum_{t=1}^{\infty} \rho_t}.$$

Estimation of Effective Sample Size

In practice, the probability function in question cannot be tractably integrated and thus the autocorrelation cannot be calculated, nor the effective sample size. Instead, these quantities must be estimated from the samples themselves. The rest of this section describes a variogram-based estimator for autocorrelations, and hence effective sample size, based on multiple chains. For simplicity, each chain θ_m will be assumed to be of length N.

One way to estimate the effective sample size is based on the variograms V_t at lag $t \in \{0, 1 \ldots\}$. The variograms are defined as follows for (univariate) samples $\theta_m^{(n)}$, where $m \in \{1, \ldots, M\}$ is the chain, and N_m is the number of samples in chain m.

$$V_t = \frac{1}{M} \sum_{m=1}^{M} \left(\frac{1}{N_m} \sum_{n=t+1}^{N_m} \left(\theta_m^{(n)} - \theta_m^{(n-t)} \right)^2 \right).$$

The variogram along with the multi-chain variance estimate $\widehat{\text{var}}^+$ introduced in the previous section can be used to estimate the autocorrelation at lag t as

$$\hat{\rho}_t = 1 - \frac{V_t}{2\,\widehat{\text{var}}^+}.$$

If the chains have not converged, the variance estimator $\widehat{\text{var}}^+$ will overestimate variance, leading to an overestimate of autocorrelation and an underestimate effective sample size.

Because of the noise in the correlation estimates $\hat{\rho}_t$ as t increases, typically only the initial estimates of $\hat{\rho}_t$ where $\hat{\rho}_t < 0$ will be used. Setting T' to be the first lag such that $\rho_{T'+1} < 0$,

$$T' = \arg\min_t \; \hat{\rho}_{t+1} < 0,$$

the effective sample size estimator is defined as

$$\hat{N}_{\text{eff}} = \frac{MN}{1 + \sum_{t=1}^{T'} \hat{\rho}_t}.$$

Thinning Samples

In the typical situation, the autocorrelation, ρ_t, decreases as the lag, t, increases. When this happens, thinning the samples will reduce the autocorrelation. For instance, consider generating one thousand samples in one of the following two ways.

1. Generate 1000 samples after convergence and save all of them.

2. Generate 10,000 samples after convergence and save every tenth sample.

Even though both produce one thousand samples, the second approach with thinning will produce more effective samples. That's because the autocorrelation ρ_t for the thinned sequence is equivalent to ρ_{10t} in the unthinned samples, so the sum of the autocorrelations will be lower and thus the effective sample size higher.

On the other hand, if memory and data storage are no object, saving all ten thousand samples will have a higher effective sample size than thinning to one thousand samples.

53. Transformations of Constrained Variables

To avoid having to deal with constraints while simulating the Hamiltonian dynamics during sampling, every (multivariate) parameter in a Stan model is transformed to an unconstrained variable behind the scenes by the model compiler. The transform is based on the constraints, if any, in the parameter's definition. Scalars or the scalar values in vectors, row vectors or matrices may be constrained with lower and/or upper bounds. Vectors may alternatively be constrained to be ordered, positive ordered, or simplexes. Matrices may be constrained to be correlation matrices or covariance matrices. This chapter provides a definition of the transforms used for each type of variable.

Stan converts models to C++ classes which define probability functions with support on all of $\mathbb{R}^K$, where K is the number of unconstrained parameters needed to define the constrained parameters defined in the program. The C++ classes also include code to transform the parameters from unconstrained to constrained and apply the appropriate Jacobians.

53.1. Changes of Variables

The support of a random variable X with density $p_X(x)$ is that subset of values for which it has non-zero density,

$$\text{supp}(X) = \{x | p_X(x) > 0\}.$$

If f is a total function defined on the support of X, then $Y = f(X)$ is a new random variable. This section shows how to compute the probability density function of Y for well-behaved transforms f. The rest of the chapter details the transforms used by Stan.

Univariate Changes of Variables

Suppose X is one dimensional and $f : \text{supp}(X) \to \mathbb{R}$ is a one-to-one, monotonic function with a differentiable inverse f^{-1}. Then the density of Y is given by

$$p_Y(y) = p_X(f^{-1}(y)) \left| \frac{d}{dy} f^{-1}(y) \right|.$$

The absolute derivative of the inverse transform measures how the scale of the transformed variable changes with respect to the underlying variable.

Multivariate Changes of Variables

The multivariate generalization of an absolute derivative is a Jacobian, or more fully the absolute value of the determinant of the Jacobian matrix of the transform. The Jacobian matrix measures the change of each output variable relative to every input variable and the absolute determinant uses that to determine the differential change in volume at a given point in the parameter space.

Suppose X is a K-dimensional random variable with probability density function $p_X(x)$. A new random variable $Y = f(X)$ may be defined by transforming X with a suitably well-behaved function f. It suffices for what follows to note that if f is one-to-one and its inverse f^{-1} has a well-defined Jacobian, then the density of Y is

$$p_Y(y) = p_X(f^{-1}(y)) \; \left| \det J_{f^{-1}}(y) \right|,$$

where det is the matrix determinant operation and $J_{f^{-1}}(y)$ is the Jacobian matrix of f^{-1} evaluated at y. Taking $x = f^{-1}(y)$, the Jacobian matrix is defined by

$$J_{f^{-1}}(y) = \begin{bmatrix} \frac{\partial x_1}{\partial y_1} & \cdots & \frac{\partial x_1}{\partial y_K} \\ \vdots & \vdots & \vdots \\ \frac{\partial x_K}{\partial y_1} & \cdots & \frac{\partial x_K}{\partial y_K} \end{bmatrix}.$$

If the Jacobian matrix is triangular, the determinant reduces to the product of the diagonal entries,

$$\det J_{f^{-1}}(y) = \prod_{k=1}^{K} \frac{\partial x_k}{\partial y_k}.$$

Triangular matrices naturally arise in situations where the variables are ordered, for instance by dimension, and each variable's transformed value depends on the previous variable's transformed values. Diagonal matrices, a simple form of triangular matrix, arise if each transformed variable only depends on a single untransformed variable.

53.2. Lower Bounded Scalar

Stan uses a logarithmic transform for lower and upper bounds.

Lower Bound Transform

If a variable X is declared to have lower bound a, it is transformed to an unbounded variable Y, where

$$Y = \log(X - a).$$

Lower Bound Inverse Transform

The inverse of the lower-bound transform maps an unbounded variable Y to a variable X that is bounded below by a by

$$X = \exp(Y) + a.$$

Absolute Derivative of the Lower Bound Inverse Transform

The absolute derivative of the inverse transform is

$$\left| \frac{d}{dy} \left(\exp(y) + a \right) \right| = \exp(y).$$

Therefore, given the density p_X of X, the density of Y is

$$p_Y(y) = p_X\big(\exp(y) + a\big) \cdot \exp(y).$$

53.3. Upper Bounded Scalar

Stan uses a negated logarithmic transform for upper bounds.

Upper Bound Transform

If a variable X is declared to have an upper bound b, it is transformed to the unbounded variable Y by

$$Y = \log(b - X).$$

Upper Bound Inverse Transform

The inverse of the upper bound transform converts the unbounded variable Y to the variable X bounded above by b through

$$X = b - \exp(Y).$$

Absolute Derivative of the Upper Bound Inverse Transform

The absolute derivative of the inverse of the upper bound transform is

$$\left| \frac{d}{dy} \left(b - \exp(y) \right) \right| = \exp(y).$$

Therefore, the density of the unconstrained variable Y is defined in terms of the density of the variable X with an upper bound of b by

$$p_Y(y) = p_X\big(b - \exp(y)\big) \cdot \exp(y).$$

53.4. Lower and Upper Bounded Scalar

For lower and upper-bounded variables, Stan uses a scaled and translated log-odds transform.

Log Odds and the Logistic Sigmoid

The log-odds function is defined for $u \in (0, 1)$ by

$$\text{logit}(u) = \log \frac{u}{1-u}.$$

The inverse of the log odds function is the logistic sigmoid, defined for $v \in (-\infty, \infty)$ by

$$\text{logit}^{-1}(v) = \frac{1}{1+\exp(-v)}.$$

The derivative of the logistic sigmoid is

$$\frac{d}{dy}\text{logit}^{-1}(y) = \text{logit}^{-1}(y) \cdot \left(1 - \text{logit}^{-1}(y)\right).$$

Lower and Upper Bounds Transform

For variables constrained to be in the open interval (a, b), Stan uses a scaled and translated log-odds transform. If variable X is declared to have lower bound a and upper bound b, then it is transformed to a new variable Y, where

$$Y = \text{logit}\left(\frac{X-a}{b-a}\right).$$

Lower and Upper Bounds Inverse Transform

The inverse of this transform is

$$X = a + (b-a) \cdot \text{logit}^{-1}(Y).$$

Absolute Derivative of the Lower and Upper Bounds Inverse Transform

The absolute derivative of the inverse transform is given by

$$\left| \frac{d}{dy}\left(a + (b-a) \cdot \text{logit}^{-1}(y)\right) \right| = (b-a) \cdot \text{logit}^{-1}(y) \cdot \left(1 - \text{logit}^{-1}(y)\right).$$

Therefore, the density of the transformed variable Y is

$$p_Y(y) = p_X\left(a + (b-a) \cdot \text{logit}^{-1}(y)\right) \cdot (b-a) \cdot \text{logit}^{-1}(y) \cdot \left(1 - \text{logit}^{-1}(y)\right).$$

Despite the apparent complexity of this expression, most of the terms are repeated and thus only need to be evaluated once. Most importantly, $\text{logit}^{-1}(y)$ only needs to be evaluated once, so there is only one call to $\exp(-y)$.

53.5. Ordered Vector

For some modeling tasks, a vector-valued random variable X is required with support on ordered sequences. One example is the set of cut points in ordered logistic regression (see Section 5.6).

In constraint terms, an ordered K-vector $x \in \mathbb{R}^K$ satisfies

$$x_k < x_{k+1}$$

for $k \in \{1, \ldots, K-1\}$.

Ordered Transform

Stan's transform follows the constraint directly. It maps an increasing vector $x \in \mathbb{R}^K$ to an unconstrained vector $y \in \mathbb{R}^K$ by setting

$$y_k = \begin{cases} x_1 & \text{if } k = 1, \text{ and} \\ \log\left(x_k - x_{k-1}\right) & \text{if } 1 < k \leq K. \end{cases}$$

Ordered Inverse Transform

The inverse transform for an unconstrained $y \in \mathbb{R}^K$ to an ordered sequence $x \in \mathbb{R}^K$ is defined by the recursion

$$x_k = \begin{cases} y_1 & \text{if } k = 1, \text{ and} \\ x_{k-1} + \exp(y_k) & \text{if } 1 < k \leq K. \end{cases}$$

x_k can also be expressed iteratively as

$$x_k = y_1 + \sum_{k'=2}^{k} \exp(y_{k'}).$$

Absolute Jacobian Determinant of the Ordered Inverse Transform

The Jacobian of the inverse transform f^{-1} is lower triangular, with diagonal elements for $1 \leq k \leq K$ of

$$J_{k,k} = \begin{cases} 1 & \text{if } k = 1, \text{ and} \\ \exp(y_k) & \text{if } 1 < k \leq K. \end{cases}$$

Because J is triangular, the absolute Jacobian determinant is

$$|\det J| = \left|\prod_{k=1}^{K} J_{k,k}\right| = \prod_{k=2}^{K} \exp(y_k).$$

Putting this all together, if p_X is the density of X, then the transformed variable Y has density p_Y given by

$$p_Y(y) = p_X(f^{-1}(y)) \prod_{k=2}^{K} \exp(y_k).$$

53.6. Unit Simplex

Variables constrained to the unit simplex show up in multivariate discrete models as both parameters (categorical and multinomial) and as variates generated by their priors (Dirichlet and multivariate logistic).

The unit K-simplex is the set of points $x \in \mathbb{R}^K$ such that for $1 \leq k \leq K$,

$$x_k > 0,$$

and

$$\sum_{k=1}^{K} x_k = 1.$$

An alternative definition is to take the convex closure of the vertices. For instance, in 2-dimensions, the simplex vertices are the extreme values $(0,1)$, and $(1,0)$ and the unit 2-simplex is the line connecting these two points; values such as $(0.3,0.7)$ and $(0.99,0.01)$ lie on the line. In 3-dimensions, the basis is $(0,0,1)$, $(0,1,0)$ and $(1,0,0)$ and the unit 3-simplex is the boundary and interior of the triangle with these vertices. Points in the 3-simplex include $(0.5,0.5,0)$, $(0.2,0.7,0.1)$ and all other triplets of non-negative values summing to 1.

As these examples illustrate, the simplex always picks out a subspace of $K-1$ dimensions from $\mathbb{R}^K$. Therefore a point x in the K-simplex is fully determined by its first $K-1$ elements $x_1, x_2, \ldots, x_{K-1}$, with

$$x_K = 1 - \sum_{k=1}^{K-1} x_k.$$

Unit Simplex Inverse Transform

Stan's unit simplex inverse transform may be understood using the following stick-breaking metaphor.[1]

[1]For an alternative derivation of the same transform using hyperspherical coordinates, see (Betancourt, 2010).

> Take a stick of unit length (i.e., length 1). Break a piece off and label it as x_1, and set it aside. Next, break a piece off what's left, label it x_2, and set it aside. Continue doing this until you have broken off $K-1$ pieces labeled $(x_1, \ldots, x_{K-1})$. Label what's left of the original stick x_K. The vector $x = (x_1, \ldots, x_K)$ is obviously a unit simplex because each piece has non-negative length and the sum of their lengths is 1.

This full inverse mapping requires the breaks to be represented as the fraction in $(0, 1)$ of the original stick that is broken off. These break ratios are themselves derived from unconstrained values in $(-\infty, \infty)$ using the inverse logit transform as described above for unidimensional variables with lower and upper bounds.

More formally, an intermediate vector $z \in \mathbb{R}^{K-1}$, whose coordinates z_k represent the proportion of the stick broken off in step k, is defined elementwise for $1 \leq k < K$ by

$$z_k = \mathrm{logit}^{-1}\left(y_k + \log\left(\frac{1}{K-k}\right)\right).$$

The logit term $\log\left(\frac{1}{K-k}\right)$ $(i.e., \mathrm{logit}\left(\frac{1}{K-k+1}\right))$ in the above definition adjusts the transform so that a zero vector y is mapped to the simplex $x = (1/K, \ldots, 1/K)$. For instance, if $y_1 = 0$, then $z_1 = 1/K$; if $y_2 = 0$, then $z_2 = 1/(K-1)$; and if $y_{K-1} = 0$, then $z_{K-1} = 1/2$.

The break proportions z are applied to determine the stick sizes and resulting value of x_k for $1 \leq k < K$ by

$$x_k = \left(1 - \sum_{k'=1}^{k-1} x_{k'}\right) z_k.$$

The summation term represents the length of the original stick left at stage k. This is multiplied by the break proportion z_k to yield x_k. Only $K-1$ unconstrained parameters are required, with the last dimension's value x_K set to the length of the remaining piece of the original stick,

$$x_K = 1 - \sum_{k=1}^{K-1} x_k.$$

Absolute Jacobian Determinant of the Unit-Simplex Inverse Transform

The Jacobian J of the inverse transform f^{-1} is lower-triangular, with diagonal entries

$$J_{k,k} = \frac{\partial x_k}{\partial y_k} = \frac{\partial x_k}{\partial z_k} \frac{\partial z_k}{\partial y_k},$$

where

$$\frac{\partial z_k}{\partial y_k} = \frac{\partial}{\partial y_k} \mathrm{logit}^{-1}\left(y_k + \log\left(\frac{1}{K-k}\right)\right) = z_k(1 - z_k),$$

and

$$\frac{\partial x_k}{\partial z_k} = \left(1 - \sum_{k'=1}^{k-1} x_{k'}\right).$$

This definition is recursive, defining x_k in terms of $x_1, \ldots, x_{k-1}$.

Because the Jacobian J of f^{-1} is lower triangular and positive, its absolute determinant reduces to

$$|\det J| = \prod_{k=1}^{K-1} J_{k,k} = \prod_{k=1}^{K-1} z_k (1 - z_k) \left(1 - \sum_{k'=1}^{k-1} x_{k'}\right).$$

Thus the transformed variable $Y = f(X)$ has a density given by

$$p_Y(y) = p_X(f^{-1}(y)) \prod_{k=1}^{K-1} z_k (1 - z_k) \left(1 - \sum_{k'=1}^{k-1} x_{k'}\right).$$

Even though it is expressed in terms of intermediate values z_k, this expression still looks more complex than it is. The exponential function need only be evaluated once for each unconstrained parameter y_k; everything else is just basic arithmetic that can be computed incrementally along with the transform.

Unit Simplex Transform

The transform $Y = f(X)$ can be derived by reversing the stages of the inverse transform. Working backwards, given the break proportions z, y is defined elementwise by

$$y_k = \operatorname{logit}(z_k) - \log\left(\frac{1}{K - k}\right).$$

The break proportions z_k are defined to be the ratio of x_k to the length of stick left after the first $k - 1$ pieces have been broken off,

$$z_k = \frac{x_k}{1 - \sum_{k'=1}^{k-1} x_{k'}}.$$

53.7. Unit Vector

Unit vectors show up in directional statistics.

The n-sphere is the set of points $x \in \mathbb{R}^n$ such that

$$\|x\|^2 = \sum_{i=1}^{n} x_i^2 = 1 .$$

Unit Vector Inverse Transform

To parametrize unit length vectors, we use hyperspherical coordinates. The unconstrained vector $y \in \mathbb{R}^{n-1}$ is a set of angles which relates to the unit vector as follows:

$$\begin{aligned}
x_1 &= \cos(y_1) \\
x_2 &= \cos(y_2)\sin(y_1) \\
x_3 &= \cos(y_3)\sin(y_1)\sin(y_2) \\
&\vdots \\
x_i &= \cos(y_i)\prod_{j=1}^{i-1}\sin(y_j) \\
&\vdots \\
x_n &= \prod_{j=1}^{n-1}\sin(y_j).
\end{aligned}$$

Note that, in practice, we use $y_i = \hat{y}_i + \frac{\pi}{2}$ and use $\hat{y}_i$ as the unconstrained parameters to avoid a singularity at $y_i = 0$.

Absolute Jacobian Determinant of the Unit Vector Inverse Transform

To derive the determinant of the Jacobian we first add a radius coordinate $r = 1$ which multiplies the vector x. The Jacobian, J, can be shown to be lower triangular, so its determinant is just the product of diagonal entries and the absolute value of the determinant is

$$|\det J| = \left| r^{n-1}\sin^{n-2}(y_1)\sin^{n-3}(y_2)...\sin(y_{n-2}) \right|.$$

53.8. Correlation Matrices

A $K \times K$ correlation matrix x must be is a symmetric, so that

$$x_{k,k'} = x_{k',k}$$

for all $k, k' \in \{1, \ldots, K\}$, it must have a unit diagonal, so that

$$x_{k,k} = 1$$

for all $k \in \{1, \ldots, K\}$, and it must be positive definite, so that for every non-zero K-vector a,

$$a^\top x a > 0.$$

To deal with this rather complicated constraint, Stan implements the transform of Lewandowski et al. (2009). The number of free parameters required to specify a $K \times K$ correlation matrix is $\binom{K}{2}$.

Correlation Matrix Inverse Transform

It is easiest to specify the inverse, going from its $\binom{K}{2}$ parameter basis to a correlation matrix. The basis will actually be broken down into two steps. To start, suppose y is a vector containing $\binom{K}{2}$ unconstrained values. These are first transformed via the bijective function $\tanh : \mathbb{R} \to (0, 1)$

$$\tanh x = \frac{\exp(2x) - 1}{\exp(2x) + 1}.$$

Then, define a $K \times K$ matrix z, the upper triangular values of which are filled by row with the transformed values. For example, in the 4×4 case, there are $\binom{4}{2}$ values arranged as

$$z = \begin{bmatrix} 0 & \tanh y_1 & \tanh y_2 & \tanh y_4 \\ 0 & 0 & \tanh y_3 & \tanh y_5 \\ 0 & 0 & 0 & \tanh y_6 \\ 0 & 0 & 0 & 0 \end{bmatrix}.$$

Lewandowski et al. show how to bijectively map the array z to a correlation matrix x. The entry $z_{i,j}$ for $i < j$ is interpreted as the canonical partial correlation (CPC) between i and j, which is the correlation between i's residuals and j's residuals when both i and j are regressed on all variables i' such that $i' < i$. In the case of $i = 1$, there are no earlier variables, so $z_{1,j}$ is just the Pearson correlation between i and j.

In Stan, the LKJ transform is reformulated in terms of a Cholesky factor w of the final correlation matrix, defined for $1 \leq i, j \leq K$ by

$$w_{i,j} = \begin{cases} 0 & \text{if } i > j, \\ 1 & \text{if } 1 = i = j, \\ \prod_{i'=1}^{i-1} \left(1 - z_{i',j}^2\right)^{1/2} & \text{if } 1 < i = j, \\ z_{i,j} & \text{if } 1 = i < j, \text{ and} \\ z_{i,j} \prod_{i'=1}^{i-1} \left(1 - z_{i',j}^2\right)^{1/2} & \text{if } 1 < i < j. \end{cases}$$

This does not require as much computation per matrix entry as it may appear; calcu-

lating the rows in terms of earlier rows yields the more manageable expression

$$w_{i,j} = \begin{cases} 0 & \text{if } i > j, \\ 1 & \text{if } 1 = i = j, \\ z_{i,j} & \text{if } 1 = i < j, \text{ and} \\ z_{i,j}\; w_{i-1,j} \left(1 - z_{i-1,j}^2\right)^{1/2} & \text{if } 1 < i \leq j. \end{cases}$$

Given the upper-triangular Cholesky factor w, the final correlation matrix is

$$x = w^\top w.$$

Lewandowski et al. show that the determinant of the correlation matrix can be defined in terms of the canonical partial correlations as

$$\det x = \prod_{i=1}^{K-1} \prod_{j=i+1}^{K} (1 - z_{i,j}^2) = \prod_{1 \leq i < j \leq K} (1 - z_{i,j}^2),$$

Absolute Jacobian Determinant of the Correlation Matrix Inverse Transform

From the inverse of equation 11 in Lewandowski et al. (2009), the absolute Jacobian determinant is

$$\sqrt{\prod_{i=1}^{K-1} \prod_{j=i+1}^{K} \left(1 - z_{i,j}^2\right)^{K-i-1}} \times \prod_{i=1}^{K-1} \prod_{j=i+1}^{K} \frac{\partial \tanh z_{i,j}}{\partial y_{i,j}}$$

Correlation Matrix Transform

The correlation transform is defined by reversing the steps of the inverse transform defined in the previous section.

Starting with a correlation matrix x, the first step is to find the unique upper triangular w such that $x = ww^\top$. Because x is positive definite, this can be done by applying the Cholesky decomposition,

$$w = \text{chol}(x).$$

The next step from the Cholesky factor w back to the array z of CPCs is simplified by the ordering of the elements in the definition of w, which when inverted yields

$$z_{i,j} = \begin{cases} 0 & \text{if } i \leq j, \\ w_{i,j} & \text{if } 1 = i < j, \text{ and} \\ w_{i,j}\; \prod_{i'=1}^{i-1} \left(1 - z_{i',j}^2\right)^{-2} & \text{if } 1 < i < j. \end{cases}$$

The final stage of the transform reverses the hyperbolic tangent transform, which is defined by

$$\tanh^{-1} v = \frac{1}{2} \log \left(\frac{1+v}{1-v} \right).$$

The inverse hyperbolic tangent function, $\tanh^{-1}$, is also called the Fisher transformation.

53.9. Covariance Matrices

A $K \times K$ matrix is a covariance matrix if it is symmetric and positive definite (see the previous section for definitions). It requires $K + \binom{K}{2}$ free parameters to specify a $K \times K$ covariance matrix.

Covariance Matrix Transform

Stan's covariance transform is based on a Cholesky decomposition composed with a log transform of the positive-constrained diagonal elements.[2]

If x is a covariance matrix (i.e., a symmetric, positive definite matrix), then there is a unique lower-triangular matrix $z = \text{chol}(x)$ with positive diagonal entries, called a Cholesky factor, such that

$$x = z\, z^{\top}.$$

The off-diagonal entries of the Cholesky factor z are unconstrained, but the diagonal entries $z_{k,k}$ must be positive for $1 \leq k \leq K$.

To complete the transform, the diagonal is log-transformed to produce a fully unconstrained lower-triangular matrix y defined by

$$y_{m,n} = \begin{cases} 0 & \text{if } m < n, \\ \log z_{m,m} & \text{if } m = n, \text{ and} \\ z_{m,n} & \text{if } m > n. \end{cases}$$

[2]An alternative to the transform in this section, which can be coded directly in Stan, is to parameterize a covariance matrix as a scaled correlation matrix. An arbitrary $K \times K$ covariance matrix Σ can be expressed in terms of a K-vector σ and correlation matrix Ω as

$$\Sigma = \text{diag}(\sigma) \times \Omega \times \text{diag}(\sigma),$$

so that each entry is just a deviation-scaled correlation,

$$\Sigma_{m,n} = \sigma_m \times \sigma_n \times \Omega_{m,n}.$$

Covariance Matrix Inverse Transform

The inverse transform reverses the two steps of the transform. Given an unconstrained lower-triangular $K \times K$ matrix y, the first step is to recover the intermediate matrix z by reversing the log transform,

$$z_{m,n} = \begin{cases} 0 & \text{if } m < n, \\ \exp(y_{m,m}) & \text{if } m = n, \text{ and} \\ y_{m,n} & \text{if } m > n. \end{cases}$$

The covariance matrix x is recovered from its Cholesky factor z by taking

$$x = z\, z^{\top}.$$

Absolute Jacobian Determinant of the Covariance Matrix Inverse Transform

The Jacobian is the product of the Jacobians of the exponential transform from the unconstrained lower-triangular matrix y to matrix z with positive diagonals and the product transform from the Cholesky factor z to x.

The transform from unconstrained y to Cholesky factor z has a diagonal Jacobian matrix, the absolute determinant of which is thus

$$\prod_{k=1}^{K} \frac{\partial}{\partial_{y_{k,k}}} \exp(y_{k,k}) \;=\; \prod_{k=1}^{K} \exp(y_{k,k}) \;=\; \prod_{k=1}^{K} z_{k,k}.$$

The Jacobian matrix of the second transform from the Cholesky factor z to the covariance matrix x is also triangular, with diagonal entries corresponding to pairs (m, n) with $m \geq n$, defined by

$$\frac{\partial}{\partial z_{m,n}} \left(z\, z^{\top}\right)_{m,n} \;=\; \frac{\partial}{\partial z_{m,n}} \left(\sum_{k=1}^{K} z_{m,k}\, z_{n,k} \right) \;=\; \begin{cases} 2\, z_{n,n} & \text{if } m = n \text{ and} \\ z_{n,n} & \text{if } m > n. \end{cases}$$

The absolute Jacobian determinant of the second transform is thus

$$2^{K} \prod_{m=1}^{K} \prod_{n=1}^{m} z_{n,n} \;=\; \prod_{n=1}^{K} \prod_{m=n}^{K} z_{n,n} \;=\; 2^{K} \prod_{k=1}^{K} z_{k,k}^{K-k+1}.$$

Finally, the full absolute Jacobian determinant of the inverse of the covariance matrix transform from the unconstrained lower-triangular y to a symmetric, positive definite matrix x is the product of the Jacobian determinants of the exponentiation and product transforms,

$$\left(\prod_{k=1}^{K} z_{k,k} \right) \left(2^{K} \prod_{k=1}^{K} z_{k,k}^{K-k+1} \right) \;=\; 2^{K} \prod_{k=1}^{K} z_{k,k}^{K-k+2}.$$

Let f^{-1} be the inverse transform from a $K + \binom{K}{2}$-vector y to the $K \times K$ covariance matrix x. A density function $p_X(x)$ defined on $K \times K$ covariance matrices is transformed to the density $p_Y(y)$ over $K + \binom{K}{2}$ vectors y by

$$p_Y(y) = p_X(f^{-1}(y))\ 2^K \prod_{k=1}^{K} z_{k,k}^{K-k+2}.$$

53.10. Cholesky Factors of Covariance Matrices

An $M \times M$ covariance matrix Σ can be Cholesky factored to a lower triangular matrix L such that $L L^\top = \Sigma$. If Σ is positive definite, then L will be $M \times M$. If Σ is only positive semi-definite, then L will be $M \times N$, with $N < M$.

A matrix is a Cholesky factor for a covariance matrix if and only if it is lower triangular, the diagonal entries are positive, and $M \geq N$. A matrix satisfying these conditions ensures that $L L^\top$ is positive semi-definite if $M > N$ and positive definite if $M = N$.

A Cholesky factor of a covariance matrix requires $N + \binom{N}{2} + (M - N)N$ unconstrained parameters.

Cholesky Factor of Covariance Matrix Transform

Stan's Cholesky factor transform only requires the first step of the covariance matrix transform, namely log transforming the positive diagonal elements. Suppose x is an $M \times N$ Cholesky factor. The above-diagonal entries are zero, the diagonal entries are positive, and the below-diagonal entries are unconstrained. The transform required is thus

$$y_{m,n} = \begin{cases} 0 & \text{if } m < n, \\ \log x_{m,m} & \text{if } m = n, \text{ and} \\ x_{m,n} & \text{if } m > n. \end{cases}$$

Cholesky Factor of Covariance Matrix Inverse Transform

The inverse transform need only invert the logarithm with an exponentiation. If y is the unconstrained matrix representation, then the elements of the constrained matrix x is defined by

$$x_{m,n} = \begin{cases} 0 & \text{if } m < n, \\ \exp(y_{m,m}) & \text{if } m = n, \text{ and} \\ y_{m,n} & \text{if } m > n. \end{cases}$$

Absolute Jacobian Determinant of Cholesky Factor Inverse Transform

The transform has a diagonal Jacobian matrix, the absolute determinant of which is

$$\prod_{n=1}^{N} \frac{\partial}{\partial_{y_{n,n}}} \exp(y_{n,n}) \;=\; \prod_{n=1}^{N} \exp(y_{n,n}) \;=\; \prod_{n=1}^{N} x_{n,n}.$$

Let $x = f^{-1}(y)$ be the inverse transform from a $N + \binom{N}{2} + (M-N)N$ vector to an $M \times N$ Cholesky factor for a covariance matrix x defined in the previous section. A density function $p_X(x)$ defined on $M \times N$ Cholesky factors of covariance matrices is transformed to the density $p_Y(y)$ over $N + \binom{N}{2} + (M-N)N$ vectors y by

$$p_Y(y) = p_X(f^{-1}(y)) \prod_{N=1}^{N} x_{n,n}.$$

53.11. Cholesky Factors of Correlation Matrices

A $K \times K$ correlation matrix Ω is positive definite and has a unit diagonal. Because it is positive definite, it can be Cholesky factored to a $K \times K$ lower-triangular matrix L with positive diagonal elements such that $\Omega = LL^\top$. Because the corelation matrix has a unit diagonal,

$$\Omega_{k,k} = L_k \, L_k^\top = 1,$$

each row vector L_k of the Cholesky factor is of unit length. The length and positivity constraint allow the diagonal elemens of L to be calculated from the off-diagonal elements, so that a Cholesky factor for a $K \times K$ correlation matrix requires only $\binom{K}{2}$ unconstrained parameters.

Cholesky Factor of Correlation Matrix Inverse Transform

It is easiest to start with the inverse transform from the $\binom{K}{2}$ unconstrained parameters y to the $K \times K$ lower-triangular Cholesky factor x. The inverse transform is based on the hyperbolic tangent function, tanh, which satisfies $\tanh(x) \in (-1, 1)$. Here it will function like an inverse logit with a sign to pick out the direction of an underlying canonical partial correlation (see Section 53.8 for more information on the relation between canonical partial correlations and the Cholesky factors of correlation matrices).

Suppose y is a vector of $\binom{K}{2}$ unconstrained values. Let z be a lower-triangular matrix with zero diagonal and below diagonal entries filled by row. For example, in

the 3×3 case,

$$z = \begin{bmatrix} 0 & 0 & 0 \\ \tanh y_1 & 0 & 0 \\ \tanh y_2 & \tanh y_3 & 0 \end{bmatrix}$$

The matrix z, with entries in the range $(-1, 1)$, is then transformed to the Cholesky factor x, by taking[3]

$$x_{i,j} = \begin{cases} 0 & \text{if } i < j \quad \text{[above diagonal]} \\[2ex] \sqrt{1 - \sum_{j' < j} x_{i,j'}^2} & \text{if } i = j \quad \text{[on diagonal]} \\[2ex] z_{i,j} \sqrt{1 - \sum_{j' < j} x_{i,j'}^2} & \text{if } i > j \quad \text{[below diagonal]} \end{cases}$$

In the 3×3 case, this yields

$$x = \begin{bmatrix} 1 & 0 & 0 \\ z_{2,1} & \sqrt{1 - x_{2,1}^2} & 0 \\ z_{3,1} & z_{3,2}\sqrt{1 - x_{3,1}^2} & \sqrt{1 - (x_{3,1}^2 + x_{3,2}^2)} \end{bmatrix},$$

where the $z_{i,j} \in (-1, 1)$ are the tanh-transformed y.

The approach is a signed stick-breaking process on the quadratic (Euclidean length) scale. Starting from length 1 at $j = 1$, each below-diagonal entry $x_{i,j}$ is determined by the (signed) fraction $z_{i,j}$ of the remaining length for the row that it consumes. The diagonal entries $x_{i,i}$ get any leftover length from earlier entries in their row. The above-diagonal entries are zero.

Cholesky Factor of Correlation Matrix Transform

Suppose x is a $K \times K$ Cholesky factor for some correlation matrix. The first step of the transform reconstructs the intermediate values z from x,

$$z_{i,j} = \frac{x_{i,j}}{\sqrt{1 - \sum_{j' < j} x_{i,j'}^2}}.$$

The mapping from the resulting z to y inverts tanh,

$$y = \tanh^{-1} z = \frac{1}{2} \left(\log(1 + z) - \log(1 - z) \right).$$

[3]For convenience, a summation with no terms, such as $\sum_{j' < 1} x_{i,j'}$, is defined to be 0. This implies $x_{1,1} = 1$ and that $x_{i,1} = z_{i,1}$ for $i > 1$.

Absolute Jacobian Determinant of Inverse Transform

The Jacobian of the full transform is the product of the Jacobians of its component transforms.

First, for the inverse transform $z = \tanh y$, the derivative is

$$\frac{d}{dy} \tanh y = \frac{1}{(\cosh y)^2}.$$

Second, for the inverse transform of z to x, the resulting Jacobian matrix J is of dimension $\binom{K}{2} \times \binom{K}{2}$, with indexes (i, j) for $(i > j)$. The Jacobian matrix is lower triangular, so that its determinant is the product of its diagonal entries, of which there is one for each (i, j) pair,

$$|\det J| = \prod_{i>j} \left| \frac{d}{dz_{i,j}} x_{i,j} \right|,$$

where

$$\frac{d}{dz_{i,j}} x_{i,j} = \sqrt{1 - \sum_{j'<j} x_{i,j'}^2}.$$

So the combined density for unconstrained y is

$$p_Y(y) = p_X(f^{-1}(y)) \prod_{n<\binom{K}{2}} \frac{1}{(\cosh y)^2} \prod_{i>j} \left(1 - \sum_{j'<j} x_{i,j'}^2\right)^{1/2},$$

where $x = f^{-1}(y)$ is used for notational convenience. The log Jacobian determinant of the complete inverse transform $x = f^{-1}(y)$ is given by

$$\log |\det J| = -2 \sum_{n \leq \binom{K}{2}} \log \cosh y + \frac{1}{2} \sum_{i>j} \log \left(1 - \sum_{j'<j} x_{i,j'}^2\right).$$

Part VIII

Algorithms & Implementations

54. Hamiltonian Monte Carlo Sampling

This part of the manual details the algorithm implementations used by Stan and how to configure them. This chapter presents the Hamiltonian Monte Carlo (HMC) algorithm and its adaptive variant the no-U-turn sampler (NUTS) along with details of their implementation and configuration in Stan; the next two chapters present Stan's optimizers and diagnostics.

54.1. Hamiltonian Monte Carlo

Hamiltonian Monte Carlo (HMC) is a Markov chain Monte Carlo (MCMC) method that uses the derivatives of the density function being sampled to generate efficient transitions spanning the posterior (see, e.g., (Betancourt and Girolami, 2013; Neal, 2011) for more details). It uses an approximate Hamiltonian dynamics simulation based on numerical integration which is then corrected by performing a Metropolis acceptance step.

This section translates the presentation of HMC by Betancourt and Girolami (2013) into the notation of Gelman et al. (2013).

Target Density

The goal of sampling is to draw from a density $p(\theta)$ for parameters θ. This is typically a Bayesian posterior $p(\theta|y)$ given data y, and in particular, a Bayesian posterior coded as a Stan program.

Auxiliary Momentum Variable

HMC introduces auxiliary momentum variables ρ and draws from a joint density

$$p(\rho, \theta) = p(\rho|\theta)p(\theta).$$

In most applications of HMC, including Stan, the auxiliary density is a multivariate normal that does not depend on the parameters θ,

$$\rho \sim \mathsf{MultiNormal}(0, \Sigma).$$

The covariance matrix Σ acts as a Euclidean metric to rotate and scale the target distribution; see (Betancourt and Stein, 2011) for details of the geometry.

In Stan, this matrix may be set to the identity matrix (i.e., unit diagonal) or estimated from warmup samples and optionally restricted to a diagonal matrix. The inverse Σ^{-1} is known as the mass matrix, and will be a unit, diagonal, or dense if Σ is.

The Hamiltonian

The joint desnity $p(\rho, \theta)$ defines a Hamiltonian

$$\begin{aligned} H(\rho, \theta) &= -\log p(\rho, \theta) \\ &= -\log p(\rho|\theta) - \log p(\theta). \\ &= T(\rho|\theta) + V(\theta), \end{aligned}$$

where the term

$$T(\rho|\theta) = -\log p(\rho|\theta)$$

is called the "kinetic energy" and the term

$$V(\theta) = -\log p(\theta)$$

is called the "potential energy." The potential energy is specified by the Stan program through its definition of a log density.

Generating Transitions

Starting from the current value of the parameters θ, a transition to a new state is generated in two stages before being subjected to a Metropolis accept step.

First, a value for the momentum is drawn independently of the current parameter values,

$$\rho \sim \mathsf{MultiNormal}(0, \Sigma).$$

Thus momentum does not persist across iterations.

Next, the joint system (θ, ρ) made up of the current parameter values θ and new momentum ρ is evolved via Hamilton's equations,

$$\begin{aligned} \frac{d\theta}{dt} &= +\frac{\partial H}{\partial \rho} &= +\frac{\partial T}{\partial \rho} \\ \frac{d\rho}{dt} &= -\frac{\partial H}{\partial \theta} &= -\frac{\partial T}{\partial \theta} - \frac{\partial V}{\partial \theta}. \end{aligned}$$

With the momentum density being independent of the target density, i.e., $p(\rho|\theta) = p(\rho)$, the first term in the momentum time derivative, $\partial T/\partial \theta$ is zero, yielding the pair time derivatives

$$\begin{aligned} \frac{d\theta}{dt} &= +\frac{\partial T}{\partial \rho} \\ \frac{d\rho}{dt} &= -\frac{\partial V}{\partial \theta}. \end{aligned}$$

Leapfrog Integrator

The last section leaves a two-state differential equation to solve. Stan, like most other HMC implementations, uses the leapfrog integrator, which is a numerical integration algorithm that's specifically adapted to provide stable results for Hamiltonian systems of equations.

Like most numerical integrators, the leapfrog algorithm takes discrete steps of some small time interval ϵ. The leapfrog algorithm begins by drawing a fresh momentum term independently of the parameter values θ or previous momentum value.

$$\rho \sim \mathsf{MultiNormal}(0, \Sigma).$$

It then alternates half-step updates of the momentum and full-step updates of the position.

$$\begin{aligned} \rho &\leftarrow \rho - \frac{\epsilon}{2} \frac{\partial V}{\partial \theta} \\ \theta &\leftarrow \theta + \epsilon \Sigma \rho \\ \rho &\leftarrow \rho - \frac{\epsilon}{2} \frac{\partial V}{\partial \theta}. \end{aligned}$$

By applying L leapfrog steps, a total of $L\,\epsilon$ time is simulated. The resulting state at the end of the simulation (L repetitions of the above three steps) will be denoted (ρ^*, θ^*).

The leapgrog integrator's error is on the order of ϵ^3 per step and ϵ^2 globally, where ϵ is the time interval (also known as the step size); Leimkuhler and Reich (2004) provide a detailed analysis of numerical integration for Hamiltonian systems, including a derivation of the error bound for the leapforg integrator.

Metropolis Accept Step

If the leapfrog integrator were perfect numerically, there would no need to do any more randomization per transition than generating a random momentum vector. Instead, what is done in practice to account for numerical errors during integration is to apply a Metropolis acceptance step, where the probability of keeping the proposal (ρ^*, θ^*) generated by transitioning from (ρ, θ) is

$$\min\left(1,\ \exp\left(H(\rho, \theta) - H(\rho^*, \theta^*)\right)\right).$$

If the proposal is not accepted, the previous parameter value is returned for the next draw and used to initialize the next iteration.

Algorithm Summary

The Hamiltonian Monte Carlo algorithm starts at a specified initial set of parameters θ; in Stan, this value is either user-specified or generated randomly. Then, for a given number of iterations, a new momentum vector is sampled and the current value of the parameter θ is updated using the leapfrog integrator with discretization time ϵ and number of steps L according to the Hamiltonian dynamics. Then a Metropolis acceptance step is applied, and a decision is made whether to update to the new state (θ^*, ρ^*) or keep the existing state.

54.2. HMC Algorithm Parameters

The Hamiltonian Monte Carlo algorithm has three parameters which must be set,

- discretization time ϵ,
- mass matrix Σ^{-1}, and
- number of steps taken L.

In practice, sampling efficiency, both in terms of iteration speed and iterations per effective sample, is highly sensitive to these three tuning parameters (Neal, 2011; Hoffman and Gelman, 2013).

If ϵ is too large, the leapfrog integrator will be inaccurate and too many proposals will be rejected. If ϵ is too small, too many small steps will be taken by the leapfrog integrator leading to long simulation times per interval. Thus the goal is to balance the acceptance rate between these extremes.

If L is too small, the trajectory traced out in each iteration will be too short and sampling will devolve to a random walk. If L is too large, the algorithm will do too much work on each iteration.

If the mass matrix Σ is poorly suited to the covariance of the posterior, the step size ϵ will have to be decreased to maintain arithmetic precision while at the same time, the number of steps L is increased in order to maintain simulation time to ensure statistical efficiency.

Automatic Parameter Tuning

Stan is able to automatically optimize ϵ to match an acceptance-rate target, able to estimate Σ based on warmup sample iterations, and able to dynamically adapt L on the fly during sampling (and during warmup) using the no-U-turn sampling (NUTS) algorithm (Hoffman and Gelman, 2013).

When adaptation is engaged (it may be turned off by fixing a step size and mass matrix), the warmup period is split into three stages, as illustrated in Figure 54.1,

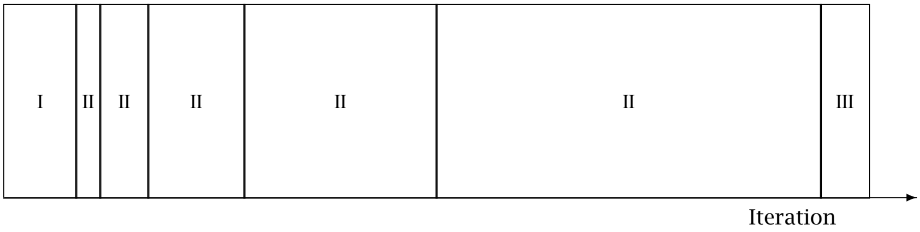

Figure 54.1: *Adaptation during warmup occurs in three stages: an initial fast adaptation interval (I), a series of expanding slow adaptation intervals (II), and a final fast adaptation interval (III). For HMC, both the fast and slow intervals are used for adapting the step size, while the slow intervals are used for learning the (co)variance necessitated by the metric. Iteration numbering starts at 1 on the left side of the figure and increases to the right.*

with two *fast* intervals surrounding a series of growing *slow* intervals. Here fast and slow refer to parameters that adapt using local and global information, respectively; the Hamiltonian Monte Carlo samplers, for example, define the step size as a fast parameter and the (co)variance as a slow parameter. The size of the the initial and final fast intervals and the initial size of the slow interval are all customizable, although user-specified values may be modified slightly in order to ensure alignment with the warmup period.

The motivation behind this partitioning of the warmup period is to allow for more robust adaptation. The stages are as follows.

I. In the initial fast interval the chain is allowed to converge towards the typical set,[1] with only parameters that can learn from local information adapted.

II. After this initial stage parameters that require global information, for example (co)variances, are estimated in a series of expanding, memoryless windows; often fast parameters will be adapted here as well.

III. Lastly, the fast parameters are allowed to adapt to the final update of the slow parameters.

These intervals may be controlled through the following configuration parameters, all of which must be positive integers:

parameter	*description*	*default*
initial buffer	width of initial fast adaptation interval	75
term buffer	width of final fast adaptation interval	50
window	initial width of slow adaptation interval	25

[1]The typical set is a concept borrowed from information theory and refers to the neighborhood (or neighborhoods in multimodal models) of substantial posterior probability mass through which the Markov chain will travel in equilibrium.

Discretization-Interval Adaptation Parameters

Stan's HMC algorithms utilize dual averaging (Nesterov, 2009) to optimize the step size.[2] This warmup optimization procedure is extremely flexible and for completeness, Stan exposes each tuning option for dual averaging, using the notation of Hoffman and Gelman (2013). In practice, the efficacy of the optimization is sensitive to the value of these parameters, but we do not recommend changing the defaults without experience with the dual-averaging algorithm. For more information, see the discussion of dual averaging in (Hoffman and Gelman, 2011, 2013).

The full set of dual-averaging parameters are

parameter	*description*	*constraint*	*default*
δ	target Metropolis acceptance rate	$\delta \in [0, 1]$	0.80
γ	adaptation regularization scale	$\gamma > 0$	0.05
κ	adaptation relaxation exponent	$\kappa > 0$	0.75
t_0	adaptation iteration offset	$t_0 > 0$	10

By setting the target acceptance parameter δ to a value closer to 1 (its value must be strictly less than 1 and its default value is 0.8), adaptation will be forced to use smaller step sizes. This can improve sampling efficiency (effective samples per iteration) at the cost of increased iteration times. Raising the value of δ will also allow some models that would otherwise get stuck to overcome their blockages.

Step-Size Jitter

All implementations of HMC use numerical integrators requiring a step size (equivalently, discretization time interval). Stan allows the step size to be adapted or set explicitly. Stan also allows the step size to be "jittered" randomly during sampling to avoid any poor interactions with a fixed step size and regions of high curvature. The jitter is a proportion that may be added or subtracted, so the maximum amount of jitter is 1, which will cause step sizes to be selected in the range of 0 to twice the adapted step size. The default value is 0, producing no jitter.

Small step sizes can get HMC samplers unstuck that would otherwise get stuck with higher step sizes. The downside is that jittering below the adapted value will increase the number of leapfrog steps required and thus slow down iterations, whereas jittering above the adapted value can cause premature rejection due to simulation error in the Hamiltonian dynamics calculation. See (Neal, 2011) for further discussion of step-size jittering.

[2]This optimization of step size during adaptation of the sampler should not be confused with running Stan's optimization method.

Euclidean Metric

All HMC implementations in Stan utilize quadratic kinetic energy functions which are specified up to the choice of a symmetric, positive-definite matrix known as a *mass matrix* or, more formally, a *metric* (Betancourt and Stein, 2011).

If the metric is constant then the resulting implementation is known as *Euclidean* HMC. Stan allows for three Euclidean HMC implementations,

- a unit metric (diagonal matrix of ones),
- a diagonal metric (diagonal matrix with positive diagonal entries), and
- a dense metric (a dense, symmetric positive definite matrix)

The user may configure the form of the metric.

If the mass matrix is specified to be diagonal, then regularized variances are estimated based on the iterations in each slow-stage block (labeled II in Figure 54.1). Each of these estimates is based only on the iterations in that block. This allows early estimates to be used to help guide warmup and then be forgotten later so that they do not influence the final covariance estimate.

If the mass matrix is specified to be dense, then regularized covariance estimates will be carried out, regularizing the estimate to a diagonal matrix, whihc is itself regularized toward a unit matrix.

Variances or covariances are estimated using Welford accumulators to avoid a loss of precision over many floating point operations.

NUTS and its Configuration

The no-U-turn sampler (NUTS) automatically selects an appropriate number of leapfrog steps in each iteration in order to allow the proposals to traverse the posterior without doing unnecessary work. The motivation is to maximize the expected squared jump distance (see, e.g., (Roberts et al., 1997)) at each step and avoid the random-walk behavior that arises in random-walk Metropolis or Gibbs samplers when there is correlation in the posterior. For a precise definition of the NUTS algorithm and a proof of detailed balance, see (Hoffman and Gelman, 2011, 2013).

NUTS generates a proposal by starting at an initial position determined by the parameters drawn in the last iteration. It then generates an independent unit-normal random momentum vector. It then evolves the initial system both forwards and backwards in time to form a balanced binary tree. At each iteration of the NUTS algorithm the tree depth is increased by one, doubling the number of leapfrog steps and effectively doubles the computation time. The algorithm terminates in one of two ways, either

- the NUTS criterion (i.e., a U-turn in Euclidean space on a subtree) is satisfied for a new subtree or the completed tree, or
- the depth of the completed tree hits the maximum depth allowed.

Rather than using a standard Metropolis step, the final parameter value is selected with slice sampling along the final evolution step (i.e., the second half of iterations generated).

Configuring the no-U-turn sample involves putting a cap on the depth of the trees that it evaluates during each iteration. This is controlled through a maximum depth parameter. The number of leapfrog steps taken is then bounded by 2 to the power of the maximum depth minus 1.

Both the tree depth and the actual number of leapfrog steps computed are reported along with the parameters in the output as `treedepth__` and `n_leapfrog__`, respectively. Because the final subtree may only be partially constructed, these two will always satisfy

$$2^{\text{treedepth}-1} - 1 < N_{\text{leapfrog}} \le 2^{\text{treedepth}} - 1.$$

Tree depth is an important diagnostic tool for NUTS. For example, a tree depth of zero occurs when the first leapfrog step is immediately rejected and the initial state returned, indicating extreme curvature and poorly-chosen step size (at least relative to the current position). On the other hand, a tree depth equal to the maximum depth indicates that NUTS is taking many leapfrog steps and being terminated prematurely to avoid excessively long execution time. Taking very many steps may be a sign of poor adaptation, may be due to targeting a very high acceptance rate, or may simply indicate a difficult posterior from which to sample. In the latter case, reparameterization may help with efficiency. But in the rare cases where the model is correctly specified and a large number of steps is necessary, the maximum depth should be increased to ensure that that the NUTS tree can grow as large as necessary.

Sampling without Parameters

In some situations, such as pure forward data simulation in a directed graphical model (e.g., where you can work down generatively from known hyperpriors to simulate parameters and data), there is no need to declare any parameters in Stan, the model block will be empty, and all output quantities will be produced in the generated quantities block. In this situation, the sampler must be configured to use the fixed-parameters setting. Without parameters there is no need for adaptation and the number of warmup iterations should be set to zero.

54.3. General Configuration Options

Stan's interfaces provide a number of configuration options that are shared among the MCMC algorithms (this chapter), the optimization algorithms (Chapter 55), and the diagnostics (Chapter 56).

Random Number Generator

The random-number generator's behavior is fully determined by the unsigned seed (positive integer) it is started with. If a seed is not specified, or a seed of 0 or less is specified, the system time is used to generate a seed. The seed is recorded and included with Stan's output regardless of whether it was specified or generated randomly from the system time.

Stan also allows a chain identifier to be specified, which is useful when running multiple Markov chains for sampling. The chain identifier is used to advance the random number generator a very large number of random variates so that two chains with different identifiers draw from non-overlapping subsequences of the random-number sequence determined by the seed. When running multiple chains from a single command, Stan's interfaces will manage the chain identifiers.

Replication

Together, the seed and chain identifier determine the behavior of the underlying random number generator. See Chapter 20 for a list of requirements for replication.

Initialization

The initial parameter values for Stan's algorithms (MCMC, optimization, or diagnostic) may be either specified by the user or generated randomly. If user-specified values are provided, all parameters must be given initial values or Stan will abort with an error message.

User-Defined Initialization

If the user specifies initial values, they must satisfy the constraints declared in the model (i.e., they are on the constrained scale).

System Constant Zero Initialization

It is also possible to provide an initialization of 0, which causes all variables to be initialized with zero values on the unconstrained scale. The transforms are arranged in such a way that zero initialization provides reasonable variable initializations for

most parameters, such as 0 for unconstrained parameters, 1 for parameters constrained to be positive, 0.5 for variables to constrained to lie between 0 and 1, a symmetric (uniform) vector for simplexes, unit matrices for both correlation and covariance matrices, and so on. See Chapter 53 for full details of the transformations.

System Random Initialization

Random initialization by default initializes the parameter values with values drawn at random from a $\text{Uniform}(-2, 2)$ distribution. Alternatively, a value other than 2 may be specified for the absolute bounds. These values are on the unconstrained scale, and are inverse transformed back to satisfy the constraints declared for parameters. See Chapter 53 for a complete description of the transforms used.

Because zero is chosen to be a reasonable default initial value for most parameters, the interval around zero provides a fairly diffuse starting point. For instance, unconstrained variables are initialized randomly in $(-2, 2)$, variables constrained to be positive are initialized rougly in $(0.14, 7.4)$, variables constrained to fall between 0 and 1 are initialized with values roughly in $(0.12, 0.88)$.

55. Optimization Algorithms

Stan provides optimization algorithms which find modes of the density specified by a Stan program. Such modes may be used as parameter estimates or as the basis of approximations to a Bayesian posterior; see Chapter 50 for background on point estimation.

Stan provides three different optimizers, a Newton optimizer, and two related quasi-Newton algorithms, BFGS and L-BFGS; see (Nocedal and Wright, 2006) for thorough description and analysis of all of these algorithms. The L-BFGS algorithm is the default optimizer. Newton's method is the least efficient of the three, but has the advantage of setting its own stepsize.

55.1. General Configuration

All of the optimizers are iterative and allow the maximum number of iterations to be specified; the default maximum number of iterations is 2000.

All of the optimizers are able to stream intermediate output reporting on their progress. Whether or not to save the intermediate iterations and stream progress is configurable.

55.2. BFGS and L-BFGS Configuration

Convergence Monitoring

Convergence monitoring in (L-)BFGS is controlled by a number of tolerance values, any one of which being satisified causes the algorithm to terminate with a solution. Any of the convergence tests can be disabled by setting its corresponding tolerance parameter to zero. The tests for convergence are as follows.

Parameter Convergence

The parameters θ_i in iteration i are considered to have converged with respect to tolerance `tol_param` if

$$||\theta_i - \theta_{i-1}|| < \texttt{tol\_param}.$$

Density Convergence

The (unnormalized) log density $\log p(\theta_i|y)$ for the parameters θ_i in iteration i given data y is considered to have converged with respect to tolerance `tol_obj` if

$$\left|\log p(\theta_i|y) - \log p(\theta_{i-1}|y)\right| < \texttt{tol\_obj}.$$

The log density is considered to have converged to within relative tolerance `tol_rel_obj` if

$$\frac{|\log p(\theta_i|y) - \log p(\theta_{i-1}|y)|}{\max(|\log p(\theta_i|y)|, |\log p(\theta_{i-1}|y)|, 1.0)} < \texttt{tol\_rel\_obj} * \epsilon.$$

Gradient Convergence

The gradient is considered to have converged to 0 relative to a specified tolerance `tol_grad` if

$$||g_i|| < \texttt{tol\_grad},$$

where ∇_θ is the gradient operator with respect to θ and $g_i = \nabla_\theta \log p(\theta_i|y)$ is the gradient at iteration i.

The gradient is considered to have converged to 0 relative to a specified relative tolerance `tol_rel_grad` if

$$\frac{g_i^T \hat{H}_i^{-1} g_i}{\max(|\log p(\theta_i|y)|, 1.0)} < \texttt{tol\_rel\_grad} * \epsilon,$$

where $\hat{H}_i$ is the estimate of the Hessian at iteration i, $|u|$ is the absolute value (L1 norm) of u, $||u||$ is the vector length (L2 norm) of u, and $\epsilon \approx 2e - 16$ is machine precision.

Initial Step Size

The initial step size parameter α for BFGS-style optimizers may be specified. If the first iteration takes a long time (and requires a lot of function evaluations) initialize α to be the roughly equal to the α used in that first iteration. The default value is intentionally small, 0.001, which is reasonable for many problems but might be too large or too small depending on the objective function and initialization. Being too big or too small just means that the first iteration will take longer (i.e., require more gradient evaluations) before the line search finds a good step length. It's not a critical parameter, but for optimizing the same model multiple times (as you tweak things or with different data), being able to tune α can save some real time.

L-BFGS History Size

L-BFGS has a command-line argument which controls the size of the history it uses to approximate the Hessian. The value should be less than the dimensionality of the parameter space and, in general, relatively small values (5–10) are sufficient; the default value is 5.

If L-BFGS performs poorly but BFGS performs well, consider increasing the history size. Increasing history size will increase the memory usage, although this is unlikely to be an issue for typical Stan models.

55.3. General Configuration Options

The general configuration options for optimization are the same as those for MCMC; see Section 54.3 for details.

56. Diagnostic Mode

Stan's diagnostic mode runs a Stan program with data, initializing parameters either randomly or with user-specified initial values, and then evaluates the log probability and its gradients. The gradients computed by the Stan program are compared to values calculated by finite differences.

Diagnostic mode may be configured with two parameters.

parameter	*description*	*constraints*	*default*
ϵ	finite difference size	$\epsilon > 0$	1e–6
error	error threshold for matching	error > 0	1e–6

If the difference between the Stan program's gradient value and that calculated by finite difference is higher than the specified threshold, the argument will be flagged.

56.1. Output

Diagnostic mode prints the log posterior density (up to a proportion) calculated by the Stan program for the specified initial values. For each parameter, it prints the gradient at the initial parameter values calculated by Stan's program and by finite differences over Stan's program for the log probability.

Unconstrained Scale

The output is for the variable values and their gradients are on the unconstrained scale, which means each variable is a vector of size corresponding to the number of unconstrained variables required to define it. For example, an $N \times N$ correlation matrix, requires $\binom{N}{2}$ unconstrained parameters. The transformations from constrained to unconstrained parameters are based on the constraints in the parameter declarations and described in detail in Chapter 53.

Includes Jacobian

The log density includes the Jacobian adjustment implied by the constraints declared on variables; see Chapter 53 for full details. The Jacobian adjustment will be turned off if optimization is used in practice, but there is as of yet no way to turn it off in diagnostic mode.

56.2. Configuration Options

The general configuration options for diagnostics are the same as those for MCMC; see Section 54.3 for details. Initial values may be specified, or they may be drawn at

random. Setting the random number generator will only have an effect if a random initialization is specified.

56.3. Speed Warning and Data Trimming

Due to the application of finite differences, the computation time grows linearly with the number of parameters. This can be require a very long time, especially in models with latent parameters that grow with the data size. It can be helpful to diagnose a model with smaller data sizes in such cases.

Part IX

Contributed Modules

57. Contributed Modules

Stan is an open-source project and welcomes user contributions.

In order to reduce maintenance on the main trunk of Stan development and to allow developer-specified licenses, contributed Stan modules are not distributed as part of Stan itself.

57.1. Contributing a Stan Module

Developers who have a Stan module to contribute should contact the Stan developers either through one of the following.

- `stan-users` mailing list:
  https://groups.google.com/forum/?fromgroups#!forum/stan-users
- Stan e-mail:
  mailto:stan@mc-stan.org

57.2. GitHub-Hosted Modules

The `stan-dev` organization on GitHub hosts contributed projects on GitHub. This is also where the Stan developers will host works in progress. The full list of contributed projects on GitHub for `stan-dev` is available at the following location.

https://github.com/stan-dev

Each contributed module on `stan-dev`'s GitHub space comes with its own documentation, indexed by the `README.md` file displayed on GitHub. Each contributed module has its own licensing the terms of which are controlled by its developers. The license for a contributed package and its dependencies can be found in a top-level directory `licenses/`.

Emacs Stan Mode

Emacs Stan mode allows syntax highlighting and automatic indentation of Stan models in the Emacs text editor.

Repository: https://github.com/stan-dev/stan-mode

License: GPLv3

Authors: Jeffrey Arnold, Daniel Lee

Appendices

A. Licensing

Stan and its two dependent libraries, Boost and Eigen, are distributed under liberal freedom-respecting[1] licenses approved by the Open Source Initiative.[2]

In particular, the licenses for Stan and its dependent libraries have no "copyleft" provisions requiring applications of Stan to be open source if they are redistributed.

This chapter describes the licenses for the tools that are distributed with Stan. The next chapter explains some of the build tools that are not distributed with Stan, but are required to build and run Stan models.

A.1. Stan's License

Stan is distributed under the BSD 3-clause license (BSD New).

> http://www.opensource.org/licenses/BSD-3-Clause

The copyright holder of each contribution is the developer or his or her assignee.[3]

A.2. Boost License

Boost is distributed under the Boost Software License version 1.0.

> http://www.opensource.org/licenses/BSL-1.0

The copyright for each Boost package is held by its developers or their assginees.

A.3. Eigen License

Eigen is distributed under the Mozilla Public License, version 2.

> http://opensource.org/licenses/mpl-2.0

The copyright of Eigen is owned jointly by its developers or their assignees.

---

[1]The link http://www.gnu.org/philosophy/open-source-misses-the-point.html leads to a discussion about terms "open source" and "freedom respecting."

[2]See http://opensource.org.

[3]Universities or companies often own the copyright of computer programs developed by their employees.

A.4. Google Test License

Stan uses Google Test for unit testing; it is not required to compile or execute models. Google Test is distributed under the BSD 2-clause license.

> `http://www.opensource.org/licenses/BSD-License`

B. Stan for Users of BUGS

From the outside, Stan and BUGS[1] are similar — they use statistically-themed modeling languages (which are similar but with some differences; see below), they can be called from R, running some specified number of chains to some specified length, producing posterior simulations that can be assessed using standard convergence diagnostics. This is not a coincidence: in designing Stan, we wanted to keep many of the useful features of Bugs.

To start, take a look at the files of translated BUGS models at `http://mc-stan.org/`. These are 40 or so models from the BUGS example volumes, all translated and tested (to provide the same answers as BUGS) in Stan. For any particular model you want to fit, you can look for similar structures in these examples.

B.1. Some Differences in How BUGS and Stan Work

- BUGS is interpreted; Stan is compiled in two steps, first a model is is translated to templated C++ and then to a platform-specific executable. Stan, unlike BUGS, allows the user to directly program in C++, but we do not describe how to do this in this Stan manual (see the getting started with C++ section of `http://mc-stan.org` for more information on using Stan directly from C++).

- BUGS performs MCMC updating one scalar parameter at a time (with some exceptions such as JAGS's implementation of regression and generalized linear models and some conjugate multivariate parameters), using conditional distributions (Gibbs sampling) where possible and otherwise using adaptive rejection sampling, slice sampling, and Metropolis jumping. BUGS figures out the dependence structure of the joint distribution as specified in its modeling language and uses this information to compute only what it needs at each step. Stan moves in the entire space of all the parameters using Hamiltonian Monte Carlo (more precisely, the no-U-turn sampler), thus avoiding some difficulties that occur with one-dimension-at-a-time sampling in high dimensions but at the cost of requiring the computation of the entire log density at each step.

- BUGS tunes its adaptive jumping (if necessary) during its warmup phase (traditionally referred to as "burn-in"). Stan uses its warmup phase to tune the no-U-turn sampler (NUTS).

- The BUGS modeling language is not directly executable. Rather, BUGS parses its model to determine the posterior density and then decides on a sampling

[1] Except where otherwise noted, we use "BUGS" to refer to WinBUGS, OpenBUGS, and JAGS, indiscriminately.

scheme. In contrast, the statements in a Stan model are directly executable: they translate exactly into C++ code that is used to compute the log posterior density (which in turn is used to compute the gradient).

- In BUGS, the order in which statements are written does not matter. They are executed according to the directed graphical model so that variables are always defined when needed. A side effect of the direct execution of Stan's modeling language is that statements execute in the order in which they are written. For instance, the following Stan program, which sets mu before using it to sample y.

  ```
  mu <- a + b * x;
  y ~ normal(mu,sigma);
  ```

  It translates to the following C++ code.

  ```
  mu = a + b * x;
  lp += normal_log(mu,sigma);
  ```

  Contrast this with the Stan program

  ```
  y ~ normal(mu,sigma)
  mu <- a + b * x
  ```

  This program is well formed, but is almost certainly a coding error, because it attempts to use mu before it is set. It translates to the following C++ code.

  ```
  lp += normal_log(mu,sigma);
  mu = a + b * x;
  ```

  The direct translation to the imperative language of C++ code highlights the potential error of using mu in the first statement.

  To trap these kinds of errors, variables are initialized to the special not-a-number (NaN) value. If NaN is passed to a log probability function, it will raise a domain exception, which will in turn be reported by the sampler. The sampler will reject the sample out of hand as if it had zero probability.

- Stan uses its own C++ algorithmic differentiation packages to compute the gradient of the log density (up to a proportion). Gradients are required during the Hamiltonian dynamics simulations within the leapfrog algorithm of the Hamiltonian Monte Carlo and NUTS samplers. BUGS computes the log density but not its gradient.

- Both BUGS and Stan are semi-automatic in that they run by themselves with no outside tuning required. Nevertheless, the user needs to pick the number

of chains and number of iterations per chain. We usually pick 4 chains and start with 10 iterations per chain (to make sure there are no major bugs and to approximately check the timing), then go to 100, 1000, or more iterations as necessary. Compared to Gibbs or Metropolis, Hamiltonian Monte Carlo can take longer per iteration (as it typically takes many "leapfrog steps" within each iteration), but the iterations typically have lower autocorrelation. So Stan might work fine with 1000 iterations in an example where BUGS would require 100,000 for good mixing. We recommend monitoring potential scale reduction statistics ($\hat{R}$) and the effective sample size to judge when to stop (stopping when $\hat{R}$ values do not counter-indicate convergence and when enough effective samples have been collected).

- WinBUGS is closed source. OpenBUGS and JAGS are both licensed under the Gnu Public License (GPL), otherwise known as copyleft due to the restrictions it places on derivative works. Stan is licensed under the much more liberal new BSD license.
- Like WinBUGS, OpenBUGS and JAGS, Stan can be run directly from the command line or through R (Python and MATLAB interfaces are in the works)
- Like OpenBUGS and JAGS, Stan can be run on Linux, Mac, and Windows platforms.

B.2. Some Differences in the Modeling Languages

- The BUGS modeling language follows an R-like syntax in which line breaks are meaningful. Stan follows the rules of C, in which line breaks are equivalent to spaces, and each statement ends in a semicolon. For example:

  ```
  y ~ normal(mu, sigma);
  ```

  and

  ```
  for (i in 1:n) y[i] ~ normal(mu, sigma);
  ```

  Or, equivalently (recall that a line break is just another form of whitespace),

  ```
  for (i in 1:n)
    y[i] ~ normal(mu, sigma);
  ```

  and also equivalently,

  ```
  for (i in 1:n) {
    y[i] ~ normal(mu, sigma);
  }
  ```

There's a semicolon after the model statement but not after the brackets indicating the body of the for loop.

- Another C thing: In Stan, variables can have names constructed using letters, numbers, and the underscore (_) symbol, but nothing else (and a variable name cannot begin with a number). BUGS variables can also include the dot, or period (`.`) symbol.

- In Stan, the second argument to the "normal" function is the standard deviation (i.e., the scale), not the variance (as in *Bayesian Data Analysis*) and not the inverse-variance (i.e., precision) (as in BUGS). Thus a normal with mean 1 and standard deviation 2 is `normal(1,2)`, not `normal(1,4)` or `normal(1,0.25)`.

- Similarly, the second argument to the "multivariate normal" function is the covariance matrix and not the inverse covariance matrix (i.e., the precision matrix) (as in BUGS). The same is true for the "multivariate student" distribution.

- The distributions have slightly different names:

BUGS	*Stan*
`dnorm`	`normal`
`dbinom`	`binomial`
`dpois`	`poisson`
⋮	⋮

- Stan, unlike BUGS, allows intermediate quantities, in the form of local variables, to be reassigned. For example, the following is legal and meaningful (if possibly inefficient) Stan code.

```
{
  total <- 0;
  for (i in 1:n){
    theta[i] ~ normal(total, sigma);
    total <- total + theta[i];
  }
}
```

In BUGS, the above model would not be legal because the variable `total` is defined more than once. But in Stan, the loop is executed in order, so `total` is overwritten in each step.

- Stan uses explicit declarations. Variables are declared with base type integer or real, and vectors, matrices, and arrays have specified dimensions. When variables are bounded, we give that information also. For data and transformed

parameters, the bounds are used for error checking. For parameters, the constraints are critical to sampling as they determine the geometry over which the Hamiltonian is simulated.

Variables can be declared as data, transformed data, parameters, transformed parameters, or generated quantities. They can also be declared as local variables within blocks. For more information, see the part of this manual devoted to the Stan programming language and examine at the example models.

- Stan allows all sorts of tricks with vector and matrix operations which can make Stan models more compact. For example, arguments to probability functions may be vectorized,[2] allowing

```
for (i in 1:n)
  y[i] ~ normal(mu[i], sigma[i]);
```

  to be expressed more compactly as

```
y ~ normal(mu,sigma);
```

  The vectorized form is also more efficient because Stan can unfold the computation of the chain rule during algorithmic differentiation.

- Stan also allows for arrays of vectors and matrices. For example, in a hierarchical model might have a vector of `K` parameters for each of `J` groups; this can be declared using

```
vector[K] theta[J];
```

  Then `theta[j]` is an expression denoting a `K`-vector and may be used in the code just like any other vector variable.

  An alternative encoding would be with a two-dimensional array, as in

```
real theta[J,K];
```

  The vector version can have some advantages, both in convenience and in computational speed for some operations.

  A third encoding would use a matrix:

```
matrix[J,K] theta;
```

[2]Most distributions have been vectorized, but currently the truncated versions may not exist and may not be vectorized.

but in this case, `theta[j]` is a row vector, not a vector, and accessing it as a vector is less efficient than with an array of vectors. The transposition operator, as in `theta[j]'`, may be used to convert the row vector `theta[j]` to a (column) vector. Column vector and row vector types are not interchangeable everywhere in Stan; see the function signature declarations in the programming language section of this manual.

- Stan supports general conditional statements using a standard if-else syntax. For example, a zero-inflated (or -deflated) Poisson mixture model is defined using the if-else syntax as described in Section 9.4.

- Stan supports general while loops using a standard syntax. While loops give Stan full Turing equivalent computational power. They are useful for defining iterative functions with complex termination conditions. As an illustration of their syntax, the for-loop

```
model {
    ....
    for (n in 1:N) {
        ... do something with n ....
    }
}
```

may be recoded using the following while loop.

```
model {
    int n;
    ...
    n <- 1;
    while (n <= N) {
        ... do something with n ...
        n <- n + 1;
    }
}
```

B.3. Some Differences in the Statistical Models that are Allowed

- Stan does not yet support estimation of discrete parameters (discrete data is supported). We plan to eventually implement a combination of Gibbs and slice sampling for discrete parameters, but we haven't done so yet.

- Stan has some distributions on covariance matrices that do not exist in BUGS, including a uniform distribution over correlation matrices which may be rescaled,

and the priors based on C-vines defined in (Lewandowski et al., 2009). In particular, the Lewandowski et al. prior allows the correlation matrix to be shrunk toward the unit matrix while the scales are given independent priors.

- In BUGS you need to define all variables. In Stan, if you declare but don't define a parameter it implicitly has a flat prior (on the scale in which the parameter is defined). For example, if you have a parameter `p` declared as

```
real<lower=0,upper=1> p;
```

and then have no sampling statement for `p` in the `model` block, then you are implicitly assigning a uniform $[0, 1]$ prior on `p`. On the other hand, if you have a parameter `theta` declared with

```
real theta;
```

and have no sampling statement for `theta` in the `model` block, then you are implicitly assigning an improper uniform prior on $(-\infty, \infty)$ to `theta`.

- BUGS models are always proper (being constructed as a product of proper marginal and conditional densities). Stan models can be improper. Here is the simplest improper Stan model:

```
parameters {
  real theta;
}
model { }
```

- Although parameters in Stan models may have improper priors, we do not want improper *posterior* distributions, as we are trying to use these distributions for Bayesian inference. There is no general way to check if a posterior distribution is improper. But if all the priors are proper, the posterior will be proper also.

- As noted earlier, each statement in a Stan model is directly translated into the C++ code for computing the log posterior. Thus, for example, the following pair of statements is legal in a Stan model:

```
y ~ normal(0,1);
y ~ normal(2,3);
```

The second line here does *not* simply overwrite the first; rather, *both* statements contribute to the density function that is evaluated. The above two lines have the effect of including the product, $\text{Norm}(y|0, 1) \times \text{Norm}(y|2, 3)$, into the density function.

For a perhaps more confusing example, consider the following two lines in a Stan model:

```
x ~ normal(0.8*y, sigma);
y ~ normal(0.8*x, sigma);
```

At first, this might look like a joint normal distribution with a correlation of 0.8. But it is not. The above are *not* interpreted as conditional entities; rather, they are factors in the joint density. Multiplying them gives, $\mathsf{Norm}(x|0.8y, \sigma) \times \mathsf{Norm}(y|0.8x, \sigma)$, which is what it is (you can work out the algebra) but it is not the joint distribution where the conditionals have regressions with slope 0.8.

- With censoring and truncation, Stan uses the censored-data or truncated-data likelihood—this is not always done in BUGS. All of the approaches to censoring and truncation discussed in (Gelman et al., 2013) and (Gelman and Hill, 2007) may be implemented in Stan directly as written.

- Stan, like BUGS, can benefit from human intervention in the form of reparameterization. More on this topic to come.

B.4. Some Differences when Running from R

- Stan can be set up from within R using two lines of code. Follow the instructions for running Stan from R on `http://mc-stan.org/`. You don't need to separately download Stan and RStan. Installing RStan will automatically set up Stan. When RStan moves to CRAN, it will get even easier.

- In practice we typically run the same Stan model repeatedly. If you pass RStan the result of a previously fitted model the model will not need be recompiled. An example is given on the running Stan from R pages available from `http://mc-stan.org/`.

- When you run Stan, it saves various conditions including starting values, some control variables for the tuning and running of the no-U-turn sampler, and the initial random seed. You can specify these values in the Stan call and thus achieve exact replication if desired. (This can be useful for debugging.)

- When running BUGS from R, you need to send exactly the data that the model needs. When running RStan, you can include extra data, which can be helpful when playing around with models. For example, if you remove a variable `x` from the model, you can keep it in the data sent from R, thus allowing you to quickly alter the Stan model without having to also change the calling information in your R script.

- As in R2WinBUGS and R2jags, after running the Stan model, you can quickly summarize using `plot()` and `print()`. You can access the simulations themselves using various extractor functions, as described in the RStan documentation.

- Various information about the sampler, such as number of leapfrog steps, log probability, and step size, is available through extractor functions. These can be useful for understanding what is going wrong when the algorithm is slow to converge.

B.5. The Stan Community

- Stan, like WinBUGS, OpenBUGS, and JAGS, has an active community, which you can access via the user's mailing list and the developer's mailing list; see `http://mc-stan.org/` for information on subscribing and posting and to look at archives.

C. Stan Program Style Guide

This appendix describes the preferred style for laying out Stan models. These are not rules of the language, but simply recommendations for laying out programs in a text editor. Although these recommendations may seem arbitrary, they are similar to those of many teams for many programming languages. Like rules for typesetting text, the goal is to achieve readability without wasting white space either vertically or horizontally.

C.1. Choose a Consistent Style

The most important point of style is consistency. Consistent coding style makes it easier to read not only a single program, but multiple programs. So when departing from this style guide, the number one recommendation is to do so consistently.

C.2. Line Length

Line lengths should not exceed 80 characters.[1] This is a typical recommendation for many programming language style guides because it makes it easier to lay out text edit windows side by side and to view the code on the web without wrapping, easier to view diffs from version control, etc. About the only thing that is sacrificed is laying out expressions on a single line.

C.3. File Extensions

The recommended file extension for Stan model files is `.stan`. For Stan data dump files, the recommended extension is `.R`, or more informatively, `.data.R`.

C.4. Variable Naming

The recommended variable naming is to follow C/C++ naming conventions, in which variables are lowercase, with the underscore character (`_`) used as a separator. Thus it is preferred to use `sigma_y`, rather than the run together `sigmay`, camel-case `sigmaY`, or capitalized camel-case `SigmaY`. Even matrix variables should be lowercased.

[1] Even 80 characters may be too many for rendering in print; for instance, in this manual, the number of code characters that fit on a line is about 65.

The exception to the lowercasing recommendation, which also follows the C/C++ conventions, is for size constants, for which the recommended form is a single uppercase letter. The reason for this is that it allows the loop variables to match. So loops over the indices of an $M \times N$ matrix a would look as follows.

```
for (m in 1:M)
  for (n in 1:N)
     a[m,n] = ...
```

C.5. Local Variable Scope

Declaring local variables in the block in which they are used aids in understanding programs because it cuts down on the amount of text scanning or memory required to reunite the declaration and definition.

The following Stan program corresponds to a direct translation of a BUGS model, which uses a different element of mu in each iteration.

```
model {
  real mu[N];
  for (n in 1:N) {
    mu[n] <- alpha * x[n] + beta;
    y[n] ~ normal(mu[n],sigma);
  }
}
```

Because variables can be reused in Stan and because they should be declared locally for clarity, this model should be recoded as follows.

```
model {
  for (n in 1:N) {
    real mu;
    mu <- alpha * x[n] + beta;
    y[n] ~ normal(mu,sigma);
  }
}
```

The local variable can be eliminated altogether, as follows.

```
model {
  for (n in 1:N)
    y[n] ~ normal(alpha * x[n] + beta, sigma);
}
```

There is unlikely to be any measurable efficiency difference between the last two implementations, but both should be a bit more efficient than the BUGS translation.

Scope of Compound Structures with Componentwise Assignment

In the case of local variables for compound structures, such as arrays, vectors, or matrices, if they are built up component by component rather than in large chunks, it can be more efficient to declare a local variable for the structure outside of the block in which it is used. This allows it to be allocated once and then reused.

```
model {
  vector[K] mu;
  for (n in 1:N) {
    for (k in 1:K)
      mu[k] <- ...;
    y[n] ~ multi_normal(mu,Sigma);
}
```

In this case, the vector mu will be allocated outside of both loops, and used a total of N times.

C.6. Parentheses and Brackets

Optional Parentheses for Single-Statement Blocks

Single-statement blocks can be rendered in one of two ways. The fully explicit bracketed way is as follows.

```
for (n in 1:N) {
  y[n] ~ normal(mu,1);
}
```

The following statement without brackets has the same effect.

```
for (n in 1:N)
  y[n] ~ normal(mu,1);
```

Single-statement blocks can also be written on a single line, as in the following example.

```
for (n in 1:N) y[n] ~ normal(mu,1);
```

These can be much harder to read than the first example. Only use this style if the statement is very simple, as in this example. Unless there are many similar cases, it's almost always clearer to put each sampling statement on its own line.

Conditional and looping statements may also be written without brackets.

The use of for loops without brackets can be dangerous. For instance, consider this program.

```
for (n in 1:N)
  z[n] ~ normal(nu,1);
  y[n] ~ normal(mu,1);
```

Because Stan ignores whitespace and the parser completes a statement as eagerly as possible (just as in C++), the previous program is equivalent to the following program.

```
for (n in 1:N) {
  z[n] ~ normal(nu,1);
}
y[n] ~ normal(mu,1);
```

Parentheses in Nested Operator Expressions

The preferred style for operators minimizes parentheses. This reduces clutter in code that can actually make it harder to read expressions. For example, the expression `a + b * c` is preferred to the equivalent `a + (b * c)` or `(a + (b * c))`. The operator precedences and associativities are given in Figure 23.1.

Similarly, comparison operators can usually be written with minimal bracketing, with the form `y[n] > 0 || x[n] != 0` preferred to the bracketed form `(y[n] > 0) || (x[n] != 0)`.

No Open Brackets on Own Line

Vertical space is valuable as it controls how much of a program you can see. The preferred Stan style is as shown in the previous section, not as follows.

```
for (n in 1:N)
{
  y[n] ~ normal(mu,1);
}
```

This also goes for parameters blocks, transformed data blocks, which should look as follows.

```
transformed parameters {
  real sigma;
  ...
}
```

C.7. Conditionals

Stan supports the full C++-style conditional syntax, allowing real or integer values to act as conditions, as follows.

```
real x;
...
if (x) {
   // executes if x not equal to 0
   ...
}
```

Explicit Comparisons of Non-Boolean Conditions

The preferred form is to write the condition out explicitly for integer or real values that are not produced as the result of a comparison or boolean operation, as follows.

```
if (x != 0) ...
```

C.8. Functions

Functions are laid out the same way as in languages such as Java and C++. For example,

```
real foo(real x, real y) {
  return sqrt(x * log(y));
}
```

The return type is flush left, the parentheses for the arguments are adjacent to the arguments and function name, and there is a space after the comma for arguments after the first. The open curly brace for the body is on the same line as the function name, following the layout of loops and conditionals. The body itself is indented; here we use two spaces. The close curly brace appears on its own line. If function names or argument lists are long, they can be written as

```
matrix
function_to_do_some_hairy_algebra(matrix thingamabob,
                                  vector doohickey2) {
  ...body...
}
```

The function starts a new line, under the type. The arguments are aligned under each other.

Function documentation should follow the Javadoc and Doxygen styles. Here's an example repeated from Section 16.7.

```
/**
 * Return a data matrix of specified size with rows
```

```
 * corresponding to items and the first column filled
 * with the value 1 to represent the intercept and the
 * remaining columns randomly filled with unit-normal draws.
 *
 * @param N Number of rows correspond to data items
 * @param K Number of predictors, counting the intercept, per
 *          item.
 * @return Simulated predictor matrix.
 */
matrix predictors_rng(int N, int K) {
  ...
```

The open comment is `/**`, asterisks are aligned below the first asterisk of the open comment, and the end comment `*/` is also aligned on the asterisk. The tags `@param` and `@return` are used to label function arguments (i.e., parameters) and return values.

C.9. White Space

Stan allows spaces between elements of a program. The white space characters allowed in Stan programs include the space (ASCII `0x20`), line feed (ASCII `0x0A`), carriage return (`0x0D`), and tab (`0x09`). Stan treats all whitespace characters interchangeably, with any sequence of whitespace characters being syntactically equivalent to a single space character. Nevertheless, effective use of whitespace is the key to good program layout.

Line Breaks Between Statements and Declarations

It is dispreferred to have multiple statements or declarations on the same line, as in the following example.

```
transformed parameters {
  real mu_centered;  real sigma;
  mu <- (mu_raw - mean_mu_raw);    sigma <- pow(tau,-2);
}
```

These should be broken into four separate lines.

No Tabs

Stan programs should not contain tab characters. They are legal and may be used anywhere other whitespace occurs. Using tabs to layout a program is highly unportable

because the number of spaces represented by a single tab character varies depending on which program is doing the rendering and how it is configured.

Two-Character Indents

Stan has standardized on two space characters of indentation, which is the standard convention for C/C++ code. Another sensible choice is four spaces, which is the convention for Java and Python. Just be consistent.

Space Between `if` and Condition

Use a space after `if`s. For instance, use `if (x < y) ...`, not `if(x < y) ...`.

No Space For Function Calls

There is no space between a function name and the function it applies to. For instance, use `normal(0,1)`, not `normal (0,1)`.

Spaces Around Operators

There should be spaces around binary operators. For instance, use `y[1] <- x`, not `y[1]<-x`, use `(x + y) * z` not `(x+y)*z`.

Breaking Expressions across Lines

Sometimes expressions are too long to fit on a single line. In that case, the recommended form is to break *before* an operator,[2] aligning the operator to indicate scoping. For example, use the following form (though not the content; inverting matrices is almost always a bad idea).

```
increment_log_prob((y - mu)' * inv(Sigma) * (y - mu));
```

Here, the multiplication operator (*) is aligned to clearly signal the multiplicands in the product.

For function arguments, break after a comma and line the next argument up underneath as follows.

```
y[n] ~ normal(alpha + beta * x + gamma * y,
              pow(tau,-0.5));
```

[2]This is the usual convention in both typesetting and other programming languages. Neither R nor BUGS allows breaks before an operator because they allow newlines to signal the end of an expression or statement.

Optional Spaces after Commas

Optionally use spaces after commas in function arguments for clarity. For example, `normal(alpha * x[n] + beta,sigma)` can also be written as `normal(alpha * x[n] + beta, sigma)`.

Unix Newlines

Wherever possible, Stan programs should use a single line feed character to separate lines. All of the Stan developers (so far, at least) work on Unix-like operating systems and using a standard newline makes the programs easier for us to read and share.

Platform Specificity of Newlines

Newlines are signaled in Unix-like operating systems such as Linux and Mac OS X with a single line-feed (LF) character (ASCII code point `0x0A`). Newlines are signaled in Windows using two characters, a carriage return (CR) character (ASCII code point `0x0D`) followed by a line-feed (LF) character.

D. Warning and Error Messages

This appendix details the specific error messages returned by the underlying Stan engine. The individual Stan interfaces (RStan, PyStan, CmdStan) also return error or warning messages in some circumstances.

D.1. Warnings vs. Errors

The messages returned by Stan come in two flavors, warnings and errors. Error messages arise when a fatal problem occurs under the hood from which there is no way to recover. Warning messages arise in circumstances from which the underlying program can continue to operaate.

An example warning message is an informational message about ill-formed input to an underlying function, which results in a Metropolis rejection during sampling or a reduction in step size for optimization, without causing a fatal error. An example error message is when either sampling or optimization cannot find a region of non-zero probability or when a program being parsed is ill formed. When an error arises, whatever program is running cannot continue and the entire execution must halt.

D.2. Parsing and Compilation

Both warning messages and error messages may arise during parsing. If a Stan program parses successfully, it should compile in C++ as well. If it does not compile, there is an underlying bug in Stan's parser and C++ code generator.

- *Jacobian may be necessary.* This message arises when the parser cannot verify that the left-hand side of a sampling statement is a linear function of a parameter. If it is not a linear function of the parameters, a Jacobian adjustment must be applied. See Chapter 14 for more information on how to adjust for the Jacobian of a non-linear transform

D.3. Initialization

- *vanishing density.* This message arrises when there is a problem with initialization not providing a finite log probability.

D.4. Function Evaluation

- *informational message.* This message shows up during sampling when there is a rejected sample due to an underlying issue with input to an underlying func-

tion (including probably functions and sampling statements). This is a *warning* message, not an error message. If it only appears at the beginning of warmup in MCMC or during early iterations of optimization, it indicates that adaptation has not yet found appropriate scales for the parameters and an appropriate overall stepsize. This causes problem like overflow or underflow and thus causes illegal inputs to be passed to functions. Even if this message persists during sampling, the Metropolis acceptance step will account for the problem and the parameter values being evaluated will be rejected. This can lead to inefficiency in the best case and lack of ability to make progress in the worst case. In cases where the message persists, it is worth investigating the arithmetic stability of the Stan program. There are several tips in this manual and in the user group about how to rewrite problematic programs.

E. Mathematical Functions

This appendix provides the definition of several mathematical functions used throughout the manual.

E.1. Beta

The beta function, $\mathrm{B}(\alpha, \beta)$, computes the normalizing constant for the beta distribution, and is defined for $a > 0$ and $b > 0$ by

$$\mathrm{B}(a, b) \;=\; \int_0^1 u^{a-1}(1-u)^{b-1}\, du \;=\; \frac{\Gamma(a)\,\Gamma(b)}{\Gamma(a+b)}.$$

E.2. Gamma

The gamma function, $\Gamma(x)$, is the generalization of the factorial function to continuous variables, defined so that for positive integers n,

$$\Gamma(n+1) = n!$$

Generalizing to all positive numbers and non-integer negative numbers,

$$\Gamma(x) = \int_0^\infty u^{x-1} \exp(-u)\, du.$$

E.3. Digamma

The digamma function Ψ is the derivative of the $\log \Gamma$ function,

$$\Psi(u) \;=\; \frac{d}{du} \log \Gamma(u) \;=\; \frac{1}{\Gamma(u)}\, \frac{d}{du} \Gamma(u).$$

Bibliography

Aguilar, O. and West, M. (2000). Bayesian dynamic factor models and portfolio allocation. *Journal of Business & Economic Statistics*, 18(3):338–357. 79

Betancourt, M. (2010). Cruising the simplex: Hamiltonian Monte Carlo and the Dirichlet distribution. *arXiv*, 1010.3436. 418

Betancourt, M. (2012). A general metric for Riemannian manifold Hamiltonian Monte Carlo. *arXiv*, 1212.4693. 182

Betancourt, M. and Girolami, M. (2013). Hamiltonian Monte Carlo for hierarchical models. *arXiv*, 1312.0906. 185, 431

Betancourt, M. and Stein, L. C. (2011). The geometry of Hamiltonian Monte Carlo. *arXiv*, 1112.4118. 431, 437

Blei, D. M. and Lafferty, J. D. (2007). A correlated topic model of *Science*. *The Annals of Applied Statistics*, 1(1):17–37. 125

Blei, D. M., Ng, A. Y., and Jordan, M. I. (2003). Latent Dirichlet allocation. *Journal of Machine Learning Research*, 3:993–1022. 123

Bowling, S. R., Khasawneh, M. T., Kaewkuekool, S., and Cho, B. R. (2009). A logistic approximation to the cumulative normal distribution. *Journal of Industrial Engineering and Management*, 2(1):114–127. 304

Chung, Y., Rabe-Hesketh, S., Dorie, V., Gelman, A., and Liu, J. (2013). A nondegenerate penalized likelihood estimator for variance parameters in multilevel models. *Psychometrika*, 78(4):685–709. 36, 47

Clayton, D. G. (1992). Models for the analysis of cohort and case-control studies with inaccurately measured exposures. In Dwyer, J. H., Feinleib, M., Lippert, P., and Hoffmeister, H., editors, *Statistical Models for Longitudinal Studies of Exposure and Health*, pages 301–331. Oxford University Press. 91, 92

Cook, S. R., Gelman, A., and Rubin, D. B. (2006). Validation of software for Bayesian models using posterior quantiles. *Journal of Computational and Graphical Statistics*, 15(3):675–692. 22

Cormack, R. M. (1964). Estimates of survival from the sighting of marked animals. *Biometrika*, 51(3/4):429–438. 102

Curtis, S. M. (2010). BUGS code for item response theory. *Journal of Statistical Software*, 36(1):1–34. 47

Daumé, III, H. (2007). HBC: Hierarchical Bayes compiler. Technical report, University of Utah. vi

Dawid, A. P. and Skene, A. M. (1979). Maximum likelihood estimation of observer error-rates using the EM algorithm. *Journal of the Royal Statistical Society. Series C (Applied Statistics)*, 28(1):20–28. 110, 112, 113, 114

Dempster, A. P., Laird, N. M., and Rubin, D. B. (1977). Maximum likelihood from incomplete data via the EM algorithm. *Journal of the Royal Statistical Society. Series B (Methodological)*, 39(1):1–38. 96

Duane, A., Kennedy, A., Pendleton, B., and Roweth, D. (1987). Hybrid Monte Carlo. *Physics Letters B*, 195(2):216–222. 16

Durbin, J. and Koopman, S. J. (2001). *Time Series Analysis by State Space Methods*. Oxford University Press, New York. 382

Efron, B. (2012). *Large-Scale Inference: Empirical Bayes Methods for Estimation, Testing, and Prediction*. Institute of Mathematical Statistics Monographs. Cambridge Univesity Press. 396

Efron, B. and Morris, C. (1975). Data analysis using stein's estimator and its generalizations. *Journal of the American Statistical Association*, 70:311–319. 396

Engle, R. F. (1982). Autoregressive conditional heteroscedasticity with estimates of variance of United Kingdom inflation. *Econometrica*, 50:987–1008. 61

Fonnesbeck, C., Patil, A., Huard, D., and Salvatier, J. (2013). *PyMC User's Guide*. Version 2.3. 96

Fowler, M., Beck, K., Brant, J., Opdyke, W., and Roberts, D. (1999). *Refactoring: Improving the Design of Existing Code*. Addison-Wesley. ix

Gay, D. M. (2005). Semiautomatic differentiation for efficient gradient computations. In Bücker, H. M., Corliss, G. F., Hovland, P., Naumann, U., and Norris, B., editors, *Automatic Differentiation: Applications, Theory, and Implementations*, volume 50 of *Lecture Notes in Computational Science and Engineering*, pages 147–158. Springer, New York. vii

Gelman, A. (2006). Prior distributions for variance parameters in hierarchical models. *Bayesian Analysis*, 1(3):515–534. 36, 37, 39

Gelman, A., Carlin, J. B., Stern, H. S., Dunson, D. B., Vehtari, A., and Rubin, D. B. (2013). *Bayesian Data Analysis*. Chapman &Hall/CRC Press, London, third edition. 77, 93, 95, 122, 141, 234, 347, 401, 403, 431, 458

Gelman, A. and Hill, J. (2007). *Data Analysis Using Regression and Multilevel-Hierarchical Models*. Cambridge University Press, Cambridge, United Kingdom. vi, 47, 48, 49, 52, 122, 269, 458

Gelman, A., Jakulin, A., Pittau, M. G., and Su, Y.-S. (2008). A weakly informative default prior distribution for logistic and other regression models. *Annals of Applied Statistics*, 2(4):1360–1383. 36, 39

Gelman, A. and Rubin, D. B. (1992). Inference from iterative simulation using multiple sequences. *Statistical Science*, 7(4):457–472. 17, 407

Giesler, G. C. (2000). MCNP software quality: Then and now. Technical Report LA-UR-00-2532, Los Alamos National Laboratory. xii

Girolami, M. and Calderhead, B. (2011). Riemann manifold Langevin and Hamiltonian Monte Carlo methods. *Journal of the Royal Statistical Society: Series B (Statistical Methodology)*, 73(2):123–214. 182

Google (2011). Google C++ testing framework. `http://code.google.com/p/googletest/`.

Guennebaud, G., Jacob, B., et al. (2010). Eigen v3. `http://eigen.tuxfamily.org`.

Hastie, T., Tibshirani, R., and Friedman, J. (2009). *The Elements of Statistical Learning: Data Mining, Inference, and Prediction*. Springer-Verlag, New York, second edition. 400

Hastings, W. K. (1970). Monte Carlo sampling methods using Markov chains and their applications. *Biometrika*, 57(1):97–109. 16

Hoerl, A. E. and Kennard, R. W. (1970). Ridge regression: biased estimation for nonorthogonal problems. *Technometrics*, 12(1):55–67. 394

Hoffman, M. D. and Gelman, A. (2011). The no-U-turn sampler: Adaptively setting path lengths in Hamiltonian Monte Carlo. *arXiv*, 1111.4246. viii, 17, 69, 176, 205, 436, 437

Hoffman, M. D. and Gelman, A. (2013). The No-U-Turn Sampler: Adaptively Setting Path Lengths in Hamiltonian Monte Carlo. *Journal of Machine Learning Research*, in press. viii, 17, 69, 176, 205, 434, 436, 437

Hunt, A. and Thomas, D. (1999). *The Pragmatic Programmer*. Addison-Wesley. 20

James, W. and Stein, C. (1961). Estimation with quadratic loss. In Neyman, J., editor, *Proceedings of the Fourth Berkeley Symposium on Mathematical Statistics and Probability*, volume 1, pages 361–379. University of California Press. 396

Jarrett, R. G. (1979). A note on the intervals between coal-mining disasters. *Biometrika*, 66(1):191–193. 96

Jolly, G. M. (1965). Explicit estimates from capture-recapture data with both death and immigration-stochastic model. *Biometrika*, 52(1/2):225–247. 102

Kim, S., Shephard, N., and Chib, S. (1998). Stochastic volatility: Likelihood inference and comparison with ARCH models. *Review of Economic Studies*, 65:361–393. 67

Lambert, D. (1992). Zero-inflated Poisson regression, with an application to defects in manufacturing. *Technometrics*, 34(1). 88

Leimkuhler, B. and Reich, S. (2004). *Simulating Hamiltonian Dynamics*. Cambridge University Press. 433

Lewandowski, D., Kurowicka, D., and Joe, H. (2009). Generating random correlation matrices based on vines and extended onion method. *Journal of Multivariate Analysis*, 100:1989–2001. 385, 422, 423, 457

Lincoln, F. C. (1930). Calculating waterfowl abundance on the basis of banding returns. *United States Department of Agriculture Circular*, 118:1–4. 101

Lunn, D., Jackson, C., Best, N., Thomas, A., and Spiegelhalter, D. (2012). *The BUGS Book: A Practical Introduction to Bayesian Analysis*. CRC Press/Chapman & Hall. 36, 37, 38

Lunn, D. J., Wakefield, J., Thomas, A., Best, N., and Spiegelhalter, D. (1999). *PKBugs User Guide*. Dept. Epidemiology and Public Health, Imperial College School of Medicine, London. 235

McConnell, S. (2004). *Code Complete: A Practical Handbook of Software Construction*. Microsoft Press, second edition. 20

Metropolis, N., Rosenbluth, A., Rosenbluth, M., Teller, M., and Teller, E. (1953). Equations of state calculations by fast computing machines. *Journal of Chemical Physics*, 21:1087–1092. 16, 406

Metropolis, N. and Ulam, S. (1949). The Monte Carlo method. *Journal of the American Statistical Association*, 44(247):335–341. xii, 405

Neal, R. (2011). MCMC using Hamiltonian dynamics. In Brooks, S., Gelman, A., Jones, G. L., and Meng, X.-L., editors, *Handbook of Markov Chain Monte Carlo*, pages 116–162. Chapman and Hall/CRC. 16, 17, 431, 434, 436

Neal, R. M. (1994). An improved acceptance procedure for the hybrid monte carlo algorithm. *Journal of Computational Physics*, 111:194–203. 16

Neal, R. M. (1996a). *Bayesian Learning for Neural Networks*. Number 118 in Lecture Notes in Statistics. Springer. 134

Neal, R. M. (1996b). Sampling from multimodal distributions using tempered transitions. *Statistics and Computing*, 6(4):353–366. 173

Neal, R. M. (1997). Monte Carlo implementation of Gaussian process models for Bayesian regression and classification. Technical Report 9702, University of Toronto, Department of Statistics. 133

Neal, R. M. (2003). Slice sampling. *Annals of Statistics*, 31(3):705–767. 180

Nesterov, Y. (2009). Primal-dual subgradient methods for convex problems. *Mathematical Programming*, 120(1):221–259. 17, 436

Nocedal, J. and Wright, S. J. (2006). *Numerical Optimization*. Springer-Verlag, Berlin, second edition. 441

Papaspiliopoulos, O., Roberts, G. O., and Sköld, M. (2007). A general framework for the parametrization of hierarchical models. *Statistical Science*, 22(1):59–73. 180

Petersen, C. G. J. (1896). The yearly immigration of young plaice into the Limfjord from the German Sea. *Report of the Danish Biological Station*, 6:5–84. 101

Pinheiro, J. C. and Bates, D. M. (1996). Unconstrained parameterizations for variance-covariance matrices. *Statistics and Computing*, 6:289–296.

Rasmussen, C. E. and Williams, C. K. I. (2006). *Gaussian Processes for Machine Learning*. MIT Press. 128

Richardson, S. and Gilks, W. R. (1993). A Bayesian approach to measurement error problems in epidemiology using conditional independence models. *American Journal of Epidemiology*, 138(6):430–442. 91

Roberts, G., Gelman, A., and Gilks, W. R. (1997). Weak convergence and optimal scaling of random walk Metropolis algorithms. *Annals of Applied Probability*, 7(1):110–120. 437

Rubin, D. B. (1981). Estimation in parallel randomized experiments. *Journal of Educational Statistics*, 6:377–401. 95

Schäling, B. (2011). The Boost C++ libraries. `http://en.highscore.de/cpp/boost/`.

Schofield, M. R. (2007). *Hierarchical Capture-Recapture Models*. PhD thesis, Department of of Statistics, University of Otago, Dunedin. 102, 104

Seber, G. A. F. (1965). A note on the multiple-recapture census. *Biometrika*, 52(1/2):249–259. 102

Smith, T. C., Spiegelhalter, D. J., and Thomas, A. (1995). Bayesian approaches to random-effects meta-analysis: a comparative study. *Statistics in Medicine*, 14(24):2685–2699. 95

Swendsen, R. H. and Wang, J.-S. (1986). Replica Monte Carlo simulation of spin glasses. *Physical Review Letters*, 57:2607–2609. 173

Tibshirani, R. (1996). Regression shrinkage and selection via the lasso. *Journal of the Royal Statistical Society, Series B*, 58(1):267–288. 395

Tokuda, T., Goodrich, B., Van Mechelen, I., Gelman, A., and Tuerlinckx, F. (2010). Visualizing distributions of covariance matrices. Technical report, Columbia University, Department of Statistics.

van Heesch, D. (2011). Doxygen: Generate documentation from source code. `http://www.stack.nl/~dimitri/doxygen/index.html`.

Warn, D. E., Thompson, S. G., and Spiegelhalter, D. J. (2002). Bayesian random effects meta-analysis of trials with binary outcomes: methods for the absolute risk difference and relative risk scales. *Statistics in Medicine*, 21:1601–1623. 93, 95

Zou, H. and Hastie, T. (2005). Regularization and variable selection via the elastic net. *Journal of the Royal Statistical Society, Series B*, 67(2):301–320. 395, 396

Zyczkowski, K. and Sommers, H. (2001). Induced measures in the space of mixed quantum states. *Journal of Physics A: Mathematical and General*, 34(35):7111. 191

Index

abs
(int *x*): int, 287
(real *x*): real, 296
acos
(real *x*): real, 301
acosh
(real *x*): real, 302
append_col
(matrix *x*, matrix *y*): matrix, 330
(matrix *x*, vector *y*): matrix, 330
(row_vector *x*, row_vector *y*): row_vector, 330
(vector *x*, matrix *y*): matrix, 330
(vector *x*, vector *y*): matrix, 330
append_row
(matrix *x*, matrix *y*): matrix, 330
(matrix *x*, row_vector *y*): matrix, 330
(row_vector *x*, matrix *y*): matrix, 330
(row_vector *x*, row_vector *y*): matrix, 330
(vector *x*, vector *y*): vector, 330
asin
(real *x*): real, 301
asinh
(real *x*): real, 302
atan
(real *x*): real, 301
atan2
(real *x*, real *y*): real, 301
atanh
(real *x*): real, 302
bernoulli
sampling statement, 339
bernoulli_ccdf_log
(ints *y*, reals *theta*): real, 339
bernoulli_cdf
(ints *y*, reals *theta*): real, 339
bernoulli_cdf_log
(ints *y*, reals *theta*): real, 339
bernoulli_log
(ints *y*, reals *theta*): real, 339
bernoulli_logit
sampling statement, 340
bernoulli_logit_log
(ints *y*, reals *alpha*): real, 340
bernoulli_rng
(real *theta*): int, 339
bessel_first_kind
(int *v*, real *x*): real, 306
bessel_second_kind
(int *v*, real *x*): real, 307
beta
sampling statement, 372
beta_binomial
sampling statement, 343
beta_binomial_ccdf_log
(ints *n*, ints *N*, reals *alpha*, reals *beta*): real, 344
beta_binomial_cdf
(ints *n*, ints *N*, reals *alpha*, reals *beta*): real, 343
beta_binomial_cdf_log
(ints *n*, ints *N*, reals *alpha*, reals *beta*): real, 344
beta_binomial_log
(ints *n*, ints *N*, reals *alpha*, reals *beta*): real, 343
beta_binomial_rng
(int *N*, real *alpha*, real *beta*): int, 344
beta_ccdf_log
(reals *theta*, reals *alpha*, reals *beta*): real, 372
beta_cdf
(reals *theta*, reals *alpha*, reals *beta*): real, 372
beta_cdf_log
(reals *theta*, reals *alpha*, reals *beta*): real, 372
beta_log
(reals *theta*, reals *alpha*, reals *beta*): real, 372
beta_rng
(real *alpha*, real *beta*): real, 373
binary_log_loss
(int *y*, real *y_hat*): real, 304
binomial
sampling statement, 341
binomial_ccdf_log
(ints *n*, ints *N*, reals *theta*): real, 342
binomial_cdf

(ints *n*, ints *N*, reals *theta*): real, 341
binomial_cdf_log
(ints *n*, ints *N*, reals *theta*): real, 342
binomial_coefficient_log
(real *x*, real *y*): real, 306
binomial_log
(ints *n*, ints *N*, reals *theta*): real, 341
binomial_logit
sampling statement, 343
binomial_logit_log
(ints *n*, ints *N*, reals *alpha*): real, 343
binomial_rng
(int *N*, real *theta*): int, 342
block
(matrix *x*, int *i*, int *j*, int *n_rows*, int *n_cols*): matrix, 328
categorical
sampling statement, 345
categorical_log
(ints *y*, vector *theta*): real, 345
categorical_logit
sampling statement, 345
categorical_logit_log
(ints *y*, vector *beta*): real, 345
categorical_rng
(vector *theta*): int, 345
cauchy
sampling statement, 357
cauchy_ccdf_log
(reals *y*, reals *mu*, reals *sigma*): real, 357
cauchy_cdf
(reals *y*, reals *mu*, reals *sigma*): real, 357
cauchy_cdf_log
(reals *y*, reals *mu*, reals *sigma*): real, 357
cauchy_log
(reals *y*, reals *mu*, reals *sigma*): real, 357
cauchy_rng
(real *mu*, real *sigma*): real, 357
cbrt
(real *x*): real, 298
ceil
(real *x*): real, 297
chi_square
sampling statement, 362
chi_square_ccdf_log
(reals *y*, reals *nu*): real, 362
chi_square_cdf
(reals *y*, reals *nu*): real, 362
chi_square_cdf_log
(reals *y*, reals *nu*): real, 362
chi_square_log
(reals *y*, reals *nu*): real, 362
chi_square_rng
(real *nu*): real, 362
cholesky_decompose
(matrix *A*): matrix, 334
col
(matrix *x*, int *n*): vector, 328
cols
(matrix *x*): int, 317
(row_vector *x*): int, 317
(vector *x*): int, 317
columns_dot_product
(matrix *x*, matrix *y*): row_vector, 322
(row_vector *x*, row_vector *y*): row_vector, 322
(vector *x*, vector *y*): row_vector, 322
columns_dot_self
(matrix *x*): row_vector, 323
(row_vector *x*): row_vector, 323
(vector *x*): row_vector, 323
cos
(real *x*): real, 300
cosh
(real *x*): real, 301
crossprod
(matrix *x*): matrix, 323
cumulative_sum
(real[] *x*): real[], 322
(row_vector *rv*): row_vector, 322
(vector *v*): vector, 322
determinant
(matrix *A*): real, 333
diag_matrix
(vector *x*): matrix, 328
diag_post_multiply
(matrix *m*, row_vector *rv*): matrix, 325
(matrix *m*, vector *v*): matrix, 324
diag_pre_multiply
(row_vector *rv*, matrix *m*): matrix, 324
(vector *v*, matrix *m*): matrix, 324
diagonal
(matrix *x*): vector, 328
digamma

(real *x*): real, 305
dims
(*T x*): int[], 313
dirichlet
sampling statement, 384
dirichlet_log
(vector *theta*, vector *alpha*): real, 384
dirichlet_rng
(vector *alpha*): vector, 384
distance
(row_vector *x*, row_vector *y*): real, 313
(row_vector *x*, vector *y*): real, 312
(vector *x*, row_vector *y*): real, 312
(vector *x*, vector *y*): real, 312
dot_product
(row_vector *x*, row_vector *y*): real, 322
(row_vector *x*, vector *y*): real, 322
(vector *x*, row_vector *y*): real, 322
(vector *x*, vector *y*): real, 322
dot_self
(row_vector *x*): real, 323
(vector *x*): real, 323
double_exponential
sampling statement, 358
double_exponential_ccdf_log
(reals *y*, reals *mu*, reals *sigma*): real, 358
double_exponential_cdf
(reals *y*, reals *mu*, reals *sigma*): real, 358
double_exponential_cdf_log
(reals *y*, reals *mu*, reals *sigma*): real, 358
double_exponential_log
(reals *y*, reals *mu*, reals *sigma*): real, 358
double_exponential_rng
(real *mu*, real *sigma*): real, 358
e
(): real, 289
eigenvalues_sym
(matrix *A*): vector, 333
eigenvectors_sym
(matrix *A*): matrix, 333
erf
(real *x*): real, 303
erfc
(real *x*): real, 303
exp
(matrix *x*): matrix, 321
(real *x*): real, 298
(row_vector *x*): row_vector, 321
(vector *x*): vector, 321
exp2
(real *x*): real, 299
exp_mod_normal
sampling statement, 354
exp_mod_normal_ccdf_log
(reals *y*, reals *mu*, reals *sigma* reals *lambda*): real, 354
exp_mod_normal_cdf
(reals *y*, reals *mu*, reals *sigma* reals *lambda*): real, 354
exp_mod_normal_cdf_log
(reals *y*, reals *mu*, reals *sigma* reals *lambda*): real, 354
exp_mod_normal_log
(reals *y*, reals *mu*, reals *sigma* reals *lambda*): real, 354
exp_mod_normal_rng
(real *mu*, real *sigma*, real *lambda*): real, 354
expm1
(real *x*): real, 308
exponential
sampling statement, 364
exponential_ccdf_log
(reals *y*, reals *beta*): real, 365
exponential_cdf
(reals *y*, reals *beta*): real, 365
exponential_cdf_log
(reals *y*, reals *beta*): real, 365
exponential_log
(reals *y*, reals *beta*): real, 364
exponential_rng
(real *beta*): real, 365
fabs
(real *x*): real, 296
falling_factorial
(real *x*, real *n*): real, 307
fdim
(real *x*, real *y*): real, 296
floor
(real *x*): real, 297
fma
(real *x*, real *y*, real *z*): real, 308
fmax
(real *x*, real *y*): real, 297
fmin

(real *x*, real *y*): real, 296
fmod
(real *x*, real *y*): real, 297
frechet
sampling statement, 368
frechet_ccdf_log
(reals *y*, reals *alpha*, reals *sigma*): real, 368
frechet_cdf
(reals *y*, reals *alpha*, reals *sigma*): real, 368
frechet_cdf_log
(reals *y*, reals *alpha*, reals *sigma*): real, 368
frechet_log
(reals *y*, reals *alpha*, reals *sigma*): real, 368
frechet_rng
(real *alpha*, real *sigma*): real, 368
gamma
sampling statement, 365
gamma_ccdf_log
(reals *y*, reals *alpha*, reals *beta*): real, 366
gamma_cdf
(reals *y*, reals *alpha*, reals *beta*): real, 365
gamma_cdf_log
(reals *y*, reals *alpha*, reals *beta*): real, 365
gamma_log
(reals *y*, reals *alpha*, reals *beta*): real, 365
gamma_p
(real *a*, real *z*): real, 306
gamma_q
(real *a*, real *z*): real, 306
gamma_rng
(real *alpha*, real *beta*): real, 366
gaussian_dlm_obs
sampling statement, 382
gaussian_dlm_obs_log
(matrix *y*, matrix *F*, matrix *G*, matrix *V* matrix *W*, vector *m0*, matrix *C0*): real, 383
(matrix *y*, matrix *F*, matrix *G*, vector *V* matrix *W*, vector *m0*, matrix *C0*): real, 383
get_lp
(): real, 290
gumbel
sampling statement, 359
gumbel_ccdf_log
(reals *y*, reals *mu*, reals *beta*): real, 360
gumbel_cdf
(reals *y*, reals *mu*, reals *beta*): real, 360
gumbel_cdf_log
(reals *y*, reals *mu*, reals *beta*): real, 360
gumbel_log
(reals *y*, reals *mu*, reals *beta*): real, 359
gumbel_rng
(real *mu*, real *beta*): real, 360
head
(T[] *sv*, int *n*): T[], 329
(row_vector *rv*, int *n*): row_vector, 329
(vector *v*, int *n*): vector, 329
hypergeometric
sampling statement, 344
hypergeometric_log
(int *n*, int *N*, int *a*, int *b*): real, 344
hypergeometric_rng
(int *N*, real *a*, real *b*): int, 344
hypot
(real *x*, real *y*): real, 300
if_else
(int *cond*, real *x*, real *y*): real, 293
increment_log_prob
(T *lp*): void, 284
int_step
(int *x*): int, 287
(real *x*): int, 287
inv
(real *x*): real, 299
inv_chi_square
sampling statement, 363
inv_chi_square_ccdf_log
(reals *y*, reals *nu*): real, 363
inv_chi_square_cdf
(reals *y*, reals *nu*): real, 363
inv_chi_square_cdf_log
(reals *y*, reals *nu*): real, 363
inv_chi_square_log
(reals *y*, reals *nu*): real, 363
inv_chi_square_rng
(real *nu*): real, 363
inv_cloglog

(real *y*): real, 303
inv_gamma
sampling statement, 366
inv_gamma_ccdf_log
(reals *y*, reals *alpha*, reals *beta*): real, 366
inv_gamma_cdf
(reals *y*, reals *alpha*, reals *beta*): real, 366
inv_gamma_cdf_log
(reals *y*, reals *alpha*, reals *beta*): real, 366
inv_gamma_log
(reals *y*, reals *alpha*, reals *beta*): real, 366
inv_gamma_rng
(real *alpha*, real *beta*): real, 366
inv_logit
(real *y*): real, 303
inv_sqrt
(real *x*): real, 300
inv_square
(real *x*): real, 300
inv_wishart
sampling statement, 389
inv_wishart_log
(matrix *W*, real *nu*, matrix *Sigma*): real, 389
inv_wishart_rng
(real *nu*, matrix *Sigma*): matrix, 389
inverse
(matrix *A*): matrix, 333
inverse_spd
(matrix *A*): matrix, 333
is_inf
(real *x*): int, 294
is_nan
(real *x*): int, 294
lbeta
(real *alpha*, real *beta*): real, 304
lgamma
(real *x*): real, 305
lkj_corr
sampling statement, 386
lkj_corr_cholesky
sampling statement, 387
lkj_corr_cholesky_log
(matrix *L*, real *eta*): real, 387
lkj_corr_cholesky_rng
(int *K*, real *eta*): matrix, 387
lkj_corr_log
(matrix *y*, real *eta*): real, 386
lkj_corr_rng
(int *K*, real *eta*): matrix, 386
lmgamma
(int *n*, real *x*): real, 305
log
(matrix *x*): matrix, 321
(real *x*): real, 299
(row_vector *x*): row_vector, 321
(vector *x*): vector, 321
log10
(): real, 289
(real *x*): real, 299
log1m
(real *x*): real, 309
log1m_exp
(real *x*): real, 309
log1m_inv_logit
(real *x*): real, 310
log1p
(real *x*): real, 309
log1p_exp
(real *x*): real, 309
log2
(): real, 289
(real *x*): real, 299
log_determinant
(matrix *A*): real, 333
log_diff_exp
(real *x*, real *y*): real, 309
log_falling_factorial
(real *x*, real *n*): real, 308
log_inv_logit
(real *x*): real, 310
log_rising_factorial
(real *x*, real *n*): real, 308
log_softmax
(vector *x*): vector, 331
log_sum_exp
(matrix *x*): real, 325
(real *x*, real *y*): real, 310
(real *x*[]): real, 311
(row_vector *x*): real, 325
(vector *x*): real, 325
logistic
sampling statement, 358
logistic_ccdf_log
(reals *y*, reals *mu*, reals *sigma*): real, 359
logistic_cdf

(reals *y*, reals *mu*, reals *sigma*): real, 359
logistic_cdf_log
(reals *y*, reals *mu*, reals *sigma*): real, 359
logistic_log
(reals *y*, reals *mu*, reals *sigma*): real, 359
logistic_rng
(real *mu*, real *sigma*): real, 359
logit
(real *x*): real, 302
lognormal
sampling statement, 361
lognormal_ccdf_log
(reals *y*, reals *mu*, reals *sigma*): real, 361
lognormal_cdf
(reals *y*, reals *mu*, reals *sigma*): real, 361
lognormal_cdf_log
(reals *y*, reals *mu*, reals *sigma*): real, 361
lognormal_log
(reals *y*, reals *mu*, reals *sigma*): real, 361
lognormal_rng
(real *mu*, real *beta*): real, 361
machine_precision
(): real, 289
max
(int *x*, int *y*): int, 288
(int *x*[]): int, 311
(matrix *x*): real, 325
(real *x*[]): real, 311
(row_vector *x*): real, 325
(vector *x*): real, 325
mdivide_left_tri_low
(matrix *A*, matrix *B*): matrix, 332
(matrix *A*, vector *B*): vector, 332
mdivide_right_tri_low
(matrix *B*, matrix *A*): matrix, 332
(row_vector *B*, matrix *A*): row_vector, 332
mean
(matrix *x*): real, 326
(real *x*[]): real, 312
(row_vector *x*): real, 326
(vector *x*): real, 326
min
(int *x*, int *y*): int, 288
(int *x*[]): int, 311
(matrix *x*): real, 325
(real *x*[]): real, 311
(row_vector *x*): real, 325
(vector *x*): real, 325
modified_bessel_first_kind
(int *v*, real *z*): real, 307
modified_bessel_second_kind
(int *v*, real *z*): real, 307
multi_gp
sampling statement, 380
multi_gp_cholesky
sampling statement, 381
multi_gp_cholesky_log
(matrix *y*, matrix *L*, vector *w*): real, 381
multi_gp_log
(matrix *y*, matrix *Sigma*, vector *w*): real, 380
multi_normal
sampling statement, 377
multi_normal_cholesky
sampling statement, 379
multi_normal_cholesky_log
(row_vectors *y*, row_vectors *mu*, matrix *L*): real, 379
(row_vectors *y*, vectors *mu*, matrix *L*): real, 379
(vectors *y*, row_vectors *mu*, matrix *L*): real, 379
(vectors *y*, vectors *mu*, matrix *L*): real, 379
multi_normal_cholesky_rng
(vector *mu*, matrix *L*): vector, 379
multi_normal_log
(row_vectors *y*, row_vectors *mu*, matrix *Sigma*): real, 378
(row_vectors *y*, vectors *mu*, matrix *Sigma*): real, 377
(vectors *y*, row_vectors *mu*, matrix *Sigma*): real, 377
(vectors *y*, vectors *mu*, matrix *Sigma*): real, 377
multi_normal_prec
sampling statement, 378
multi_normal_prec_log
(row_vectors *y*, row_vectors *mu*, matrix *Omega*): real, 378
(row_vectors *y*, vectors *mu*, matrix *Omega*): real, 378

(vectors *y*, row_vectors *mu*, matrix *Omega*): real, 378
(vectors *y*, vectors *mu*, matrix *Omega*): real, 378
multi_normal_rng
(vector *mu*, matrix *Sigma*): vector, 378
multi_student_t
sampling statement, 381
multi_student_t_log
(row_vectors *y*, real *nu*, row_vectors *mu*, matrix *Sigma*): real, 382
(row_vectors *y*, real *nu*, vectors *mu*, matrix *Sigma*): real, 382
(vectors *y*, real *nu*, row_vectors *mu*, matrix *Sigma*): real, 381
(vectors *y*, real *nu*, vectors *mu*, matrix *Sigma*): real, 381
multi_student_t_rng
(real *nu*, vector *mu*, matrix *Sigma*): vector, 382
multinomial
sampling statement, 351
multinomial_log
(int[] *y*, vector *theta*): real, 351
multinomial_rng
(vector *theta*, int *N*): vector, 351
multiply_log
(real *x*, real *y*): real, 309
multiply_lower_tri_self_transpose
(matrix *x*): matrix, 324
neg_binomial
sampling statement, 347
neg_binomial_2
sampling statement, 348
neg_binomial_2_log
(ints *y*, reals *mu*, reals *phi*): real, 349
sampling statement, 348
neg_binomial_2_log_log
(ints *y*, reals *eta*, reals *phi*): real, 349
neg_binomial_2_log_rng
(real *eta*, real *phi*): int, 349
neg_binomial_2_rng
(real *mu*, real *phi*): int, 349
neg_binomial_ccdf_log
(ints *n*, reals *alpha*, reals *beta*): real, 348
neg_binomial_cdf
(ints *n*, reals *alpha*, reals *beta*): real, 347
neg_binomial_cdf_log
(ints *n*, reals *alpha*, reals *beta*): real, 347
neg_binomial_log
(ints *n*, reals *alpha*, reals *beta*): real, 347
neg_binomial_rng
(real *alpha*, real *beta*): int, 348
negative_infinity
(): real, 289
normal
sampling statement, 353
normal_ccdf_log
(reals *y*, reals *mu*, reals *sigma*): real, 353
normal_cdf
(reals *y*, reals *mu*, reals *sigma*): real, 353
normal_cdf_log
(reals *y*, reals *mu*, reals *sigma*): real, 353
normal_log
(reals *y*, reals *mu*, reals *sigma*): real, 353
normal_rng
(real *mu*, real *sigma*): real, 354
not_a_number
(): real, 289
num_elements
(matrix *x*): int, 317
(row_vector *x*): int, 317
(vector *x*): int, 317
(*T*[] *x*): int, 313
operator!
(int *x*): int, 292
(real *x*): int, 292
operator!=
(int *x*, int *y*): int, 291
(real *x*, real *y*): int, 292
operator'
(matrix *x*): matrix, 331
(row_vector *x*): vector, 331
(vector *x*): row_vector, 331
operator*
(int *x*, int *y*): int, 287
(matrix *x*, matrix *y*): matrix, 319
(matrix *x*, real *y*): matrix, 319
(matrix *x*, vector *y*): vector, 319
(real *x*, matrix *y*): matrix, 318
(real *x*, real *y*): real, 295

(real *x*, row_vector *y*): row_vector, 318
(real *x*, vector *y*): vector, 318
(row_vector *x*, matrix *y*): row_vector, 319
(row_vector *x*, real *y*): row_vector, 319
(row_vector *x*, vector *y*): real, 319
(vector *x*, real *y*): vector, 318
(vector *x*, row_vector *y*): matrix, 319

operator+
(int *x*): int, 287
(int *x*, int *y*): int, 287
(matrix *x*, matrix *y*): matrix, 318
(matrix *x*, real *y*): matrix, 319
(real *x*): real, 295
(real *x*, matrix *y*): matrix, 319
(real *x*, real *y*): real, 294
(real *x*, row_vector *y*): row_vector, 319
(real *x*, vector *y*): vector, 319
(row_vector *x*, real *y*): row_vector, 319
(row_vector *x*, row_vector *y*): row_vector, 318
(vector *x*, real *y*): vector, 319
(vector *x*, vector *y*): vector, 318

operator-
(int *x*): int, 287
(int *x*, int *y*): int, 287
(matrix *x*): matrix, 318
(matrix *x*, matrix *y*): matrix, 318
(matrix *x*, real *y*): matrix, 320
(real *x*): real, 295
(real *x*, matrix *y*): matrix, 320
(real *x*, real *y*): real, 295
(real *x*, row_vector *y*): row_vector, 320
(real *x*, vector *y*): vector, 320
(row_vector *x*): row_vector, 318
(row_vector *x*, real *y*): row_vector, 320
(row_vector *x*, row_vector *y*): row_vector, 318
(vector *x*): vector, 318
(vector *x*, real *y*): vector, 319
(vector *x*, vector *y*): vector, 318

operator.*
(matrix *x*, matrix *y*): matrix, 320
(row_vector *x*, row_vector *y*): row_vector, 320
(vector *x*, vector *y*): vector, 320

operator./
(matrix *x*, matrix *y*): matrix, 321
(matrix *x*, real *y*): matrix, 321
(real *x*, matrix *y*): matrix, 321
(real *x*, row_vector *y*): row_vector, 321
(real *x*, vector *y*): vector, 321
(row_vector *x*, real *y*): row_vector, 321
(row_vector *x*, row_vector *y*): row_vector, 321
(vector *x*, real *y*): vector, 320
(vector *x*, vector *y*): vector, 320

operator/
(int *x*, int *y*): int, 287
(matrix *B*, matrix *A*): matrix, 331
(matrix *x*, real *y*): matrix, 320
(real *x*, real *y*): real, 295
(row_vector *b*, matrix *A*): row_vector, 331
(row_vector *x*, real *y*): row_vector, 320
(vector *x*, real *y*): vector, 320

operator<
(int *x*, int *y*): int, 290
(real *x*, real *y*): int, 291

operator<=
(int *x*, int *y*): int, 291
(real *x*, real *y*): int, 291

operator>
(int *x*, int *y*): int, 291
(real *x*, real *y*): int, 291

operator>=
(int *x*, int *y*): int, 291
(real *x*, real *y*): int, 291

operator^
(real *x*, real *y*): real, 295

operator
(matrix *A*, matrix *B*): matrix, 332
(matrix *A*, vector *b*): vector, 332

operator==
(int *x*, int *y*): int, 291
(real *x*, real *y*): int, 291

operator&&
(int *x*, int *y*): int, 292
(real *x*, real *y*): int, 292

operator||
(int *x*, int *y*): int, 292
(real *x*, real *y*): int, 292

ordered_logistic

sampling statement, 346
ordered_logistic_log
(int *k*, real *eta*, vector *c*): real, 346
ordered_logistic_rng
(real *eta*, vector *c*): int, 346
owens_t
(real *h*, real *a*): real, 304
pareto
sampling statement, 370
pareto_ccdf_log
(reals *y*, reals *y_min*, reals *alpha*): real, 370
pareto_cdf
(reals *y*, reals *y_min*, reals *alpha*): real, 370
pareto_cdf_log
(reals *y*, reals *y_min*, reals *alpha*): real, 370
pareto_log
(reals *y*, reals *y_min*, reals *alpha*): real, 370
pareto_rng
(real *y_min*, real *alpha*): real, 370
pareto_type_2
sampling statement, 371
pareto_type_2_ccdf_log
(reals *y*, reals *mu*, reals *lambda*, reals *alpha*): real, 371
pareto_type_2_cdf
(reals *y*, reals *mu*, reals *lambda*, reals *alpha*): real, 371
pareto_type_2_cdf_log
(reals *y*, reals *mu*, reals *lambda*, reals *alpha*): real, 371
pareto_type_2_log
(reals *y*, reals *mu*, reals *lambda*, reals *alpha*): real, 371
pareto_type_2_rng
(real *mu*, real *lambda*, real *alpha*): real, 371
Phi
(real *x*): real, 303
Phi_approx
(real *x*): real, 304
pi
(): real, 289
poisson
sampling statement, 349
poisson_ccdf_log
(ints *n*, reals *lambda*): real, 350
poisson_cdf
(ints *n*, reals *lambda*): real, 349
poisson_cdf_log
(ints *n*, reals *lambda*): real, 349
poisson_log
(ints *n*, reals *lambda*): real, 349
sampling statement, 350
poisson_log_log
(ints *n*, reals *alpha*): real, 350
poisson_rng
(real *lambda*): int, 350
positive_infinity
(): real, 289
pow
(real *x*, real *y*): real, 299
print
(T1 *x1*,..., TN *xN*): void, 285
prod
(int *x*[]): real, 311
(matrix *x*): real, 326
(real *x*[]): real, 311
(row_vector *x*): real, 326
(vector *x*): real, 326
qr_Q
(matrix *A*): matrix, 333
qr_R
(matrix *A*): matrix, 334
quad_form
(matrix *A*, matrix *B*): matrix, 323
(matrix *A*, vector *B*): real, 324
quad_form_diag
(matrix *m*, row_vector *rv*): matrix, 324
(matrix *m*, vector *v*): matrix, 324
quad_form_sym
(matrix *A*, matrix *B*): matrix, 324
(matrix *A*, vector *B*): real, 324
rank
(int[] *v*, int *s*): int, 316
(real[] *v*, int *s*): int, 316
(row_vector *v*, int *s*): int, 335
(vector *v*, int *s*): int, 335
rayleigh
sampling statement, 369
rayleigh_ccdf_log
(real *y*, real *sigma*): real, 369
rayleigh_cdf
(real *y*, real *sigma*): real, 369
rayleigh_cdf_log
(real *y*, real *sigma*): real, 369
rayleigh_log
(reals *y*, reals *sigma*): real, 369
rayleigh_rng

(real *sigma*): real, 369
rep_array
(*T x*, int *k*, int *m*, int *n*): *T*[, ,], 314
(*T x*, int *m*, int *n*): *T*[,], 314
(*T x*, int *n*): *T*[], 314
rep_matrix
(real *x*, int *m*, int *n*): matrix, 327
(row_vector *rv*, int *m*): matrix, 327
(vector *v*, int *n*): matrix, 327
rep_row_vector
(real *x*, int *n*): row_vector, 327
rep_vector
(real *x*, int *m*): vector, 327
rising_factorial
(real *x*, real *n*): real, 308
round
(real *x*): real, 298
row
(matrix *x*, int *m*): row_vector, 328
rows
(matrix *x*): int, 317
(row_vector *x*): int, 317
(vector *x*): int, 317
rows_dot_product
(matrix *x*, matrix *y*): vector, 323
(row_vector *x*, row_vector *y*): vector, 322
(vector *x*, vector *y*): vector, 322
rows_dot_self
(matrix *x*): vector, 323
(row_vector *x*): vector, 323
(vector *x*): vector, 323
scaled_inv_chi_square
sampling statement, 363
scaled_inv_chi_square_ccdf_log
(reals *y*, reals *nu*, reals *sigma*): real, 364
scaled_inv_chi_square_cdf
(reals *y*, reals *nu*, reals *sigma*): real, 364
scaled_inv_chi_square_cdf_log
(reals *y*, reals *nu*, reals *sigma*): real, 364
scaled_inv_chi_square_log
(reals *y*, reals *nu*, reals *sigma*): real, 364
scaled_inv_chi_square_rng
(real *nu*, real *sigma*): real, 364
sd
(matrix *x*): real, 327
(real *x*[]): real, 312
(row_vector *x*): real, 326
(vector *x*): real, 326
segment
(T[] *sv*, int *i*, int *n*): T[], 329
(row_vector *v*, int *i*, int *n*): row_vector, 329
(vector *v*, int *i*, int *n*): vector, 329
sin
(real *x*): real, 300
singular_values
(matrix *A*): vector, 334
sinh
(real *x*): real, 301
size
(*T*[] *x*): int, 314
skew_normal
sampling statement, 355
skew_normal_ccdf_log
(reals *y*, reals *mu*, reals *sigma* reals *alpha*): real, 355
skew_normal_cdf
(reals *y*, reals *mu*, reals *sigma*, reals *alpha*): real, 355
skew_normal_cdf_log
(reals *y*, reals *mu*, reals *sigma* reals *alpha*): real, 355
skew_normal_log
(reals *y*, reals *mu*, reals *sigma*, reals *alpha*): real, 355
skew_normal_rng
(real *mu*, real *sigma*, real *alpha*): real, 355
softmax
(vector *x*): vector, 331
sort_asc
(int[] *v*): int[], 315
(real[] *v*): real[], 315
(row_vector *v*): row_vector, 334
(vector *v*): vector, 334
sort_desc
(int[] *v*): int[], 316
(real[] *v*): real[], 315
(row_vector *v*): row_vector, 335
(vector *v*): vector, 335
sort_indices_asc
(int[] *v*): int[], 316
(real[] *v*): int[], 316
(row_vector *v*): int[], 335
(vector *v*): int[], 335
sort_indices_desc

(int[] *v*): int[], 316
(real[] *v*): int[], 316
(row_vector *v*): int[], 335
(vector *v*): int[], 335
sqrt
(real *x*): real, 298
sqrt2
(): real, 289
square
(real *x*): real, 298
squared_distance
(row_vector *x*, row_vector *y*[]): real, 313
(row_vector *x*, vector *y*[]): real, 313
(vector *x*, row_vector *y*[]): real, 313
(vector *x*, vector *y*[]): real, 313
step
(real *x*): real, 293
student_t
sampling statement, 356
student_t_ccdf_log
(reals *y*, reals *nu*, reals *mu*, reals *sigma*): real, 356
student_t_cdf
(reals *y*, reals *nu*, reals *mu*, reals *sigma*): real, 356
student_t_cdf_log
(reals *y*, reals *nu*, reals *mu*, reals *sigma*): real, 356
student_t_log
(reals *y*, reals *nu*, reals *mu*, reals *sigma*): real, 356
student_t_rng
(real *nu*, real *mu*, real *sigma*): real, 356
sub_col
(matrix *x*, int *i*, int *j*, int *n_rows*): vector, 328
sub_row
(matrix *x*, int *i*, int *j*, int *n_cols*): row_vector, 328
sum
(int *x*[]): int, 311
(matrix *x*): real, 326
(real *x*[]): real, 311
(row_vector *x*): real, 326
(vector *x*): real, 325
tail
(T[] *sv*, int *n*): T[], 329
(row_vector *rv*, int *n*): row_vector, 329
(vector *v*, int *n*): vector, 329
tan
(real *x*): real, 300
tanh
(real *x*): real, 302
tcrossprod
(matrix *x*): matrix, 323
tgamma
(real *x*): real, 305
to_array_1d
(int[...] *a*): int[], 337
(matrix *m*): real[], 337
(real[...] *a*): real[], 337
(row_vector *v*): real[], 337
(vector *v*): real[], 337
to_array_2d
(matrix *m*): real[,], 337
to_matrix
(int[,] *a*): matrix, 336
(matrix *m*): matrix, 336
(real[,] *a*): matrix, 336
(row_vector *v*): matrix, 336
(vector *v*): matrix, 336
to_row_vector
(int[] *a*): row_vector, 337
(matrix *m*): row_vector, 337
(real[] *a*): row_vector, 337
(row_vector *v*): row_vector, 337
(vector *v*): row_vector, 337
to_vector
(int[] *a*): vector, 336
(matrix *m*): vector, 336
(real[] *a*): vector, 336
(row_vector *v*): vector, 336
(vector *v*): vector, 336
trace
(matrix *A*): real, 332
trace_gen_quad_form
(matrix *D*,matrix *A*, matrix *B*): real, 324
trace_quad_form
(matrix *A*, matrix *B*): real, 324
trigamma
(real *x*): real, 305
trunc
(real *x*): real, 298
uniform
sampling statement, 376
uniform_ccdf_log
(reals *y*, reals *alpha*, reals *beta*): real, 376

uniform_cdf
(reals *y*, reals *alpha*, reals *beta*): real, 376
uniform_cdf_log
(reals *y*, reals *alpha*, reals *beta*): real, 376
uniform_log
(reals *y*, reals *alpha*, reals *beta*): real, 376
uniform_rng
(real *alpha*, real *beta*): real, 376
variance
(matrix *x*): real, 326
(real *x*[]): real, 312
(row_vector *x*): real, 326
(vector *x*): real, 326
von_mises
sampling statement, 374
von_mises_log
(reals *y*, reals *mu*, reals *kappa*): real, 374
von_mises_rng
(reals *mu*, reals *kappa*): real, 374
weibull
sampling statement, 367
weibull_ccdf_log
(reals *y*, reals *alpha*, reals *sigma*): real, 367
weibull_cdf
(reals *y*, reals *alpha*, reals *sigma*): real, 367
weibull_cdf_log
(reals *y*, reals *alpha*, reals *sigma*): real, 367
weibull_log
(reals *y*, reals *alpha*, reals *sigma*): real, 367
weibull_rng
(real *alpha*, real *sigma*): real, 367
wishart
sampling statement, 388
wishart_log
(matrix *W*, real *nu*, matrix *Sigma*): real, 388
wishart_rng
(real *nu*, matrix *Sigma*): matrix, 388
